# Measurement of the inclusive jet cross-section in *pp* collisions at $\sqrt{s}=2.76\ \mbox{TeV}$ and comparison to the inclusive jet cross-section at $\sqrt{s} =7\ \mbox{TeV}$ using the ATLAS detector

**DOI:** 10.1140/epjc/s10052-013-2509-4

**Published:** 2013-08-03

**Authors:** The ATLAS Collaboration, G. Aad, T. Abajyan, B. Abbott, J. Abdallah, S. Abdel Khalek, A. A. Abdelalim, O. Abdinov, R. Aben, B. Abi, M. Abolins, O. S. AbouZeid, H. Abramowicz, H. Abreu, B. S. Acharya, L. Adamczyk, D. L. Adams, T. N. Addy, J. Adelman, S. Adomeit, P. Adragna, T. Adye, S. Aefsky, J. A. Aguilar-Saavedra, M. Agustoni, M. Aharrouche, S. P. Ahlen, F. Ahles, A. Ahmad, M. Ahsan, G. Aielli, T. P. A. Åkesson, G. Akimoto, A. V. Akimov, M. S. Alam, M. A. Alam, J. Albert, S. Albrand, M. Aleksa, I. N. Aleksandrov, F. Alessandria, C. Alexa, G. Alexander, G. Alexandre, T. Alexopoulos, M. Alhroob, M. Aliev, G. Alimonti, J. Alison, B. M. M. Allbrooke, P. P. Allport, S. E. Allwood-Spiers, J. Almond, A. Aloisio, R. Alon, A. Alonso, F. Alonso, A. Altheimer, B. Alvarez Gonzalez, M. G. Alviggi, K. Amako, C. Amelung, V. V. Ammosov, S. P. Amor Dos Santos, A. Amorim, N. Amram, C. Anastopoulos, L. S. Ancu, N. Andari, T. Andeen, C. F. Anders, G. Anders, K. J. Anderson, A. Andreazza, V. Andrei, M-L. Andrieux, X. S. Anduaga, S. Angelidakis, P. Anger, A. Angerami, F. Anghinolfi, A. V. Anisenkov, N. Anjos, A. Annovi, A. Antonaki, M. Antonelli, A. Antonov, J. Antos, F. Anulli, M. Aoki, S. Aoun, L. Aperio Bella, R. Apolle, G. Arabidze, I. Aracena, Y. Arai, A. T. H. Arce, S. Arfaoui, J-F. Arguin, S. Argyropoulos, E. Arik, M. Arik, A. J. Armbruster, O. Arnaez, V. Arnal, C. Arnault, A. Artamonov, G. Artoni, D. Arutinov, S. Asai, S. Ask, B. Åsman, L. Asquith, K. Assamagan, A. Astbury, M. Atkinson, B. Aubert, E. Auge, K. Augsten, M. Aurousseau, G. Avolio, R. Avramidou, D. Axen, G. Azuelos, Y. Azuma, M. A. Baak, G. Baccaglioni, C. Bacci, A. M. Bach, H. Bachacou, K. Bachas, M. Backes, M. Backhaus, J. Backus Mayes, E. Badescu, P. Bagnaia, S. Bahinipati, Y. Bai, D. C. Bailey, T. Bain, J. T. Baines, O. K. Baker, M. D. Baker, S. Baker, P. Balek, E. Banas, P. Banerjee, Sw. Banerjee, D. Banfi, A. Bangert, V. Bansal, H. S. Bansil, L. Barak, S. P. Baranov, A. Barbaro Galtieri, T. Barber, E. L. Barberio, D. Barberis, M. Barbero, D. Y. Bardin, T. Barillari, M. Barisonzi, T. Barklow, N. Barlow, B. M. Barnett, R. M. Barnett, A. Baroncelli, G. Barone, A. J. Barr, F. Barreiro, J. Barreiro Guimarães da Costa, P. Barrillon, R. Bartoldus, A. E. Barton, V. Bartsch, A. Basye, R. L. Bates, L. Batkova, J. R. Batley, A. Battaglia, M. Battistin, F. Bauer, H. S. Bawa, S. Beale, T. Beau, P. H. Beauchemin, R. Beccherle, P. Bechtle, H. P. Beck, K. Becker, S. Becker, M. Beckingham, K. H. Becks, A. J. Beddall, A. Beddall, S. Bedikian, V. A. Bednyakov, C. P. Bee, L. J. Beemster, M. Begel, S. Behar Harpaz, P. K. Behera, M. Beimforde, C. Belanger-Champagne, P. J. Bell, W. H. Bell, G. Bella, L. Bellagamba, M. Bellomo, A. Belloni, O. L. Beloborodova, K. Belotskiy, O. Beltramello, O. Benary, D. Benchekroun, K. Bendtz, N. Benekos, Y. Benhammou, E. Benhar Noccioli, J. A. Benitez Garcia, D. P. Benjamin, M. Benoit, J. R. Bensinger, K. Benslama, S. Bentvelsen, D. Berge, E. Bergeaas Kuutmann, N. Berger, F. Berghaus, E. Berglund, J. Beringer, P. Bernat, R. Bernhard, C. Bernius, T. Berry, C. Bertella, A. Bertin, F. Bertolucci, M. I. Besana, G. J. Besjes, N. Besson, S. Bethke, W. Bhimji, R. M. Bianchi, L. Bianchini, M. Bianco, O. Biebel, S. P. Bieniek, K. Bierwagen, J. Biesiada, M. Biglietti, H. Bilokon, M. Bindi, S. Binet, A. Bingul, C. Bini, B. Bittner, C. W. Black, K. M. Black, R. E. Blair, J.-B. Blanchard, G. Blanchot, T. Blazek, I. Bloch, C. Blocker, J. Blocki, A. Blondel, W. Blum, U. Blumenschein, G. J. Bobbink, V. S. Bobrovnikov, S. S. Bocchetta, A. Bocci, C. R. Boddy, M. Boehler, J. Boek, T. T. Boek, N. Boelaert, J. A. Bogaerts, A. G. Bogdanchikov, A. Bogouch, C. Bohm, J. Bohm, V. Boisvert, T. Bold, V. Boldea, N. M. Bolnet, M. Bomben, M. Bona, M. Bondioli, M. Boonekamp, S. Bordoni, C. Borer, A. Borisov, G. Borissov, I. Borjanovic, M. Borri, S. Borroni, J. Bortfeldt, V. Bortolotto, K. Bos, D. Boscherini, M. Bosman, H. Boterenbrood, J. Bouchami, J. Boudreau, E. V. Bouhova-Thacker, D. Boumediene, C. Bourdarios, N. Bousson, A. Boveia, J. Boyd, I. R. Boyko, I. Bozovic-Jelisavcic, J. Bracinik, P. Branchini, A. Brandt, G. Brandt, O. Brandt, U. Bratzler, B. Brau, J. E. Brau, H. M. Braun, S. F. Brazzale, B. Brelier, J. Bremer, K. Brendlinger, R. Brenner, S. Bressler, D. Britton, F. M. Brochu, I. Brock, R. Brock, F. Broggi, C. Bromberg, J. Bronner, G. Brooijmans, T. Brooks, W. K. Brooks, G. Brown, H. Brown, P. A. Bruckman de Renstrom, D. Bruncko, R. Bruneliere, S. Brunet, A. Bruni, G. Bruni, M. Bruschi, T. Buanes, Q. Buat, F. Bucci, J. Buchanan, P. Buchholz, R. M. Buckingham, A. G. Buckley, S. I. Buda, I. A. Budagov, B. Budick, L. Bugge, O. Bulekov, A. C. Bundock, M. Bunse, T. Buran, H. Burckhart, S. Burdin, T. Burgess, S. Burke, E. Busato, V. Büscher, P. Bussey, C. P. Buszello, B. Butler, J. M. Butler, C. M. Buttar, J. M. Butterworth, W. Buttinger, M. Byszewski, S. Cabrera Urbán, D. Caforio, O. Cakir, P. Calafiura, G. Calderini, P. Calfayan, R. Calkins, L. P. Caloba, R. Caloi, D. Calvet, S. Calvet, R. Camacho Toro, P. Camarri, D. Cameron, L. M. Caminada, R. Caminal Armadans, S. Campana, M. Campanelli, V. Canale, F. Canelli, A. Canepa, J. Cantero, R. Cantrill, L. Capasso, M. D. M. Capeans Garrido, I. Caprini, M. Caprini, D. Capriotti, M. Capua, R. Caputo, R. Cardarelli, T. Carli, G. Carlino, L. Carminati, B. Caron, S. Caron, E. Carquin, G. D. Carrillo-Montoya, A. A. Carter, J. R. Carter, J. Carvalho, D. Casadei, M. P. Casado, M. Cascella, C. Caso, A. M. Castaneda Hernandez, E. Castaneda-Miranda, V. Castillo Gimenez, N. F. Castro, G. Cataldi, P. Catastini, A. Catinaccio, J. R. Catmore, A. Cattai, G. Cattani, S. Caughron, V. Cavaliere, D. Cavalli, M. Cavalli-Sforza, V. Cavasinni, F. Ceradini, A. S. Cerqueira, A. Cerri, L. Cerrito, F. Cerutti, S. A. Cetin, A. Chafaq, D. Chakraborty, I. Chalupkova, K. Chan, P. Chang, B. Chapleau, J. D. Chapman, J. W. Chapman, E. Chareyre, D. G. Charlton, V. Chavda, C. A. Chavez Barajas, S. Cheatham, S. Chekanov, S. V. Chekulaev, G. A. Chelkov, M. A. Chelstowska, C. Chen, H. Chen, S. Chen, X. Chen, Y. Chen, Y. Cheng, A. Cheplakov, R. Cherkaoui El Moursli, V. Chernyatin, E. Cheu, S. L. Cheung, L. Chevalier, G. Chiefari, L. Chikovani, J. T. Childers, A. Chilingarov, G. Chiodini, A. S. Chisholm, R. T. Chislett, A. Chitan, M. V. Chizhov, G. Choudalakis, S. Chouridou, I. A. Christidi, A. Christov, D. Chromek-Burckhart, M. L. Chu, J. Chudoba, G. Ciapetti, A. K. Ciftci, R. Ciftci, D. Cinca, V. Cindro, A. Ciocio, M. Cirilli, P. Cirkovic, Z. H. Citron, M. Citterio, M. Ciubancan, A. Clark, P. J. Clark, R. N. Clarke, W. Cleland, J. C. Clemens, B. Clement, C. Clement, Y. Coadou, M. Cobal, A. Coccaro, J. Cochran, S. Coelli, L. Coffey, J. G. Cogan, J. Coggeshall, E. Cogneras, J. Colas, S. Cole, A. P. Colijn, N. J. Collins, C. Collins-Tooth, J. Collot, T. Colombo, G. Colon, G. Compostella, P. Conde Muiño, E. Coniavitis, M. C. Conidi, S. M. Consonni, V. Consorti, S. Constantinescu, C. Conta, G. Conti, F. Conventi, M. Cooke, B. D. Cooper, A. M. Cooper-Sarkar, K. Copic, T. Cornelissen, M. Corradi, F. Corriveau, A. Corso-Radu, A. Cortes-Gonzalez, G. Cortiana, G. Costa, M. J. Costa, D. Costanzo, D. Côté, L. Courneyea, G. Cowan, C. Cowden, B. E. Cox, K. Cranmer, S. Crépé-Renaudin, F. Crescioli, M. Cristinziani, G. Crosetti, C.-M. Cuciuc, C. Cuenca Almenar, T. Cuhadar Donszelmann, J. Cummings, M. Curatolo, C. J. Curtis, C. Cuthbert, P. Cwetanski, H. Czirr, P. Czodrowski, Z. Czyczula, S. D’Auria, M. D’Onofrio, A. D’Orazio, M. J. Da Cunha Sargedas De Sousa, C. Da Via, W. Dabrowski, A. Dafinca, T. Dai, C. Dallapiccola, M. Dam, M. Dameri, D. S. Damiani, H. O. Danielsson, V. Dao, G. Darbo, G. L. Darlea, J. A. Dassoulas, W. Davey, T. Davidek, N. Davidson, R. Davidson, E. Davies, M. Davies, O. Davignon, A. R. Davison, Y. Davygora, E. Dawe, I. Dawson, R. K. Daya-Ishmukhametova, K. De, R. de Asmundis, S. De Castro, S. De Cecco, J. de Graat, N. De Groot, P. de Jong, C. De La Taille, H. De la Torre, F. De Lorenzi, L. de Mora, L. De Nooij, D. De Pedis, A. De Salvo, U. De Sanctis, A. De Santo, J. B. De Vivie De Regie, G. De Zorzi, W. J. Dearnaley, R. Debbe, C. Debenedetti, B. Dechenaux, D. V. Dedovich, J. Degenhardt, J. Del Peso, T. Del Prete, T. Delemontex, M. Deliyergiyev, A. Dell’Acqua, L. Dell’Asta, M. Della Pietra, D. della Volpe, M. Delmastro, P. A. Delsart, C. Deluca, S. Demers, M. Demichev, B. Demirkoz, S. P. Denisov, D. Derendarz, J. E. Derkaoui, F. Derue, P. Dervan, K. Desch, E. Devetak, P. O. Deviveiros, A. Dewhurst, B. DeWilde, S. Dhaliwal, R. Dhullipudi, A. Di Ciaccio, L. Di Ciaccio, C. Di Donato, A. Di Girolamo, B. Di Girolamo, S. Di Luise, A. Di Mattia, B. Di Micco, R. Di Nardo, A. Di Simone, R. Di Sipio, M. A. Diaz, E. B. Diehl, J. Dietrich, T. A. Dietzsch, S. Diglio, K. Dindar Yagci, J. Dingfelder, F. Dinut, C. Dionisi, P. Dita, S. Dita, F. Dittus, F. Djama, T. Djobava, M. A. B. do Vale, A. Do Valle Wemans, T. K. O. Doan, M. Dobbs, D. Dobos, E. Dobson, J. Dodd, C. Doglioni, T. Doherty, T. Dohmae, Y. Doi, J. Dolejsi, I. Dolenc, Z. Dolezal, B. A. Dolgoshein, M. Donadelli, J. Donini, J. Dopke, A. Doria, A. Dos Anjos, A. Dotti, M. T. Dova, A. D. Doxiadis, A. T. Doyle, N. Dressnandt, M. Dris, J. Dubbert, S. Dube, E. Duchovni, G. Duckeck, D. Duda, A. Dudarev, F. Dudziak, I. P. Duerdoth, L. Duflot, M-A. Dufour, L. Duguid, M. Dührssen, M. Dunford, H. Duran Yildiz, M. Düren, M. Dwuznik, J. Ebke, S. Eckweiler, K. Edmonds, W. Edson, C. A. Edwards, N. C. Edwards, W. Ehrenfeld, T. Eifert, G. Eigen, K. Einsweiler, E. Eisenhandler, T. Ekelof, M. El Kacimi, M. Ellert, S. Elles, F. Ellinghaus, K. Ellis, N. Ellis, J. Elmsheuser, M. Elsing, D. Emeliyanov, R. Engelmann, A. Engl, J. Erdmann, A. Ereditato, D. Eriksson, J. Ernst, M. Ernst, J. Ernwein, D. Errede, S. Errede, E. Ertel, M. Escalier, H. Esch, C. Escobar, X. Espinal Curull, B. Esposito, F. Etienne, A. I. Etienvre, E. Etzion, D. Evangelakou, H. Evans, L. Fabbri, C. Fabre, R. M. Fakhrutdinov, S. Falciano, Y. Fang, M. Fanti, A. Farbin, A. Farilla, J. Farley, T. Farooque, S. Farrell, S. M. Farrington, P. Farthouat, F. Fassi, P. Fassnacht, D. Fassouliotis, B. Fatholahzadeh, A. Favareto, L. Fayard, S. Fazio, R. Febbraro, P. Federic, O. L. Fedin, W. Fedorko, M. Fehling-Kaschek, L. Feligioni, C. Feng, E. J. Feng, A. B. Fenyuk, J. Ferencei, W. Fernando, S. Ferrag, J. Ferrando, V. Ferrara, A. Ferrari, P. Ferrari, R. Ferrari, D. E. Ferreira de Lima, A. Ferrer, D. Ferrere, C. Ferretti, A. Ferretto Parodi, M. Fiascaris, F. Fiedler, A. Filipčič, F. Filthaut, M. Fincke-Keeler, M. C. N. Fiolhais, L. Fiorini, A. Firan, G. Fischer, M. J. Fisher, M. Flechl, I. Fleck, J. Fleckner, P. Fleischmann, S. Fleischmann, T. Flick, A. Floderus, L. R. Flores Castillo, M. J. Flowerdew, T. Fonseca Martin, A. Formica, A. Forti, D. Fortin, D. Fournier, H. Fox, P. Francavilla, M. Franchini, S. Franchino, D. Francis, T. Frank, M. Franklin, S. Franz, M. Fraternali, S. Fratina, S. T. French, C. Friedrich, F. Friedrich, R. Froeschl, D. Froidevaux, J. A. Frost, C. Fukunaga, E. Fullana Torregrosa, B. G. Fulsom, J. Fuster, C. Gabaldon, O. Gabizon, T. Gadfort, S. Gadomski, G. Gagliardi, P. Gagnon, C. Galea, B. Galhardo, E. J. Gallas, V. Gallo, B. J. Gallop, P. Gallus, K. K. Gan, Y. S. Gao, A. Gaponenko, F. Garberson, C. García, J. E. García Navarro, M. Garcia-Sciveres, R. W. Gardner, N. Garelli, V. Garonne, C. Gatti, G. Gaudio, B. Gaur, L. Gauthier, P. Gauzzi, I. L. Gavrilenko, C. Gay, G. Gaycken, E. N. Gazis, P. Ge, Z. Gecse, C. N. P. Gee, D. A. A. Geerts, Ch. Geich-Gimbel, K. Gellerstedt, C. Gemme, A. Gemmell, M. H. Genest, S. Gentile, M. George, S. George, A. Gershon, H. Ghazlane, N. Ghodbane, B. Giacobbe, S. Giagu, V. Giakoumopoulou, V. Giangiobbe, F. Gianotti, B. Gibbard, A. Gibson, S. M. Gibson, M. Gilchriese, D. Gillberg, A. R. Gillman, D. M. Gingrich, N. Giokaris, M. P. Giordani, R. Giordano, F. M. Giorgi, P. Giovannini, P. F. Giraud, D. Giugni, M. Giunta, B. K. Gjelsten, L. K. Gladilin, C. Glasman, J. Glatzer, A. Glazov, K. W. Glitza, G. L. Glonti, J. R. Goddard, J. Godfrey, J. Godlewski, M. Goebel, C. Goeringer, S. Goldfarb, T. Golling, A. Gomes, L. S. Gomez Fajardo, R. Gonçalo, J. Goncalves Pinto Firmino Da Costa, L. Gonella, S. González de la Hoz, G. Gonzalez Parra, M. L. Gonzalez Silva, S. Gonzalez-Sevilla, J. J. Goodson, L. Goossens, T. Göpfert, P. A. Gorbounov, H. A. Gordon, I. Gorelov, G. Gorfine, B. Gorini, E. Gorini, A. Gorišek, E. Gornicki, A. T. Goshaw, M. Gosselink, C. Gössling, M. I. Gostkin, I. Gough Eschrich, M. Gouighri, D. Goujdami, M. P. Goulette, A. G. Goussiou, C. Goy, S. Gozpinar, I. Grabowska-Bold, P. Grafström, K-J. Grahn, E. Gramstad, F. Grancagnolo, S. Grancagnolo, V. Grassi, V. Gratchev, N. Grau, H. M. Gray, J. A. Gray, E. Graziani, O. G. Grebenyuk, T. Greenshaw, Z. D. Greenwood, K. Gregersen, I. M. Gregor, P. Grenier, J. Griffiths, N. Grigalashvili, A. A. Grillo, S. Grinstein, Ph. Gris, Y. V. Grishkevich, J.-F. Grivaz, E. Gross, J. Grosse-Knetter, J. Groth-Jensen, K. Grybel, D. Guest, C. Guicheney, E. Guido, S. Guindon, U. Gul, J. Gunther, B. Guo, J. Guo, P. Gutierrez, N. Guttman, O. Gutzwiller, C. Guyot, C. Gwenlan, C. B. Gwilliam, A. Haas, S. Haas, C. Haber, H. K. Hadavand, D. R. Hadley, P. Haefner, F. Hahn, Z. Hajduk, H. Hakobyan, D. Hall, K. Hamacher, P. Hamal, K. Hamano, M. Hamer, A. Hamilton, S. Hamilton, L. Han, K. Hanagaki, K. Hanawa, M. Hance, C. Handel, P. Hanke, J. R. Hansen, J. B. Hansen, J. D. Hansen, P. H. Hansen, P. Hansson, K. Hara, T. Harenberg, S. Harkusha, D. Harper, R. D. Harrington, O. M. Harris, J. Hartert, F. Hartjes, T. Haruyama, A. Harvey, S. Hasegawa, Y. Hasegawa, S. Hassani, S. Haug, M. Hauschild, R. Hauser, M. Havranek, C. M. Hawkes, R. J. Hawkings, A. D. Hawkins, T. Hayakawa, T. Hayashi, D. Hayden, C. P. Hays, H. S. Hayward, S. J. Haywood, S. J. Head, V. Hedberg, L. Heelan, S. Heim, B. Heinemann, S. Heisterkamp, L. Helary, C. Heller, M. Heller, S. Hellman, D. Hellmich, C. Helsens, R. C. W. Henderson, M. Henke, A. Henrichs, A. M. Henriques Correia, S. Henrot-Versille, C. Hensel, T. Henß, C. M. Hernandez, Y. Hernández Jiménez, R. Herrberg-Schubert, G. Herten, R. Hertenberger, L. Hervas, G. G. Hesketh, N. P. Hessey, E. Higón-Rodriguez, J. C. Hill, K. H. Hiller, S. Hillert, S. J. Hillier, I. Hinchliffe, E. Hines, M. Hirose, F. Hirsch, D. Hirschbuehl, J. Hobbs, N. Hod, M. C. Hodgkinson, P. Hodgson, A. Hoecker, M. R. Hoeferkamp, J. Hoffman, D. Hoffmann, J. I. Hofmann, M. Hohlfeld, M. Holder, S. O. Holmgren, T. Holy, J. L. Holzbauer, T. M. Hong, L. Hooft van Huysduynen, S. Horner, J-Y. Hostachy, S. Hou, A. Hoummada, J. Howard, J. Howarth, I. Hristova, J. Hrivnac, T. Hryn’ova, P. J. Hsu, S.-C. Hsu, D. Hu, Z. Hubacek, F. Hubaut, F. Huegging, A. Huettmann, T. B. Huffman, E. W. Hughes, G. Hughes, M. Huhtinen, M. Hurwitz, N. Huseynov, J. Huston, J. Huth, G. Iacobucci, G. Iakovidis, M. Ibbotson, I. Ibragimov, L. Iconomidou-Fayard, J. Idarraga, P. Iengo, O. Igonkina, Y. Ikegami, M. Ikeno, D. Iliadis, N. Ilic, T. Ince, P. Ioannou, M. Iodice, K. Iordanidou, V. Ippolito, A. Irles Quiles, C. Isaksson, M. Ishino, M. Ishitsuka, R. Ishmukhametov, C. Issever, S. Istin, A. V. Ivashin, W. Iwanski, H. Iwasaki, J. M. Izen, V. Izzo, B. Jackson, J. N. Jackson, P. Jackson, M. R. Jaekel, V. Jain, K. Jakobs, S. Jakobsen, T. Jakoubek, J. Jakubek, D. O. Jamin, D. K. Jana, E. Jansen, H. Jansen, J. Janssen, A. Jantsch, M. Janus, R. C. Jared, G. Jarlskog, L. Jeanty, I. Jen-La Plante, D. Jennens, P. Jenni, P. Jež, S. Jézéquel, M. K. Jha, H. Ji, W. Ji, J. Jia, Y. Jiang, M. Jimenez Belenguer, S. Jin, O. Jinnouchi, M. D. Joergensen, D. Joffe, M. Johansen, K. E. Johansson, P. Johansson, S. Johnert, K. A. Johns, K. Jon-And, G. Jones, R. W. L. Jones, T. J. Jones, P. M. Jorge, K. D. Joshi, J. Jovicevic, T. Jovin, X. Ju, C. A. Jung, R. M. Jungst, V. Juranek, P. Jussel, A. Juste Rozas, S. Kabana, M. Kaci, A. Kaczmarska, P. Kadlecik, M. Kado, H. Kagan, M. Kagan, E. Kajomovitz, S. Kalinin, L. V. Kalinovskaya, S. Kama, N. Kanaya, M. Kaneda, S. Kaneti, T. Kanno, V. A. Kantserov, J. Kanzaki, B. Kaplan, A. Kapliy, J. Kaplon, D. Kar, M. Karagounis, K. Karakostas, M. Karnevskiy, V. Kartvelishvili, A. N. Karyukhin, L. Kashif, G. Kasieczka, R. D. Kass, A. Kastanas, Y. Kataoka, E. Katsoufis, J. Katzy, V. Kaushik, K. Kawagoe, T. Kawamoto, G. Kawamura, M. S. Kayl, S. Kazama, V. F. Kazanin, M. Y. Kazarinov, R. Keeler, P. T. Keener, R. Kehoe, M. Keil, G. D. Kekelidze, J. S. Keller, M. Kenyon, O. Kepka, N. Kerschen, B. P. Kerševan, S. Kersten, K. Kessoku, J. Keung, F. Khalil-zada, H. Khandanyan, A. Khanov, D. Kharchenko, A. Khodinov, A. Khomich, T. J. Khoo, G. Khoriauli, A. Khoroshilov, V. Khovanskiy, E. Khramov, J. Khubua, H. Kim, S. H. Kim, N. Kimura, O. Kind, B. T. King, M. King, R. S. B. King, J. Kirk, A. E. Kiryunin, T. Kishimoto, D. Kisielewska, T. Kitamura, T. Kittelmann, K. Kiuchi, E. Kladiva, M. Klein, U. Klein, K. Kleinknecht, M. Klemetti, A. Klier, P. Klimek, A. Klimentov, R. Klingenberg, J. A. Klinger, E. B. Klinkby, T. Klioutchnikova, P. F. Klok, S. Klous, E.-E. Kluge, T. Kluge, P. Kluit, S. Kluth, E. Kneringer, E. B. F. G. Knoops, A. Knue, B. R. Ko, T. Kobayashi, M. Kobel, M. Kocian, P. Kodys, S. Koenig, F. Koetsveld, P. Koevesarki, T. Koffas, E. Koffeman, L. A. Kogan, S. Kohlmann, F. Kohn, Z. Kohout, T. Kohriki, T. Koi, H. Kolanoski, V. Kolesnikov, I. Koletsou, J. Koll, A. A. Komar, Y. Komori, T. Kondo, K. Köneke, A. C. König, T. Kono, A. I. Kononov, R. Konoplich, N. Konstantinidis, R. Kopeliansky, S. Koperny, L. Köpke, K. Korcyl, K. Kordas, A. Korn, A. A. Korol, I. Korolkov, E. V. Korolkova, V. A. Korotkov, O. Kortner, S. Kortner, V. V. Kostyukhin, S. Kotov, V. M. Kotov, A. Kotwal, C. Kourkoumelis, V. Kouskoura, A. Koutsman, R. Kowalewski, T. Z. Kowalski, W. Kozanecki, A. S. Kozhin, V. Kral, V. A. Kramarenko, G. Kramberger, M. W. Krasny, A. Krasznahorkay, J. K. Kraus, S. Kreiss, F. Krejci, J. Kretzschmar, N. Krieger, P. Krieger, K. Kroeninger, H. Kroha, J. Kroll, J. Kroseberg, J. Krstic, U. Kruchonak, H. Krüger, T. Kruker, N. Krumnack, Z. V. Krumshteyn, M. K. Kruse, T. Kubota, S. Kuday, S. Kuehn, A. Kugel, T. Kuhl, D. Kuhn, V. Kukhtin, Y. Kulchitsky, S. Kuleshov, C. Kummer, M. Kuna, J. Kunkle, A. Kupco, H. Kurashige, M. Kurata, Y. A. Kurochkin, V. Kus, E. S. Kuwertz, M. Kuze, J. Kvita, R. Kwee, A. La Rosa, L. La Rotonda, L. Labarga, J. Labbe, S. Lablak, C. Lacasta, F. Lacava, J. Lacey, H. Lacker, D. Lacour, V. R. Lacuesta, E. Ladygin, R. Lafaye, B. Laforge, T. Lagouri, S. Lai, E. Laisne, L. Lambourne, C. L. Lampen, W. Lampl, E. Lançon, U. Landgraf, M. P. J. Landon, V. S. Lang, C. Lange, A. J. Lankford, F. Lanni, K. Lantzsch, A. Lanza, S. Laplace, C. Lapoire, J. F. Laporte, T. Lari, A. Larner, M. Lassnig, P. Laurelli, V. Lavorini, W. Lavrijsen, P. Laycock, O. Le Dortz, E. Le Guirriec, E. Le Menedeu, T. LeCompte, F. Ledroit-Guillon, H. Lee, J. S. H. Lee, S. C. Lee, L. Lee, M. Lefebvre, M. Legendre, F. Legger, C. Leggett, M. Lehmacher, G. Lehmann Miotto, A. G. Leister, M. A. L. Leite, R. Leitner, D. Lellouch, B. Lemmer, V. Lendermann, K. J. C. Leney, T. Lenz, G. Lenzen, B. Lenzi, K. Leonhardt, S. Leontsinis, F. Lepold, C. Leroy, J-R. Lessard, C. G. Lester, C. M. Lester, J. Levêque, D. Levin, L. J. Levinson, A. Lewis, G. H. Lewis, A. M. Leyko, M. Leyton, B. Li, B. Li, H. Li, H. L. Li, S. Li, X. Li, Z. Liang, H. Liao, B. Liberti, P. Lichard, M. Lichtnecker, K. Lie, W. Liebig, C. Limbach, A. Limosani, M. Limper, S. C. Lin, F. Linde, J. T. Linnemann, E. Lipeles, A. Lipniacka, T. M. Liss, D. Lissauer, A. Lister, A. M. Litke, C. Liu, D. Liu, H. Liu, J. B. Liu, L. Liu, M. Liu, Y. Liu, M. Livan, S. S. A. Livermore, A. Lleres, J. Llorente Merino, S. L. Lloyd, F. Lo Sterzo, E. Lobodzinska, P. Loch, W. S. Lockman, T. Loddenkoetter, F. K. Loebinger, A. E. Loevschall-Jensen, A. Loginov, C. W. Loh, T. Lohse, K. Lohwasser, M. Lokajicek, V. P. Lombardo, R. E. Long, L. Lopes, D. Lopez Mateos, J. Lorenz, N. Lorenzo Martinez, M. Losada, P. Loscutoff, M. J. Losty, X. Lou, A. Lounis, K. F. Loureiro, J. Love, P. A. Love, A. J. Lowe, F. Lu, H. J. Lubatti, C. Luci, A. Lucotte, A. Ludwig, D. Ludwig, I. Ludwig, J. Ludwig, F. Luehring, G. Luijckx, W. Lukas, L. Luminari, E. Lund, B. Lundberg, J. Lundberg, O. Lundberg, B. Lund-Jensen, J. Lundquist, M. Lungwitz, D. Lynn, E. Lytken, H. Ma, L. L. Ma, G. Maccarrone, A. Macchiolo, B. Maček, J. Machado Miguens, D. Macina, R. Mackeprang, R. J. Madaras, H. J. Maddocks, W. F. Mader, R. Maenner, M. Maeno, T. Maeno, L. Magnoni, E. Magradze, K. Mahboubi, J. Mahlstedt, S. Mahmoud, G. Mahout, C. Maiani, C. Maidantchik, A. Maio, S. Majewski, Y. Makida, N. Makovec, P. Mal, B. Malaescu, Pa. Malecki, P. Malecki, V. P. Maleev, F. Malek, U. Mallik, D. Malon, C. Malone, S. Maltezos, V. M. Malyshev, S. Malyukov, R. Mameghani, J. Mamuzic, L. Mandelli, I. Mandić, R. Mandrysch, J. Maneira, A. Manfredini, L. Manhaes de Andrade Filho, J. A. Manjarres Ramos, A. Mann, P. M. Manning, A. Manousakis-Katsikakis, B. Mansoulie, A. Mapelli, L. Mapelli, L. March, J. F. Marchand, F. Marchese, G. Marchiori, M. Marcisovsky, C. P. Marino, C. N. Marques, F. Marroquim, Z. Marshall, L. F. Marti, S. Marti-Garcia, B. Martin, B. Martin, J. P. Martin, T. A. Martin, V. J. Martin, B. Martin dit Latour, M. Martinez, V. Martinez Outschoorn, S. Martin-Haugh, A. C. Martyniuk, M. Marx, F. Marzano, A. Marzin, L. Masetti, T. Mashimo, R. Mashinistov, J. Masik, A. L. Maslennikov, I. Massa, G. Massaro, N. Massol, P. Mastrandrea, A. Mastroberardino, T. Masubuchi, P. Matricon, H. Matsunaga, T. Matsushita, P. Mättig, S. Mättig, C. Mattravers, J. Maurer, S. J. Maxfield, D. A. Maximov, A. Mayne, R. Mazini, M. Mazur, L. Mazzaferro, M. Mazzanti, J. Mc Donald, S. P. Mc Kee, A. McCarn, R. L. McCarthy, T. G. McCarthy, N. A. McCubbin, K. W. McFarlane, J. A. Mcfayden, G. Mchedlidze, T. Mclaughlan, S. J. McMahon, R. A. McPherson, A. Meade, J. Mechnich, M. Mechtel, M. Medinnis, S. Meehan, R. Meera-Lebbai, T. Meguro, S. Mehlhase, A. Mehta, K. Meier, B. Meirose, C. Melachrinos, B. R. Mellado Garcia, F. Meloni, L. Mendoza Navas, Z. Meng, A. Mengarelli, S. Menke, E. Meoni, K. M. Mercurio, P. Mermod, L. Merola, C. Meroni, F. S. Merritt, H. Merritt, A. Messina, J. Metcalfe, A. S. Mete, C. Meyer, C. Meyer, J-P. Meyer, J. Meyer, J. Meyer, S. Michal, R. P. Middleton, S. Migas, L. Mijović, G. Mikenberg, M. Mikestikova, M. Mikuž, D. W. Miller, R. J. Miller, W. J. Mills, C. Mills, A. Milov, D. A. Milstead, D. Milstein, A. A. Minaenko, M. Miñano Moya, I. A. Minashvili, A. I. Mincer, B. Mindur, M. Mineev, Y. Ming, L. M. Mir, G. Mirabelli, J. Mitrevski, V. A. Mitsou, S. Mitsui, P. S. Miyagawa, J. U. Mjörnmark, T. Moa, V. Moeller, S. Mohapatra, W. Mohr, R. Moles-Valls, A. Molfetas, K. Mönig, J. Monk, E. Monnier, J. Montejo Berlingen, F. Monticelli, S. Monzani, R. W. Moore, G. F. Moorhead, C. Mora Herrera, A. Moraes, N. Morange, J. Morel, G. Morello, D. Moreno, M. Moreno Llácer, P. Morettini, M. Morgenstern, M. Morii, A. K. Morley, G. Mornacchi, J. D. Morris, L. Morvaj, N. Möser, H. G. Moser, M. Mosidze, J. Moss, R. Mount, E. Mountricha, S. V. Mouraviev, E. J. W. Moyse, F. Mueller, J. Mueller, K. Mueller, T. Mueller, D. Muenstermann, T. A. Müller, Y. Munwes, W. J. Murray, I. Mussche, E. Musto, A. G. Myagkov, M. Myska, O. Nackenhorst, J. Nadal, K. Nagai, R. Nagai, K. Nagano, A. Nagarkar, Y. Nagasaka, M. Nagel, A. M. Nairz, Y. Nakahama, K. Nakamura, T. Nakamura, I. Nakano, G. Nanava, A. Napier, R. Narayan, M. Nash, T. Nattermann, T. Naumann, G. Navarro, H. A. Neal, P. Yu. Nechaeva, T. J. Neep, A. Negri, G. Negri, M. Negrini, S. Nektarijevic, A. Nelson, T. K. Nelson, S. Nemecek, P. Nemethy, A. A. Nepomuceno, M. Nessi, M. S. Neubauer, M. Neumann, A. Neusiedl, R. M. Neves, P. Nevski, F. M. Newcomer, P. R. Newman, V. Nguyen Thi Hong, R. B. Nickerson, R. Nicolaidou, B. Nicquevert, F. Niedercorn, J. Nielsen, N. Nikiforou, A. Nikiforov, V. Nikolaenko, I. Nikolic-Audit, K. Nikolics, K. Nikolopoulos, H. Nilsen, P. Nilsson, Y. Ninomiya, A. Nisati, R. Nisius, T. Nobe, L. Nodulman, M. Nomachi, I. Nomidis, S. Norberg, M. Nordberg, P. R. Norton, J. Novakova, M. Nozaki, L. Nozka, I. M. Nugent, A.-E. Nuncio-Quiroz, G. Nunes Hanninger, T. Nunnemann, E. Nurse, B. J. O’Brien, D. C. O’Neil, V. O’Shea, L. B. Oakes, F. G. Oakham, H. Oberlack, J. Ocariz, A. Ochi, S. Oda, S. Odaka, J. Odier, H. Ogren, A. Oh, S. H. Oh, C. C. Ohm, T. Ohshima, W. Okamura, H. Okawa, Y. Okumura, T. Okuyama, A. Olariu, A. G. Olchevski, S. A. Olivares Pino, M. Oliveira, D. Oliveira Damazio, E. Oliver Garcia, D. Olivito, A. Olszewski, J. Olszowska, A. Onofre, P. U. E. Onyisi, C. J. Oram, M. J. Oreglia, Y. Oren, D. Orestano, N. Orlando, I. O. Orlov, C. Oropeza Barrera, R. S. Orr, B. Osculati, R. Ospanov, C. Osuna, G. Otero y Garzon, J. P. Ottersbach, M. Ouchrif, E. A. Ouellette, F. Ould-Saada, A. Ouraou, Q. Ouyang, A. Ovcharova, M. Owen, S. Owen, V. E. Ozcan, N. Ozturk, A. Pacheco Pages, C. Padilla Aranda, S. Pagan Griso, E. Paganis, C. Pahl, F. Paige, P. Pais, K. Pajchel, G. Palacino, C. P. Paleari, S. Palestini, D. Pallin, A. Palma, J. D. Palmer, Y. B. Pan, E. Panagiotopoulou, J. G. Panduro Vazquez, P. Pani, N. Panikashvili, S. Panitkin, D. Pantea, A. Papadelis, Th. D. Papadopoulou, A. Paramonov, D. Paredes Hernandez, W. Park, M. A. Parker, F. Parodi, J. A. Parsons, U. Parzefall, S. Pashapour, E. Pasqualucci, S. Passaggio, A. Passeri, F. Pastore, Fr. Pastore, G. Pásztor, S. Pataraia, N. D. Patel, J. R. Pater, S. Patricelli, T. Pauly, M. Pecsy, S. Pedraza Lopez, M. I. Pedraza Morales, S. V. Peleganchuk, D. Pelikan, H. Peng, B. Penning, A. Penson, J. Penwell, M. Perantoni, D. V. Perepelitsa, K. Perez, T. Perez Cavalcanti, E. Perez Codina, M. T. Pérez García-Estañ, V. Perez Reale, L. Perini, H. Pernegger, R. Perrino, P. Perrodo, V. D. Peshekhonov, K. Peters, B. A. Petersen, J. Petersen, T. C. Petersen, E. Petit, A. Petridis, C. Petridou, E. Petrolo, F. Petrucci, D. Petschull, M. Petteni, R. Pezoa, A. Phan, P. W. Phillips, G. Piacquadio, A. Picazio, E. Piccaro, M. Piccinini, S. M. Piec, R. Piegaia, D. T. Pignotti, J. E. Pilcher, A. D. Pilkington, J. Pina, M. Pinamonti, A. Pinder, J. L. Pinfold, B. Pinto, C. Pizio, M. Plamondon, M.-A. Pleier, E. Plotnikova, A. Poblaguev, S. Poddar, F. Podlyski, L. Poggioli, D. Pohl, M. Pohl, G. Polesello, A. Policicchio, A. Polini, J. Poll, V. Polychronakos, D. Pomeroy, K. Pommès, L. Pontecorvo, B. G. Pope, G. A. Popeneciu, D. S. Popovic, A. Poppleton, X. Portell Bueso, G. E. Pospelov, S. Pospisil, I. N. Potrap, C. J. Potter, C. T. Potter, G. Poulard, J. Poveda, V. Pozdnyakov, R. Prabhu, P. Pralavorio, A. Pranko, S. Prasad, R. Pravahan, S. Prell, K. Pretzl, D. Price, J. Price, L. E. Price, D. Prieur, M. Primavera, K. Prokofiev, F. Prokoshin, S. Protopopescu, J. Proudfoot, X. Prudent, M. Przybycien, H. Przysiezniak, S. Psoroulas, E. Ptacek, E. Pueschel, J. Purdham, M. Purohit, P. Puzo, Y. Pylypchenko, J. Qian, A. Quadt, D. R. Quarrie, W. B. Quayle, F. Quinonez, M. Raas, V. Radeka, V. Radescu, P. Radloff, F. Ragusa, G. Rahal, A. M. Rahimi, D. Rahm, S. Rajagopalan, M. Rammensee, M. Rammes, A. S. Randle-Conde, K. Randrianarivony, F. Rauscher, T. C. Rave, M. Raymond, A. L. Read, D. M. Rebuzzi, A. Redelbach, G. Redlinger, R. Reece, K. Reeves, A. Reinsch, I. Reisinger, C. Rembser, Z. L. Ren, A. Renaud, M. Rescigno, S. Resconi, B. Resende, P. Reznicek, R. Rezvani, R. Richter, E. Richter-Was, M. Ridel, M. Rijpstra, M. Rijssenbeek, A. Rimoldi, L. Rinaldi, R. R. Rios, I. Riu, G. Rivoltella, F. Rizatdinova, E. Rizvi, S. H. Robertson, A. Robichaud-Veronneau, D. Robinson, J. E. M. Robinson, A. Robson, J. G. Rocha de Lima, C. Roda, D. Roda Dos Santos, A. Roe, S. Roe, O. Røhne, S. Rolli, A. Romaniouk, M. Romano, G. Romeo, E. Romero Adam, N. Rompotis, L. Roos, E. Ros, S. Rosati, K. Rosbach, A. Rose, M. Rose, G. A. Rosenbaum, E. I. Rosenberg, P. L. Rosendahl, O. Rosenthal, V. Rossetti, E. Rossi, L. P. Rossi, M. Rotaru, I. Roth, J. Rothberg, D. Rousseau, C. R. Royon, A. Rozanov, Y. Rozen, X. Ruan, F. Rubbo, I. Rubinskiy, N. Ruckstuhl, V. I. Rud, C. Rudolph, G. Rudolph, F. Rühr, A. Ruiz-Martinez, L. Rumyantsev, Z. Rurikova, N. A. Rusakovich, A. Ruschke, J. P. Rutherfoord, P. Ruzicka, Y. F. Ryabov, M. Rybar, G. Rybkin, N. C. Ryder, A. F. Saavedra, I. Sadeh, H. F-W. Sadrozinski, R. Sadykov, F. Safai Tehrani, H. Sakamoto, G. Salamanna, A. Salamon, M. Saleem, D. Salek, D. Salihagic, A. Salnikov, J. Salt, B. M. Salvachua Ferrando, D. Salvatore, F. Salvatore, A. Salvucci, A. Salzburger, D. Sampsonidis, B. H. Samset, A. Sanchez, J. Sánchez, V. Sanchez Martinez, H. Sandaker, H. G. Sander, M. P. Sanders, M. Sandhoff, T. Sandoval, C. Sandoval, R. Sandstroem, D. P. C. Sankey, A. Sansoni, C. Santoni, R. Santonico, H. Santos, I. Santoyo Castillo, J. G. Saraiva, T. Sarangi, E. Sarkisyan-Grinbaum, B. Sarrazin, F. Sarri, G. Sartisohn, O. Sasaki, Y. Sasaki, N. Sasao, I. Satsounkevitch, G. Sauvage, E. Sauvan, J. B. Sauvan, P. Savard, V. Savinov, D. O. Savu, L. Sawyer, D. H. Saxon, J. Saxon, C. Sbarra, A. Sbrizzi, D. A. Scannicchio, M. Scarcella, J. Schaarschmidt, P. Schacht, D. Schaefer, A. Schaelicke, S. Schaepe, S. Schaetzel, U. Schäfer, A. C. Schaffer, D. Schaile, R. D. Schamberger, V. Scharf, V. A. Schegelsky, D. Scheirich, M. Schernau, M. I. Scherzer, C. Schiavi, J. Schieck, M. Schioppa, S. Schlenker, E. Schmidt, K. Schmieden, C. Schmitt, S. Schmitt, B. Schneider, U. Schnoor, L. Schoeffel, A. Schoening, A. L. S. Schorlemmer, M. Schott, D. Schouten, J. Schovancova, M. Schram, C. Schroeder, N. Schroer, M. J. Schultens, H.-C. Schultz-Coulon, H. Schulz, M. Schumacher, B. A. Schumm, Ph. Schune, A. Schwartzman, Ph. Schwegler, Ph. Schwemling, R. Schwienhorst, R. Schwierz, J. Schwindling, T. Schwindt, M. Schwoerer, F. G. Sciacca, G. Sciolla, W. G. Scott, J. Searcy, G. Sedov, E. Sedykh, S. C. Seidel, A. Seiden, F. Seifert, J. M. Seixas, G. Sekhniaidze, S. J. Sekula, K. E. Selbach, D. M. Seliverstov, G. Sellers, M. Seman, N. Semprini-Cesari, C. Serfon, L. Serin, L. Serkin, R. Seuster, H. Severini, A. Sfyrla, E. Shabalina, M. Shamim, A. G. Shamov, L. Y. Shan, J. T. Shank, Q. T. Shao, M. Shapiro, P. B. Shatalov, K. Shaw, D. Sherman, P. Sherwood, S. Shimizu, M. Shimojima, T. Shin, M. Shiyakova, A. Shmeleva, M. J. Shochet, D. Short, S. Shrestha, E. Shulga, M. A. Shupe, P. Sicho, A. Sidoti, F. Siegert, Dj. Sijacki, O. Silbert, J. Silva, Y. Silver, D. Silverstein, S. B. Silverstein, V. Simak, O. Simard, Lj. Simic, S. Simion, E. Simioni, B. Simmons, R. Simoniello, M. Simonyan, P. Sinervo, N. B. Sinev, V. Sipica, G. Siragusa, A. Sircar, A. N. Sisakyan, S. Yu. Sivoklokov, J. Sjölin, T. B. Sjursen, L. A. Skinnari, H. P. Skottowe, K. Yu. Skovpen, P. Skubic, M. Slater, T. Slavicek, K. Sliwa, V. Smakhtin, B. H. Smart, L. Smestad, S. Yu. Smirnov, Y. Smirnov, L. N. Smirnova, O. Smirnova, B. C. Smith, D. Smith, K. M. Smith, M. Smizanska, K. Smolek, A. A. Snesarev, J. Snow, S. Snyder, R. Sobie, J. Sodomka, A. Soffer, D. A. Soh, C. A. Solans, M. Solar, J. Solc, E. Yu. Soldatov, U. Soldevila, E. Solfaroli Camillocci, A. A. Solodkov, O. V. Solovyanov, V. Solovyev, N. Soni, A. Sood, V. Sopko, B. Sopko, M. Sosebee, R. Soualah, A. M. Soukharev, S. Spagnolo, F. Spanò, R. Spighi, G. Spigo, R. Spiwoks, M. Spousta, T. Spreitzer, B. Spurlock, R. D. St. Denis, J. Stahlman, R. Stamen, E. Stanecka, R. W. Stanek, C. Stanescu, M. Stanescu-Bellu, M. M. Stanitzki, S. Stapnes, E. A. Starchenko, J. Stark, P. Staroba, P. Starovoitov, R. Staszewski, A. Staude, P. Stavina, G. Steele, P. Steinbach, P. Steinberg, I. Stekl, B. Stelzer, H. J. Stelzer, O. Stelzer-Chilton, H. Stenzel, S. Stern, G. A. Stewart, J. A. Stillings, M. C. Stockton, K. Stoerig, G. Stoicea, S. Stonjek, P. Strachota, A. R. Stradling, A. Straessner, J. Strandberg, S. Strandberg, A. Strandlie, M. Strang, E. Strauss, M. Strauss, P. Strizenec, R. Ströhmer, D. M. Strom, J. A. Strong, R. Stroynowski, B. Stugu, I. Stumer, J. Stupak, P. Sturm, N. A. Styles, D. Su, HS. Subramania, R. Subramaniam, A. Succurro, Y. Sugaya, C. Suhr, M. Suk, V. V. Sulin, S. Sultansoy, T. Sumida, X. Sun, J. E. Sundermann, K. Suruliz, G. Susinno, M. R. Sutton, Y. Suzuki, Y. Suzuki, M. Svatos, S. Swedish, I. Sykora, T. Sykora, D. Ta, K. Tackmann, A. Taffard, R. Tafirout, N. Taiblum, Y. Takahashi, H. Takai, R. Takashima, H. Takeda, T. Takeshita, Y. Takubo, M. Talby, A. A. Talyshev, M. C. Tamsett, K. G. Tan, J. Tanaka, R. Tanaka, S. Tanaka, S. Tanaka, A. J. Tanasijczuk, K. Tani, N. Tannoury, S. Tapprogge, D. Tardif, S. Tarem, F. Tarrade, G. F. Tartarelli, P. Tas, M. Tasevsky, E. Tassi, Y. Tayalati, C. Taylor, F. E. Taylor, G. N. Taylor, W. Taylor, M. Teinturier, F. A. Teischinger, M. Teixeira Dias Castanheira, P. Teixeira-Dias, K. K. Temming, H. Ten Kate, P. K. Teng, S. Terada, K. Terashi, J. Terron, M. Testa, R. J. Teuscher, J. Therhaag, T. Theveneaux-Pelzer, S. Thoma, J. P. Thomas, E. N. Thompson, P. D. Thompson, P. D. Thompson, A. S. Thompson, L. A. Thomsen, E. Thomson, M. Thomson, W. M. Thong, R. P. Thun, F. Tian, M. J. Tibbetts, T. Tic, V. O. Tikhomirov, Yu. A. Tikhonov, S. Timoshenko, E. Tiouchichine, P. Tipton, S. Tisserant, T. Todorov, S. Todorova-Nova, B. Toggerson, J. Tojo, S. Tokár, K. Tokushuku, K. Tollefson, M. Tomoto, L. Tompkins, K. Toms, A. Tonoyan, C. Topfel, N. D. Topilin, E. Torrence, H. Torres, E. Torró Pastor, J. Toth, F. Touchard, D. R. Tovey, T. Trefzger, L. Tremblet, A. Tricoli, I. M. Trigger, S. Trincaz-Duvoid, M. F. Tripiana, N. Triplett, W. Trischuk, B. Trocmé, C. Troncon, M. Trottier-McDonald, P. True, M. Trzebinski, A. Trzupek, C. Tsarouchas, J. C-L. Tseng, M. Tsiakiris, P. V. Tsiareshka, D. Tsionou, G. Tsipolitis, S. Tsiskaridze, V. Tsiskaridze, E. G. Tskhadadze, I. I. Tsukerman, V. Tsulaia, J.-W. Tsung, S. Tsuno, D. Tsybychev, A. Tua, A. Tudorache, V. Tudorache, J. M. Tuggle, M. Turala, D. Turecek, I. Turk Cakir, E. Turlay, R. Turra, P. M. Tuts, A. Tykhonov, M. Tylmad, M. Tyndel, K. Uchida, I. Ueda, R. Ueno, M. Ugland, M. Uhlenbrock, M. Uhrmacher, F. Ukegawa, G. Unal, A. Undrus, G. Unel, Y. Unno, D. Urbaniec, P. Urquijo, G. Usai, M. Uslenghi, L. Vacavant, V. Vacek, B. Vachon, S. Vahsen, J. Valenta, S. Valentinetti, A. Valero, S. Valkar, E. Valladolid Gallego, S. Vallecorsa, J. A. Valls Ferrer, R. Van Berg, P. C. Van Der Deijl, R. van der Geer, H. van der Graaf, R. Van Der Leeuw, E. van der Poel, D. van der Ster, N. van Eldik, P. van Gemmeren, I. van Vulpen, M. Vanadia, W. Vandelli, A. Vaniachine, P. Vankov, F. Vannucci, G. Vardanyan, R. Vari, E. W. Varnes, T. Varol, D. Varouchas, A. Vartapetian, K. E. Varvell, V. I. Vassilakopoulos, F. Vazeille, T. Vazquez Schroeder, G. Vegni, J. J. Veillet, F. Veloso, R. Veness, S. Veneziano, A. Ventura, D. Ventura, M. Venturi, N. Venturi, V. Vercesi, M. Verducci, W. Verkerke, J. C. Vermeulen, A. Vest, M. C. Vetterli, I. Vichou, T. Vickey, O. E. Vickey Boeriu, G. H. A. Viehhauser, S. Viel, M. Villa, M. Villaplana Perez, E. Vilucchi, M. G. Vincter, E. Vinek, V. B. Vinogradov, M. Virchaux, J. Virzi, O. Vitells, M. Viti, I. Vivarelli, F. Vives Vaque, S. Vlachos, D. Vladoiu, M. Vlasak, A. Vogel, P. Vokac, G. Volpi, M. Volpi, G. Volpini, H. von der Schmitt, H. von Radziewski, E. von Toerne, V. Vorobel, V. Vorwerk, M. Vos, R. Voss, J. H. Vossebeld, N. Vranjes, M. Vranjes Milosavljevic, V. Vrba, M. Vreeswijk, T. Vu Anh, R. Vuillermet, I. Vukotic, W. Wagner, P. Wagner, S. Wahrmund, J. Wakabayashi, S. Walch, J. Walder, R. Walker, W. Walkowiak, R. Wall, P. Waller, B. Walsh, C. Wang, H. Wang, H. Wang, J. Wang, J. Wang, R. Wang, S. M. Wang, T. Wang, A. Warburton, C. P. Ward, D. R. Wardrope, M. Warsinsky, A. Washbrook, C. Wasicki, I. Watanabe, P. M. Watkins, A. T. Watson, I. J. Watson, M. F. Watson, G. Watts, S. Watts, A. T. Waugh, B. M. Waugh, M. S. Weber, J. S. Webster, A. R. Weidberg, P. Weigell, J. Weingarten, C. Weiser, P. S. Wells, T. Wenaus, D. Wendland, Z. Weng, T. Wengler, S. Wenig, N. Wermes, M. Werner, P. Werner, M. Werth, M. Wessels, J. Wetter, C. Weydert, K. Whalen, A. White, M. J. White, S. White, S. R. Whitehead, D. Whiteson, D. Whittington, F. Wicek, D. Wicke, F. J. Wickens, W. Wiedenmann, M. Wielers, P. Wienemann, C. Wiglesworth, L. A. M. Wiik-Fuchs, P. A. Wijeratne, A. Wildauer, M. A. Wildt, I. Wilhelm, H. G. Wilkens, J. Z. Will, E. Williams, H. H. Williams, W. Willis, S. Willocq, J. A. Wilson, M. G. Wilson, A. Wilson, I. Wingerter-Seez, S. Winkelmann, F. Winklmeier, M. Wittgen, S. J. Wollstadt, M. W. Wolter, H. Wolters, W. C. Wong, G. Wooden, B. K. Wosiek, J. Wotschack, M. J. Woudstra, K. W. Wozniak, K. Wraight, M. Wright, B. Wrona, S. L. Wu, X. Wu, Y. Wu, E. Wulf, B. M. Wynne, S. Xella, M. Xiao, S. Xie, C. Xu, D. Xu, L. Xu, B. Yabsley, S. Yacoob, M. Yamada, H. Yamaguchi, A. Yamamoto, K. Yamamoto, S. Yamamoto, T. Yamamura, T. Yamanaka, T. Yamazaki, Y. Yamazaki, Z. Yan, H. Yang, U. K. Yang, Y. Yang, Z. Yang, S. Yanush, L. Yao, Y. Yao, Y. Yasu, G. V. Ybeles Smit, J. Ye, S. Ye, M. Yilmaz, R. Yoosoofmiya, K. Yorita, R. Yoshida, K. Yoshihara, C. Young, C. J. S. Young, S. Youssef, D. Yu, D. R. Yu, J. Yu, J. Yu, L. Yuan, A. Yurkewicz, B. Zabinski, R. Zaidan, A. M. Zaitsev, Z. Zajacova, L. Zanello, D. Zanzi, A. Zaytsev, C. Zeitnitz, M. Zeman, A. Zemla, C. Zendler, O. Zenin, T. Ženiš, D. Zerwas, G. Zevi della Porta, D. Zhang, H. Zhang, J. Zhang, X. Zhang, Z. Zhang, L. Zhao, Z. Zhao, A. Zhemchugov, J. Zhong, B. Zhou, N. Zhou, Y. Zhou, C. G. Zhu, H. Zhu, J. Zhu, Y. Zhu, X. Zhuang, V. Zhuravlov, A. Zibell, D. Zieminska, N. I. Zimin, R. Zimmermann, S. Zimmermann, S. Zimmermann, Z. Zinonos, M. Ziolkowski, R. Zitoun, L. Živković, V. V. Zmouchko, G. Zobernig, A. Zoccoli, M. zur Nedden, V. Zutshi, L. Zwalinski

**Affiliations:** 1 CERN, 1211 Geneva 23, Switzerland; 2 School of Chemistry and Physics, University of Adelaide, Adelaide, Australia; 3 Physics Department, SUNY Albany, Albany, NY United States of America; 4 Department of Physics, University of Alberta, Edmonton, AB Canada; 5 Department of Physics, Ankara University, Ankara, Turkey; 6 Department of Physics, Dumlupinar University, Kutahya, Turkey; 7 Department of Physics, Gazi University, Ankara, Turkey; 8 Division of Physics, TOBB University of Economics and Technology, Ankara, Turkey; 9 Turkish Atomic Energy Authority, Ankara, Turkey; 10 LAPP, CNRS/IN2P3 and Université de Savoie, Annecy-le-Vieux, France; 11 High Energy Physics Division, Argonne National Laboratory, Argonne, IL United States of America; 12 Department of Physics, University of Arizona, Tucson, AZ United States of America; 13 Department of Physics, The University of Texas at Arlington, Arlington, TX United States of America; 14 Physics Department, University of Athens, Athens, Greece; 15 Physics Department, National Technical University of Athens, Zografou, Greece; 16 Institute of Physics, Azerbaijan Academy of Sciences, Baku, Azerbaijan; 17 Institut de Física d’Altes Energies and Departament de Física de la Universitat Autònoma de Barcelona, Barcelona, Spain; 18 Institute of Physics, University of Belgrade, Belgrade, Serbia; 19 Vinca Institute of Nuclear Sciences, University of Belgrade, Belgrade, Serbia; 20 Department for Physics and Technology, University of Bergen, Bergen, Norway; 21 Physics Division, Lawrence Berkeley National Laboratory and University of California, Berkeley, CA United States of America; 22 Department of Physics, Humboldt University, Berlin, Germany; 23 Albert Einstein Center for Fundamental Physics and Laboratory for High Energy Physics, University of Bern, Bern, Switzerland; 24 School of Physics and Astronomy, University of Birmingham, Birmingham, United Kingdom; 25 Department of Physics, Bogazici University, Istanbul, Turkey; 26 Department of Physics, Dogus University, Istanbul, Turkey; 27 Department of Physics Engineering, Gaziantep University, Gaziantep, Turkey; 28 Department of Physics, Istanbul Technical University, Istanbul, Turkey; 29 INFN Sezione di Bologna, Bologna, Italy; 30 Dipartimento di Fisica e Astronomia, Università di Bologna, Bologna, Italy; 31 Physikalisches Institut, University of Bonn, Bonn, Germany; 32 Department of Physics, Boston University, Boston, MA United States of America; 33 Department of Physics, Brandeis University, Waltham, MA United States of America; 34 Universidade Federal do Rio De Janeiro COPPE/EE/IF, Rio de Janeiro, Brazil; 35 Federal University of Juiz de Fora (UFJF), Juiz de Fora, Brazil; 36 Federal University of Sao Joao del Rei (UFSJ), Sao Joao del Rei, Brazil; 37 Instituto de Fisica, Universidade de Sao Paulo, Sao Paulo, Brazil; 38 Physics Department, Brookhaven National Laboratory, Upton, NY United States of America; 39 National Institute of Physics and Nuclear Engineering, Bucharest, Romania; 40 University Politehnica Bucharest, Bucharest, Romania; 41 West University in Timisoara, Timisoara, Romania; 42 Departamento de Física, Universidad de Buenos Aires, Buenos Aires, Argentina; 43 Cavendish Laboratory, University of Cambridge, Cambridge, United Kingdom; 44 Department of Physics, Carleton University, Ottawa, ON Canada; 45 CERN, Geneva, Switzerland; 46 Enrico Fermi Institute, University of Chicago, Chicago, IL United States of America; 47 Departamento de Física, Pontificia Universidad Católica de Chile, Santiago, Chile; 48 Departamento de Física, Universidad Técnica Federico Santa María, Valparaíso, Chile; 49 Institute of High Energy Physics, Chinese Academy of Sciences, Beijing, China; 50 Department of Modern Physics, University of Science and Technology of China, Anhui, China; 51 Department of Physics, Nanjing University, Jiangsu, China; 52 School of Physics, Shandong University, Shandong, China; 53 Laboratoire de Physique Corpusculaire, Clermont Université and Université Blaise Pascal and CNRS/IN2P3, Clermont-Ferrand, France; 54 Nevis Laboratory, Columbia University, Irvington, NY United States of America; 55 Niels Bohr Institute, University of Copenhagen, Kobenhavn, Denmark; 56 INFN Gruppo Collegato di Cosenza, Rende, Italy; 57 Dipartimento di Fisica, Università della Calabria, Rende, Italy; 58 AGH University of Science and Technology, Faculty of Physics and Applied Computer Science, Krakow, Poland; 59 The Henryk Niewodniczanski Institute of Nuclear Physics, Polish Academy of Sciences, Krakow, Poland; 60 Physics Department, Southern Methodist University, Dallas, TX United States of America; 61 Physics Department, University of Texas at Dallas, Richardson, TX United States of America; 62 DESY, Hamburg and Zeuthen, Germany; 63 Institut für Experimentelle Physik IV, Technische Universität Dortmund, Dortmund, Germany; 64 Institut für Kern- und Teilchenphysik, Technische Universität Dresden, Dresden, Germany; 65 Department of Physics, Duke University, Durham, NC United States of America; 66 SUPA - School of Physics and Astronomy, University of Edinburgh, Edinburgh, United Kingdom; 67 INFN Laboratori Nazionali di Frascati, Frascati, Italy; 68 Fakultät für Mathematik und Physik, Albert-Ludwigs-Universität, Freiburg, Germany; 69 Section de Physique, Université de Genève, Geneva, Switzerland; 70 INFN Sezione di Genova, Genova, Italy; 71 Dipartimento di Fisica, Università di Genova, Genova, Italy; 72 E. Andronikashvili Institute of Physics, Iv. Javakhishvili Tbilisi State University, Tbilisi, Georgia; 73 High Energy Physics Institute, Tbilisi State University, Tbilisi, Georgia; 74 II Physikalisches Institut, Justus-Liebig-Universität Giessen, Giessen, Germany; 75 SUPA - School of Physics and Astronomy, University of Glasgow, Glasgow, United Kingdom; 76 II Physikalisches Institut, Georg-August-Universität, Göttingen, Germany; 77 Laboratoire de Physique Subatomique et de Cosmologie, Université Joseph Fourier and CNRS/IN2P3 and Institut National Polytechnique de Grenoble, Grenoble, France; 78 Department of Physics, Hampton University, Hampton, VA United States of America; 79 Laboratory for Particle Physics and Cosmology, Harvard University, Cambridge, MA United States of America; 80 Kirchhoff-Institut für Physik, Ruprecht-Karls-Universität Heidelberg, Heidelberg, Germany; 81 Physikalisches Institut, Ruprecht-Karls-Universität Heidelberg, Heidelberg, Germany; 82 ZITI Institut für technische Informatik, Ruprecht-Karls-Universität Heidelberg, Mannheim, Germany; 83 Faculty of Applied Information Science, Hiroshima Institute of Technology, Hiroshima, Japan; 84 Department of Physics, Indiana University, Bloomington, IN United States of America; 85 Institut für Astro- und Teilchenphysik, Leopold-Franzens-Universität, Innsbruck, Austria; 86 University of Iowa, Iowa City, IA United States of America; 87 Department of Physics and Astronomy, Iowa State University, Ames, IA United States of America; 88 Joint Institute for Nuclear Research, JINR Dubna, Dubna, Russia; 89 KEK, High Energy Accelerator Research Organization, Tsukuba, Japan; 90 Graduate School of Science, Kobe University, Kobe, Japan; 91 Faculty of Science, Kyoto University, Kyoto, Japan; 92 Kyoto University of Education, Kyoto, Japan; 93 Department of Physics, Kyushu University, Fukuoka, Japan; 94 Instituto de Física La Plata, Universidad Nacional de La Plata and CONICET, La Plata, Argentina; 95 Physics Department, Lancaster University, Lancaster, United Kingdom; 96 INFN Sezione di Lecce, Lecce, Italy; 97 Dipartimento di Matematica e Fisica, Università del Salento, Lecce, Italy; 98 Oliver Lodge Laboratory, University of Liverpool, Liverpool, United Kingdom; 99 Department of Physics, Jožef Stefan Institute and University of Ljubljana, Ljubljana, Slovenia; 100 School of Physics and Astronomy, Queen Mary University of London, London, United Kingdom; 101 Department of Physics, Royal Holloway University of London, Surrey, United Kingdom; 102 Department of Physics and Astronomy, University College London, London, United Kingdom; 103 Louisiana Tech University, Ruston, LA United States of America; 104 Laboratoire de Physique Nucléaire et de Hautes Energies, UPMC and Université Paris-Diderot and CNRS/IN2P3, Paris, France; 105 Fysiska institutionen, Lunds universitet, Lund, Sweden; 106 Departamento de Fisica Teorica C-15, Universidad Autonoma de Madrid, Madrid, Spain; 107 Institut für Physik, Universität Mainz, Mainz, Germany; 108 School of Physics and Astronomy, University of Manchester, Manchester, United Kingdom; 109 CPPM, Aix-Marseille Université and CNRS/IN2P3, Marseille, France; 110 Department of Physics, University of Massachusetts, Amherst, MA United States of America; 111 Department of Physics, McGill University, Montreal, QC Canada; 112 School of Physics, University of Melbourne, Victoria, Australia; 113 Department of Physics, The University of Michigan, Ann Arbor, MI United States of America; 114 Department of Physics and Astronomy, Michigan State University, East Lansing, MI United States of America; 115 INFN Sezione di Milano, Milano, Italy; 116 Dipartimento di Fisica, Università di Milano, Milano, Italy; 117 B.I. Stepanov Institute of Physics, National Academy of Sciences of Belarus, Minsk, Republic of Belarus; 118 National Scientific and Educational Centre for Particle and High Energy Physics, Minsk, Republic of Belarus; 119 Department of Physics, Massachusetts Institute of Technology, Cambridge, MA United States of America; 120 Group of Particle Physics, University of Montreal, Montreal, QC Canada; 121 P.N. Lebedev Institute of Physics, Academy of Sciences, Moscow, Russia; 122 Institute for Theoretical and Experimental Physics (ITEP), Moscow, Russia; 123 Moscow Engineering and Physics Institute (MEPhI), Moscow, Russia; 124 D.V. Skobeltsyn Institute of Nuclear Physics, M.V. Lomonosov Moscow State University, Moscow, Russia; 125 Fakultät für Physik, Ludwig-Maximilians-Universität München, München, Germany; 126 Max-Planck-Institut für Physik (Werner-Heisenberg-Institut), München, Germany; 127 Nagasaki Institute of Applied Science, Nagasaki, Japan; 128 Graduate School of Science and Kobayashi-Maskawa Institute, Nagoya University, Nagoya, Japan; 129 INFN Sezione di Napoli, Napoli, Italy; 130 Dipartimento di Scienze Fisiche, Università di Napoli, Napoli, Italy; 131 Department of Physics and Astronomy, University of New Mexico, Albuquerque, NM United States of America; 132 Institute for Mathematics, Astrophysics and Particle Physics, Radboud University Nijmegen/Nikhef, Nijmegen, Netherlands; 133 Nikhef National Institute for Subatomic Physics and University of Amsterdam, Amsterdam, Netherlands; 134 Department of Physics, Northern Illinois University, DeKalb, IL United States of America; 135 Budker Institute of Nuclear Physics, SB RAS, Novosibirsk, Russia; 136 Department of Physics, New York University, New York, NY United States of America; 137 Ohio State University, Columbus, OH United States of America; 138 Faculty of Science, Okayama University, Okayama, Japan; 139 Homer L. Dodge Department of Physics and Astronomy, University of Oklahoma, Norman, OK United States of America; 140 Department of Physics, Oklahoma State University, Stillwater, OK United States of America; 141 RCPTM, Palacký University, Olomouc, Czech Republic; 142 Center for High Energy Physics, University of Oregon, Eugene, OR United States of America; 143 LAL, Université Paris-Sud and CNRS/IN2P3, Orsay, France; 144 Graduate School of Science, Osaka University, Osaka, Japan; 145 Department of Physics, University of Oslo, Oslo, Norway; 146 Department of Physics, Oxford University, Oxford, United Kingdom; 147 INFN Sezione di Pavia, Pavia, Italy; 148 Dipartimento di Fisica, Università di Pavia, Pavia, Italy; 149 Department of Physics, University of Pennsylvania, Philadelphia, PA United States of America; 150 Petersburg Nuclear Physics Institute, Gatchina, Russia; 151 INFN Sezione di Pisa, Pisa, Italy; 152 Dipartimento di Fisica E. Fermi, Università di Pisa, Pisa, Italy; 153 Department of Physics and Astronomy, University of Pittsburgh, Pittsburgh, PA United States of America; 154 Laboratorio de Instrumentacao e Fisica Experimental de Particulas - LIP, Lisboa, Portugal; 155 Departamento de Fisica Teorica y del Cosmos and CAFPE, Universidad de Granada, Granada, Spain; 156 Institute of Physics, Academy of Sciences of the Czech Republic, Praha, Czech Republic; 157 Czech Technical University in Prague, Praha, Czech Republic; 158 Faculty of Mathematics and Physics, Charles University in Prague, Praha, Czech Republic; 159 State Research Center Institute for High Energy Physics, Protvino, Russia; 160 Particle Physics Department, Rutherford Appleton Laboratory, Didcot, United Kingdom; 161 Physics Department, University of Regina, Regina, SK Canada; 162 Ritsumeikan University, Kusatsu, Shiga Japan; 163 INFN Sezione di Roma I, Roma, Italy; 164 Dipartimento di Fisica, Università La Sapienza, Roma, Italy; 165 INFN Sezione di Roma Tor Vergata, Roma, Italy; 166 Dipartimento di Fisica, Università di Roma Tor Vergata, Roma, Italy; 167 INFN Sezione di Roma Tre, Roma, Italy; 168 Dipartimento di Matematica e Fisica, Università Roma Tre, Roma, Italy; 169 Faculté des Sciences Ain Chock, Réseau Universitaire de Physique des Hautes Energies - Université Hassan II, Casablanca, Morocco; 170 Centre National de l’Energie des Sciences Techniques Nucleaires, Rabat, Morocco; 171 Faculté des Sciences Semlalia, Université Cadi Ayyad, LPHEA-Marrakech, Morocco; 172 Faculté des Sciences, Université Mohamed Premier and LPTPM, Oujda, Morocco; 173 Faculté des sciences, Université Mohammed V-Agdal, Rabat, Morocco; 174 DSM/IRFU (Institut de Recherches sur les Lois Fondamentales de l’Univers), CEA Saclay (Commissariat à l’Energie Atomique et aux Energies Alternatives), Gif-sur-Yvette, France; 175 Santa Cruz Institute for Particle Physics, University of California Santa Cruz, Santa Cruz, CA United States of America; 176 Department of Physics, University of Washington, Seattle, WA United States of America; 177 Department of Physics and Astronomy, University of Sheffield, Sheffield, United Kingdom; 178 Department of Physics, Shinshu University, Nagano, Japan; 179 Fachbereich Physik, Universität Siegen, Siegen, Germany; 180 Department of Physics, Simon Fraser University, Burnaby, BC Canada; 181 SLAC National Accelerator Laboratory, Stanford, CA United States of America; 182 Faculty of Mathematics, Physics & Informatics, Comenius University, Bratislava, Slovak Republic; 183 Department of Subnuclear Physics, Institute of Experimental Physics of the Slovak Academy of Sciences, Kosice, Slovak Republic; 184 Department of Physics, University of Cape Town, Cape Town, South Africa; 185 Department of Physics, University of Johannesburg, Johannesburg, South Africa; 186 School of Physics, University of the Witwatersrand, Johannesburg, South Africa; 187 Department of Physics, Stockholm University, Stockholm, Sweden; 188 The Oskar Klein Centre, Stockholm, Sweden; 189 Physics Department, Royal Institute of Technology, Stockholm, Sweden; 190 Departments of Physics & Astronomy and Chemistry, Stony Brook University, Stony Brook, NY United States of America; 191 Department of Physics and Astronomy, University of Sussex, Brighton, United Kingdom; 192 School of Physics, University of Sydney, Sydney, Australia; 193 Institute of Physics, Academia Sinica, Taipei, Taiwan; 194 Department of Physics, Technion: Israel Institute of Technology, Haifa, Israel; 195 Raymond and Beverly Sackler School of Physics and Astronomy, Tel Aviv University, Tel Aviv, Israel; 196 Department of Physics, Aristotle University of Thessaloniki, Thessaloniki, Greece; 197 International Center for Elementary Particle Physics and Department of Physics, The University of Tokyo, Tokyo, Japan; 198 Graduate School of Science and Technology, Tokyo Metropolitan University, Tokyo, Japan; 199 Department of Physics, Tokyo Institute of Technology, Tokyo, Japan; 200 Department of Physics, University of Toronto, Toronto, ON Canada; 201 TRIUMF, Vancouver, BC Canada; 202 Department of Physics and Astronomy, York University, Toronto, ON Canada; 203 Faculty of Pure and Applied Sciences, University of Tsukuba, Tsukuba, Japan; 204 Department of Physics and Astronomy, Tufts University, Medford, MA United States of America; 205 Centro de Investigaciones, Universidad Antonio Narino, Bogota, Colombia; 206 Department of Physics and Astronomy, University of California Irvine, Irvine, CA United States of America; 207 INFN Gruppo Collegato di Udine, Udine, Italy; 208 ICTP, Trieste, Italy; 209 Dipartimento di Chimica, Fisica e Ambiente, Università di Udine, Udine, Italy; 210 Department of Physics, University of Illinois, Urbana, IL United States of America; 211 Department of Physics and Astronomy, University of Uppsala, Uppsala, Sweden; 212 Instituto de Física Corpuscular (IFIC) and Departamento de Física Atómica, Molecular y Nuclear and Departamento de Ingeniería Electrónica and Instituto de Microelectrónica de Barcelona (IMB-CNM), University of Valencia and CSIC, Valencia, Spain; 213 Department of Physics, University of British Columbia, Vancouver, BC Canada; 214 Department of Physics and Astronomy, University of Victoria, Victoria, BC Canada; 215 Department of Physics, University of Warwick, Coventry, United Kingdom; 216 Waseda University, Tokyo, Japan; 217 Department of Particle Physics, The Weizmann Institute of Science, Rehovot, Israel; 218 Department of Physics, University of Wisconsin, Madison, WI United States of America; 219 Fakultät für Physik und Astronomie, Julius-Maximilians-Universität, Würzburg, Germany; 220 Fachbereich C Physik, Bergische Universität Wuppertal, Wuppertal, Germany; 221 Department of Physics, Yale University, New Haven, CT United States of America; 222 Yerevan Physics Institute, Yerevan, Armenia; 223 Centre de Calcul de l’Institut National de Physique Nucléaire et de Physique des Particules (IN2P3), Villeurbanne, France

## Abstract

The inclusive jet cross-section has been measured in proton–proton collisions at $\sqrt{s} = 2.76~\mbox{TeV}$ in a dataset corresponding to an integrated luminosity of $0.20~\mbox {pb$^{-1}$}$ collected with the ATLAS detector at the Large Hadron Collider in 2011. Jets are identified using the anti-*k*_*t*_ algorithm with two radius parameters of 0.4 and 0.6. The inclusive jet double-differential cross-section is presented as a function of the jet transverse momentum *p*_T_ and jet rapidity *y*, covering a range of 20≤*p*_T_<430 GeV and |*y*|<4.4. The ratio of the cross-section to the inclusive jet cross-section measurement at $\sqrt{s} = 7~\mbox{TeV}$, published by the ATLAS Collaboration, is calculated as a function of both transverse momentum and the dimensionless quantity $x_{\mathrm{T}} = 2 p_{\mathrm{T}} / \sqrt{s}$, in bins of jet rapidity. The systematic uncertainties on the ratios are significantly reduced due to the cancellation of correlated uncertainties in the two measurements. Results are compared to the prediction from next-to-leading order perturbative QCD calculations corrected for non-perturbative effects, and next-to-leading order Monte Carlo simulation. Furthermore, the ATLAS jet cross-section measurements at $\sqrt{s} = 2.76~\mbox{TeV}$ and $\sqrt{s}=7~\mbox{TeV}$ are analysed within a framework of next-to-leading order perturbative QCD calculations to determine parton distribution functions of the proton, taking into account the correlations between the measurements.

## Introduction

Collimated jets of hadrons are a dominant feature of high-energy particle interactions. In Quantum Chromodynamics (QCD) they can be interpreted in terms of the fragmentation of quarks and gluons produced in a scattering process. The inclusive jet production cross-section provides information on the strong coupling and the structure of the proton, and tests the validity of perturbative QCD (pQCD) down to the shortest accessible distances.

The inclusive jet cross-section has been measured at high energy in proton–antiproton ($p\overline{p}$) collisions with $\sqrt{s} = 546~\mbox{GeV}$ and 630  GeV at the SPS [1–5], and with $\sqrt{s} = 546~\mbox{GeV}$, 630  GeV, 1.8 TeV and 1.96 TeV at the Tevatron [6–22].

The Large Hadron Collider (LHC) [23] at CERN allows the production of jets with transverse momenta in the TeV regime, colliding protons on protons (*pp*) with a centre-of-mass energy of currently up to $\sqrt{s} = 8~\mbox{TeV}$. The ATLAS Collaboration has presented early measurements of the inclusive jet cross-section at $\sqrt{s}=7~\mbox{TeV}$ based on a dataset with an integrated luminosity of 17 nb^−1^ for jets with a transverse momentum of 60≤*p*_T_<600 GeV and a rapidity^1^ of |*y*|<2.8 [24], as well as for the entire dataset of 37 pb^−1^ taken in 2010 for jets with 20≤*p*_T_<1500 GeV and |*y*|<4.4 [25]. The CMS Collaboration has presented results in the kinematic range of 18≤*p*_T_<1100 GeV and |*y*|<3 in a dataset of 34 pb^−1^ [26], in the range of 35≤*p*_T_<150 GeV and 3.2<|*y*|<4.7 using 3.1 pb^−1^ [27], and for 0.1≤*p*_T_<2 TeV and |*y*|<2.5 using 5.0 fb^−1^ [28]. These data are found to be generally well described by next-to-leading order (NLO) pQCD calculations, corrected for non-perturbative effects from hadronisation and the underlying event.

At the start of the 2011 data taking period of the LHC, the ATLAS experiment collected *pp* collision data at $\sqrt{s} = 2.76~\mbox{TeV}$ corresponding to an integrated luminosity of $0.20~\mbox {pb$^{-1}$}$. Having a centre-of-mass energy close to the highest energies reached in $p\overline{p}$ collisions, the dataset provides a connection from LHC measurements to previous measurements at the Tevatron. Moreover, measurements with the same detector at different centre-of-mass energies provide stringent tests of the theory, since the dominant systematic uncertainties are correlated. These correlations can be explored in a common fit to the measurements at different $\sqrt{s}$ or in ratios of the inclusive jet double-differential cross-sections. Hence, uncertainties can be significantly reduced. Such ratios were reported by previous experiments, UA2 [2], UA1 [4], CDF [7, 9] and D0 [12].

In this paper the inclusive jet double-differential cross-section is measured for 20≤*p*_T_<430 GeV and rapidities of |*y*|<4.4 at $\sqrt{s} = 2.76~\mbox{TeV}$. Moreover, the ratio to the previously measured cross-section at $\sqrt{s} = 7~\mbox{TeV}$ [25] is determined as a function of *p*_T_ and as a function of the dimensionless quantity $x_{\mathrm{T}} = 2 p_{\mathrm{T}} /\sqrt{s}$ [29]. For the ratio measured as a function of *p*_T_, many experimental systematic uncertainties cancel, while for the ratio measured as a function of *x*_T_, theoretical uncertainties are reduced. This allows a precise test of NLO pQCD calculations.

The outline of the paper is as follows. The definition of the jet cross-section is given in the next section, followed by a brief description of the ATLAS detector in Sect. 3 and the data taking in Sect. 4. The Monte Carlo simulation, the theoretical predictions and the uncertainties on the predictions are described in Sects. 5 and 6, followed by the event selection in Sect. 7 and the jet reconstruction and calibration in Sect. 8. The unfolding of detector effects and the treatment of systematic uncertainties are discussed in Sects. 9 and 10, followed by the results of the inclusive jet cross-section at $\sqrt{s} = 2.76~\mbox{TeV}$ in Sect. 11. The results of the ratio measurement, including the discussion of its uncertainties, are presented in Sect. 12. In Sect. 13 the results of an NLO pQCD fit to these data are discussed. The conclusion is given in Sect. 14.

## Definition of the measured variables

### Inclusive single-jet cross-section

Jets are identified using the anti-*k*_*t*_ algorithm [30] implemented in the FastJet [31, 32] software package. Two different values of the radius parameter, *R*=0.4 and *R*=0.6, are used. Inputs to the jet algorithm can be partons in the NLO pQCD calculation, stable particles after the hadronisation process in the Monte Carlo simulation, or energy deposits in the calorimeter in data.

Throughout this paper, the jet cross-section refers to the cross-section of jets built from stable particles, defined by having a proper mean lifetime of *cτ*>10 mm. Muons and neutrinos from decaying hadrons are included in this definition.

The inclusive jet double-differential cross-section, *d* ^2^ *σ*/*dp*_T_ *dy*, is measured as a function of the jet transverse momentum *p*_T_ in bins of rapidity *y*. The kinematic range of the measurement is 20≤*p*_T_<430 GeV and |*y*|<4.4.

The jet cross-section is also measured as a function of the dimensionless quantity *x*_T_. For a pure 2→2 central scattering of the partons, *x*_T_ gives the momentum fraction of the initial-state partons with respect to the parent proton.

### Ratio of jet cross-sections at different centre-of-mass energies

The inclusive jet double-differential cross-section can be related to the invariant cross-section according to

1 $$ E \frac{d^{3}\sigma}{dp^{3}} = \frac{1}{2\pi p_{\mathrm {T}} } \frac{d^{2}\sigma}{d p_{\mathrm{T}} \,dy}, $$

where *E* and *p* denote the energy and momentum of the jet, respectively. The dimensionless scale-invariant cross-section *F*(*y*,*x*_T_) can be defined as [33]:

2 $$ F(y, x_{\mathrm{T}} , \sqrt{s}) = p_{\mathrm {T}} ^4 E \frac{d^{3}\sigma}{dp^{3}} = \frac{ p_{\mathrm {T}} ^3}{2 \pi} \frac{d^{2}\sigma}{d p_{\mathrm{T}} \,dy} =\frac{s}{8 \pi} x_{\mathrm{T}} ^3 \frac{d^2\sigma }{d x_{\mathrm{T}} \,dy}. $$

In the simple quark–parton model [34, 35], *F* does not depend on the centre-of-mass energy, as follows from dimensional analysis. In QCD, however, several effects lead to a violation of the scaling behaviour, introducing a *p*_T_ (or $\sqrt{s}$) dependence to *F*. The main effects are the scale dependence of the parton distribution functions (PDFs) and the strong coupling constant *α*_S_.

The cross-section ratio of the invariant jet cross-section measured at $\sqrt{s}=2.76~\mbox{TeV}$ to the one measured at $\sqrt{s}=7~\mbox{TeV}$ is then denoted by:

3 $$ \rho(y, x_{\mathrm{T}} ) = \frac{F(y, x_{\mathrm{T}} , 2.76~\mbox{TeV})}{F(y, x_{\mathrm {T}} , 7~\mbox{TeV})}. $$

The violation of the $\sqrt{s}$ scaling leads to a deviation of *ρ*(*y*,*x*_T_) from one. *ρ*(*y*,*x*_T_) is calculated by measuring the bin-averaged inclusive jet double-differential cross-sections at the two centre-of-mass energies in the same *x*_T_ ranges:

4 $$ \rho(y, x_{\mathrm{T}} ) = \biggl(\frac{2.76~\mbox {TeV}}{7~\mbox{TeV}} \biggr)^3\cdot\frac{\sigma(y, x_{\mathrm{T}} , 2.76~\mbox{TeV})}{\sigma(y, x_{\mathrm {T}} , 7~\mbox{TeV})}, $$

where $\sigma(y, x_{\mathrm{T}} , \sqrt{s})$ corresponds to the measured averaged cross-section *d* ^2^ *σ*/*dp*_T_ *dy* in a bin $(y, p_{\mathrm{T}} =\sqrt {s}\cdot x_{\mathrm{T}} /2)$, and *x*_T_ is chosen to be at the bin centre. Here, the *p*_T_ binning for the inclusive jet cross-section at $\sqrt{s}=2.76~\mbox{TeV}$ is chosen such that it corresponds to the same *x*_T_ ranges obtained from the *p*_T_ bins of the jet cross-section measurement at $\sqrt{s}=7~\mbox{TeV}$. The bin boundaries are listed in Appendix A.

The ratio of inclusive double-differential cross-sections is also measured as a function of *p*_T_, where the same *p*_T_ binning is used for both centre-of-mass energies. This ratio is denoted by

5 $$ \begin{aligned} \rho(y, p_{\mathrm{T}} ) &= \frac{\sigma(y, p_{\mathrm{T}} , 2.76~\mbox{TeV})}{\sigma(y, p_{\mathrm {T}} , 7~\mbox{TeV})}, \end{aligned} $$

where $\sigma(y, p_{\mathrm{T}} , \sqrt{s})$ is the measured averaged cross-section *d* ^2^ *σ*/*dp*_T_ *dy* in a bin (*y*,*p*_T_) at a centre-of-mass energy of $\sqrt{s}$. Since the uncertainty due to the jet energy scale is the dominant experimental uncertainty at a given *p*_T_, the experimental systematic uncertainty is significantly reduced by taking the cross-section ratio in the same *p*_T_ bins.

## The ATLAS detector

The ATLAS detector consists of a tracking system (inner detector) in a 2 T axial magnetic field up to a pseudorapidity^2^ of |*η*|= 2.5, sampling electromagnetic and hadronic calorimeters up to |*η*|=4.9, and muon chambers in an azimuthal magnetic field provided by a system of toroidal magnets. A detailed description of the ATLAS detector can be found elsewhere [36].

The inner detector consists of layers of silicon pixel detectors, silicon microstrip detectors and transition radiation tracking detectors. It is used in this analysis to identify candidate collision events by constructing vertices from tracks. Jets are reconstructed using the energy deposits in the calorimeter, whose granularity and material varies as a function of *η*. The electromagnetic calorimeter uses lead as an absorber, liquid argon (LAr) as the active medium and has a fine granularity. It consists of a barrel (|*η*|<1.475) and an endcap (1.375<|*η*|<3.2) region. The hadronic calorimeter is divided into three distinct regions: a barrel region (|*η*|<0.8) and an extended barrel region (0.8<|*η*|<1.7) instrumented with a steel/scintillating-tile modules, and an endcap region (1.5<|*η*|<3.2) using copper/LAr modules. Finally, the forward calorimeter (3.1<|*η*|<4.9) is instrumented with copper/LAr and tungsten/LAr modules to provide electromagnetic and hadronic energy measurements, respectively.

The ATLAS trigger system is composed of three consecutive levels: level 1, level 2 and the event filter, with progressively increasing computing time per event, finer granularity and access to more detector systems. For jet triggering, the relevant systems are the minimum bias trigger scintillators (MBTS), located in front of the endcap cryostats covering 2.1<|*η*|<3.8, as well as calorimeter triggers for central jets, covering |*η*|<3.2, and for forward jets, covering 3.1<|*η*|<4.9, respectively.

## Data taking

The proton–proton collision data at $\sqrt{s} = 2.76~\mbox{TeV}$ were collected at the start of the 2011 data taking period of the LHC. The total integrated luminosity of the collected data is $0.20~\mbox {pb$^{-1}$}$. The proton bunches were grouped in nine bunch trains. The time interval between two consecutive bunches was 525 ns. The average number of interactions per bunch crossing is found to be *μ*=0.24. All events used in this analysis were collected with good operational status of the relevant detector components for jet measurements.

The data at $\sqrt{s} = 7~\mbox{TeV}$ have a total integrated luminosity of $37~\mbox {pb$^{-1}$}$. Further details are given in Ref. [25].

## Monte Carlo simulation

Events used in the simulation of the detector response are produced by the Pythia 6.423 generator [37], using the MRST 2007 LO* PDFs [38]. The generator utilises leading-order (LO) pQCD matrix elements for 2→2 processes, along with a leading-logarithmic *p*_T_-ordered parton shower [39], an underlying event simulation with multiple parton interactions [40], and the Lund string model for hadronisation [41]. The event generation uses the ATLAS Minimum Bias Tune 1 (AMBT1) set of parameters [42]. Additional proton–proton collisions occurring in the same bunch crossing have not been simulated because the average number of interactions per beam crossing is so small.

The Geant software toolkit [43] within the ATLAS simulation framework [44] simulates the propagation of the generated particles through the ATLAS detector and their interactions with the detector material.

The Herwig++ 2.5.1 [45, 46] generator is used in addition to Pythia in the evaluation of non-perturbative effects in the theory prediction. It is based on the 2→2 LO pQCD matrix elements and a leading-logarithmic angular-ordered parton shower [47]. The cluster model [48] is used for the hadronisation, and an underlying event simulation is based on the eikonal model [49].

## Theoretical predictions

### NLO pQCD prediction

The NLO pQCD predictions are calculated using the NLOJET++ 4.1.2 [50] program. For fast and flexible calculations with various PDFs and factorisation and renormalisation scales, the APPLgrid software [51] is interfaced with NLOJET++. The renormalisation scale, *μ*_*R*_, and the factorisation scale, *μ*_*F*_, are chosen for each event as $\mu_{R}=\mu_{F}= p_{\mathrm{T}} ^{\mathrm{max}}(y_{i})$, where $p_{\mathrm{T}} ^{\mathrm{max}}(y_{i})$ is the maximum jet transverse momentum found in a rapidity bin *y*_*i*_. If jets are present in different rapidity bins, several scales within the event are used.

The default calculation uses the CT10 [52] PDF set. Predictions using the PDF sets MSTW 2008 [53], NNPDF 2.1 (100) [54, 55], HERAPDF 1.5 [56] and ABM 11 NLO (*n*_*f*_=5) [57] are also made for comparison. The value for *α*_S_ is taken from the corresponding PDF set.

Three sources of uncertainty in the NLO pQCD calculation are considered, namely the uncertainty on the PDF sets, the choice of factorisation and renormalisation scales, and the uncertainty on the value of the strong coupling constant, *α*_S_. The PDF uncertainty is defined at 68 % confidence level (CL) and evaluated following the prescriptions given for each PDF set and the PDF4LHC recommendations [58]. The uncertainty on the scale choice is evaluated by varying the renormalisation scale and the factorisation scale by a factor of two with respect to the original choice in the calculation. The considered variations are

6 $$\begin{aligned} (f_{\mu_R}, f_{\mu_F})={}& (0.5,0.5),\ (0.5,1),\ (1,0.5), \\ & (1,2),\ (2,1),\ (2,2), \end{aligned}$$

where $f_{\mu_{R}}$ and $f_{\mu_{F}}$ are factors for the variation of renormalisation and factorisation scales, hence $\mu_{R} = f_{\mu _{R}}\cdot p_{\mathrm{T}} ^{\mathrm{max}}$ and $\mu_{F} = f_{\mu_{F}} \cdot p_{\mathrm{T}} ^{\mathrm{max}}$. The envelope of the resulting variations is taken as the scale uncertainty. The uncertainty reflecting the *α*_S_ measurement precision is evaluated following the recommendation of the CTEQ group [59], by calculating the cross-section using a series of PDFs which are derived with various fixed *α*_S_ values. Electroweak corrections are not included in the theory predictions. The effect is found to be *O*(10 %) at high *p*_T_, and negligible at small *p*_T_ for $\sqrt{s}=7~\mbox{TeV}$ [60].

The theoretical predictions for the cross-section ratios at the two different energies, *ρ*(*y*,*x*_T_) or *ρ*(*y*,*p*_T_), are also obtained from the NLO pQCD calculations. The evaluation of the prediction at $\sqrt{s}=7~\mbox{TeV}$ is given in Ref. [25], and the procedure is identical to the one used for $\sqrt{s}=2.76~\mbox{TeV}$ in the present analysis. Hence, the uncertainty on the ratio is determined using the same variation in each component of the considered uncertainties simultaneously for both $\sqrt{s}=2.76~\mbox{TeV}$ and $\sqrt{s}=7~\mbox{TeV}$ cross-section predictions.

### Non-perturbative corrections

The fixed-order NLO pQCD calculations, described in Sect. 6.1, predict the parton-level cross-section, which should be corrected for non-perturbative effects before comparison with the measurement at particle level. The corrections are derived using LO Monte Carlo event generators complemented by the leading-logarithmic parton shower by evaluating the bin-wise ratio of the cross-section with and without hadronisation and the underlying event. Each bin of the NLO pQCD cross-section is then multiplied by the corresponding correction for non-perturbative effects. The baseline correction factors are obtained from Pythia 6.425 [37] with the AUET2B CTEQ6L1 tune [61]. The uncertainty is estimated as the envelope of the correction factors obtained from a series of different generators and tunes: Pythia 6.425 using the tunes AUET2B LO** [61], AUET2 LO** [62], AMBT2B CTEQ6L1 [61], AMBT1 [42], Perugia 2010 [63] and Perugia 2011 [63]; Pythia 8.150 [64] with tune 4C [61]; and Herwig++ 2.5.1 [45] with tune UE7000-2 [61]. The AMBT2B CTEQ6L1 and AMBT1 tunes, which are based on observables sensitive to the modelling of minimum bias interactions, are included to provide a systematically different estimate of the underlying event activity.

The NLO pQCD prediction for the cross-section ratio also needs corrections for non-perturbative effects. The same procedure is used to evaluate non-perturbative corrections for the cross-section at $\sqrt{s}=7~\mbox{TeV}$ using the same series of generator tunes. A ratio of corrections at $\sqrt{s}=2.76~\mbox{TeV}$ and $\sqrt{s}=7~\mbox{TeV}$ is calculated for each generator tune. As for the cross-section, Pythia 6.425 with the AUET2B CTEQ6L1 tune is used as the central value of the correction factor for the cross-section ratio and the envelope of the correction factors from the other tunes is taken as an uncertainty.

### Predictions from NLO matrix elements with parton-shower Monte Carlo simulation

The measured jet cross-section is also compared to predictions from Powheg jet pair production, revision 2169 [65, 66]. Powheg is an NLO generator that uses the Powheg Box 1.0 package [67–69], which can be interfaced to different Monte Carlo programs to simulate the parton shower, the hadronisation and the underlying event. This simulation using a matched parton shower is expected to produce a more accurate theoretical prediction. However, ambiguities in the matching procedure, non-optimal tuning of parton shower-parameters, and the fact that it is a hybrid between an NLO matrix element calculation and the currently available LO parton-shower generators may introduce additional theoretical uncertainties.

In the Powheg algorithm, each event is built by first producing a QCD 2→2 partonic scattering. The renormalisation and factorisation scales for the fixed-order NLO prediction are set to be equal to the transverse momentum of the outgoing partons, $p_{\mathrm{T}} ^{\mathrm{Born}}$. In addition to the hard scatter, Powheg also generates the hardest partonic emission in the event. The event is evolved to the particle level using a parton-shower event generator, where the radiative emissions in the parton showers are limited by the matching scale *μ*_*M*_ provided by Powheg. The simulation of parton showers uses Pythia with the ATLAS underlying event tunes, AUET2B [61] and Perugia 2011 [63]. The tunes are derived from the standalone versions of these event generators, with no optimisation for the Powheg predictions. The CT10 PDF set is used in both Powheg and Pythia.

To avoid fluctuations in the final observables after the showering process, the Powheg event generation is performed using a new option^3^ that became available recently [66]. For *p*_T_<100 GeV, this new prediction differs by *O*(10 %) from the Powheg prediction at $\sqrt{s}=7~\mbox{TeV}$ from the previous analysis, which followed a different approach [25]. The uncertainty from the renormalisation and factorisation scales for the Powheg prediction is expected to be similar to that obtained with NLOJET++. The matching scale can potentially have a large impact on the cross-section prediction at particle level, affecting the parton shower, initial-state radiation and multiple interactions, but a procedure to estimate this uncertainty is currently not well defined. Therefore no uncertainties are shown for the Powheg curves.

### Prediction for the inclusive jet cross-section at $\sqrt {s}=2.76\mbox{ TeV}$

**Fig. 1:**
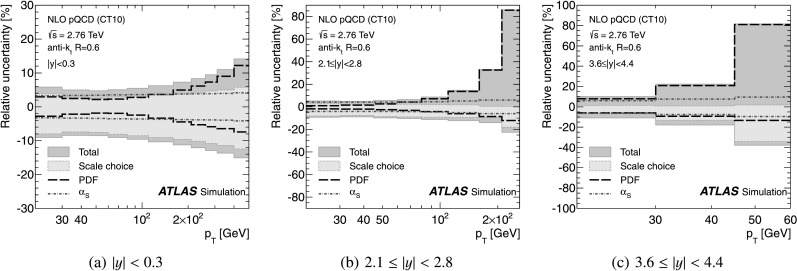
The uncertainty in the NLO pQCD prediction of the inclusive jet cross-section at $\sqrt{s}=2.76~\mbox{TeV}$, calculated using NLOJET++ with the CT10 PDF set, for anti-*k*_*t*_ jets with *R*=0.6 shown in three representative rapidity bins as a function of the jet *p*_T_. In addition to the total uncertainty, the uncertainties from the scale choice, the PDF set and the strong coupling constant, *α*_S_, are shown separately

The evaluated relative uncertainties of the NLO pQCD calculation for the inclusive jet cross-section at $\sqrt{s}=2.76~\mbox{TeV}$ are shown in Fig. 1 as a function of the jet *p*_T_ for representative rapidity bins and *R*=0.6. In the central rapidity region, the uncertainties are about 5 % for *p*_T_≲100 GeV, increasing to about 15 % in the highest jet *p*_T_ bin. In the most forward region, they are 10 % in the lowest *p*_T_ bin and up to 80 % in the highest *p*_T_ bin. In the higher *p*_T_ region, the upper bound on the uncertainty is driven by the PDF uncertainty, while the lower bound and the uncertainty at low *p*_T_ are dominated by the scale choice. The uncertainties for *R*=0.4 are similar.

**Fig. 2:**
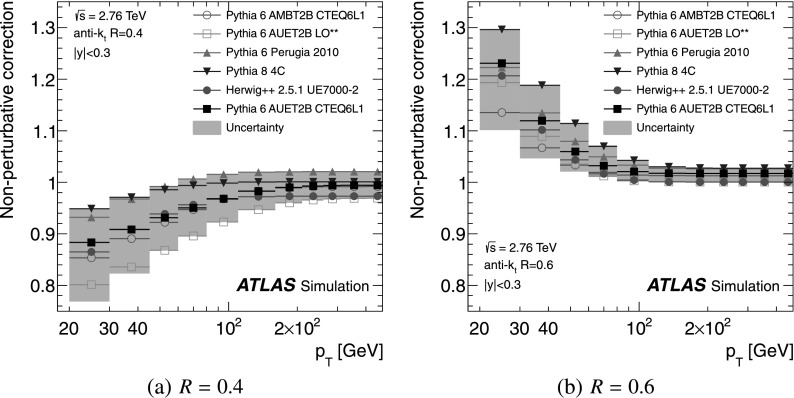
Non-perturbative correction factors for the inclusive jet cross-section for anti-*k*_*t*_ jets with (**a**) *R*=0.4 and (**b**) *R*=0.6 in the jet rapidity region |*y*|<0.3 as a function of the jet *p*_T_ for Monte Carlo simulations with various tunes. The correction factors derived from Pythia 6 with the AUET2B CTEQ6L1 tune (*full-square*) are used for the NLO pQCD prediction in this measurement, with the uncertainty indicated by the *shaded area*. For better visibility, some tunes used in the uncertainty determination are not shown

The correction factors for non-perturbative effects and their uncertainties are shown in Fig. 2 for the inclusive jet cross-section at $\sqrt{s}=2.76~\mbox{TeV}$ in the central rapidity bin. For jets with *R*=0.4, the correction is about −10 % in the lowest *p*_T_ bin, while for jets with *R*=0.6, it is about +20 % as a result of the interplay of the hadronisation and the underlying event for the different jet sizes. In the high-*p*_T_ region, the corrections are almost unity for both jet radius parameters, and the uncertainty is at the level of ±2 %.

### Prediction for the cross-section ratio

**Fig. 3:**
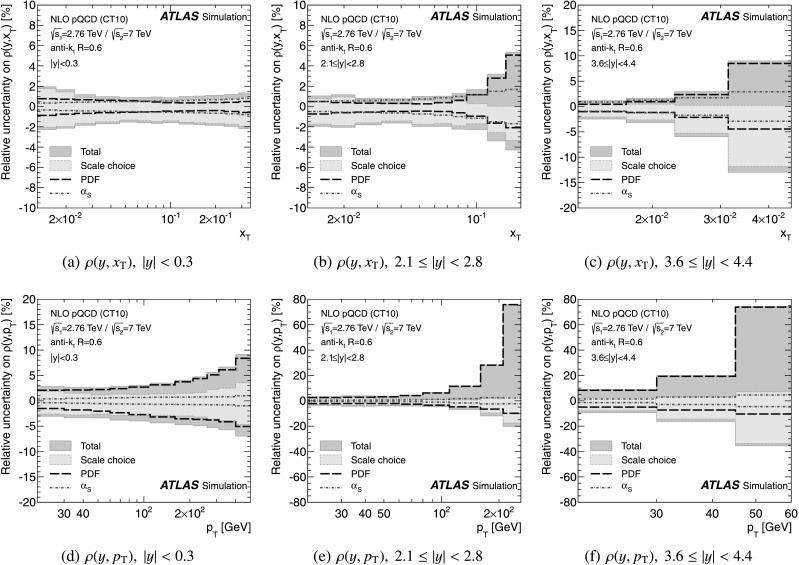
The uncertainty in the NLO pQCD prediction of the cross-section ratio *ρ*(*y*,*x*_T_) ((**a**)–(**c**)) and *ρ*(*y*,*p*_T_) ((**d**)–(**f**)), calculated using NLOJET++ with the CT10 PDF set, for anti-*k*_*t*_ jets with *R*=0.6 shown in three representative rapidity bins as a function of the jet *x*_T_ and of the jet *p*_T_, respectively. In addition to the total uncertainty, the uncertainties from the scale choice, the PDF set and the strong coupling constant, *α*_S_, are shown separately

Figures 3(a)–(c) show the uncertainty on the NLO pQCD calculation of *ρ*(*y*,*x*_T_) in representative rapidity bins for *R*=0.6. They are significantly reduced to a level of a few percent in the central rapidity region compared to the uncertainties on the cross-sections shown in Fig. 1. The dominant uncertainty at low *p*_T_ is the uncertainty on the renormalisation and factorisation scale choice, while at high *p*_T_ the uncertainty due to the PDF contributes again significantly. The NLO pQCD calculation of *ρ*(*y*,*p*_T_) has an uncertainty of less than ±5 % for *p*_T_ up to 200  GeV in the central rapidity region, as shown in Fig. 3(d). The uncertainty increases for higher *p*_T_ of the jet due mostly to the uncertainties on the PDFs, which are below 10 % for central jets. In the forward region, it reaches up to 80 % in the highest *p*_T_ bins, as shown in Figs. 3(e) and 3(f). The corresponding uncertainties for jets with *R*=0.4 are similar, except for a larger contribution due to the scale choice in the uncertainty on *ρ*(*y*,*p*_T_).

**Fig. 4:**
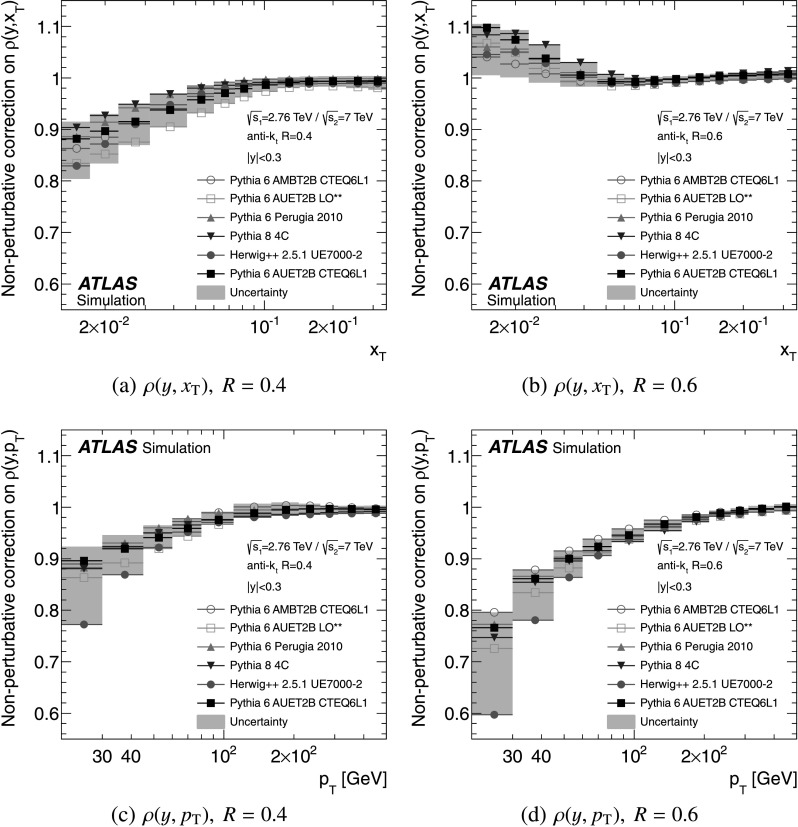
Non-perturbative correction factors for the cross-section ratios, *ρ*(*y*,*x*_T_) and *ρ*(*y*,*p*_T_), for anti-*k*_*t*_ jets with *R*=0.4 or *R*=0.6 shown for a jet rapidity of |*y*|<0.3 for Monte Carlo simulations with various tunes as a function of the jet *x*_T_ and of the jet *p*_T_, respectively. The correction factors derived from Pythia 6 with the AUET2B CTEQ6L1 tune (*full-square*) are used for the NLO pQCD prediction in this measurement, with the uncertainty indicated by the *shaded area*. For better visibility, some tunes used in the uncertainty determination are not shown

Non-perturbative corrections to *ρ*(*y*,*x*_T_) have a different *x*_T_ dependence for jets with *R*=0.4 and *R*=0.6, as shown in Figs. 4(a) and 4(b). The behaviour of *ρ*(*y*,*x*_T_) is driven by the corrections for the cross-section at $\sqrt{s}=2.76~\mbox{TeV}$ since $p_{\mathrm{T}} ^{7~\text{TeV}}=(7/2.76)\cdot p_{\mathrm{T}} ^{2.76~\text{TeV}}$ in the same *x*_T_ bins (see Appendix A) and since the non-perturbative correction is almost flat in the high-*p*_T_ region. For jets with *R*=0.4, the correction is −10 % in the lowest *x*_T_ bin. For *R*=0.6, the correction in this region is in the opposite direction, increasing the prediction by +10 %. The uncertainty in the lowest *x*_T_ bin for both radius parameters is ∼ ±10 %. The non-perturbative corrections to *ρ*(*y*,*p*_T_) are shown in Figs. 4(c) and 4(d), where a similar *p*_T_ dependence for *R*=0.4 and *R*=0.6 is found. They amount to −10 % for jets with *R*=0.4 and −25 % for jets with *R*=0.6 in the lowest *p*_T_ bins. This is due to the correction factors for the NLO pQCD prediction at $\sqrt{s}=7~\mbox{TeV}$ [25] being larger than those at $\sqrt{s}=2.76~\mbox{TeV}$. Corrections obtained from Pythia with various tunes generally agree within 5 % for central jets, while the non-perturbative corrections from Herwig++ deviate from the ones of the Pythia tunes by more than 10 % in the lowest *p*_T_ bin.

## Event selection

Events are selected online using various trigger definitions according to the *p*_T_ and the rapidity *y* of the jets [70]. In the lowest *p*_T_ region (*p*_T_<35 GeV for |*y*|<2.1, *p*_T_<30 GeV for 2.1≤|*y*|<2.8, *p*_T_<28 GeV for 2.8≤|*y*|<3.6, and *p*_T_<26 GeV for 3.6≤|*y*|<4.4), a trigger requiring at least two hits in the MBTS is used. For the higher *p*_T_ region, jet-based triggers are used, which select events that contain a jet with sufficient transverse energy at the electromagnetic scale.^4^ The efficiency of the jet-based triggers is determined using the MBTS, and the one for MBTS using the independent trigger from the Zero Degree Calorimeter [71]. Only triggers that are >99 % efficient for a given jet *p*_T_ value are used. In the region 2.8<|*y*|<3.6, both a central and a forward jet trigger are used in combination to reach an efficiency of >99 %. Events are required to have at least one well-reconstructed event vertex, which must have at least three associated tracks with a minimum *p*_T_ of 150 MeV.

## Jet reconstruction and calibration

The reconstruction procedure and the calibration factors for the jet cross-section measurement at $\sqrt{s}=2.76~\mbox{TeV}$ are nearly identical to those used for the measurement at $\sqrt{s}=7~\mbox {TeV}$ with 2010 data [25]; the few exceptions are explicitly specified below.

Jets are reconstructed with the anti-*k*_*t*_ algorithm using as input objects topological clusters [72, 73] of energy deposits in the calorimeter, calibrated at the electromagnetic scale. The four-momenta of the reconstructed jets are corrected event-by-event using the actual vertex position. A jet energy scale (JES) correction is then applied to correct for detector effects such as energy loss in dead material in front of the calorimeter or between calorimeter segments, and to compensate for the lower calorimeter response to hadrons than to electrons or photons [72, 73]. Due to the low number of interactions per bunch crossing, an offset correction accounting for additional energy depositions from multiple interactions in the same bunch crossing, so-called pile-up, is not applied in this measurement.

The estimation of the uncertainty in the jet energy measurement uses single-hadron calorimeter response measurements [74] and systematic Monte Carlo simulation variations. An uncertainty of about 2.5 % in the central calorimeter region over a wide momentum range of 60≤*p*_T_<800 GeV is obtained [73]. For jets with lower *p*_T_ and for forward jets the uncertainties are larger.

All reconstructed jets with *p*_T_>20 GeV, |*y*|<4.4 and a positive decision from the trigger that is used in the corresponding jet kinematic region are considered in this analysis. Jets are furthermore required to pass jet quality selections to reject fake jets reconstructed from non-collision signals, such as beam-related background, cosmic rays or detector noise. The applied selections were established with the $\sqrt{s}=7~\mbox{TeV}$ data in 2010 [25, 73] and are validated in the $\sqrt {s}=2.76~\mbox{TeV}$ data by studying distributions of the selection variables with techniques similar to those in Ref. [73]. The rate of fake jets after the jet selection is negligible.

The efficiency of the jet quality selection is measured using a tag-and-probe method [73]. The largest inefficiency is found to be below 4 % for jets with *p*_T_=20 GeV. Within the statistical uncertainty, the measured efficiency is in good agreement with the efficiency previously measured for $\sqrt{s}=7~\mbox{TeV}$ data in 2010. Because of the larger number of events in the 2010 data at $\sqrt {s}=7~\mbox{TeV}$, the jet selection efficiency from the 2010 data is taken.

Various types of validity and consistency checks have been performed on the data, such as testing the expected invariance of the jet cross-section as a function of *ϕ*, or the stability of the jet yield over time. No statistically significant variations are detected. The basic kinematic variables are described by the Monte Carlo simulation within the systematic uncertainties.

## Unfolding of detector effects

Corrections for the detector inefficiencies and resolutions are performed to extract the particle-level cross-section, based on a transfer matrix that relates the *p*_T_ of the jet at particle-level and the reconstruction-level.

For the unfolding, the Iterative, Dynamically Stabilised (IDS) Bayesian unfolding method [75] is used. The method takes into account the migrations of events across the bins and uses data-driven regularisation. It is performed separately for each rapidity bin, since migrations across *p*_T_ bins are significant. The migrations across rapidity bins, which are much smaller, are taken into account using the bin-by-bin unfolding.

The Monte Carlo simulation to derive the transfer matrix is described in Sect. 5. The Monte Carlo samples are reweighted on a jet-by-jet basis as a function of jet *p*_T_ and rapidity. The reweighting factors are obtained from the ratio of calculated cross-sections using the MSTW 2008 NLO PDF set [53] with respect to the MRST 2007 LO* PDF set [38]. This improves the description of the jet *p*_T_ distribution in data. Additionally, a jet selection similar to the jet quality criteria in data is applied to jets with low *p*_T_ in the Monte Carlo simulation at |*η*|∼1.

The transfer matrix for the jet *p*_T_ is derived by matching a particle-level jet to a reconstructed jet based on a geometrical criterion, in which a particle-level jet and a reconstructed jet should be closest to each other within a radius of *R*′=0.3 in the (*η*,*ϕ*)-plane. The spectra of unmatched particle-level and reconstructed jets are used to provide the matching efficiencies, obtained from the number of the matched jets divided by the number of all jets including unmatched jets, both for particle-level jets, *ϵ* ^part^, and for reconstructed jets, *ϵ* ^reco^.

The data are unfolded to particle level using a three-step procedure, namely, correction for matching inefficiency at reconstructed level, unfolding for detector effects and then correction for matching inefficiency at particle level. The final result is given by the equation:

7 $$ N^\mathrm{part}_i=\sum_jN^\mathrm{reco}_j \cdot\epsilon^\mathrm {reco}_j\ A_{ij}\ / \epsilon^\mathrm{part}_i, $$

where *i* and *j* are the particle-level and reconstructed bin indices, respectively, and $N^{\mathrm{part}}_{k}$ and $N^{\mathrm{reco}}_{k}$ are the number of particle-level jets and the number of reconstructed jets in bin *k*. *A*_*ij*_ is an unfolding matrix, which gives the probability for a reconstructed-level jet with a certain reconstructed-level *p*_T_ to have a given particle-level *p*_T_. It is determined using the IDS method. The number of iterations is chosen such that the bias in the closure test (see below) is small and at most at the percent level. In this measurement, this is achieved after one iteration.

The precision of the unfolding technique has been studied using a data-driven closure test [75]. In this study the particle-level *p*_T_ spectrum in the Monte Carlo simulation is reweighted and convolved through the folding matrix, which gives the probability for a particle-level jet with a certain particle-level *p*_T_ to have a given reconstructed-level *p*_T_. The weights are chosen such that significantly improved agreement between the resulting reconstructed spectrum and data is attained. The reconstructed spectrum in this reweighted Monte Carlo simulation is then unfolded using the same procedure as for the data. Comparison of the spectrum obtained from the unfolding with the original reweighted particle-level spectrum provides an estimate of the bias, which is interpreted as the systematic uncertainty.

As an estimate of further systematic uncertainties, the unfolding procedure is repeated using different transfer matrices created with tighter and looser matching criteria of *R*′=0.2 and *R*′=0.4. The deviations of the results from the nominal unfolding result are considered as an additional uncertainty on the unfolding procedure.

The statistical uncertainties are propagated through the unfolding by performing pseudo-experiments. An ensemble of pseudo-experiments is created in which each bin of the transfer matrix is varied according to its statistical uncertainty from the Monte Carlo samples. A separate set of pseudo-experiments is performed in which the data spectrum is fluctuated according to the statistical uncertainty taking the correlation between jets produced in the same event into account. The unfolding is then applied to each pseudo-experiment, and the resulting ensembles are used to calculate the covariance matrix of the corrected spectrum, from which the uncertainties are obtained.

The unfolding procedure is repeated for the propagation of the uncertainties on the jet energy and angle measurements, as described in the next section.

## Systematic uncertainties on the cross-section measurement

The following sources of systematic uncertainty are considered in this measurement: the trigger efficiency, jet reconstruction and calibration, the unfolding procedure and the luminosity measurement.

An uncertainty on the trigger efficiency of 1 % is conservatively chosen for most of the kinematic region (|*y*|<2.8; *p*_T_≥45 GeV in 2.8≤|*y*|<3.6; and *p*_T_≥30 GeV in 3.6≤|*y*|<4.4). A 2 % systematic uncertainty is assigned for jets with *p*_T_<45 GeV in the region 2.8≤|*y*|<3.6 or with *p*_T_<30 GeV in the region 3.6≤|*y*|<4.4, as the triggers are used for *p*_T_ close to the lowest *p*_T_ point with 99 % efficiency for these jets.

The uncertainty on the jet reconstruction efficiency is the same as in the previous measurement at $\sqrt{s}=7~\mbox{TeV}$ [25] and is 2 % for *p*_T_<30 GeV and 1 % for *p*_T_>30 GeV. It is evaluated using jets reconstructed from tracks [73]. The uncertainty on the jet selection efficiency from the measurement at $\sqrt{s}=7~\mbox{TeV}$ is applied in this measurement, but a minimal uncertainty of 0.5 % is retained. The latter accounts for the level of agreement of the central value in the comparison between the used jet selection efficiency and the measured jet selection efficiency at $\sqrt{s} = 2.76~\mbox{TeV}$.

The uncertainty due to the jet energy calibration is evaluated using the same uncertainties on the sources as in the previous measurement at $\sqrt{s}=7~\mbox{TeV}$ [25]. Effects from the systematic uncertainty sources are propagated through the unfolding procedure to provide the uncertainties on the measured cross-sections. The JES uncertainty and its sources are described in detail in Ref. [73], where the total JES uncertainty is found to be less than 2.5 % in the central calorimeter region for jets with 60<*p*_T_<800 GeV, and maximally 14 % for *p*_T_<30 GeV in the most forward region. The JES applied to the reconstructed jets in the Monte Carlo simulation is varied separately for each JES uncertainty source both up and down by one standard deviation. The resulting *p*_T_ spectra are unfolded using the nominal unfolding matrix. The relative shifts with respect to the nominal unfolded spectrum are taken as uncertainties on the cross-section.

The uncertainty on the jet energy resolution (JER) is assigned by considering the difference between data and Monte Carlo simulation in the estimated JER using in situ techniques [76]. The measured resolution uncertainty ranges from 20 % to 10 % for jets within |*y*|<2.8 and with transverse momenta increasing from 30 GeV to 500 GeV. The difference between data and MC is found to be within 10 %. The effect of this uncertainty on the cross-section measurement is evaluated by smearing the energy of reconstructed jets in the Monte Carlo simulation such that the resolution is worsened by the one-standard-deviation uncertainty. Then a new transfer matrix is constructed and used to unfold the data spectra. The relative difference between the cross-sections unfolded with the modified transfer matrix and with the nominal one is taken as the uncertainty in the measurement.

The jet angular resolution is estimated in Monte Carlo simulation from the polar angle between the reconstructed jet and its matched jet at particle level. A new transfer matrix with angular resolution degraded by 10 % is used for the data unfolding, and the relative difference from the nominal unfolded result is assigned as the resulting uncertainty.

The uncertainties in the unfolding procedure are described in Sect. 9. The closure test and the variation of the matching criterion used to construct the transfer matrix are examined. The impact of a possible mis-modelling of the jet *p*_T_ spectrum in the Monte Carlo simulation is assessed in the closure test of the unfolding procedure.

The integrated luminosity is calculated by measuring *pp* interaction rates with several ATLAS devices. The absolute calibration is derived from van der Meer scans [77, 78]. In total, four scan sessions were performed during the collection of the dataset used in the jet cross-section measurements reported here. The uncertainty in the luminosity determination arises from three main contributions: bunch-population measurements, beam conditions during the luminosity calibration scans, and long-term consistency of the different algorithms used to measure the instantaneous luminosity during data collection. The uncertainty on the luminosity for the 2.76 TeV dataset is ±2.7 %, dominated by the irreproducibility of beam conditions during the calibration scans. The total systematic uncertainty for the 2010 dataset at $\sqrt{s} = 7~\mbox{TeV}$ is ±3.4 % [79], dominated by bunch-population measurement uncertainties. Because of significant improvements to the beam instrumentation implemented between the two running periods, and because the dominant systematic uncertainties are of independent origins in the two datasets, these luminosity uncertainties are treated as uncorrelated.

**Fig. 5:**
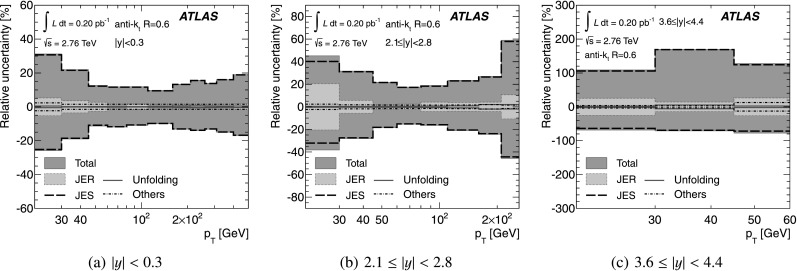
The systematic uncertainty on the inclusive jet cross-section measurement for anti-*k*_*t*_ jets with *R*=0.6 in three representative rapidity bins, as a function of the jet *p*_T_. In addition to the total uncertainty, the uncertainties from the jet energy scale (JES), the jet energy resolution (JER), the unfolding procedure and the other systematic sources are shown separately. The 2.7 % uncertainty from the luminosity measurement and the statistical uncertainty are not shown

The evaluated systematic uncertainties on the measured cross-section are added in quadrature and shown in Fig. 5 for representative rapidity bins and *R*=0.6. Results for jets with *R*=0.4 are similar. The systematic uncertainty on this measurement is driven by the uncertainties on the JES. The very steeply falling jet *p*_T_ spectrum, especially for large rapidity, transforms even relatively modest uncertainties on the transverse momentum into large changes in the measured differential cross-section. The uncertainty on the jet energy resolution also has a sizable effect on the total systematic uncertainty of the measurement in the low *p*_T_ bins. Other sources of uncertainty are found to have a smaller impact on the results.

A total of 22 independent sources of systematic uncertainty have been considered. The correlations of the systematic uncertainties across *p*_T_ and *y* are examined and summarised in Table 1. In the table, 88 independent nuisance parameters describe the correlations of systematic uncertainties over the whole phase space. The systematic effect on the cross-section measurement associated with each nuisance parameter is treated as completely correlated in *p*_T_ and *y*. The table also shows the correlation with respect to the previous $\sqrt{s}=7~\mbox{TeV}$ measurement using 2010 data, which is used in the extraction of the cross-section ratio in Sect. 12.

**Table 1:** Description of the bin-to-bin uncertainty correlation in the measurement of the inclusive jet cross-section at $\sqrt {s}=2.76~\mbox{TeV}$. Each number corresponds to a nuisance parameter for which the corresponding uncertainty is fully correlated in the *p*_T_ of the jet. Bins with the same nuisance parameter are treated as fully correlated, while bins with different nuisance parameters are uncorrelated. Numbers are assigned to be the same as in the previous publication [25]. The sources labelled by *u*_*i*_ are sources uncorrelated in *p*_T_ and *y* of the jet. The correlation with the previous cross-section measurement at $\sqrt {s}=7~\mbox{TeV}$ [25] is indicated in the last column, where full correlation is indicated by a Y and no correlation by a N. The description of the JES uncertainty sources can be found in Refs. [73, 74]. JES14 is a source due to the pile-up correction and is not considered in this measurement. The sources JES6 and JES15 were merged together in the previous measurement and the sum of the two uncertainties added in quadrature is fully correlated with the JES6 in the previous measurement, indicated by the symbol “*” in the table. The nuisance parameter label 31 is skipped in order to be able to keep the same numbers for corresponding nuisance parameters in the two jet cross-section measurements. The values for the nuisance parameters are given in Tables 4–45

Uncertainty source	|*y*| bins							Correlation to 7 TeV
	0–0.3	0.3–0.8	0.8–1.2	1.2–2.1	2.1–2.8	2.8–3.6	3.6–4.4	
Trigger efficiency	*u*_1_	*u*_1_	*u*_1_	*u*_1_	*u*_1_	*u*_1_	*u*_1_	N
Jet reconstruction eff.	83	83	83	83	84	85	86	Y
Jet selection eff.	*u*_2_	*u*_2_	*u*_2_	*u*_2_	*u*_2_	*u*_2_	*u*_2_	N
JES1: Noise thresholds	1	1	2	3	4	5	6	Y
JES2: Theory UE	7	7	8	9	10	11	12	Y
JES3: Theory showering	13	13	14	15	16	17	18	Y
JES4: Non-closure	19	19	20	21	22	23	24	Y
JES5: Dead material	25	25	26	27	28	29	30	Y
JES6: Forward JES generators	88	88	88	88	88	88	88	*
JES7: *E*/*p* response	32	32	33	34	35	36	37	Y
JES8: *E*/*p* selection	38	38	39	40	41	42	43	Y
JES9: EM + neutrals	44	44	45	46	47	48	49	Y
JES10: HAD *E*-scale	50	50	51	52	53	54	55	Y
JES11: High *p*_T_	56	56	57	58	59	60	61	Y
JES12: *E*/*p* bias	62	62	63	64	65	66	67	Y
JES13: Test-beam bias	68	68	69	70	71	72	73	Y
JES15: Forward JES detector	89	89	89	89	89	89	89	*
Jet energy resolution	76	76	77	78	79	80	81	Y
Jet angle resolution	82	82	82	82	82	82	82	Y
Unfolding: Closure test	74	74	74	74	74	74	74	N
Unfolding: Jet matching	75	75	75	75	75	75	75	N
Luminosity	87	87	87	87	87	87	87	N

## Inclusive jet cross-section at $\sqrt{s} = 2.76\mbox{ TeV}$

**Fig. 6:**
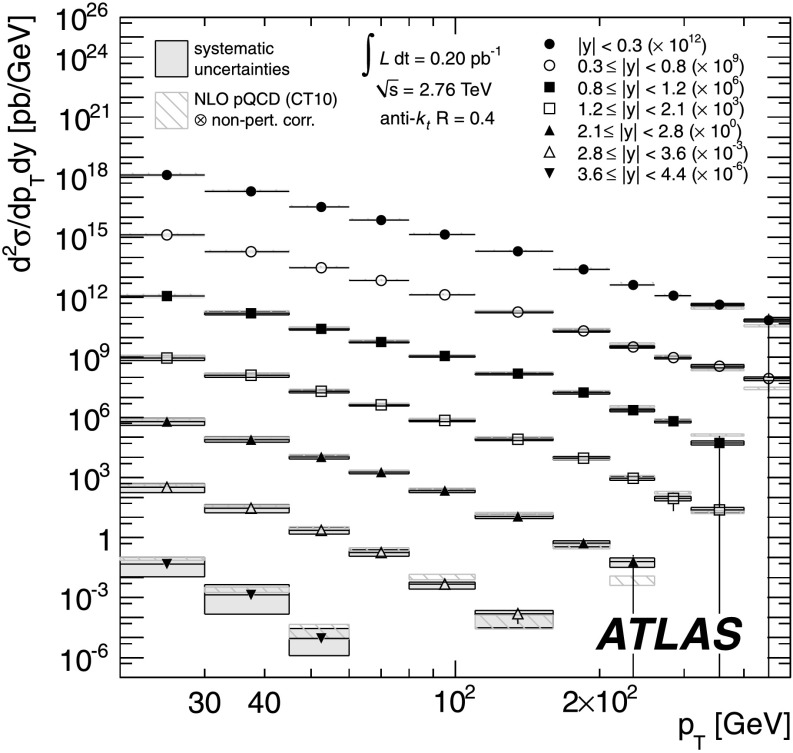
Inclusive jet double-differential cross-section as a function of the jet *p*_T_ in bins of rapidity, for anti-*k*_*t*_ jets with *R*=0.4. For presentation, the cross-section is multiplied by the factors indicated in the legend. The *shaded area* indicates the experimental systematic uncertainties. The data are compared to NLO pQCD predictions calculated using NLOJET++ with the CT10 PDF set, to which non-perturbative corrections have been applied. The *hashed area* indicates the predictions with their uncertainties. The 2.7 % uncertainty from the luminosity measurements is not shown

**Fig. 7:**
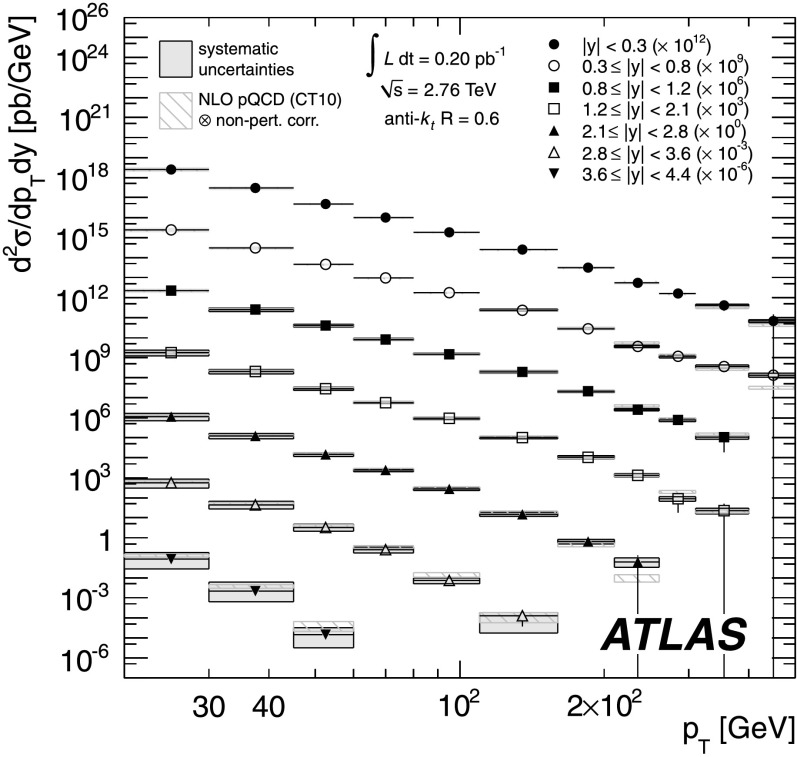
Inclusive jet double-differential cross-section as a function of the jet *p*_T_ in bins of rapidity, for anti-*k*_*t*_ jets with *R*=0.6. For presentation, the cross-section is multiplied by the factors indicated in the legend. The *shaded area* indicates the experimental systematic uncertainties. The data are compared to NLO pQCD predictions calculated using NLOJET++ with the CT10 PDF set, to which non-perturbative corrections have been applied. The *hashed area* indicates the predictions with their uncertainties. The 2.7 % uncertainty from the luminosity measurements is not shown

The inclusive jet double-differential cross-section is shown in Figs. 6 and 7 for jets reconstructed with the anti-*k*_*t*_ algorithm with *R*=0.4 and *R*=0.6, respectively. The measurement spans jet transverse momenta from 20 GeV to 430 GeV in the rapidity region of |*y*|<4.4, covering seven orders of magnitude in cross-section. The results are compared to NLO pQCD predictions calculated with NLOJET++ using the CT10 PDF set. Corrections for non-perturbative effects are applied.

**Fig. 8:**
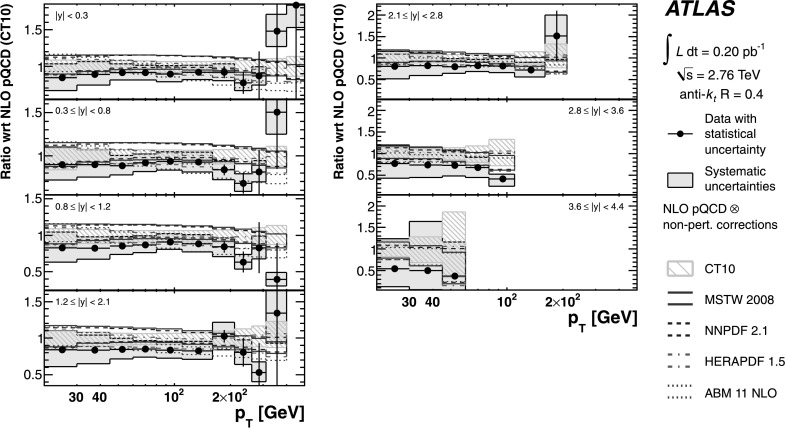
Ratio of the measured inclusive jet double-differential cross-section to the NLO pQCD prediction calculated with NLOJET++ with the CT10 PDF set corrected for non-perturbative effects. The ratio is shown as a function of the jet *p*_T_ in bins of jet rapidity, for anti-*k*_*t*_ jets with *R*=0.4. The figure also shows NLO pQCD predictions obtained with different PDF sets, namely ABM 11, NNPDF 2.1, HERAPDF 1.5 and MSTW2008. Statistically insignificant data points at large *p*_T_ are omitted. The 2.7 % uncertainty from the luminosity measurements is not shown

**Fig. 9:**
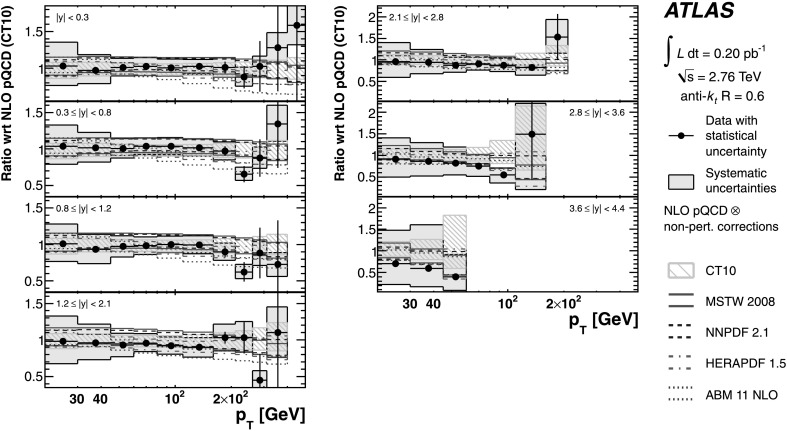
Ratio of the measured inclusive jet double-differential cross-section to the NLO pQCD prediction calculated with NLOJET++ with the CT10 PDF set corrected for non-perturbative effects. The ratio is shown as a function of the jet *p*_T_ in bins of jet rapidity, for anti-*k*_*t*_ jets with *R*=0.6. The figure also shows NLO pQCD predictions obtained with different PDF sets, namely ABM 11, NNPDF 2.1, HERAPDF 1.5 and MSTW2008. Statistically insignificant data points at large *p*_T_ are omitted. The 2.7 % uncertainty from the luminosity measurements is not shown

The ratio of the measured cross-sections to the NLO pQCD predictions using the CT10 PDF set is presented in Figs. 8 and 9 for jets with *R*=0.4 and *R*=0.6, respectively. The results are also compared to the predictions obtained using the PDF sets MSTW 2008, NNPDF 2.1, HERAPDF 1.5 and ABM 11. The measurement is consistent with all the theory predictions using different PDF sets within their systematic uncertainties for jets with both radius parameters. However, the data for jets with *R*=0.4 have a systematically lower cross-section than any of the theory predictions, while such a tendency is seen only in the forward rapidity regions in the measurement for jets with *R*=0.6.

**Fig. 10:**
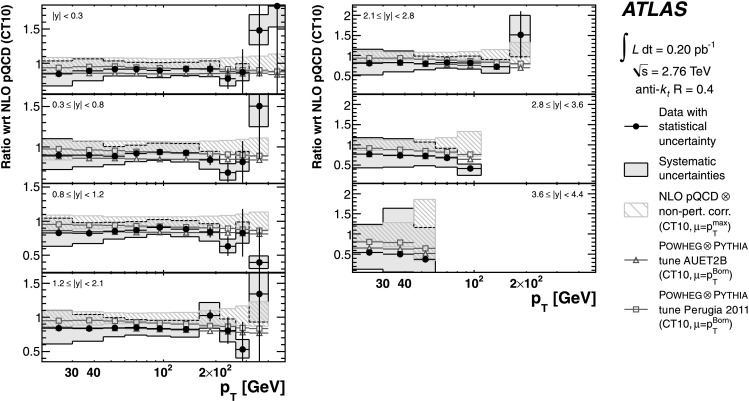
Ratio of the measured inclusive jet double-differential cross-section to the NLO pQCD prediction calculated with NLOJET++ with the CT10 PDF set corrected for non-perturbative effects. The ratio is shown as a function of the jet *p*_T_ in bins of jet rapidity, for anti-*k*_*t*_ jets with *R*=0.4. The figure also shows predictions from Powheg using Pythia for the simulation of the parton shower and hadronisation with the AUET2B tune and the Perugia 2011 tune. Only the statistical uncertainty is shown on the Powheg predictions. Statistically insignificant data points at large *p*_T_ are omitted. The 2.7 % uncertainty from the luminosity measurements is not shown

**Fig. 11:**
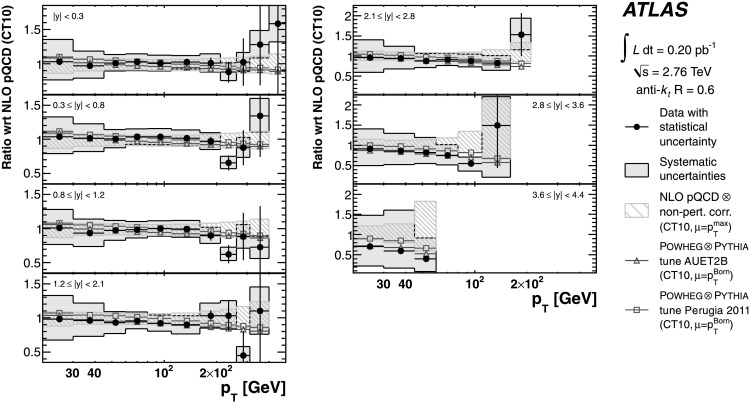
Ratio of the measured inclusive jet double-differential cross-section to the NLO pQCD prediction calculated with NLOJET++ with the CT10 PDF set corrected for non-perturbative effects. The ratio is shown as a function of the jet *p*_T_ in bins of jet rapidity, for anti-*k*_*t*_ jets with *R*=0.6. The figure also shows predictions from Powheg using Pythia for the simulation of the parton shower and hadronisation with the AUET2B tune and the Perugia 2011 tune. Only the statistical uncertainty is shown on the Powheg predictions. Statistically insignificant data points at large *p*_T_ are omitted. The 2.7 % uncertainty from the luminosity measurements is not shown

The comparison of the data with the Powheg prediction for anti-*k*_*t*_ jets with *R*=0.4 and *R*=0.6 is shown in Figs. 10 and 11 as a function of the jet *p*_T_ in bins of rapidity. In general, the Powheg prediction is found to be in good agreement with the data. Especially in the forward region, the shape of the data is very well reproduced by the Powheg prediction, while small differences are observed in the central region. As seen in the previous measurement at $\sqrt{s}=7~\mbox{TeV}$ [25], the Perugia 2011 tune gives a consistently larger prediction than the default Pythia tune AUET2B, which is generally in closer agreement with data. In contrast to the NLO pQCD prediction with corrections for non-perturbative effects, the Powheg prediction agrees well with data for both radius parameters *R*=0.4 and *R*=0.6. This might be attributed to the matched parton shower approach from Powheg (see Sect. 6.3).

## Cross-section ratio of $\sqrt{s}=2.76\mbox{ TeV}$ to $\sqrt {s}=7\mbox{ TeV}$

### Experimental systematic uncertainty

As indicated in Table 1, the systematic uncertainties on the measurement due to jet reconstruction and calibration are considered as fully correlated between the measurements at $\sqrt{s}=2.76~\mbox{TeV}$ and $\sqrt {s}=7~\mbox{TeV}$. For each correlated systematic source *s*_*i*_, the relative uncertainty $\Delta\rho_{s_{i}}/\rho$ on the cross-section ratio is calculated as

8 $$ \frac{\Delta\rho_{s_i}}{\rho}=\frac{1+\delta^{2.76~\text {TeV}}_{s_i}}{1+\delta^{7~\text{TeV}}_{s_i}}-1, $$

where $\delta^{2.76~\text{TeV}}_{s_{i}}$ and $\delta^{7~\text {TeV}}_{s_{i}}$ are relative uncertainties caused by a source *s*_*i*_ in the cross-section measurements at $\sqrt{s}=2.76~\mbox{TeV}$ and $\sqrt{s}=7~\mbox{TeV}$, respectively. Systematic uncertainties that are uncorrelated between the two centre-of-mass energies are added in quadrature. The uncertainties on the trigger efficiency and the jet selection efficiency, and the ones from the unfolding procedure are conservatively considered as uncorrelated between the two measurements at the different energies. The measurement at $\sqrt{s}=7~\mbox{TeV}$ has an additional uncertainty due to pile-up effects in the jet energy calibration. It is added to the uncertainty in the cross-section ratio. The uncertainties in the luminosity measurements are also treated as uncorrelated (see Sect. 10), resulting in a luminosity uncertainty of 4.3 %. The uncertainty on the momentum of the proton beam, based on the LHC magnetic model, is at the level of 0.1 % [80] and highly correlated between different centre-of-mass energies; hence, it is negligible for the ratio.

**Fig. 12:**
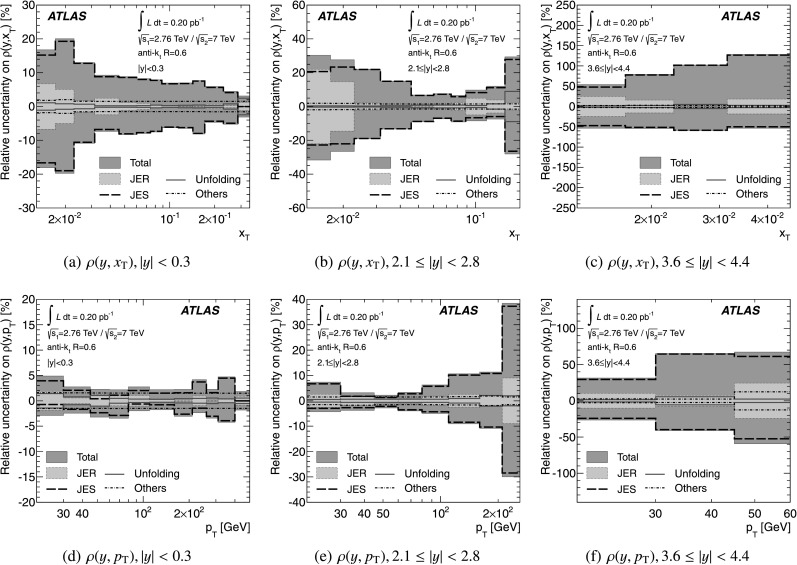
The systematic uncertainty on the cross-section ratios, *ρ*(*y*,*x*_T_) and *ρ*(*y*,*p*_T_), for anti-*k*_*t*_ jets with *R*=0.6 in three representative rapidity bins, as a function of the jet *x*_T_ and of the jet *p*_T_, respectively. In addition to the total uncertainty, the uncertainties from the jet energy scale (JES), the jet energy resolution (JER), the unfolding procedure and other systematic sources are shown separately. The 4.3 % uncertainty from the luminosity measurements and the statistical uncertainty are not shown

The experimental systematic uncertainties on both *ρ*(*y*,*x*_T_) and *ρ*(*y*,*p*_T_) are shown in Fig. 12 for representative rapidity bins for jets with *R*=0.6. For *ρ*(*y*,*x*_T_) the uncertainties are 5 %–20 % for the central jets and ${}^{+160~\%}_{-60~\%}$ for the forward jets. For jets with *R*=0.4, uncertainties are similar, except for central jets with low *p*_T_ where the uncertainty is within ±15 %. A significant reduction of the uncertainty is obtained for *ρ*(*y*,*p*_T_), being well below 5 % in the central region. In the forward region, the uncertainty is ±70 % for jets with *R*=0.6, and ${}^{+100~\%}_{-70~\%}$ for jets with *R*=0.4.

### Results

**Fig. 13:**
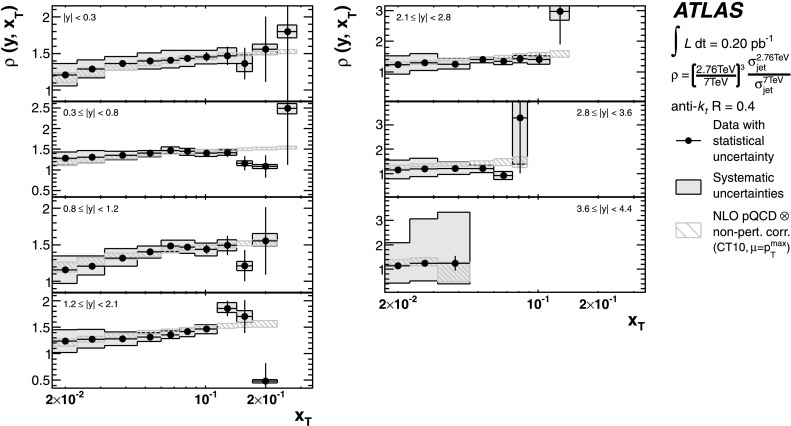
Ratio of the inclusive jet cross-section at $\sqrt {s}=2.76~\mbox{TeV}$ to the one at $\sqrt{s}=7~\mbox{TeV}$ as a function of *x*_T_ in bins of jet rapidity, for anti-*k*_*t*_ jets with *R*=0.4. The theoretical prediction is calculated at next-to-leading order with the CT10 PDF set and corrected for non-perturbative effects. Statistically insignificant data points at large *x*_T_ are omitted. The 4.3 % uncertainty from the luminosity measurements is not shown

**Fig. 14:**
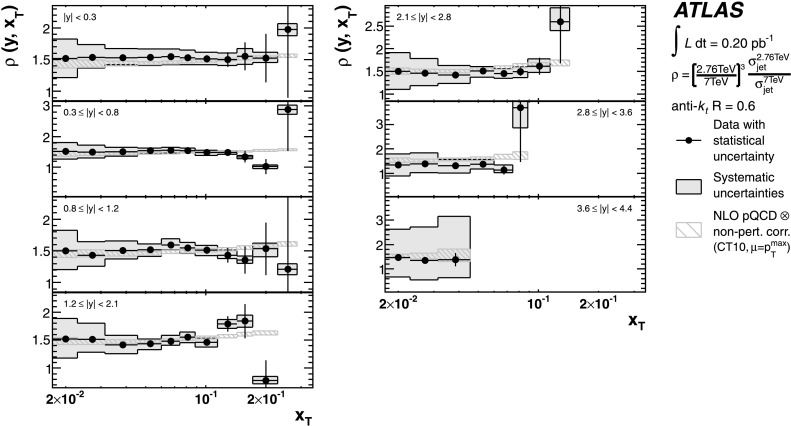
Ratio of the inclusive jet cross-section at $\sqrt {s}=2.76~\mbox{TeV}$ to the one at $\sqrt{s}=7~\mbox{TeV}$ as a function of *x*_T_ in bins of jet rapidity, for anti-*k*_*t*_ jets with *R*=0.6. The theoretical prediction is calculated at next-to-leading order with the CT10 PDF set and corrected for non-perturbative effects. Statistically insignificant data points at large *x*_T_ are omitted. The 4.3 % uncertainty from the luminosity measurements is not shown

Figures 13 and 14 show the extracted cross-section ratio of the inclusive jet cross-section measured at $\sqrt{s}=2.76~\mbox{TeV}$ to the one measured at $\sqrt{s}=7~\mbox{TeV}$, as a function of *x*_T_, for jets with *R*=0.4 and *R*=0.6, respectively. The measured cross-section ratio is found to be 1.1<*ρ*(*y*,*x*_T_)<1.5 for both radius parameters. This approximately constant behaviour reflects both the asymptotic freedom of QCD and evolution of the gluon distribution in the proton as a function of the QCD scale. The measurement shows a slightly different *x*_T_ dependence for jets with *R*=0.4 and *R*=0.6, which may be attributed to different *x*_T_ dependencies of non-perturbative corrections for the two radius parameters, already seen in Figs. 4(a) and 4(b). The measurement is then compared to the NLO pQCD prediction, to which corrections for non-perturbative effects are applied, obtained using the CT10 PDF set. It is in good agreement with the prediction.

**Fig. 15:**
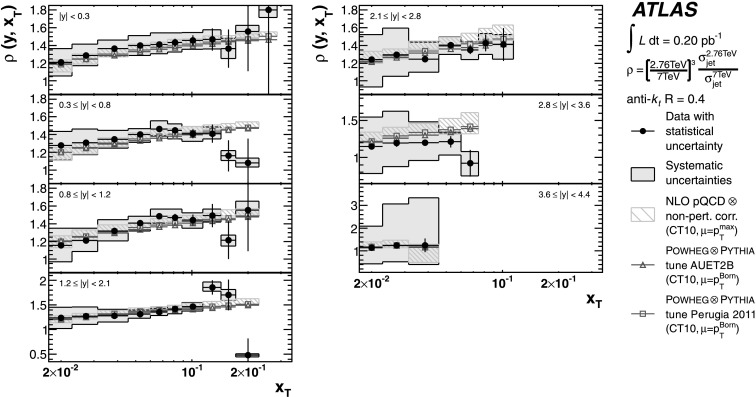
Ratio of the measured inclusive jet double-differential cross-section at $\sqrt{s}=2.76~\mbox{TeV}$ to the one at $\sqrt{s}=7~\mbox{TeV}$ as a function of the jet *x*_T_ in bins of jet rapidity, for anti-*k*_*t*_ jet with *R*=0.4. The theoretical prediction from NLOJET++ is calculated using the CT10 PDF set with corrections for non-perturbative effects applied. Also shown are Powheg predictions using Pythia for the simulation of the parton shower and hadronisation with the AUET2B tune and the Perugia 2011 tune. Only the statistical uncertainty is shown on the Powheg predictions. Statistically insignificant data points at large *x*_T_ are omitted. The 4.3 % uncertainty from the luminosity measurements is not shown

**Fig. 16:**
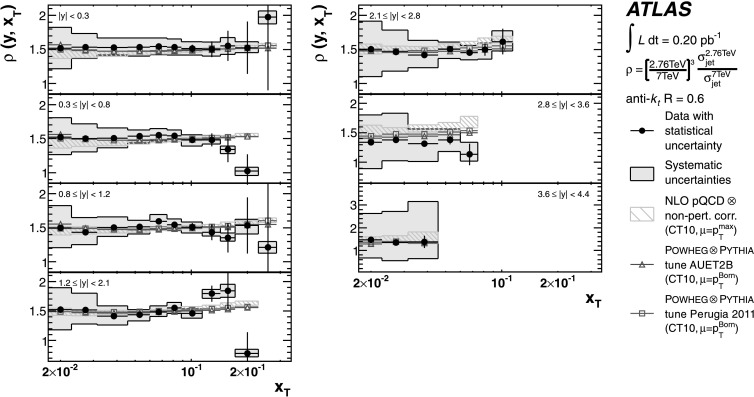
Ratio of the measured inclusive jet double-differential cross-section at $\sqrt{s}=2.76~\mbox{TeV}$ to the one at $\sqrt{s}=7~\mbox{TeV}$ as a function of the jet *x*_T_ in bins of jet rapidity, for anti-*k*_*t*_ jet with *R*=0.6. The theoretical prediction from NLOJET++ is calculated using the CT10 PDF set with corrections for non-perturbative effects applied. Also shown are Powheg predictions using Pythia for the simulation of the parton shower and hadronisation with the AUET2B tune and the Perugia 2011 tune. Only the statistical uncertainty is shown on the Powheg predictions. Statistically insignificant data points at large *x*_T_ are omitted. The 4.3 % uncertainty from the luminosity measurements is not shown

Figures 15 and 16 show the same cross-section ratio compared to predictions from Powheg with the CT10 PDF set. The tunes AUET2B and Perugia 2011 give very similar predictions in general, and also agree well with the pQCD prediction with non-perturbative corrections applied.

**Fig. 17:**
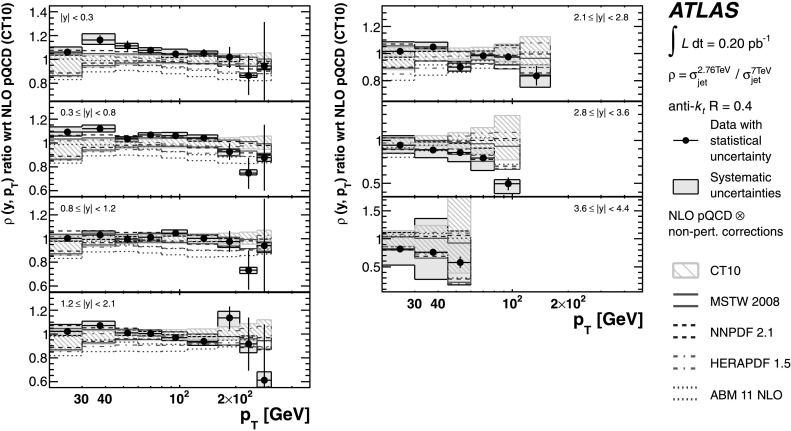
Ratio of the inclusive jet cross-section at $\sqrt {s}=2.76~\mbox{TeV}$ to the one at $\sqrt{s}=7~\mbox{TeV}$, shown as a double ratio to the theoretical prediction calculated with the CT10 PDFs as a function of the jet *p*_T_ in bins of jet rapidity, for anti-*k*_*t*_ jets with *R*=0.4. Statistically insignificant data points at large *p*_T_ are omitted. The 4.3 % uncertainty from the luminosity measurements is not shown

**Fig. 18:**
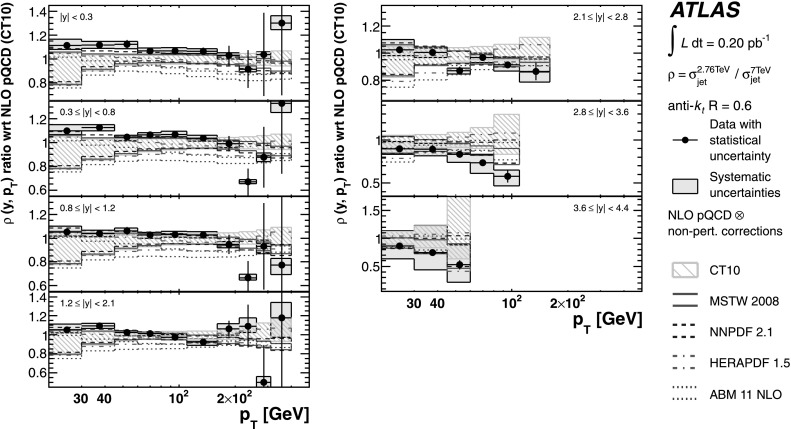
Ratio of the inclusive jet cross-section at $\sqrt {s}=2.76~\mbox{TeV}$ to the one at $\sqrt{s}=7~\mbox{TeV}$, shown as a double ratio to the theoretical prediction calculated with the CT10 PDFs as a function of the jet *p*_T_ in bins of jet rapidity, for anti-*k*_*t*_ jets with *R*=0.6. Statistically insignificant data points at large *p*_T_ are omitted. The 4.3 % uncertainty from the luminosity measurements is not shown

Figures 17 and 18 show the cross-section ratio as a function of the jet *p*_T_, plotted as the double ratio with respect to the NLO pQCD prediction using the CT10 PDF set with non-perturbative corrections applied, for anti-*k*_*t*_ jets with *R*=0.4 and *R*=0.6.^5^

The systematic uncertainty on the measurement is significantly reduced and is generally smaller than the theory uncertainties. The measurement is also compared to the predictions using different PDF sets, namely MSTW2008, NNPDF 2.1, HERAPDF 1.5 and ABM 11. In general, the measured points are slightly higher than the predictions in the central rapidity regions and are lower in the forward rapidity regions. The deviation is more pronounced for the prediction using the ABM 11 PDF set in the barrel region, which yields a different shape with respect to the other PDF sets.

The very small systematic uncertainty in the *ρ*(*y*,*p*_T_) measurement suggests that the measured jet cross-section at $\sqrt{s}=2.76~\mbox{TeV}$ may contribute to constrain the PDF uncertainties in a global PDF fit in the pQCD framework by correctly taking the correlation of systematic uncertainties to the previous $\sqrt{s}=7~\mbox{TeV}$ measurement into account. Such an NLO pQCD analysis is described in Sect. 13.

**Fig. 19:**
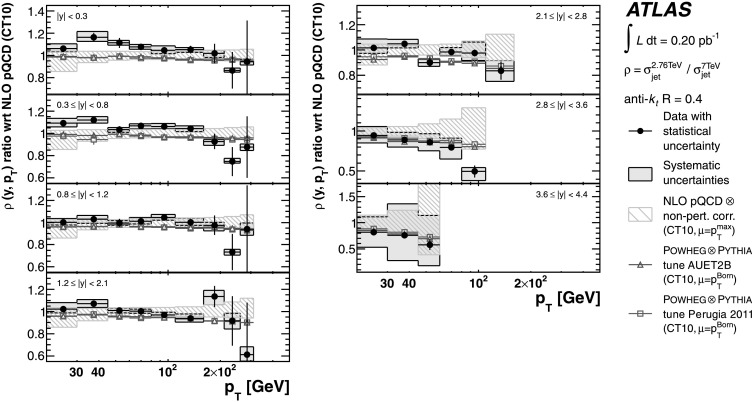
Ratio of the inclusive jet cross-section at $\sqrt {s}=2.76~\mbox{TeV}$ to the one at $\sqrt{s}=7~\mbox{TeV}$, shown as a double ratio to the theoretical prediction calculated with the CT10 PDFs as a function of *p*_T_ in bins of jet rapidity, for anti-*k*_*t*_ jets with *R*=0.4. Also shown are Powheg predictions using Pythia for the simulation of the parton shower and hadronisation with the AUET2B tune and the Perugia 2011 tune. Only the statistical uncertainty is shown on the Powheg predictions. Statistically insignificant data points at large *p*_T_ are omitted. The 4.3 % uncertainty from the luminosity measurements is not shown

**Fig. 20:**
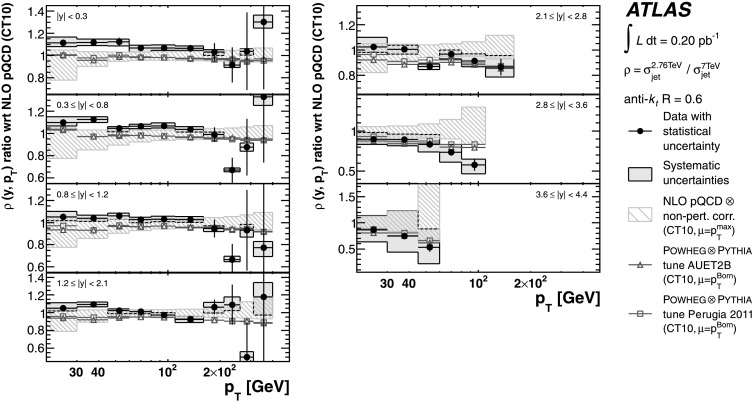
Ratio of the inclusive jet cross-section at $\sqrt {s}=2.76~\mbox{TeV}$ to the one at $\sqrt{s}=7~\mbox{TeV}$, shown as a double ratio to the theoretical prediction calculated with the CT10 PDFs as a function of *p*_T_ in bins of jet rapidity, for anti-*k*_*t*_ jets with *R*=0.6. Also shown are Powheg predictions using Pythia for the simulation of the parton shower and hadronisation with the AUET2B tune and the Perugia 2011 tune. Only the statistical uncertainty is shown on the Powheg predictions. Statistically insignificant data points at large *p*_T_ are omitted. The 4.3 % uncertainty from the luminosity measurements is not shown

A comparison of the jet cross-section ratio as a function of *p*_T_ to the Powheg prediction is made in Figs. 19 and 20. Differences between the tunes used in Pythia for the parton shower are very small, and deviations are seen only in the forward region for large *p*_T_. Like the NLO pQCD prediction with non-perturbative corrections, the Powheg prediction has a different trend in the central rapidity region with respect to data, deviating by more than 10 %. However, it follows the data very well in the forward region.

## NLO pQCD analysis of HERA and ATLAS jet data

Knowledge of the PDFs of the proton comes mainly from deep-inelastic lepton–proton scattering experiments covering a broad range of momentum-transfer squared *Q* ^2^ and of Bjorken *x*. The PDFs are determined from data using pQCD in the DGLAP formalism [81–85]. The quark distributions in the region *x*≲0.01 are in general well constrained by the precise measurement of the proton structure function *F*_2_(*x*,*Q* ^2^) at HERA [86]. However, the gluon momentum distribution *xg*(*x*,*Q* ^2^) at *x* values above 0.01 has not been as precisely determined in deep-inelastic scattering. The inclusive jet *p*_T_ spectrum at low and moderate *p*_T_ is sensitive to the gluon distribution function.

The systematic uncertainty on the jet cross-section at $\sqrt {s}=2.76~\mbox{TeV}$ is strongly correlated with the ATLAS jet cross-section measured at $\sqrt{s}=7~\mbox{TeV}$, as described in Sect. 8. Therefore, increased sensitivity to the PDFs is expected when these two jet cross-section datasets are analysed together, with proper treatment of correlation between the measurements.

A combined NLO pQCD analysis of the inclusive jet cross-section in *pp* collisions at $\sqrt{s}=2.76~\mbox{TeV}$ together with the ATLAS inclusive jet cross-section in *pp* collisions at $\sqrt{s}=7~\mbox{TeV}$ [25] and HERA I data [86] is presented here. The analysis is performed using the HERAFitter package [86–88], which uses the light-quark coefficient functions calculated to NLO as implemented in QCDNUM [89] and the heavy-quark coefficient functions from the variable-flavour number scheme (VFNS) [90, 91] for the PDF evolution, as well as MINUIT [92] for minimisation of *χ* ^2^. The data are compared to the theory using the *χ* ^2^ function defined in Refs. [93–95]. The heavy quark masses are chosen to be *m*_*c*_=1.4 GeV and *m*_*b*_=4.75 GeV [53]. The strong coupling constant is fixed to *α*_S_(*M*_*Z*_)=0.1176, as in Ref. [86]. A minimum *Q* ^2^ cut of $Q^{2}_{\mathrm{min}} = 3.5~\mbox{GeV}^{2}$ is imposed on the HERA data to avoid the non-perturbative region. The prediction for the ATLAS jet data is obtained from the NLO pQCD calculation to which the non-perturbative correction is applied as described in Sect. 6. Due to the large values of the non-perturbative corrections and their large uncertainties at low *p*_T_ of the jet, all the bins with *p*_T_<45 GeV are excluded from the analysis.

The DGLAP evolution equations yield the PDFs at any value of *Q* ^2^, given that they are parameterised as functions of *x* at an initial scale $Q^{2}_{0}$. In the present analysis, this scale is chosen to be $Q^{2}_{0} = 1.9~\mbox{GeV}^{2}$ such that it is below $m_{c}^{2}$. PDFs are parameterised at the evolution starting scale $Q^{2}_{0}$ using a HERAPDF-inspired ansatz as in Ref. [96]:

9 $$\begin{aligned} \begin{aligned} &x u_v(x) = A_{u_v} x^{B_{u_v}} (1-x)^{C_{u_v}} \bigl( 1 + E_{u_v} x^2\bigr), \\ &x d_v(x) = A_{d_v} x^{B_{d_v}} (1-x)^{C_{d_v}}, \\ &x \bar{U} (x) = A_{\bar{U}} x^{B_{\bar{U}}} (1-x)^{C_{\bar{U}}}, \\ &x \bar{D} (x) = A_{\bar{D}} x^{B_{\bar{D}}} (1-x)^{C_{\bar{D}}}, \\ &x g(x) = A_g x^{B_g} (1-x)^{C_g} - A'_gx^{B'_g}(1-x)^{C'_g} . \end{aligned} \end{aligned}$$

Here $\bar{U}=\bar{u}$ whereas $\bar{D}=\bar{d} + \bar{s}$. The parameters $A_{u_{v}}$ and $A_{d_{v}}$ are fixed using the quark counting rule and *A*_*g*_ using the momentum sum rule. The normalisation and slope parameters, *A* and *B*, of $\bar{u}$ and $\bar{d}$ are set equal such that $x\bar{u} = x\bar{d}$ at *x*→0. An extra term for the valence distribution ($E_{u_{v}}$) is observed to improve the fit quality significantly. The strange-quark distribution is constrained to a certain fraction of $\bar{D}$ as $x\bar{s}=f_{s}x\bar{D}$, where *f*_*s*_=0.31 is chosen in this analysis. The gluon distribution uses the so-called flexible form, suggested by MSTW analyses, with $C'_{g}=25$ [53]. This value of the $C'_{g}$ parameter ensures that the additional term contributes at low *x* only. With all these additional constraints applied, the fit has 13 free parameters to describe the parton densities.

To see the impact of the ATLAS jet data on the PDFs, a fit only to the HERA dataset is performed first. Then, the fit parameters are fixed and the *χ* ^2^ value between jet data and the fit prediction is calculated using experimental uncertainties only. The data are included taking into account bin-to-bin correlations. Finally, fits to HERA + ATLAS jet data are performed for *R*=0.4 and *R*=0.6 jet sizes independently, since correlations of uncertainties between measurements based on two different jet radius parameters have not been determined. The correlations of systematic uncertainties between the $\sqrt {s}=7~\mbox{TeV}$ and $\sqrt{s}=2.76~\mbox{TeV}$ datasets are treated as described in Sect. 12. The PDF uncertainties are determined using the Hessian method [97, 98].

The consistency of the PDF fit with different datasets in terms of the *χ* ^2^ values is given in Appendix B. Very good fit quality is found for both radius parameters. The *χ* ^2^ values also show the pull of ATLAS jet data for both jet radius parameters, while the description of the HERA data is almost unaffected.

The fits determine shifts for the correlated systematic uncertainties in the data, which are applied to the theory predictions. Typically these shifts are smaller than half a standard deviation and comparable in size for the fits to the two different jet radius parameters. Larger differences are found for the normalisation parameters in the fit using the $\sqrt{s}=2.76~\mbox{TeV}$ jet data, being 0.0 % for *R*=0.4 and −2.4 % for *R*=0.6, respectively, in spite of the fact that the integrated luminosity is the same in the two cases and thus 100 % correlated. Since this correlation is not implemented in the fitting method, the differences between the data and theory prediction for jets with *R*=0.4 and *R*=0.6 (see Sect. 11) are compensated using shifts of the nuisance parameters. Interestingly, the gluon PDFs obtained from the two fits are very similar. Additional studies where the normalisation is fixed in the fit show that the impact of the difference in normalisation on the parton distributions is small.

In the fits using the HERA data and both of the ATLAS jet datasets at the different centre-of-mass energies, the shifts of jet-related systematic uncertainties modelled by 88 nuisance parameters contribute 19 (12) units in total to the correlated components of the *χ* ^2^ for the fit using *R*=0.4 (*R*=0.6). A few shifts of jet systematic uncertainties are found to be different between the *R*=0.4 and *R*=0.6 fits, e.g. the jet energy resolution in forward rapidity bins differs by ∼0.5*σ*. In order to evaluate the impact of the larger shifts on the fit parameters, a special fit is performed in which several systematic uncertainties with the largest shifts are treated as uncorrelated. In these special fits, the PDF parameters in Eq. (9) are found to be compatible with the results of the default fits.

**Fig. 21:**
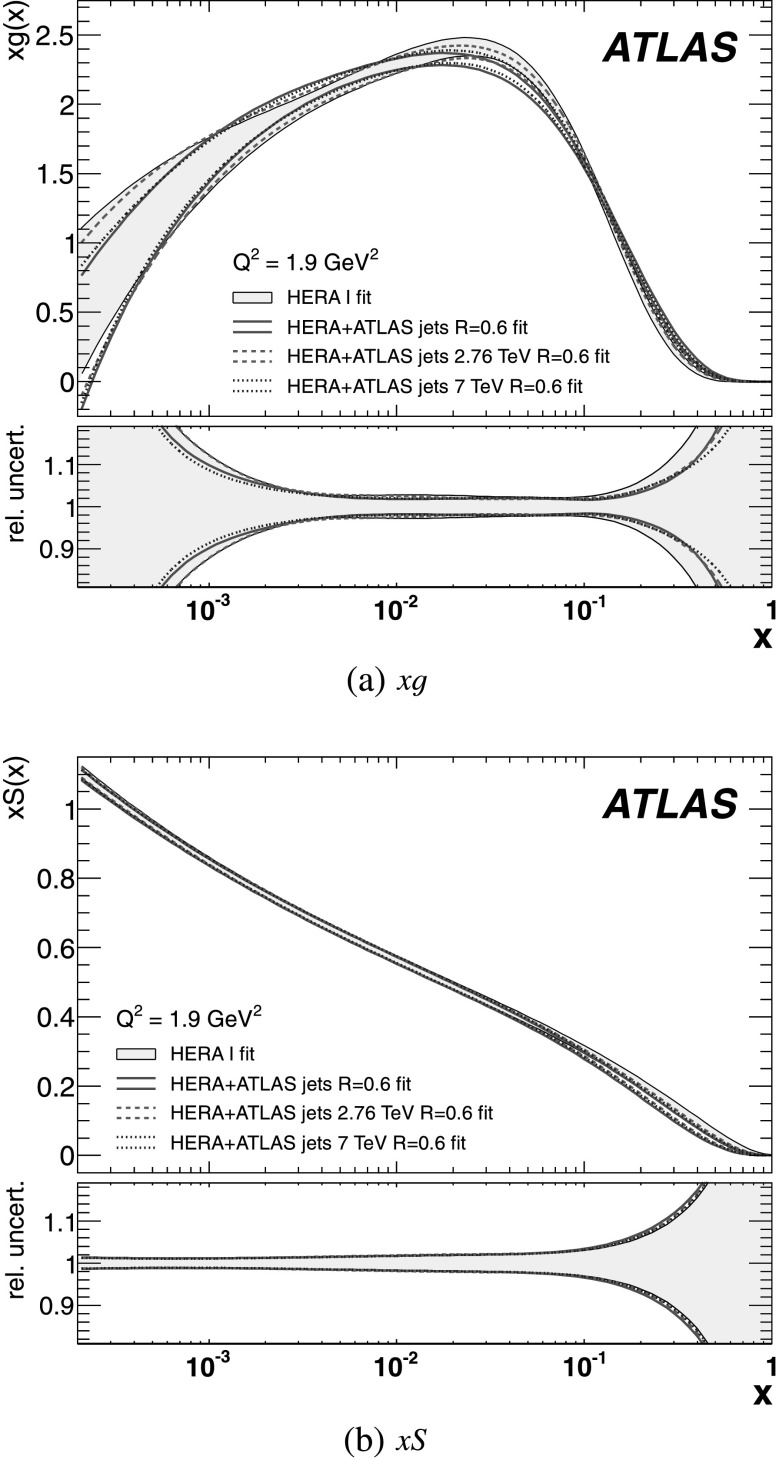
Momentum distributions of the (**a**) gluon *xg*(*x*) and (**b**) sea quarks *xS*(*x*) together with their relative experimental uncertainty as a function of *x* for *Q* ^2^=1.9 GeV^2^. The filled area indicates a fit to HERA data only. The bands show fits to HERA data in combination with both ATLAS jet datasets, and with the individual ATLAS jet datasets separately, each for jets with *R*=0.6. For each fit the uncertainty in the PDF is centred on unity

In the following, the results for the fit using jet data with *R*=0.6 are presented. The results for *R*=0.4 are compatible. The results of the fits to HERA data and to the combined data from HERA and ATLAS jet measurements are presented in Fig. 21, which shows the momentum distribution of the gluon *xg* and sea quarks $xS=2(x\bar{u}+x\bar{d}+x\bar{s})$ at the scale *Q* ^2^=1.9 GeV^2^. The gluon momentum distribution tends to be harder after the inclusion of the jet data than that obtained from HERA data alone. Furthermore, the uncertainty in *xg* is reduced if the ATLAS jet data are included in the fit. Being smaller in the high-*x* region, the sea quark momentum distribution tends to be softer with the ATLAS jet data used in the fit. This reduction of the central value results in a larger relative uncertainty on *xS*.

The fit is also performed for HERA data in combination with the ATLAS jet data at $\sqrt{s}=2.76~\mbox{TeV}$ and $\sqrt{s}=7~\mbox{TeV}$ separately (see Fig. 21). It is found that the impact on the gluon momentum distribution is largely reduced. Hence, the full potential of the ATLAS jet data for PDF fits can be exploited only when both datasets and the information about the correlations are used.

**Fig. 22:**
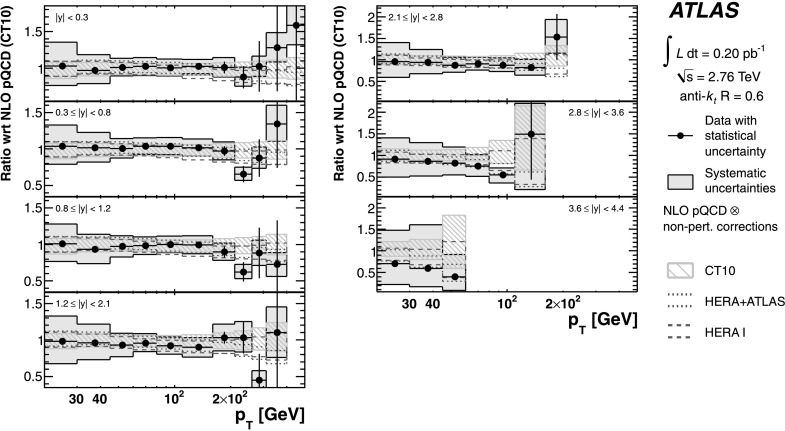
Comparison of NLO pQCD predictions of the jet cross-section at $\sqrt{s}=2.76~\mbox{TeV}$ calculated with the CT10 PDF set, the fitted PDF set using the HERA data only and the one using HERA data and the ATLAS jet data with *R*=0.6. The predictions are normalised to the one using the CT10 PDF set. Also shown is the measured jet cross-section. The 2.7 % uncertainty from the luminosity measurement is not shown

**Fig. 23:**
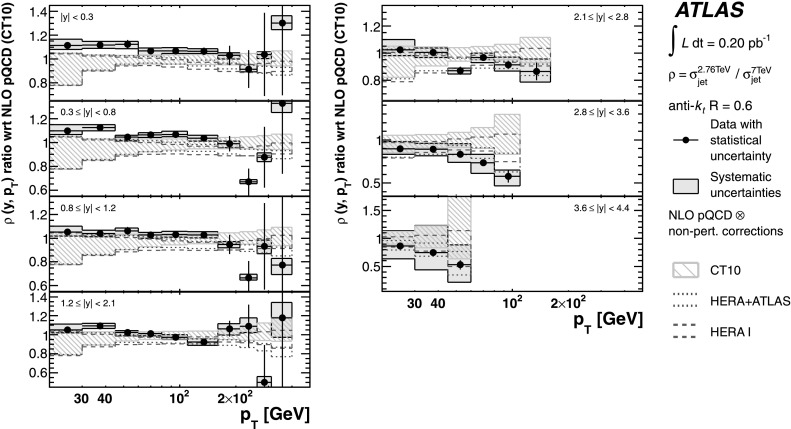
Comparison of NLO pQCD predictions of the jet cross-section ratio of $\sqrt{s}=2.76~\mbox{TeV}$ to $\sqrt {s}=7~\mbox{TeV}$ calculated with the CT10 PDF set, the fitted PDF set using the HERA data only and the one using HERA data and the ATLAS jet data with *R*=0.6. The predictions are normalised to the one using the CT10 PDF set. Also shown is the measured jet cross-section ratio. The 4.3 % uncertainty from the luminosity measurements is not shown

The measured jet cross-section and the cross-section ratio, *ρ*(*y*,*p*_T_), are compared to the predictions based on fitted PDF sets in Figs. 22 and 23, respectively. The data are well described by the prediction based on the refit PDFs after the addition of the jet data. The description is particularly improved in the forward region.

## Conclusion

The inclusive jet cross-section in *pp* collisions at $\sqrt {s}=2.76~\mbox{TeV}$ has been measured for jets reconstructed with the anti-*k*_*t*_ algorithm with two radius parameters of *R*=0.4 and *R*=0.6, based on the data collected using the ATLAS detector at the beginning of 2011 LHC operation, corresponding to an integrated luminosity of $0.20~\mbox {pb$^{-1}$}$. The measurement is performed as a function of the jet transverse momentum, in bins of jet rapidity.

The ratio of the inclusive jet cross-sections at $\sqrt{s}=2.76~\mbox {TeV}$ and $\sqrt{s}=7~\mbox{TeV}$ is shown in this paper. The correlation of the sources of uncertainty common to the two measurements is fully taken into account, resulting in a reduction of systematic uncertainties in the ratio measurement.

The measurements are compared to fixed-order NLO perturbative QCD calculations, to which corrections for non-perturbative effects are applied. The comparison is performed with five different PDF sets. The predictions are in good agreement with the data in general, in both the jet cross-section and the cross-section ratio. This confirms that perturbative QCD can describe jet production at high jet transverse momentum. Due to the reduced systematic uncertainties, the ratio measurement starts to show preferences for certain PDF sets. The measurement is also compared to predictions from NLO matrix elements with matched parton-shower Monte Carlo simulation. In particular in the forward region, the central value of the prediction describes the data well.

An NLO pQCD analysis in the DGLAP formalism has been performed using the ATLAS inclusive jet cross-section data at $\sqrt{s}=2.76~\mbox {TeV}$ and $\sqrt{s}=7~\mbox{TeV}$, together with HERA I data. By including the ATLAS jet data, a harder gluon distribution and a softer sea-quark distribution in the high Bjorken-*x* region are obtained with respect to the fit of HERA data only. Furthermore, it is shown that the full potential of the ATLAS jet data for PDF fits can be exploited further when the information about the correlations between the measurements at $\sqrt{s}=2.76~\mbox{TeV}$ and $\sqrt {s}=7~\mbox{TeV}$ is used.

## Appendix A: Bin boundaries of the measured cross-section

The bin boundaries of the cross-section measurement are given in Table 2 for the *x*_T_ binning, and for the *p*_T_ binning at both centre-of-mass energies, $\sqrt{s}=7~\mbox{TeV}$ and $\sqrt {s}=2.76~\mbox{TeV}$.

**Table 2:** Bin boundaries in the variable *x*_T_ used in the extraction of *ρ*(*y*,*x*_T_), the cross-section ratio as a function of *x*_T_ at different centre-of-mass energies. Also shown are the corresponding jet *p*_T_ values at each centre-of-mass energy

*x*_T_	*p*_T_ [GeV] at $\sqrt {s}=7~\mbox{TeV}$	*p*_T_ [GeV] at $\sqrt{s}=2.76~\mbox{TeV}$
0.0171	60	23.65
0.0229	80	31.54
0.0314	110	43.37
0.0457	160	63.08
0.0600	210	82.80
0.0743	260	102.5
0.0886	310	122.2
0.1143	400	157.7
0.1429	500	197.1
0.1714	600	236.5
0.2286	800	315.4
0.2857	1000	394.2
0.3429	1200	473.1

## Appendix B: Tables of *χ*^2^ values for the PDF fit

Table 3 shows the *χ* ^2^ values for the PDF fits with various combinations of HERA and ATLAS jet data for different radius parameters *R* of the anti-*k*_*t*_ algorithm.

**Table 3:** Agreement of the PDF fit with different datasets in terms of the *χ* ^2^ values, using various combinations of input datasets for the fit. HERA is used as baseline and in combination with ATLAS jet data from the single-jet double-differential cross-section measurements at $\sqrt{s} = 2.76~\mbox{TeV}$ and $\sqrt {s} = 7~\mbox{TeV}$ for anti-*k*_*t*_ jets with two radius parameters, *R*=0.4 and *R*=0.6. When both ATLAS jet datasets at $\sqrt{s} = 2.76~\mbox{TeV}$ and $\sqrt{s} = 7~\mbox{TeV}$ are used, all correlations between the measurements are taken into account. The *χ* ^2^ value of the fit with respect to the individual datasets tested is given separately for the uncorrelated and the correlated components as $\chi^{2}_{\mathrm{uncor}}$ and $\chi^{2}_{\mathrm{cor}}$, where the shifts of the systematic uncertainties are summed in quadrature for each category. In general, a very good fit quality is found. Comparison of *χ* ^2^ values of the fit using HERA data only with fits including both HERA and ATLAS jet data shows the pull of ATLAS jet data for both jet radius parameters, while the description of the HERA  data is almost unaffected. For example, $\chi^{2}_{\mathrm{uncor}}$ of the HERA dataset worsens only slightly by 8 units from 556 to 564 when the ATLAS jet data for *R*=0.6 is included in the fit, while $\chi^{2}_{\mathrm{uncor}}$ improved from 33 (50) to 29 (40) for the 2.76 TeV (7 TeV) jet data. There is also an improvement from 22 to 12 in $\chi^{2}_{\mathrm{cor}}$ for jets

Input datasets	Test dataset	$\chi^{2}_{\mathrm{uncor}}$	$\chi ^{2}_{\mathrm{cor}}$	*N*_points_
HERA	HERA	556	3.0	592
	ATLAS jets 2.76 TeV, *R*=0.4	29	21	40
	ATLAS jets 7 TeV, *R*=0.4	44		76
	ATLAS jets 2.76 TeV, *R*=0.6	33	22	40
	ATLAS jets 7 TeV, *R*=0.6	50		76
HERA ATLAS jets 2.76 TeV, *R*=0.4 ATLAS jets 7 TeV, *R*=0.4	HERA	562	3.6	592
	ATLAS jets 2.76 TeV, *R*=0.4	27	19	40
	ATLAS jets 7 TeV, *R*=0.4	33		76
	ATLAS jets 2.76 TeV, *R*=0.6	29	13	40
	ATLAS jets 7 TeV, *R*=0.6	41		76
HERA ATLAS jets 2.76 TeV, *R*=0.4	HERA	557	3.1	592
	ATLAS jets 2.76 TeV, *R*=0.4	20	7.4	40
HERA ATLAS jets 7 TeV, *R*=0.4	HERA	559	3.4	592
	ATLAS jets 7 TeV, *R*=0.4	28	14	76
HERA ATLAS jets 2.76 TeV, *R*=0.6 ATLAS jets 7 TeV, *R*=0.6	HERA	564	4.0	592
	ATLAS jets 2.76 TeV, *R*=0.6 jets	29	12	40
	ATLAS jets 7 TeV, *R*=0.6	40		76
	ATLAS jets 2.76 TeV, *R*=0.4	26	18	40
	ATLAS jets 7 TeV, *R*=0.4	32		76
HERA ATLAS jets 2.76 TeV, *R*=0.6	HERA	558	3.2	592
	ATLAS jets 2.76 TeV, *R*=0.6	21	4.9	40
HERA ATLAS jets 7 TeV, *R*=0.6	HERA	560	3.6	592
	ATLAS jets 7 TeV, *R*=0.6	34	9.4	76

## Appendix C: Tables of the measured jet cross-sections and cross-section ratios

The measured inclusive single-jet cross-sections are shown in Tables 4–10 and Tables 11–17 for jets with *R*=0.4 and *R*=0.6, respectively. Tables 18–24 and 25–31 show the measured cross-section ratio, *ρ*(*y*,*x*_T_), for *R*=0.4 and *R*=0.6, and Tables 32–38 and Tables 39–45 show the measured cross-section ratio, *ρ*(*y*,*p*_T_), for *R*=0.4 and *R*=0.6, respectively.

**Table 4:** Measured jet cross section for anti-*k*_*t*_ jets with *R*=0.4 in the rapidity bin |*y*|<0.3. NPCorr stands for multiplicative non-perturbative corrections, *σ* is the cross section and $\delta_{\mathrm{stat.}}$ is the statistical uncertainty. *γ*_*i*_ and *u*_*j*_ correspond to the correlated and uncorrelated systematic uncertainties given in %. They are described in Table 1, where *i* in *γ*_*i*_ denotes a nuisance parameter. For each table entry, the outcome of shifting the corresponding nuisance parameter by one standard deviation, up or down, is shown as superscript or subscript, respectively. In some bins these shifts may lead to cross-section variations in the same direction. The 2.7 % uncertainty from the luminosity measurement is not in the table

*p*_T_ (GeV)	NPCorr	*σ* (nb/GeV)	$\delta_{\mathrm{stat.}}$ %	*γ*_1_ %	*γ*_7_ %	*γ*_13_ %	*γ*_19_ %	*γ*_25_ %	*γ*_88_ %	*γ*_32_ %	*γ*_38_ %	*γ*_44_ %	*γ*_50_ %	*γ*_56_ %	*γ*_62_ %	*γ*_68_ %	*γ*_89_ %	*γ*_76_ %	*γ*_82_ %	*γ*_74_ %	*γ*_75_ %	*γ*_83_ %	*u*_1_ %	*u*_2_ %
20–30	$0.88^{+ 0.07}_{- 0.11}$	1.308e+03	1.7	${}^{+13.}_{-9.6}$	${}^{ +12}_{ -10}$	${}^{+ 7.8}_{-7.4}$	${}^{+ 7.2}_{-9.2}$	${}^{+ 4.9}_{-4.0}$	0.0	${}^{+ 1.8}_{-2.1}$	${}^{+ 5.3}_{-5.0}$	${}^{+ 4.5}_{-4.7}$	${}^{-0.0}_{+ 0.0}$	${}^{+ 0.0}_{-0.0}$	${}^{+ 1.3}_{-2.1}$	${}^{-0.0}_{+ 0.0}$	0.0	±4.6	∓0.1	∓0.1	±0.3	±2.0	±1.0	±0.5
30–45	$0.91^{+ 0.06}_{- 0.09}$	1.945e+02	2.9	${}^{+ 4.6}_{-3.5}$	${}^{+ 9.6}_{-8.0}$	${}^{+ 6.7}_{-5.4}$	${}^{+12.}_{-9.4}$	${}^{+ 1.8}_{-1.2}$	0.0	${}^{+ 3.3}_{-2.5}$	${}^{+ 4.4}_{-3.5}$	${}^{+ 5.1}_{-4.3}$	${}^{-0.0}_{+ 0.0}$	${}^{+ 0.0}_{-0.0}$	${}^{+ 4.4}_{-3.7}$	${}^{-0.0}_{+ 0.0}$	0.0	±3.5	∓0.1	±0.0	±0.1	±1.0	±1.0	±0.5
45–60	$0.93^{+ 0.06}_{- 0.06}$	3.317e+01	2.6	${}^{+ 1.5}_{-1.2}$	${}^{+ 7.6}_{-6.9}$	${}^{+ 4.2}_{-3.4}$	${}^{+ 5.6}_{-4.5}$	${}^{+ 0.5}_{-0.2}$	0.0	${}^{+ 3.2}_{-2.6}$	${}^{+ 2.4}_{-1.9}$	${}^{+ 4.3}_{-3.8}$	${}^{+ 0.3}_{-0.0}$	${}^{+ 0.1}_{+ 0.0}$	${}^{+ 5.0}_{-4.4}$	${}^{-0.0}_{-0.0}$	0.0	±1.4	±0.1	±0.2	±0.1	±1.0	±1.0	±0.5
60–80	$0.95^{+ 0.06}_{- 0.05}$	7.315e+00	1.1	${}^{-0.8}_{+ 0.7}$	${}^{+ 6.6}_{-6.8}$	${}^{+ 2.4}_{-2.5}$	${}^{+ 3.9}_{-3.8}$	${}^{+ 1.0}_{-0.9}$	0.0	${}^{+ 3.1}_{-3.1}$	${}^{+ 1.6}_{-1.5}$	${}^{+ 4.6}_{-4.5}$	${}^{+ 0.5}_{-0.3}$	${}^{+ 0.1}_{-0.1}$	${}^{+ 4.7}_{-4.3}$	${}^{-0.1}_{+ 0.1}$	0.0	±0.4	±0.1	±0.2	±0.0	±1.0	±1.0	±0.5
80–110	$0.97^{+ 0.05}_{- 0.05}$	1.378e+00	1.7	${}^{-0.5}_{+ 0.4}$	${}^{+ 8.2}_{-7.5}$	${}^{+ 4.5}_{-4.2}$	${}^{+ 4.0}_{-3.7}$	${}^{+ 2.2}_{-2.3}$	0.0	${}^{+ 3.6}_{-3.4}$	${}^{+ 1.4}_{-1.7}$	${}^{+ 5.4}_{-5.0}$	${}^{+ 1.0}_{-1.2}$	${}^{+ 0.1}_{-0.1}$	${}^{+ 2.5}_{-2.5}$	${}^{-0.1}_{+ 0.1}$	0.0	±1.3	∓0.1	±0.2	±0.0	±1.0	±1.0	±0.5
110–160	$0.98^{+ 0.04}_{- 0.04}$	1.987e-01	3.2	${}^{-0.1}_{+ 0.0}$	${}^{+ 5.7}_{-5.6}$	${}^{+ 3.8}_{-4.0}$	${}^{+ 3.6}_{-3.8}$	${}^{+ 0.7}_{-0.7}$	0.0	${}^{+ 3.1}_{-3.3}$	${}^{+ 1.0}_{-1.0}$	${}^{+ 5.7}_{-5.9}$	${}^{+ 1.7}_{-1.9}$	${}^{+ 0.1}_{-0.2}$	${}^{-0.2}_{+ 0.1}$	${}^{+ 1.4}_{-1.6}$	0.0	±1.3	±0.1	±0.3	±0.0	±1.0	±1.0	±0.5
160–210	$0.99^{+ 0.03}_{- 0.03}$	2.552e-02	8.6	${}^{-0.0}_{+ 0.0}$	${}^{+ 4.3}_{-4.6}$	${}^{+ 4.5}_{-5.0}$	${}^{+ 3.1}_{-3.1}$	${}^{+ 1.2}_{-1.1}$	0.0	${}^{+ 2.9}_{-3.1}$	${}^{+ 0.8}_{-0.7}$	${}^{+ 7.2}_{-7.0}$	${}^{+ 2.8}_{-2.9}$	${}^{+ 0.3}_{-0.3}$	${}^{-0.3}_{+ 0.3}$	${}^{+ 5.6}_{-5.8}$	0.0	±1.5	±0.2	±0.6	±0.0	±1.0	±1.0	±0.5
210–260	$0.99^{+ 0.03}_{- 0.03}$	4.231e-03	19.	${}^{-0.0}_{+ 0.0}$	${}^{+ 5.9}_{-5.8}$	${}^{+ 6.2}_{-5.9}$	${}^{+ 2.3}_{-2.4}$	${}^{+ 1.8}_{-1.7}$	0.0	${}^{+ 3.0}_{-2.9}$	${}^{+ 0.6}_{-0.7}$	${}^{+ 7.1}_{-6.6}$	${}^{+ 4.0}_{-3.7}$	${}^{+ 0.3}_{-0.4}$	${}^{-0.1}_{+ 0.1}$	${}^{+ 7.2}_{-6.9}$	0.0	∓0.1	±0.1	±0.6	±0.0	±1.0	±1.0	±0.5
260–310	$0.99^{+ 0.03}_{- 0.03}$	1.196e-03	39.	${}^{-0.0}_{+ 0.0}$	${}^{+ 5.9}_{-5.6}$	${}^{+ 4.1}_{-3.9}$	${}^{+ 2.0}_{-1.8}$	${}^{+ 1.9}_{-1.9}$	0.0	${}^{+ 2.9}_{-2.6}$	${}^{+ 0.6}_{-0.6}$	${}^{+ 7.1}_{-7.3}$	${}^{+ 4.7}_{-4.7}$	${}^{+ 0.3}_{-0.5}$	${}^{-0.0}_{+ 0.0}$	${}^{+ 7.9}_{-8.0}$	0.0	±0.1	±0.1	±0.6	±0.0	±1.0	±1.0	±0.5
310–400	$0.99^{+ 0.03}_{- 0.03}$	4.272e-04	46.	${}^{-0.0}_{+ 0.0}$	${}^{+ 3.9}_{-3.6}$	${}^{+ 2.4}_{-2.4}$	${}^{+ 1.0}_{-1.1}$	${}^{+ 2.3}_{-2.5}$	0.0	${}^{+ 2.2}_{-2.2}$	${}^{+ 0.4}_{-0.4}$	${}^{+ 8.7}_{-7.7}$	${}^{+ 5.7}_{-5.3}$	${}^{+ 0.4}_{-0.4}$	${}^{-0.0}_{+ 0.0}$	${}^{+ 9.9}_{-8.9}$	0.0	±0.6	∓0.1	±0.4	±0.0	±1.0	±1.0	±0.5
400–500	$0.99^{+ 0.03}_{- 0.03}$	7.039e-05	115	${}^{-0.0}_{+ 0.0}$	${}^{+ 1.1}_{-1.1}$	${}^{+ 0.7}_{-0.7}$	${}^{+ 3.5}_{-3.1}$	${}^{+ 3.7}_{-3.3}$	0.0	${}^{+ 2.3}_{-2.3}$	${}^{+ 0.7}_{-0.7}$	${}^{+10.}_{-9.1}$	${}^{+ 7.7}_{-7.2}$	${}^{+ 0.9}_{-0.8}$	${}^{-0.0}_{+ 0.0}$	${}^{ +12}_{ -11}$	0.0	±1.0	±0.3	±0.5	±0.0	±1.0	±1.0	±0.5

**Table 5:** Measured jet cross section for anti-*k*_*t*_ jets with *R*=0.4 in the rapidity bin 0.3≤|*y*|<0.8. See caption of Table 4 for details

*p*_T_ (GeV)	NPCorr	*σ* (nb/GeV)	$\delta_{\mathrm{stat.}}$ %	*γ*_1_ %	*γ*_7_ %	*γ*_13_ %	*γ*_19_ %	*γ*_25_ %	*γ*_88_ %	*γ*_32_ %	*γ*_38_ %	*γ*_44_ %	*γ*_50_ %	*γ*_56_ %	*γ*_62_ %	*γ*_68_ %	*γ*_89_ %	*γ*_76_ %	*γ*_82_ %	*γ*_74_ %	*γ*_75_ %	*γ*_83_ %	*u*_1_ %	*u*_2_ %
20–30	$0.88^{+ 0.07}_{- 0.12}$	1.304e+03	1.4	${}^{+13.}_{-9.1}$	${}^{+ 6.6}_{-8.4}$	${}^{+12.}_{-9.3}$	${}^{+ 6.2}_{-7.5}$	${}^{+ 4.3}_{-4.0}$	${}^{+ 0.1}_{-0.1}$	${}^{+ 2.0}_{-2.3}$	${}^{+ 4.5}_{-4.3}$	${}^{+ 4.6}_{-4.7}$	${}^{-0.0}_{+ 0.0}$	${}^{+ 0.0}_{-0.0}$	${}^{+ 2.7}_{-3.0}$	${}^{-0.0}_{+ 0.0}$	${}^{+ 0.1}_{-0.0}$	±5.7	±0.0	∓0.1	±0.2	±2.0	±1.0	±0.5
30–45	$0.91^{+ 0.06}_{- 0.09}$	1.887e+02	2.3	${}^{+ 4.2}_{-3.4}$	${}^{ +12}_{ -10}$	${}^{+ 6.8}_{-5.7}$	${}^{+ 9.6}_{-7.9}$	${}^{+ 2.3}_{-2.0}$	${}^{+ 0.1}_{-0.1}$	${}^{+ 2.8}_{-2.7}$	${}^{+ 3.4}_{-3.0}$	${}^{+ 5.0}_{-4.5}$	${}^{-0.0}_{-0.0}$	${}^{+ 0.0}_{-0.1}$	${}^{+ 3.6}_{-3.3}$	${}^{-0.2}_{+ 0.2}$	${}^{+ 0.1}_{-0.1}$	±2.6	∓0.1	±0.0	±0.1	±1.0	±1.0	±0.5
45–60	$0.93^{+ 0.06}_{- 0.07}$	3.068e+01	2.1	${}^{+ 1.4}_{-1.5}$	${}^{+ 8.1}_{-7.2}$	${}^{+ 5.9}_{-5.7}$	${}^{+ 5.2}_{-4.6}$	${}^{+ 1.7}_{-1.7}$	${}^{+ 0.1}_{-0.1}$	${}^{+ 3.0}_{-3.0}$	${}^{+ 2.0}_{-1.8}$	${}^{+ 4.7}_{-4.4}$	${}^{+ 0.1}_{-0.1}$	${}^{+ 0.0}_{-0.0}$	${}^{+ 2.7}_{-2.5}$	${}^{+ 0.2}_{-0.3}$	${}^{+ 0.1}_{-0.0}$	±0.6	±0.2	±0.2	±0.1	±1.0	±1.0	±0.5
60–80	$0.95^{+ 0.05}_{- 0.06}$	6.991e+00	0.9	${}^{-0.8}_{+ 0.7}$	${}^{+ 6.2}_{-6.2}$	${}^{+ 4.8}_{-4.9}$	${}^{+ 3.8}_{-4.0}$	${}^{+ 2.5}_{-2.5}$	${}^{+ 0.0}_{-0.0}$	${}^{+ 3.0}_{-3.0}$	${}^{+ 1.1}_{-1.2}$	${}^{+ 4.4}_{-4.6}$	${}^{+ 0.4}_{-0.4}$	${}^{+ 0.0}_{-0.0}$	${}^{+ 0.6}_{-0.7}$	${}^{+ 1.8}_{-2.0}$	${}^{+ 0.0}_{-0.0}$	±1.1	∓0.4	±0.2	±0.0	±1.0	±1.0	±0.5
80–110	$0.97^{+ 0.05}_{- 0.05}$	1.357e+00	1.3	${}^{-0.5}_{+ 0.4}$	${}^{+ 6.5}_{-6.1}$	${}^{+ 4.4}_{-4.2}$	${}^{+ 3.5}_{-3.4}$	${}^{+ 0.9}_{-0.9}$	${}^{+ 0.0}_{-0.0}$	${}^{+ 3.0}_{-3.0}$	${}^{+ 1.1}_{-1.2}$	${}^{+ 5.3}_{-5.0}$	${}^{+ 1.1}_{-1.2}$	${}^{+ 0.0}_{-0.0}$	${}^{-0.3}_{+ 0.3}$	${}^{+ 4.3}_{-4.2}$	${}^{+ 0.0}_{-0.0}$	±1.1	±0.1	±0.2	±0.0	±1.0	±1.0	±0.5
110–160	$0.98^{+ 0.04}_{- 0.04}$	1.860e-01	2.6	${}^{-0.1}_{+ 0.0}$	${}^{+ 6.4}_{-6.1}$	${}^{+ 4.2}_{-3.9}$	${}^{+ 3.6}_{-3.4}$	${}^{+ 1.0}_{-0.9}$	${}^{+ 0.0}_{-0.0}$	${}^{+ 2.7}_{-2.7}$	${}^{+ 0.8}_{-0.8}$	${}^{+ 5.9}_{-5.8}$	${}^{+ 1.9}_{-1.9}$	${}^{+ 0.1}_{-0.1}$	${}^{-0.1}_{+ 0.1}$	${}^{+ 6.0}_{-5.9}$	${}^{+ 0.0}_{-0.0}$	±0.8	∓0.3	±0.6	±0.0	±1.0	±1.0	±0.5
160–210	$0.99^{+ 0.03}_{- 0.03}$	2.128e-02	7.4	${}^{-0.0}_{+ 0.0}$	${}^{+ 4.9}_{-5.2}$	${}^{+ 3.9}_{-4.4}$	${}^{+ 3.3}_{-3.7}$	${}^{+ 1.8}_{-2.0}$	${}^{+ 0.0}_{-0.0}$	${}^{+ 2.4}_{-2.7}$	${}^{+ 0.6}_{-0.8}$	${}^{+ 6.3}_{-6.6}$	${}^{+ 2.8}_{-3.1}$	${}^{+ 0.1}_{-0.1}$	${}^{-0.0}_{+ 0.0}$	${}^{+ 7.3}_{-7.5}$	${}^{+ 0.0}_{-0.0}$	±0.3	∓0.1	±0.3	±0.0	±1.0	±1.0	±0.5
210–260	$0.99^{+ 0.03}_{- 0.03}$	3.343e-03	18.	${}^{-0.0}_{+ 0.0}$	${}^{+ 5.4}_{-4.9}$	${}^{+ 4.2}_{-3.8}$	${}^{+ 2.7}_{-2.5}$	${}^{+ 2.8}_{-2.6}$	${}^{+ 0.0}_{-0.0}$	${}^{+ 2.6}_{-2.3}$	${}^{+ 0.7}_{-0.6}$	${}^{+ 8.1}_{-6.9}$	${}^{+ 4.1}_{-3.8}$	${}^{+ 0.1}_{-0.2}$	${}^{-0.0}_{+ 0.0}$	${}^{+ 9.8}_{-8.5}$	${}^{+ 0.0}_{+ 0.0}$	±0.7	∓0.2	∓0.1	±0.0	±1.0	±1.0	±0.5
260–310	$0.99^{+ 0.03}_{- 0.02}$	9.730e-04	32.	${}^{-0.0}_{+ 0.0}$	${}^{+ 4.6}_{-4.0}$	${}^{+ 3.0}_{-2.9}$	${}^{+ 1.8}_{-1.6}$	${}^{+ 3.0}_{-2.9}$	${}^{+ 0.1}_{-0.0}$	${}^{+ 1.9}_{-1.6}$	${}^{+ 0.5}_{-0.5}$	${}^{+ 8.5}_{-7.5}$	${}^{+ 4.5}_{-4.2}$	${}^{+ 0.2}_{-0.2}$	${}^{-0.0}_{+ 0.0}$	${}^{+11.}_{-9.6}$	${}^{+ 0.0}_{-0.0}$	±1.1	∓0.1	±0.2	±0.0	±1.0	±1.0	±0.5
310–400	$0.99^{+ 0.03}_{- 0.02}$	3.594e-04	44.	${}^{-0.0}_{+ 0.0}$	${}^{+ 4.8}_{-4.1}$	${}^{+ 3.7}_{-2.9}$	${}^{+ 2.4}_{-1.8}$	${}^{+ 4.2}_{-3.5}$	${}^{+ 0.0}_{-0.0}$	${}^{+ 2.2}_{-1.7}$	${}^{+ 0.7}_{-0.5}$	${}^{+ 9.8}_{-8.6}$	${}^{+ 6.5}_{-5.4}$	${}^{+ 0.3}_{-0.2}$	${}^{-0.0}_{+ 0.0}$	${}^{ +13}_{ -12}$	${}^{+ 0.0}_{-0.0}$	±0.7	±0.0	±0.2	±0.0	±1.0	±1.0	±0.5
400–500	$0.99^{+ 0.03}_{- 0.02}$	8.801e-05	78.	0.0	${}^{+ 5.2}_{-6.0}$	${}^{+ 4.0}_{-5.1}$	${}^{+ 1.9}_{-2.4}$	${}^{+ 3.7}_{-4.6}$	${}^{+ 0.0}_{+ 0.0}$	${}^{+ 1.8}_{-2.4}$	${}^{+ 0.4}_{-0.7}$	${}^{ +11}_{ -11}$	${}^{+ 7.0}_{-7.9}$	${}^{+ 0.2}_{-0.4}$	0.0	${}^{ +16}_{ -15}$	${}^{+ 0.0}_{+ 0.0}$	±0.6	±0.1	±0.5	±0.0	±1.0	±1.0	±0.5

**Table 6:** Measured jet cross section for anti-*k*_*t*_ jets with *R*=0.4 in the rapidity bin 0.8≤|*y*|<1.2. See caption of Table 4 for details

*p*_T_ (GeV)	NPCorr	*σ* (nb/GeV)	$\delta_{\mathrm{stat.}}$ %	*γ*_2_ %	*γ*_8_ %	*γ*_14_ %	*γ*_20_ %	*γ*_26_ %	*γ*_88_ %	*γ*_33_ %	*γ*_39_ %	*γ*_45_ %	*γ*_51_ %	*γ*_57_ %	*γ*_63_ %	*γ*_69_ %	*γ*_89_ %	*γ*_77_ %	*γ*_82_ %	*γ*_74_ %	*γ*_75_ %	*γ*_83_ %	*u*_1_ %	*u*_2_ %
20–30	$0.89^{+ 0.07}_{-0.11}$	1.144e+03	1.6	${}^{+11.}_{-8.7}$	${}^{+ 5.7}_{-8.1}$	${}^{+ 9.8}_{-8.9}$	${}^{ +14}_{ -12}$	${}^{+3.9}_{-3.7}$	${}^{+ 6.2}_{-6.8}$	${}^{+ 1.9}_{-2.0}$	${}^{+4.1}_{-4.0}$	${}^{+ 4.2}_{-4.3}$	${}^{-0.0}_{+ 0.0}$	${}^{+0.0}_{-0.0}$	${}^{+ 2.5}_{-2.6}$	${}^{-0.0}_{+ 0.0}$	${}^{+3.6}_{-3.8}$	±9.5	±1.2	∓0.3	±0.1	±2.0	±1.0	±0.5
30–45	$0.92^{+ 0.06}_{-0.09}$	1.599e+02	2.7	${}^{+ 3.6}_{-3.6}$	${}^{ +12}_{ -10}$	${}^{+ 6.3}_{-6.2}$	${}^{+ 3.5}_{-3.3}$	${}^{+1.9}_{-2.0}$	${}^{+ 7.4}_{-7.1}$	${}^{+ 2.5}_{-2.8}$	${}^{+3.0}_{-3.2}$	${}^{+ 4.5}_{-4.7}$	${}^{-0.0}_{+ 0.0}$	${}^{+0.0}_{-0.0}$	${}^{+ 3.2}_{-3.4}$	${}^{-0.2}_{+ 0.2}$	${}^{+4.1}_{-4.1}$	±5.3	±0.1	∓0.0	±0.7	±1.0	±1.0	±0.5
45–60	$0.94^{+ 0.05}_{-0.07}$	2.690e+01	2.4	${}^{+ 1.6}_{-1.2}$	${}^{+ 8.7}_{-7.1}$	${}^{+ 6.4}_{-5.7}$	${}^{+ 1.4}_{-1.1}$	${}^{+1.6}_{-1.4}$	${}^{+ 6.7}_{-5.7}$	${}^{+ 3.0}_{-2.6}$	${}^{+1.8}_{-1.5}$	${}^{+ 4.8}_{-4.2}$	${}^{+ 0.0}_{-0.1}$	${}^{+0.0}_{-0.0}$	${}^{+ 2.7}_{-2.2}$	${}^{+ 0.1}_{-0.0}$	${}^{+3.7}_{-3.2}$	±3.9	∓0.5	±0.5	±0.1	±1.0	±1.0	±0.5
60–80	$0.96^{+ 0.05}_{-0.06}$	5.937e+00	1.1	${}^{-0.7}_{+ 0.7}$	${}^{+ 7.0}_{-6.1}$	${}^{+ 5.8}_{-4.9}$	${}^{+ 3.4}_{-2.9}$	${}^{+2.9}_{-2.4}$	${}^{+ 5.7}_{-4.9}$	${}^{+ 3.8}_{-3.1}$	${}^{+1.8}_{-1.3}$	${}^{+ 5.4}_{-4.7}$	${}^{+ 0.4}_{-0.5}$	${}^{+0.0}_{-0.1}$	${}^{+ 0.9}_{-0.7}$	${}^{+ 2.3}_{-2.1}$	${}^{+3.6}_{-3.0}$	±1.2	±0.1	±0.5	±0.1	±1.0	±1.0	±0.5
80–110	$0.98^{+ 0.04}_{-0.05}$	1.159e+00	1.6	${}^{-0.5}_{+ 0.4}$	${}^{+ 6.7}_{-5.6}$	${}^{+ 4.3}_{-3.7}$	${}^{+ 2.9}_{-2.6}$	${}^{+0.9}_{-0.9}$	${}^{+ 3.9}_{-3.4}$	${}^{+ 2.9}_{-2.6}$	${}^{+1.2}_{-1.1}$	${}^{+ 5.3}_{-4.6}$	${}^{+ 1.2}_{-1.1}$	${}^{+0.0}_{-0.1}$	${}^{-0.4}_{+ 0.3}$	${}^{+ 4.3}_{-3.8}$	${}^{+2.0}_{-1.6}$	±2.8	±0.3	±0.5	±0.0	±1.0	±1.0	±0.5
110–160	$0.99^{+ 0.04}_{-0.04}$	1.508e-01	3.1	${}^{-0.1}_{+ 0.0}$	${}^{+ 6.8}_{-5.8}$	${}^{+ 4.8}_{-3.8}$	${}^{+ 2.8}_{-2.3}$	${}^{+1.1}_{-1.0}$	${}^{+ 3.9}_{-3.0}$	${}^{+ 3.1}_{-2.5}$	${}^{+1.0}_{-0.9}$	${}^{+ 6.4}_{-5.6}$	${}^{+ 2.2}_{-1.9}$	${}^{+0.1}_{-0.1}$	${}^{-0.2}_{+ 0.1}$	${}^{+ 6.5}_{-5.8}$	${}^{+1.3}_{-1.1}$	±3.0	∓0.0	±1.4	±0.0	±1.0	±1.0	±0.5
160–210	$0.99^{+ 0.03}_{-0.03}$	1.714e-02	9.1	${}^{-0.0}_{+ 0.0}$	${}^{+ 5.4}_{-5.9}$	${}^{+ 4.2}_{-4.8}$	${}^{+ 2.6}_{-3.3}$	${}^{+2.0}_{-1.9}$	${}^{+ 3.8}_{-4.4}$	${}^{+ 2.4}_{-3.2}$	${}^{+0.7}_{-0.7}$	${}^{+ 7.1}_{-7.3}$	${}^{+ 3.0}_{-3.6}$	${}^{+0.1}_{-0.1}$	${}^{-0.0}_{+ 0.0}$	${}^{+ 8.2}_{-8.2}$	${}^{+1.5}_{-1.4}$	±2.5	±0.1	±0.2	±0.0	±1.0	±1.0	±0.5
210–260	$0.99^{+ 0.03}_{-0.03}$	2.282e-03	22.	${}^{-0.0}_{+ 0.0}$	${}^{+ 5.0}_{-4.8}$	${}^{+ 4.1}_{-3.7}$	${}^{+ 2.6}_{-2.2}$	${}^{+2.6}_{-2.4}$	${}^{+ 4.4}_{-4.1}$	${}^{+ 2.3}_{-2.1}$	${}^{+0.6}_{-0.5}$	${}^{+ 7.9}_{-7.5}$	${}^{+ 4.1}_{-3.7}$	${}^{+0.2}_{-0.1}$	${}^{-0.0}_{+ 0.0}$	${}^{+ 9.6}_{-9.5}$	${}^{+1.6}_{-1.6}$	±0.3	±0.0	∓0.4	±0.0	±1.0	±1.0	±0.5
260–310	$0.99^{+ 0.03}_{-0.03}$	6.512e-04	42.	${}^{-0.0}_{+ 0.0}$	${}^{+ 5.3}_{-4.8}$	${}^{+ 3.4}_{-3.1}$	${}^{+ 1.3}_{-1.3}$	${}^{+3.6}_{-3.3}$	${}^{+ 5.2}_{-4.8}$	${}^{+ 2.0}_{-1.9}$	${}^{+0.7}_{-0.4}$	${}^{+ 9.6}_{-9.2}$	${}^{+ 5.5}_{-5.2}$	${}^{+0.2}_{-0.2}$	${}^{-0.0}_{+ 0.0}$	${}^{ +12}_{ -12}$	${}^{+ 1.7}_{-1.7}$	±2.9	±0.1	±0.6	±0.0	±1.0	±1.0	±0.5
310–400	$0.99^{+ 0.03}_{-0.02}$	5.245e-05	124	${}^{-0.5}_{+ 0.4}$	${}^{+ 5.6}_{-5.5}$	${}^{+ 4.2}_{-3.9}$	${}^{+ 1.3}_{-1.5}$	${}^{+4.9}_{-4.6}$	${}^{+ 6.6}_{-6.2}$	${}^{+ 2.4}_{-2.5}$	${}^{+0.5}_{-0.8}$	${}^{ +11}_{ -11}$	${}^{+ 7.8}_{-7.2}$	${}^{+ 0.2}_{-0.2}$	${}^{-0.1}_{+ 0.1}$	${}^{ +16}_{ -14}$	${}^{+ 2.2}_{-2.3}$	±2.1	±0.2	±2.1	±0.0	±1.0	±1.0	±0.5

**Table 7:** Measured jet cross section for anti-*k*_*t*_ jets with *R*=0.4 in the rapidity bin 1.2≤|*y*|<2.1. See caption of Table 4 for details

*p*_T_ (GeV)	NPCorr	*σ* (nb/GeV)	$\delta_{\mathrm{stat.}}$ %	*γ*_3_ %	*γ*_9_ %	*γ*_15_ %	*γ*_21_ %	*γ*_27_ %	*γ*_88_ %	*γ*_34_ %	*γ*_40_ %	*γ*_46_ %	*γ*_52_ %	*γ*_58_ %	*γ*_64_ %	*γ*_70_ %	*γ*_89_ %	*γ*_78_ %	*γ*_82_ %	*γ*_74_ %	*γ*_75_ %	*γ*_83_ %	*u*_1_ %	*u*_2_ %
20–30	$0.89^{+ 0.08}_{- 0.11}$	9.462e+02	1.2	${}^{+13.}_{-9.1}$	${}^{+ 6.5}_{-8.3}$	${}^{+12.}_{-9.3}$	${}^{+13.}_{-8.5}$	${}^{+ 4.4}_{-3.9}$	${}^{ +15}_{ -14}$	${}^{+ 2.1}_{-2.2}$	${}^{+ 4.5}_{-4.1}$	${}^{+ 4.5}_{-4.5}$	${}^{-0.0}_{+ 0.0}$	${}^{+ 0.0}_{-0.0}$	${}^{+ 2.6}_{-2.9}$	${}^{-0.0}_{+ 0.0}$	${}^{+ 5.0}_{-5.2}$	±12.	∓0.6	∓0.1	±0.6	±2.0	±1.0	±0.5
30–45	$0.92^{+ 0.06}_{- 0.08}$	1.270e+02	2.1	${}^{+ 4.3}_{-3.8}$	${}^{ +13}_{ -11}$	${}^{+ 7.0}_{-6.5}$	${}^{+ 1.0}_{-0.6}$	${}^{+ 2.3}_{-2.0}$	${}^{ +17}_{ -14}$	${}^{+ 2.9}_{-2.8}$	${}^{+ 3.4}_{-3.3}$	${}^{+ 5.1}_{-4.9}$	${}^{-0.0}_{+ 0.0}$	${}^{+ 0.0}_{-0.0}$	${}^{+ 3.6}_{-3.6}$	${}^{-0.2}_{+ 0.1}$	${}^{+ 6.0}_{-5.7}$	±5.5	∓0.4	±0.1	±0.1	±1.0	±1.0	±0.5
45–60	$0.94^{+ 0.05}_{- 0.06}$	2.007e+01	1.9	${}^{+ 1.8}_{-1.4}$	${}^{+ 8.7}_{-7.2}$	${}^{+ 6.5}_{-5.7}$	${}^{-0.4}_{+ 0.3}$	${}^{+ 2.0}_{-1.6}$	${}^{+11.}_{-8.8}$	${}^{+ 3.5}_{-2.9}$	${}^{+ 2.3}_{-1.7}$	${}^{+ 5.0}_{-4.2}$	${}^{+ 0.2}_{-0.1}$	${}^{+ 0.0}_{-0.0}$	${}^{+ 3.0}_{-2.3}$	${}^{+ 0.3}_{-0.2}$	${}^{+ 5.5}_{-4.5}$	±1.7	∓0.1	±0.2	±0.1	±1.0	±1.0	±0.5
60–80	$0.96^{+ 0.04}_{- 0.05}$	4.185e+00	0.9	${}^{-0.8}_{+ 0.7}$	${}^{+ 6.6}_{-6.5}$	${}^{+ 5.0}_{-5.0}$	${}^{+ 0.5}_{-0.5}$	${}^{+ 2.5}_{-2.4}$	${}^{+ 6.2}_{-6.0}$	${}^{+ 2.9}_{-3.0}$	${}^{+ 1.3}_{-1.3}$	${}^{+ 4.6}_{-4.8}$	${}^{+ 0.5}_{-0.5}$	${}^{+ 0.0}_{-0.0}$	${}^{+ 0.7}_{-0.7}$	${}^{+ 1.8}_{-2.0}$	${}^{+ 4.2}_{-4.2}$	±1.3	∓0.1	±0.3	±0.0	±1.0	±1.0	±0.5
80–110	$0.97^{+ 0.04}_{- 0.04}$	7.174e-01	1.3	${}^{-0.5}_{+ 0.4}$	${}^{+ 7.2}_{-6.7}$	${}^{+ 4.7}_{-4.6}$	${}^{+ 1.2}_{-1.2}$	${}^{+ 1.0}_{-1.0}$	${}^{+ 5.2}_{-4.8}$	${}^{+ 3.5}_{-3.3}$	${}^{+ 1.4}_{-1.3}$	${}^{+ 5.9}_{-5.5}$	${}^{+ 1.4}_{-1.4}$	${}^{+ 0.0}_{-0.1}$	${}^{-0.4}_{+ 0.3}$	${}^{+ 4.9}_{-4.7}$	${}^{+ 4.1}_{-3.8}$	±1.8	∓0.3	±0.3	±0.0	±1.0	±1.0	±0.5
110–160	$0.98^{+ 0.04}_{- 0.03}$	8.204e-02	2.8	${}^{-0.1}_{+ 0.0}$	${}^{+ 7.5}_{-6.6}$	${}^{+ 4.8}_{-4.5}$	${}^{+ 1.2}_{-1.1}$	${}^{+ 1.2}_{-1.1}$	${}^{+ 4.8}_{-4.6}$	${}^{+ 3.0}_{-2.9}$	${}^{+ 1.1}_{-0.9}$	${}^{+ 7.1}_{-6.4}$	${}^{+ 2.2}_{-2.2}$	${}^{+ 0.2}_{-0.1}$	${}^{-0.1}_{+ 0.1}$	${}^{+ 7.2}_{-6.5}$	${}^{+ 3.1}_{-3.0}$	±2.3	∓0.1	±0.4	±0.0	±1.0	±1.0	±0.5
160–210	$0.99^{+ 0.04}_{- 0.03}$	9.146e-03	8.4	${}^{-0.0}_{+ 0.0}$	${}^{+ 6.5}_{-6.3}$	${}^{+ 5.2}_{-5.1}$	${}^{+ 1.0}_{-1.0}$	${}^{+ 2.3}_{-2.5}$	${}^{+ 6.2}_{-6.1}$	${}^{+ 2.9}_{-3.1}$	${}^{+ 0.6}_{-0.7}$	${}^{+ 8.6}_{-8.3}$	${}^{+ 3.5}_{-3.7}$	${}^{+ 0.1}_{-0.1}$	${}^{-0.0}_{+ 0.0}$	${}^{+10.}_{-9.6}$	${}^{+ 3.8}_{-4.0}$	±2.3	∓0.2	±1.1	±0.0	±1.0	±1.0	±0.5
210–260	$0.99^{+ 0.04}_{- 0.03}$	9.061e-04	24.	${}^{-0.0}_{+ 0.0}$	${}^{+ 6.3}_{-6.2}$	${}^{+ 5.0}_{-4.8}$	${}^{+ 2.2}_{-2.1}$	${}^{+ 3.3}_{-3.3}$	${}^{+ 7.2}_{-6.9}$	${}^{+ 2.9}_{-2.7}$	${}^{+ 0.8}_{-0.8}$	${}^{+ 9.9}_{-9.5}$	${}^{+ 5.2}_{-5.1}$	${}^{+ 0.1}_{-0.2}$	${}^{-0.0}_{+ 0.0}$	${}^{ +12}_{ -12}$	${}^{+ 4.8}_{-4.7}$	±3.4	∓0.1	±1.2	±0.0	±1.0	±1.0	±0.5
260–310	$0.99^{+ 0.04}_{- 0.03}$	8.945e-05	77.	${}^{-0.0}_{+ 0.0}$	${}^{+ 7.1}_{-5.5}$	${}^{+ 5.1}_{-4.0}$	${}^{+ 2.9}_{-2.7}$	${}^{+ 5.3}_{-4.2}$	${}^{+ 9.3}_{-7.6}$	${}^{+ 3.1}_{-2.7}$	${}^{+ 0.9}_{-0.9}$	${}^{ +14}_{ -12}$	${}^{+ 7.5}_{-6.0}$	${}^{+ 0.3}_{-0.3}$	${}^{-0.0}_{+ 0.0}$	${}^{ +17}_{ -14}$	${}^{+ 6.2}_{-4.9}$	±0.8	∓0.1	±0.7	±0.0	±1.0	±1.0	±0.5
310–400	$0.99^{+ 0.04}_{- 0.03}$	2.379e-05	122	${}^{-0.1}_{+ 0.1}$	${}^{+ 6.9}_{-7.5}$	${}^{+ 5.6}_{-5.6}$	${}^{+ 2.4}_{-3.2}$	${}^{+ 6.2}_{-6.3}$	${}^{ +10}_{ -11}$	${}^{+ 3.0}_{-3.6}$	${}^{+ 0.8}_{-1.0}$	${}^{ +14}_{ -14}$	${}^{+ 9.0}_{-9.2}$	${}^{+ 0.2}_{-0.6}$	${}^{-0.0}_{+ 0.0}$	${}^{ +19}_{ -18}$	${}^{+ 7.0}_{-7.3}$	±3.4	±0.0	±0.2	±0.0	±1.0	±1.0	±0.5

**Table 8:** Measured jet cross section for anti-*k*_*t*_ jets with *R*=0.4 in the rapidity bin 2.1≤|*y*|<2.8. See caption of Table 4 for details

*p*_T_ (GeV)	NPCorr	*σ* (nb/GeV)	$\delta_{\mathrm{stat.}}$ %	*γ*_4_ %	*γ*_10_ %	*γ*_16_ %	*γ*_22_ %	*γ*_28_ %	*γ*_88_ %	*γ*_35_ %	*γ*_41_ %	*γ*_47_ %	*γ*_53_ %	*γ*_59_ %	*γ*_65_ %	*γ*_71_ %	*γ*_89_ %	*γ*_79_ %	*γ*_82_ %	*γ*_74_ %	*γ*_75_ %	*γ*_84_ %	*u*_1_ %	*u*_2_ %
20–30	$0.88^{+ 0.09}_{- 0.11}$	6.251e+02	1.9	${}^{+14.}_{-9.3}$	${}^{+ 6.7}_{-8.1}$	${}^{+13.}_{-9.3}$	${}^{+ 8.1}_{-6.3}$	${}^{+ 4.9}_{-3.9}$	${}^{ +29}_{ -24}$	${}^{+ 2.1}_{-2.1}$	${}^{+ 5.0}_{-4.1}$	${}^{+ 4.9}_{-4.4}$	${}^{-0.0}_{+ 0.0}$	${}^{+ 0.0}_{-0.0}$	${}^{+ 2.8}_{-2.7}$	${}^{-0.0}_{+ 0.0}$	${}^{+ 4.2}_{-3.8}$	±18.	∓0.4	∓0.0	±0.0	±2.0	±1.0	±0.5
30–45	$0.92^{+ 0.07}_{- 0.08}$	7.790e+01	1.5	${}^{+ 4.2}_{-3.5}$	${}^{ +13}_{ -10}$	${}^{+ 7.0}_{-6.2}$	${}^{+ 4.6}_{-3.9}$	${}^{+ 2.2}_{-1.8}$	${}^{ +30}_{ -22}$	${}^{+ 3.0}_{-2.5}$	${}^{+ 3.6}_{-2.9}$	${}^{+ 5.0}_{-4.3}$	${}^{-0.0}_{+ 0.0}$	${}^{+ 0.0}_{-0.0}$	${}^{+ 3.7}_{-3.2}$	${}^{-0.2}_{+ 0.1}$	${}^{+ 4.5}_{-3.9}$	±5.1	∓0.8	±0.3	±0.1	±1.0	±1.0	±0.5
45–60	$0.94^{+ 0.06}_{- 0.06}$	1.031e+01	2.6	${}^{+ 2.2}_{-2.0}$	${}^{+ 9.3}_{-8.0}$	${}^{+ 7.3}_{-6.4}$	${}^{+ 2.5}_{-2.3}$	${}^{+ 2.1}_{-1.9}$	${}^{ +18}_{ -14}$	${}^{+ 3.4}_{-3.2}$	${}^{+ 2.3}_{-2.0}$	${}^{+ 5.5}_{-4.8}$	${}^{+ 0.1}_{-0.2}$	${}^{+ 0.0}_{-0.0}$	${}^{+ 3.0}_{-2.7}$	${}^{+ 0.5}_{-0.3}$	${}^{+ 3.8}_{-3.6}$	±0.3	∓0.1	±0.4	±0.0	±1.0	±1.0	±0.5
60–80	$0.95^{+ 0.05}_{- 0.05}$	1.804e+00	1.3	${}^{-0.8}_{+ 0.7}$	${}^{+ 8.3}_{-7.6}$	${}^{+ 6.2}_{-6.0}$	${}^{+ 1.6}_{-1.4}$	${}^{+ 3.2}_{-3.0}$	${}^{+11.}_{-9.9}$	${}^{+ 3.8}_{-3.6}$	${}^{+ 2.0}_{-1.8}$	${}^{+ 6.0}_{-5.8}$	${}^{+ 0.7}_{-0.7}$	${}^{+ 0.0}_{-0.0}$	${}^{+ 0.9}_{-0.9}$	${}^{+ 2.6}_{-2.7}$	${}^{+ 3.4}_{-3.3}$	±2.6	±0.2	±0.4	±0.0	±1.0	±1.0	±0.5
80–110	$0.96^{+ 0.05}_{- 0.04}$	2.169e-01	2.7	${}^{-0.4}_{+ 0.3}$	${}^{+10.}_{-9.2}$	${}^{+ 7.4}_{-6.1}$	${}^{+ 1.2}_{-1.2}$	${}^{+ 1.4}_{-1.5}$	${}^{+10.}_{-9.1}$	${}^{+ 5.2}_{-4.4}$	${}^{+ 1.7}_{-1.8}$	${}^{+ 8.7}_{-7.3}$	${}^{+ 2.0}_{-2.0}$	${}^{+ 0.1}_{-0.1}$	${}^{-0.4}_{+ 0.4}$	${}^{+ 7.5}_{-6.3}$	${}^{+ 2.8}_{-2.8}$	±2.7	∓0.8	±0.5	±0.0	±1.0	±1.0	±0.5
110–160	$0.96^{+ 0.05}_{- 0.03}$	1.123e-02	8.6	${}^{-0.0}_{+ 0.0}$	${}^{ +11}_{ -10}$	${}^{+ 7.1}_{-6.9}$	${}^{+ 1.9}_{-1.7}$	${}^{+ 1.9}_{-1.7}$	${}^{ +11}_{ -10}$	${}^{+ 4.9}_{-3.9}$	${}^{+ 1.4}_{-1.3}$	${}^{ +10}_{ -10}$	${}^{+ 3.0}_{-3.0}$	${}^{+ 0.1}_{-0.0}$	${}^{-0.1}_{+ 0.1}$	${}^{ +10}_{ -10}$	${}^{+ 1.7}_{-1.5}$	±1.4	±0.0	±0.8	±0.0	±1.0	±1.0	±0.5
160–210	$0.96^{+ 0.07}_{- 0.03}$	5.265e-04	39.	${}^{-0.0}_{+ 0.0}$	${}^{ +11}_{ -11}$	${}^{+7.6}_{-10.}$	${}^{+ 2.1}_{-2.3}$	${}^{+ 3.3}_{-4.2}$	${}^{ +16}_{ -17}$	${}^{+ 5.0}_{-4.6}$	${}^{+ 1.0}_{-1.1}$	${}^{ +14}_{ -17}$	${}^{+ 6.6}_{-8.1}$	${}^{+ 0.1}_{-0.2}$	${}^{-0.0}_{+ 0.0}$	${}^{ +17}_{ -19}$	${}^{+ 2.2}_{-2.7}$	±1.0	±0.9	±1.8	±0.0	±1.0	±1.0	±0.5
210–260	$0.96^{+ 0.07}_{- 0.03}$	6.264e-05	113	${}^{-0.0}_{+ 0.0}$	${}^{+12.}_{-9.2}$	${}^{+ 7.8}_{-6.2}$	${}^{-0.8}_{-1.8}$	${}^{+ 4.3}_{-5.4}$	${}^{ +23}_{ -24}$	${}^{+ 0.7}_{-3.5}$	${}^{-0.5}_{-2.1}$	${}^{ +17}_{ -24}$	${}^{+ 8.9}_{-8.8}$	${}^{-0.0}_{-1.2}$	${}^{-0.0}_{+ 0.0}$	${}^{ +34}_{ -29}$	${}^{+ 0.4}_{-3.0}$	±6.2	±3.1	±11.4	±0.0	±1.0	±1.0	±0.5

**Table 9:** Measured jet cross section for anti-*k*_*t*_ jets with *R*=0.4 in the rapidity bin 2.8≤|*y*|<3.6. See caption of Table 4 for details

*p*_T_ (GeV)	NPCorr	*σ* (nb/GeV)	$\delta_{\mathrm{stat.}}$ %	*γ*_5_ %	*γ*_11_ %	*γ*_17_ %	*γ*_23_ %	*γ*_29_ %	*γ*_88_ %	*γ*_36_ %	*γ*_42_ %	*γ*_48_ %	*γ*_54_ %	*γ*_60_ %	*γ*_66_ %	*γ*_72_ %	*γ*_89_ %	*γ*_80_ %	*γ*_82_ %	*γ*_74_ %	*γ*_75_ %	*γ*_85_ %	*u*_1_ %	*u*_2_ %
20–30	$0.86^{+ 0.11}_{- 0.09}$	3.133e+02	2.3	${}^{+15.}_{-10.}$	${}^{+ 7.4}_{-9.4}$	${}^{ +13}_{ -10}$	${}^{+11.}_{-8.3}$	${}^{+ 5.0}_{-4.4}$	${}^{ +43}_{ -33}$	${}^{+ 2.6}_{-2.5}$	${}^{+ 5.2}_{-4.6}$	${}^{+ 5.3}_{-5.1}$	${}^{-0.0}_{+ 0.0}$	${}^{+ 0.0}_{-0.0}$	${}^{+ 3.2}_{-3.2}$	${}^{-0.0}_{+ 0.0}$	${}^{+ 4.9}_{-4.9}$	±19.	±0.1	∓0.1	±0.4	±2.0	±2.0	±0.5
30–45	$0.89^{+ 0.09}_{- 0.06}$	2.821e+01	2.3	${}^{+ 4.2}_{-4.0}$	${}^{ +15}_{ -13}$	${}^{+ 8.0}_{-7.9}$	${}^{+ 6.2}_{-5.8}$	${}^{+ 2.4}_{-2.4}$	${}^{ +51}_{ -34}$	${}^{+ 3.5}_{-3.3}$	${}^{+ 3.9}_{-3.7}$	${}^{+ 6.4}_{-5.6}$	${}^{+ 0.1}_{+ 0.0}$	${}^{+ 0.0}_{+ 0.0}$	${}^{+ 4.2}_{-4.0}$	${}^{-0.1}_{+ 0.1}$	${}^{+ 5.9}_{-5.5}$	±5.8	±0.3	∓0.5	±0.5	±1.0	±2.0	±0.5
45–60	$0.91^{+ 0.08}_{- 0.05}$	2.254e+00	2.3	${}^{+ 2.1}_{-1.6}$	${}^{ +13}_{ -12}$	${}^{+10.}_{-9.8}$	${}^{+ 6.3}_{-5.2}$	${}^{+ 3.2}_{-2.4}$	${}^{ +41}_{ -27}$	${}^{+ 5.2}_{-4.6}$	${}^{+ 3.2}_{-2.3}$	${}^{+ 8.1}_{-7.3}$	${}^{+ 0.2}_{-0.2}$	${}^{+ 0.0}_{-0.0}$	${}^{+ 4.1}_{-3.1}$	${}^{+ 1.0}_{-0.8}$	${}^{+ 6.6}_{-6.1}$	±4.0	∓0.7	±2.2	±0.0	±1.0	±1.0	±0.5
60–80	$0.91^{+ 0.08}_{- 0.04}$	1.774e-01	3.9	${}^{-1.6}_{+ 1.5}$	${}^{ +12}_{ -13}$	${}^{+8.6}_{-10.}$	${}^{+ 7.2}_{-7.4}$	${}^{+ 3.9}_{-4.4}$	${}^{ +27}_{ -23}$	${}^{+ 5.9}_{-6.4}$	${}^{+ 2.8}_{-2.2}$	${}^{+8.8}_{-10.}$	${}^{+ 1.8}_{-1.1}$	${}^{+ 0.1}_{-0.1}$	${}^{+ 1.0}_{-0.2}$	${}^{+ 4.7}_{-5.4}$	${}^{+ 5.8}_{-6.6}$	±8.3	∓0.9	±4.4	±0.0	±1.0	±1.0	±0.5
80–110	$0.91^{+ 0.08}_{- 0.04}$	4.533e-03	15.	${}^{-0.9}_{+ 0.8}$	${}^{ +11}_{ -16}$	${}^{+5.9}_{-12.}$	${}^{+6.4}_{-12.}$	${}^{+ 1.3}_{-3.1}$	${}^{ +18}_{ -23}$	${}^{+5.1}_{-10.}$	${}^{+ 1.7}_{-3.7}$	${}^{+7.9}_{-15.}$	${}^{+ 2.7}_{-4.4}$	${}^{+ 0.1}_{+ 0.0}$	${}^{-1.1}_{+ 0.9}$	${}^{+8.2}_{-14.}$	${}^{+ 4.3}_{-6.8}$	±2.6	±1.8	∓2.5	±0.0	±1.0	±1.0	±0.5
110–160	$0.91^{+ 0.08}_{- 0.03}$	1.507e-04	69.	${}^{-0.2}_{+ 0.1}$	${}^{+1.9}_{-28.}$	${}^{+0.11}_{-14.}$	${}^{+0.60}_{-15.}$	${}^{-1.2}_{-4.7}$	${}^{ +29}_{ -41}$	${}^{+ 1.3}_{-6.9}$	${}^{-1.0}_{-5.1}$	${}^{+0.25}_{-29.}$	${}^{-0.5}_{-5.6}$	${}^{-0.0}_{+ 0.0}$	${}^{-0.5}_{+ 0.3}$	${}^{+1.7}_{-28.}$	${}^{-2.0}_{-3.8}$	±30.	∓0.2	±26.5	±0.0	±1.0	±1.0	±0.5

**Table 10:** Measured jet cross section for anti-*k*_*t*_ jets with *R*=0.4 in the rapidity bin 3.6≤|*y*|<4.4. See caption of Table 4 for details

*p*_T_ (GeV)	NPCorr	*σ* (nb/GeV)	$\delta_{\mathrm{stat.}}$ %	*γ*_6_ %	*γ*_12_ %	*γ*_18_ %	*γ*_24_ %	*γ*_30_ %	*γ*_88_ %	*γ*_37_ %	*γ*_43_ %	*γ*_49_ %	*γ*_55_ %	*γ*_61_ %	*γ*_67_ %	*γ*_73_ %	*γ*_89_ %	*γ*_81_ %	*γ*_82_ %	*γ*_74_ %	*γ*_75_ %	*γ*_86_ %	*u*_1_ %	*u*_2_ %
20–30	$0.78^{+ 0.14}_{- 0.08}$	4.651e+01	5.8	${}^{ +23}_{ -14}$	${}^{ +12}_{ -14}$	${}^{ +21}_{ -15}$	${}^{+ 5.4}_{-5.0}$	${}^{+ 7.4}_{-6.6}$	${}^{+116}_{ -62}$	${}^{+ 3.5}_{-4.1}$	${}^{+ 7.6}_{-7.0}$	${}^{+ 7.9}_{-8.2}$	${}^{ 0.0}_{+ 0.0}$	${}^{+ 0.0}_{-0.0}$	${}^{+ 4.6}_{-5.1}$	${}^{-0.0}_{+ 0.0}$	${}^{ +21}_{ -18}$	±31.	±0.0	∓0.3	±1.5	±2.0	±2.0	±0.5
30–45	$0.78^{+ 0.14}_{- 0.09}$	1.330e+00	6.4	${}^{+7.2}_{-13.}$	${}^{ +28}_{ -28}$	${}^{ +16}_{ -19}$	${}^{+ 3.7}_{-4.2}$	${}^{+ 5.5}_{-5.6}$	${}^{+218}_{ -68}$	${}^{+ 7.1}_{-8.0}$	${}^{+6.9}_{-13.}$	${}^{ +11}_{ -16}$	${}^{-0.0}_{-0.0}$	${}^{-0.0}_{-0.0}$	${}^{+7.6}_{-13.}$	${}^{+ 0.0}_{-0.0}$	${}^{ +32}_{ -30}$	±17.	±1.9	±3.0	±1.9	±1.0	±1.0	±0.5
45–60	$0.78^{+ 0.14}_{- 0.10}$	9.127e-03	18.	${}^{+ 0.9}_{-5.5}$	${}^{ +28}_{ -22}$	${}^{ +17}_{ -17}$	${}^{+ 2.4}_{-5.2}$	${}^{+ 3.7}_{-7.6}$	${}^{+196}_{ -65}$	${}^{+7.5}_{-10.}$	${}^{+ 4.6}_{-7.7}$	${}^{ +15}_{ -15}$	${}^{-0.0}_{-0.9}$	${}^{-0.0}_{+ 0.0}$	${}^{+ 5.4}_{-8.3}$	${}^{+ 2.0}_{-2.7}$	${}^{ +65}_{ -37}$	±17.	±1.8	±15.1	±0.2	±1.0	±1.0	±0.5

**Table 11:** Measured jet cross section for anti-*k*_*t*_ jets with *R*=0.6 in the rapidity bin |*y*|<0.3. See caption of Table 4 for details

*p*_T_ (GeV)	NPCorr	*σ* (nb/GeV)	$\delta_{\mathrm{stat.}}$ %	*γ*_1_ %	*γ*_7_ %	*γ*_13_ %	*γ*_19_ %	*γ*_25_ %	*γ*_88_ %	*γ*_32_ %	*γ*_38_ %	*γ*_44_ %	*γ*_50_ %	*γ*_56_ %	*γ*_62_ %	*γ*_68_ %	*γ*_89_ %	*γ*_76_ %	*γ*_82_ %	*γ*_74_ %	*γ*_75_ %	*γ*_83_ %	*u*_1_ %	*u*_2_ %
20–30	$1.24^{+ 0.06}_{- 0.13}$	2.542e+03	1.4	${}^{ +14}_{ -12}$	${}^{ +11}_{ -11}$	${}^{ +20}_{ -15}$	${}^{+10.}_{-9.3}$	${}^{+ 7.3}_{-5.3}$	0.0	${}^{+ 2.1}_{-2.3}$	${}^{+ 6.1}_{-5.3}$	${}^{+ 5.1}_{-4.8}$	${}^{-0.0}_{+ 0.0}$	${}^{+ 0.0}_{-0.0}$	${}^{+ 1.7}_{-2.3}$	${}^{-0.0}_{+ 0.0}$	0.0	±5.3	∓0.3	∓0.1	±0.5	±2.0	±1.0	±0.5
30–45	$1.12^{+ 0.07}_{- 0.07}$	3.004e+02	2.4	${}^{+ 8.4}_{-7.2}$	${}^{+12.}_{-9.9}$	${}^{+11.}_{-8.6}$	${}^{+ 8.7}_{-7.5}$	${}^{+ 1.7}_{-1.9}$	0.0	${}^{+ 2.6}_{-2.8}$	${}^{+ 3.9}_{-3.9}$	${}^{+ 5.0}_{-4.7}$	${}^{-0.0}_{-0.0}$	${}^{-0.0}_{-0.1}$	${}^{+ 4.0}_{-4.1}$	${}^{-0.0}_{+ 0.0}$	0.0	±3.6	∓0.1	±0.0	±0.1	±1.0	±1.0	±0.5
45–60	$1.06^{+ 0.05}_{- 0.04}$	4.742e+01	2.2	${}^{+ 1.3}_{-1.3}$	${}^{+ 7.7}_{-6.8}$	${}^{+ 4.1}_{-3.6}$	${}^{+ 3.9}_{-3.4}$	${}^{+ 0.8}_{-1.0}$	0.0	${}^{+ 3.0}_{-2.7}$	${}^{+ 2.0}_{-2.0}$	${}^{+ 4.3}_{-3.9}$	${}^{+ 0.1}_{-0.1}$	${}^{+ 0.0}_{-0.0}$	${}^{+ 4.9}_{-4.5}$	${}^{-0.0}_{+ 0.0}$	0.0	±2.7	∓0.0	±0.1	±0.1	±1.0	±1.0	±0.5
60–80	$1.03^{+ 0.04}_{- 0.02}$	1.008e+01	1.0	${}^{-1.2}_{+ 1.1}$	${}^{+ 7.0}_{-7.4}$	${}^{+ 1.4}_{-1.2}$	${}^{+ 4.1}_{-4.1}$	${}^{+ 1.1}_{-1.1}$	0.0	${}^{+ 3.5}_{-3.5}$	${}^{+ 1.7}_{-1.7}$	${}^{+ 5.0}_{-5.1}$	${}^{+ 0.5}_{-0.6}$	${}^{+ 0.1}_{-0.0}$	${}^{+ 5.0}_{-4.7}$	${}^{-0.1}_{+ 0.1}$	0.0	∓0.3	∓0.1	±0.1	±0.0	±1.0	±1.0	±0.5
80–110	$1.02^{+ 0.02}_{- 0.02}$	1.836e+00	1.5	${}^{-0.5}_{+ 0.4}$	${}^{+ 7.3}_{-6.5}$	${}^{+ 1.5}_{-1.5}$	${}^{+ 4.8}_{-4.4}$	${}^{+ 1.3}_{-1.1}$	0.0	${}^{+ 3.7}_{-3.6}$	${}^{+ 1.7}_{-1.5}$	${}^{+ 5.6}_{-5.3}$	${}^{+ 1.2}_{-0.9}$	${}^{+ 0.1}_{-0.1}$	${}^{+ 2.6}_{-2.4}$	${}^{+ 0.0}_{+ 0.1}$	0.0	±1.6	∓0.0	±0.1	±0.0	±1.0	±1.0	±0.5
110–160	$1.02^{+ 0.01}_{- 0.02}$	2.553e-01	2.8	${}^{-0.1}_{+ 0.0}$	${}^{+ 4.9}_{-4.8}$	${}^{+ 2.8}_{-3.2}$	${}^{+ 3.1}_{-3.4}$	${}^{+ 1.0}_{-1.2}$	0.0	${}^{+ 3.0}_{-3.2}$	${}^{+ 0.8}_{-1.0}$	${}^{+ 5.9}_{-5.7}$	${}^{+ 1.5}_{-1.9}$	${}^{+ 0.1}_{-0.2}$	${}^{-0.3}_{+ 0.2}$	${}^{+ 1.2}_{-1.8}$	0.0	±0.4	∓0.0	±0.2	±0.0	±1.0	±1.0	±0.5
160–210	$1.02^{+ 0.01}_{- 0.02}$	3.188e-02	7.9	${}^{-0.0}_{+ 0.0}$	${}^{+ 6.9}_{-6.7}$	${}^{+ 4.9}_{-4.9}$	${}^{+ 2.0}_{-2.1}$	${}^{+ 1.4}_{-1.4}$	0.0	${}^{+ 3.0}_{-3.0}$	${}^{+ 0.9}_{-0.8}$	${}^{+ 7.0}_{-6.6}$	${}^{+ 2.8}_{-2.9}$	${}^{+ 0.3}_{-0.4}$	${}^{-0.3}_{+ 0.3}$	${}^{+ 5.4}_{-5.6}$	0.0	±0.9	±0.1	±0.4	±0.0	±1.0	±1.0	±0.5
210–260	$1.02^{+ 0.01}_{- 0.02}$	5.418e-03	17.	${}^{-0.0}_{+ 0.0}$	${}^{+ 8.5}_{-7.4}$	${}^{+ 4.5}_{-3.9}$	${}^{+ 1.6}_{-1.5}$	${}^{+ 2.0}_{-1.9}$	0.0	${}^{+ 3.1}_{-2.8}$	${}^{+ 0.6}_{-0.7}$	${}^{+ 7.5}_{-6.7}$	${}^{+ 4.0}_{-3.6}$	${}^{+ 0.2}_{-0.3}$	${}^{-0.1}_{+ 0.1}$	${}^{+ 7.7}_{-7.0}$	0.0	±1.0	±0.2	±0.4	±0.0	±1.0	±1.0	±0.5
260–310	$1.02^{+ 0.01}_{- 0.02}$	1.592e-03	34.	${}^{-0.0}_{+ 0.0}$	${}^{+ 4.9}_{-4.4}$	${}^{+ 2.8}_{-2.6}$	${}^{+ 1.4}_{-1.4}$	${}^{+ 2.6}_{-2.7}$	0.0	${}^{+ 2.6}_{-2.5}$	${}^{+ 0.8}_{-0.7}$	${}^{+ 7.3}_{-7.0}$	${}^{+ 4.6}_{-4.3}$	${}^{+ 0.5}_{-0.5}$	${}^{-0.0}_{+ 0.0}$	${}^{+ 8.2}_{-7.9}$	0.0	±0.7	±0.0	±0.4	±0.0	±1.0	±1.0	±0.5
310–400	$1.02^{+ 0.01}_{- 0.02}$	4.135e-04	47.	${}^{-0.0}_{+ 0.0}$	${}^{+ 3.4}_{-3.5}$	${}^{+ 3.7}_{-3.7}$	${}^{+ 0.7}_{-0.7}$	${}^{+ 3.7}_{-3.6}$	0.0	${}^{+ 2.3}_{-2.5}$	${}^{+ 0.6}_{-0.5}$	${}^{+ 8.8}_{-7.9}$	${}^{+ 5.9}_{-5.8}$	${}^{+ 0.6}_{-0.5}$	${}^{-0.0}_{+ 0.0}$	${}^{+10.}_{-8.8}$	0.0	∓0.1	±0.1	±0.3	±0.0	±1.0	±1.0	±0.5
400–500	$1.02^{+ 0.01}_{- 0.02}$	6.752e-05	120	0.0	${}^{+ 4.6}_{-3.4}$	${}^{+ 3.3}_{-2.4}$	${}^{+ 0.9}_{-0.6}$	${}^{+ 3.9}_{-2.9}$	0.0	${}^{+ 2.9}_{-2.0}$	${}^{+ 0.6}_{-0.5}$	${}^{+ 9.9}_{-9.1}$	${}^{+ 8.0}_{-7.2}$	${}^{+ 0.6}_{-0.6}$	${}^{-0.0}_{+ 0.0}$	${}^{ +12}_{ -11}$	0.0	±1.7	±0.1	±0.4	±0.0	±1.0	±1.0	±0.5

**Table 12:** Measured jet cross section for anti-*k*_*t*_ jets with *R*=0.6 in the rapidity bin 0.3≤|*y*|<0.8. See caption of Table 4 for details

*p*_T_ (GeV)	NPCorr	*σ* (nb/GeV)	$\delta_{\mathrm{stat.}}$ %	*γ*_1_ %	*γ*_7_ %	*γ*_13_ %	*γ*_19_ %	*γ*_25_ %	*γ*_88_ %	*γ*_32_ %	*γ*_38_ %	*γ*_44_ %	*γ*_50_ %	*γ*_56_ %	*γ*_62_ %	*γ*_68_ %	*γ*_89_ %	*γ*_76_ %	*γ*_82_ %	*γ*_74_ %	*γ*_75_ %	*γ*_83_ %	*u*_1_ %	*u*_2_ %
20–30	$1.24^{+ 0.07}_{- 0.13}$	2.446e+03	1.1	${}^{ +14}_{ -11}$	${}^{+11.}_{-9.6}$	${}^{ +17}_{ -13}$	${}^{+ 8.8}_{-8.4}$	${}^{+ 5.3}_{-4.7}$	${}^{+ 0.1}_{-0.1}$	${}^{+ 2.3}_{-2.3}$	${}^{+ 4.8}_{-4.4}$	${}^{+ 5.0}_{-4.9}$	${}^{-0.0}_{+ 0.0}$	${}^{+ 0.0}_{-0.0}$	${}^{+ 3.0}_{-3.0}$	${}^{-0.0}_{+ 0.0}$	${}^{+ 0.1}_{-0.1}$	±4.6	∓0.1	∓0.2	±0.6	±2.0	±1.0	±0.5
30–45	$1.13^{+ 0.07}_{- 0.08}$	3.025e+02	1.9	${}^{+ 8.1}_{-7.2}$	${}^{+11.}_{-10.}$	${}^{+10.0}_{-9.3}$	${}^{+ 8.0}_{-7.3}$	${}^{+ 2.7}_{-2.8}$	${}^{+ 0.1}_{-0.1}$	${}^{+ 2.8}_{-3.1}$	${}^{+ 3.4}_{-3.4}$	${}^{+ 5.2}_{-5.0}$	${}^{-0.0}_{+ 0.0}$	${}^{+ 0.0}_{-0.0}$	${}^{+ 3.6}_{-3.7}$	${}^{-0.2}_{+ 0.1}$	${}^{+ 0.1}_{-0.1}$	±3.3	∓0.7	±0.1	±0.3	±1.0	±1.0	±0.5
45–60	$1.07^{+ 0.05}_{- 0.04}$	4.526e+01	1.8	${}^{+ 1.3}_{-1.6}$	${}^{+ 8.7}_{-7.9}$	${}^{+ 6.9}_{-6.4}$	${}^{+ 3.6}_{-3.7}$	${}^{+ 1.6}_{-1.9}$	${}^{+ 0.1}_{-0.1}$	${}^{+ 2.9}_{-3.2}$	${}^{+ 1.8}_{-1.9}$	${}^{+ 4.5}_{-4.7}$	${}^{+ 0.1}_{-0.0}$	${}^{+ 0.0}_{+ 0.0}$	${}^{+ 2.6}_{-2.7}$	${}^{+ 0.2}_{-0.2}$	${}^{+ 0.0}_{-0.0}$	±1.1	∓0.1	±0.4	±0.1	±1.0	±1.0	±0.5
60–80	$1.04^{+ 0.03}_{- 0.03}$	9.701e+00	0.8	${}^{-1.2}_{+ 1.1}$	${}^{+ 6.4}_{-6.1}$	${}^{+ 3.8}_{-3.6}$	${}^{+ 3.0}_{-2.9}$	${}^{+ 3.6}_{-3.5}$	${}^{+ 0.0}_{-0.1}$	${}^{+ 3.2}_{-3.2}$	${}^{+ 1.6}_{-1.3}$	${}^{+ 4.8}_{-4.7}$	${}^{+ 0.4}_{-0.5}$	${}^{+ 0.0}_{-0.0}$	${}^{+ 0.7}_{-0.7}$	${}^{+ 2.1}_{-2.0}$	${}^{+ 0.0}_{-0.0}$	±1.2	∓0.0	±0.3	±0.0	±1.0	±1.0	±0.5
80–110	$1.02^{+ 0.02}_{- 0.02}$	1.791e+00	1.1	${}^{-0.5}_{+ 0.5}$	${}^{+ 6.3}_{-6.1}$	${}^{+ 3.1}_{-3.1}$	${}^{+ 2.8}_{-2.9}$	${}^{+ 2.4}_{-2.5}$	${}^{+ 0.0}_{-0.1}$	${}^{+ 3.1}_{-3.1}$	${}^{+ 1.1}_{-1.3}$	${}^{+ 5.4}_{-5.2}$	${}^{+ 1.1}_{-1.4}$	${}^{+ 0.1}_{-0.0}$	${}^{-0.3}_{+ 0.4}$	${}^{+ 4.4}_{-4.4}$	${}^{+ 0.0}_{-0.0}$	±1.0	±0.1	±0.4	±0.0	±1.0	±1.0	±0.5
110–160	$1.02^{+ 0.01}_{- 0.02}$	2.374e-01	2.3	${}^{-0.0}_{+ 0.0}$	${}^{+ 6.1}_{-5.9}$	${}^{+ 3.1}_{-3.0}$	${}^{+ 2.5}_{-2.4}$	${}^{+ 0.5}_{-0.5}$	${}^{+ 0.1}_{-0.0}$	${}^{+ 2.7}_{-2.6}$	${}^{+ 0.9}_{-0.9}$	${}^{+ 5.9}_{-5.8}$	${}^{+ 2.0}_{-1.9}$	${}^{+ 0.1}_{-0.1}$	${}^{-0.1}_{+ 0.1}$	${}^{+ 5.9}_{-6.0}$	${}^{+ 0.0}_{-0.0}$	±0.8	±0.2	±0.8	±0.0	±1.0	±1.0	±0.5
160–210	$1.02^{+ 0.01}_{- 0.02}$	2.827e-02	6.6	${}^{-0.0}_{+ 0.0}$	${}^{+ 3.9}_{-3.6}$	${}^{+ 2.8}_{-2.9}$	${}^{+ 2.4}_{-2.3}$	${}^{+ 1.8}_{-1.8}$	${}^{+ 0.1}_{-0.0}$	${}^{+ 2.6}_{-2.5}$	${}^{+ 0.7}_{-0.7}$	${}^{+ 6.9}_{-6.7}$	${}^{+ 2.9}_{-3.0}$	${}^{+ 0.1}_{-0.1}$	${}^{-0.0}_{+ 0.0}$	${}^{+ 8.1}_{-7.7}$	${}^{+ 0.0}_{-0.0}$	±1.4	∓0.2	±0.3	±0.0	±1.0	±1.0	±0.5
210–260	$1.02^{+ 0.01}_{- 0.02}$	3.640e-03	17.	${}^{-0.0}_{+ 0.0}$	${}^{+ 3.4}_{-3.6}$	${}^{+ 3.5}_{-3.4}$	${}^{+ 1.5}_{-1.6}$	${}^{+ 2.7}_{-2.7}$	${}^{+ 0.0}_{-0.0}$	${}^{+ 2.2}_{-2.1}$	${}^{+ 0.6}_{-0.6}$	${}^{+ 7.7}_{-7.2}$	${}^{+ 3.7}_{-3.6}$	${}^{+ 0.1}_{-0.2}$	${}^{-0.0}_{+ 0.0}$	${}^{+ 9.4}_{-8.6}$	${}^{+ 0.0}_{-0.0}$	±0.5	∓0.1	±0.1	±0.0	±1.0	±1.0	±0.5
260–310	$1.02^{+ 0.01}_{- 0.02}$	1.182e-03	29.	${}^{-0.0}_{+ 0.0}$	${}^{+ 5.9}_{-5.2}$	${}^{+ 3.9}_{-3.4}$	${}^{+ 2.0}_{-1.7}$	${}^{+ 4.3}_{-3.9}$	${}^{+ 0.0}_{-0.0}$	${}^{+ 2.3}_{-2.2}$	${}^{+ 0.6}_{-0.4}$	${}^{+ 8.9}_{-8.3}$	${}^{+ 5.3}_{-4.9}$	${}^{+ 0.2}_{-0.2}$	${}^{-0.0}_{+ 0.0}$	${}^{ +11}_{ -10}$	${}^{-0.0}_{-0.0}$	±0.6	∓0.2	±0.5	±0.0	±1.0	±1.0	±0.5
310–400	$1.02^{+ 0.01}_{- 0.02}$	3.585e-04	45.	${}^{-0.0}_{+ 0.0}$	${}^{+ 5.4}_{-5.1}$	${}^{+ 4.4}_{-4.3}$	${}^{+ 1.1}_{-1.0}$	${}^{+ 4.2}_{-3.8}$	${}^{+ 0.0}_{-0.0}$	${}^{+ 2.3}_{-2.1}$	${}^{+ 0.5}_{-0.5}$	${}^{+ 9.7}_{-8.8}$	${}^{+ 6.1}_{-5.6}$	${}^{+ 0.1}_{-0.2}$	${}^{-0.0}_{+ 0.0}$	${}^{ +13}_{ -12}$	${}^{+ 0.0}_{-0.0}$	±0.1	∓0.1	±0.4	±0.0	±1.0	±1.0	±0.5
400–500	$1.02^{+ 0.01}_{- 0.02}$	1.351e-04	63.	0.0	${}^{+ 6.9}_{-6.3}$	${}^{+ 4.8}_{-4.1}$	${}^{+ 0.6}_{-0.4}$	${}^{+ 3.2}_{-3.0}$	${}^{+ 0.0}_{-0.0}$	${}^{+ 1.9}_{-1.7}$	${}^{+ 0.5}_{-0.5}$	${}^{+11.}_{-9.6}$	${}^{+ 7.5}_{-6.8}$	${}^{+ 0.2}_{-0.2}$	0.0	${}^{ +15}_{ -15}$	${}^{+ 0.0}_{-0.0}$	±1.2	±0.0	±0.7	±0.0	±1.0	±1.0	±0.5

**Table 13:** Measured jet cross section for anti-*k*_*t*_ jets with *R*=0.6 in the rapidity bin 0.8≤|*y*|<1.2. See caption of Table 4 for details

*p*_T_ (GeV)	NPCorr	*σ* (nb/GeV)	$\delta_{\mathrm{stat.}}$ %	*γ*_2_ %	*γ*_8_ %	*γ*_14_ %	*γ*_20_ %	*γ*_26_ %	*γ*_88_ %	*γ*_33_ %	*γ*_39_ %	*γ*_45_ %	*γ*_51_ %	*γ*_57_ %	*γ*_63_ %	*γ*_69_ %	*γ*_89_ %	*γ*_77_ %	*γ*_82_ %	*γ*_74_ %	*γ*_75_ %	*γ*_83_ %	*u*_1_ %	*u*_2_ %
20–30	$1.25^{+ 0.08}_{- 0.12}$	2.275e+03	1.3	${}^{ +12}_{ -10}$	${}^{+ 8.6}_{-8.7}$	${}^{ +14}_{ -12}$	${}^{+10.}_{-8.2}$	${}^{+ 4.7}_{-4.0}$	${}^{+ 3.8}_{-3.9}$	${}^{+ 1.9}_{-2.0}$	${}^{+ 4.3}_{-3.8}$	${}^{+ 4.4}_{-4.2}$	${}^{-0.0}_{+ 0.0}$	${}^{+ 0.0}_{-0.0}$	${}^{+ 2.5}_{-2.7}$	${}^{-0.0}_{+ 0.0}$	${}^{+ 3.2}_{-3.3}$	±10.	±1.3	∓0.3	±0.4	±2.0	±1.0	±0.5
30–45	$1.13^{+ 0.07}_{- 0.07}$	2.557e+02	2.2	${}^{+ 8.2}_{-7.6}$	${}^{+11.}_{-10.}$	${}^{+10.}_{-9.3}$	${}^{+ 5.0}_{-4.8}$	${}^{+ 3.0}_{-2.9}$	${}^{+ 4.8}_{-4.6}$	${}^{+ 3.0}_{-3.2}$	${}^{+ 3.7}_{-3.6}$	${}^{+ 5.5}_{-5.3}$	${}^{-0.0}_{-0.0}$	${}^{+ 0.0}_{-0.1}$	${}^{+ 3.9}_{-3.9}$	${}^{-0.1}_{+ 0.2}$	${}^{+ 4.3}_{-4.2}$	±7.6	±0.0	±0.1	±0.2	±1.0	±1.0	±0.5
45–60	$1.07^{+ 0.05}_{- 0.04}$	3.977e+01	2.1	${}^{+ 1.5}_{-2.0}$	${}^{+ 8.8}_{-8.4}$	${}^{+ 7.3}_{-6.7}$	${}^{+ 2.8}_{-3.4}$	${}^{+ 1.6}_{-2.4}$	${}^{+ 3.5}_{-3.8}$	${}^{+ 3.1}_{-3.6}$	${}^{+ 1.9}_{-2.4}$	${}^{+ 4.7}_{-5.0}$	${}^{+ 0.1}_{-0.1}$	${}^{+ 0.0}_{-0.0}$	${}^{+ 2.6}_{-3.1}$	${}^{+ 0.2}_{-0.4}$	${}^{+ 3.0}_{-3.5}$	±2.5	±0.4	±0.5	±0.1	±1.0	±1.0	±0.5
60–80	$1.04^{+ 0.04}_{- 0.02}$	8.237e+00	1.0	${}^{-1.2}_{+ 1.2}$	${}^{+ 6.7}_{-6.4}$	${}^{+ 3.8}_{-3.9}$	${}^{+ 1.9}_{-1.9}$	${}^{+ 3.5}_{-3.7}$	${}^{+ 3.2}_{-3.3}$	${}^{+ 3.2}_{-3.3}$	${}^{+ 1.5}_{-1.6}$	${}^{+ 5.0}_{-5.1}$	${}^{+ 0.6}_{-0.6}$	${}^{+ 0.0}_{-0.0}$	${}^{+ 0.8}_{-0.7}$	${}^{+ 2.2}_{-2.3}$	${}^{+ 2.5}_{-2.5}$	±2.9	±0.4	±0.5	±0.0	±1.0	±1.0	±0.5
80–110	$1.03^{+ 0.02}_{- 0.02}$	1.505e+00	1.4	${}^{-0.5}_{+ 0.5}$	${}^{+ 6.3}_{-5.7}$	${}^{+ 3.2}_{-3.0}$	${}^{+ 1.3}_{-1.0}$	${}^{+ 2.6}_{-2.2}$	${}^{+ 3.4}_{-3.3}$	${}^{+ 3.1}_{-3.1}$	${}^{+ 1.2}_{-1.0}$	${}^{+ 5.4}_{-4.9}$	${}^{+ 1.3}_{-1.1}$	${}^{+ 0.0}_{-0.0}$	${}^{-0.4}_{+ 0.4}$	${}^{+ 4.6}_{-4.4}$	${}^{+ 1.4}_{-1.1}$	±1.9	∓0.4	±0.5	±0.0	±1.0	±1.0	±0.5
110–160	$1.02^{+ 0.02}_{- 0.02}$	1.959e-01	2.8	${}^{-0.1}_{+ 0.1}$	${}^{+ 6.2}_{-5.9}$	${}^{+ 3.0}_{-2.8}$	${}^{+ 1.9}_{-1.9}$	${}^{+ 0.5}_{-0.6}$	${}^{+ 3.3}_{-3.2}$	${}^{+ 2.8}_{-2.6}$	${}^{+ 0.8}_{-0.8}$	${}^{+ 6.1}_{-5.9}$	${}^{+ 1.9}_{-1.9}$	${}^{+ 0.1}_{-0.1}$	${}^{-0.1}_{+ 0.1}$	${}^{+ 6.2}_{-6.0}$	${}^{+ 0.4}_{-0.4}$	±2.0	∓0.3	±1.4	±0.0	±1.0	±1.0	±0.5
160–210	$1.02^{+ 0.02}_{- 0.02}$	2.073e-02	8.3	${}^{-0.0}_{+ 0.0}$	${}^{+ 3.6}_{-3.4}$	${}^{+ 2.8}_{-2.7}$	${}^{+ 2.1}_{-1.9}$	${}^{+ 1.7}_{-1.6}$	${}^{+ 3.5}_{-3.5}$	${}^{+ 2.4}_{-2.4}$	${}^{+ 0.6}_{-0.7}$	${}^{+ 6.9}_{-6.3}$	${}^{+ 3.0}_{-2.9}$	${}^{+ 0.1}_{-0.1}$	${}^{-0.0}_{+ 0.0}$	${}^{+ 8.1}_{-7.4}$	${}^{+ 0.6}_{-0.6}$	±3.1	±0.3	±0.2	±0.0	±1.0	±1.0	±0.5
210–260	$1.02^{+ 0.02}_{- 0.02}$	2.537e-03	21.	${}^{-0.0}_{+ 0.0}$	${}^{+ 4.0}_{-4.0}$	${}^{+ 4.0}_{-3.5}$	${}^{+ 2.4}_{-2.0}$	${}^{+ 3.3}_{-2.6}$	${}^{+ 4.4}_{-4.1}$	${}^{+ 2.5}_{-2.0}$	${}^{+ 0.5}_{-0.5}$	${}^{+ 8.3}_{-7.6}$	${}^{+ 4.3}_{-3.8}$	${}^{+ 0.2}_{-0.1}$	${}^{-0.0}_{+ 0.0}$	${}^{+11.}_{-9.3}$	${}^{+ 0.7}_{-0.7}$	±0.9	±0.2	∓0.3	±0.0	±1.0	±1.0	±0.5
260–310	$1.02^{+ 0.02}_{- 0.02}$	7.773e-04	39.	${}^{-0.0}_{+ 0.0}$	${}^{+ 5.8}_{-5.6}$	${}^{+ 3.5}_{-3.7}$	${}^{+ 2.1}_{-2.4}$	${}^{+ 4.1}_{-4.3}$	${}^{+ 4.8}_{-4.9}$	${}^{+ 2.2}_{-2.5}$	${}^{+ 0.5}_{-0.7}$	${}^{+10.}_{-9.1}$	${}^{+ 5.3}_{-5.3}$	${}^{+ 0.1}_{-0.2}$	${}^{-0.0}_{+ 0.0}$	${}^{ +13}_{ -12}$	${}^{+ 0.9}_{-1.0}$	±4.1	±0.4	±0.6	±0.0	±1.0	±1.0	±0.5
310–400	$1.02^{+ 0.02}_{- 0.02}$	1.077e-04	83.	${}^{-0.5}_{+ 0.5}$	${}^{+ 6.0}_{-6.2}$	${}^{+ 4.0}_{-4.7}$	${}^{+ 0.8}_{-1.0}$	${}^{+ 4.2}_{-4.5}$	${}^{+ 5.8}_{-5.8}$	${}^{+ 2.2}_{-2.3}$	${}^{+ 0.2}_{-0.5}$	${}^{ +11}_{ -11}$	${}^{+ 7.1}_{-7.3}$	${}^{+ 0.1}_{-0.2}$	${}^{-0.1}_{+ 0.1}$	${}^{ +15}_{ -15}$	${}^{+ 0.7}_{-1.1}$	±2.7	±0.2	±1.8	±0.0	±1.0	±1.0	±0.5

**Table 14:** Measured jet cross section for anti-*k*_*t*_ jets with *R*=0.6 in the rapidity bin 1.2≤|*y*|<2.1. See caption of Table 4 for details

*p*_T_ (GeV)	NPCorr	*σ* (nb/GeV)	$\delta_{\mathrm{stat.}}$ %	*γ*_3_ %	*γ*_9_ %	*γ*_15_ %	*γ*_21_ %	*γ*_27_ %	*γ*_88_ %	*γ*_34_ %	*γ*_40_ %	*γ*_46_ %	*γ*_52_ %	*γ*_58_ %	*γ*_64_ %	*γ*_70_ %	*γ*_89_ %	*γ*_78_ %	*γ*_82_ %	*γ*_74_ %	*γ*_75_ %	*γ*_83_ %	*u*_1_ %	*u*_2_ %
20–30	$1.24 ^{+ 0.09}_{-0.11}$	1.775e+03	1.0	${}^{ +14}_{ -11}$	${}^{+10.}_{-9.8}$	${}^{ +17}_{ -13}$	${}^{ +14}_{ -10}$	${}^{+5.2}_{-4.7}$	${}^{ +14}_{ -14}$	${}^{+ 2.3}_{-2.4}$	${}^{+ 4.8}_{-4.3}$	${}^{+ 4.9}_{-4.8}$	${}^{-0.0}_{+ 0.0}$	${}^{+ 0.0}_{-0.0}$	${}^{+3.0}_{-3.1}$	${}^{-0.0}_{+ 0.0}$	${}^{+ 5.7}_{-5.7}$	±13.	∓0.4	∓0.1	±0.4	±2.0	±1.0	±0.5
30–45	$1.12 ^{+ 0.08}_{-0.06}$	2.067e+02	1.8	${}^{+ 8.4}_{-7.5}$	${}^{+11.}_{-9.9}$	${}^{+10.}_{-9.2}$	${}^{+ 4.9}_{-4.6}$	${}^{+3.2}_{-2.7}$	${}^{ +15}_{ -13}$	${}^{+ 3.3}_{-2.9}$	${}^{+ 3.8}_{-3.5}$	${}^{+ 5.3}_{-5.1}$	${}^{+ 0.0}_{+ 0.0}$	${}^{+ 0.0}_{-0.0}$	${}^{+4.0}_{-3.8}$	${}^{-0.1}_{+ 0.1}$	${}^{+ 6.6}_{-6.3}$	±6.8	∓0.2	±0.1	±0.2	±1.0	±1.0	±0.5
45–60	$1.07 ^{+ 0.05}_{-0.03}$	2.838e+01	1.7	${}^{+ 1.6}_{-1.5}$	${}^{+ 9.0}_{-8.7}$	${}^{+ 7.3}_{-7.2}$	${}^{+ 3.4}_{-3.1}$	${}^{+1.8}_{-1.8}$	${}^{+ 8.2}_{-7.8}$	${}^{+ 3.3}_{-3.1}$	${}^{+2.0}_{-1.9}$	${}^{+ 5.1}_{-5.0}$	${}^{+ 0.1}_{-0.2}$	${}^{+0.0}_{-0.0}$	${}^{+ 2.8}_{-2.6}$	${}^{+ 0.3}_{-0.4}$	${}^{+5.8}_{-5.6}$	±1.6	∓0.3	±0.3	±0.1	±1.0	±1.0	±0.5
60–80	$1.04 ^{+ 0.04}_{-0.02}$	5.732e+00	0.8	${}^{-1.2}_{+ 1.2}$	${}^{+ 7.2}_{-6.3}$	${}^{+ 4.1}_{-3.5}$	${}^{+ 1.6}_{-1.4}$	${}^{+3.7}_{-3.4}$	${}^{+ 5.2}_{-4.4}$	${}^{+ 3.4}_{-3.0}$	${}^{+1.4}_{-1.2}$	${}^{+ 5.2}_{-4.6}$	${}^{+ 0.5}_{-0.5}$	${}^{+0.0}_{-0.0}$	${}^{+ 0.7}_{-0.6}$	${}^{+ 2.1}_{-2.0}$	${}^{+5.0}_{-4.4}$	±2.5	∓0.3	±0.3	±0.0	±1.0	±1.0	±0.5
80–110	$1.03 ^{+ 0.03}_{-0.02}$	9.314e-01	1.2	${}^{-0.4}_{+ 0.4}$	${}^{+ 6.8}_{-6.4}$	${}^{+ 3.4}_{-3.1}$	${}^{+ 1.1}_{-1.1}$	${}^{+2.4}_{-2.4}$	${}^{+ 4.2}_{-3.8}$	${}^{+ 3.1}_{-3.1}$	${}^{+1.2}_{-1.2}$	${}^{+ 5.8}_{-5.5}$	${}^{+ 1.3}_{-1.3}$	${}^{+0.0}_{-0.1}$	${}^{-0.4}_{+ 0.4}$	${}^{+ 4.8}_{-4.5}$	${}^{+4.2}_{-3.7}$	±2.2	∓0.1	±0.3	±0.0	±1.0	±1.0	±0.5
110–160	$1.03 ^{+ 0.02}_{-0.02}$	1.041e-01	2.6	${}^{-0.0}_{+0.0}$	${}^{+ 6.9}_{-6.8}$	${}^{+ 3.6}_{-3.8}$	${}^{+ 1.4}_{-1.5}$	${}^{+ 0.6}_{-0.8}$	${}^{+ 4.6}_{-4.9}$	${}^{+ 3.2}_{-3.4}$	${}^{+1.0}_{-1.1}$	${}^{+ 6.9}_{-6.9}$	${}^{+ 2.5}_{-2.4}$	${}^{+0.1}_{-0.1}$	${}^{-0.1}_{+ 0.1}$	${}^{+ 7.0}_{-7.1}$	${}^{+3.6}_{-3.9}$	±1.6	∓0.0	±0.5	±0.0	±1.0	±1.0	±0.5
160–210	$1.03 ^{+ 0.02}_{-0.02}$	1.057e-02	7.7	${}^{-0.0}_{+0.0}$	${}^{+ 5.0}_{-4.5}$	${}^{+ 3.7}_{-3.8}$	${}^{+ 1.3}_{-1.3}$	${}^{+ 2.4}_{-2.3}$	${}^{+ 6.1}_{-5.8}$	${}^{+ 3.2}_{-3.1}$	${}^{+0.9}_{-0.9}$	${}^{+ 9.1}_{-8.6}$	${}^{+ 3.9}_{-4.0}$	${}^{+0.1}_{-0.1}$	${}^{-0.0}_{+ 0.0}$	${}^{+10.}_{-9.9}$	${}^{+ 4.8}_{-4.6}$	±2.0	∓0.2	±1.0	±0.0	±1.0	±1.0	±0.5
210–260	$1.03 ^{+ 0.02}_{-0.02}$	1.331e-03	21.	${}^{-0.0}_{+0.0}$	${}^{+ 5.0}_{-4.6}$	${}^{+ 4.7}_{-4.1}$	${}^{+ 0.6}_{-0.5}$	${}^{+ 3.8}_{-3.5}$	${}^{+ 7.1}_{-6.0}$	${}^{+ 3.0}_{-2.5}$	${}^{+0.7}_{-0.6}$	${}^{+10.}_{-9.0}$	${}^{+ 5.3}_{-4.7}$	${}^{+ 0.2}_{-0.2}$	${}^{-0.0}_{+ 0.0}$	${}^{ +13}_{ -11}$	${}^{+ 5.3}_{-4.6}$	±3.1	∓0.4	±1.1	±0.0	±1.0	±1.0	±0.5
260–310	$1.03 ^{+ 0.02}_{-0.02}$	8.750e-05	79.	${}^{-0.0}_{+0.0}$	${}^{+ 8.5}_{-7.2}$	${}^{+ 5.4}_{-4.6}$	${}^{+ 1.3}_{-1.0}$	${}^{+ 6.0}_{-5.2}$	${}^{+ 8.7}_{-7.8}$	${}^{+ 3.3}_{-2.7}$	${}^{+1.1}_{-0.7}$	${}^{ +14}_{ -12}$	${}^{+ 7.8}_{-7.0}$	${}^{+ 0.3}_{-0.3}$	${}^{-0.0}_{+ 0.0}$	${}^{ +17}_{ -15}$	${}^{+ 7.0}_{-6.3}$	±0.8	∓0.4	±1.0	±0.0	±1.0	±1.0	±0.5
310–400	$1.03 ^{+ 0.02}_{-0.02}$	2.248e-05	125	${}^{-0.1}_{+0.1}$	${}^{+ 7.4}_{-7.9}$	${}^{+ 6.1}_{-6.3}$	${}^{+ 1.2}_{-1.6}$	${}^{+ 5.3}_{-5.6}$	${}^{+ 8.9}_{-9.1}$	${}^{+ 2.4}_{-2.8}$	${}^{+0.6}_{-0.5}$	${}^{ +15}_{ -15}$	${}^{+ 8.8}_{-8.9}$	${}^{+ 0.3}_{-0.1}$	${}^{-0.0}_{+ 0.0}$	${}^{ +21}_{ -19}$	${}^{+ 7.1}_{-7.4}$	±5.7	∓1.0	±0.9	±0.0	±1.0	±1.0	±0.5

**Table 15:** Measured jet cross section for anti-*k*_*t*_ jets with *R*=0.6 in the rapidity bin 2.1≤|*y*|<2.8. See caption of Table 4 for details

*p*_T_ (GeV)	NPCorr	*σ* (nb/GeV)	$\delta_{\mathrm{stat.}}$ %	*γ*_4_ %	*γ*_10_ %	*γ*_16_ %	*γ*_22_ %	*γ*_28_ %	*γ*_88_ %	*γ*_35_ %	*γ*_41_ %	*γ*_47_ %	*γ*_53_ %	*γ*_59_ %	*γ*_65_ %	*γ*_71_ %	*γ*_89_ %	*γ*_79_ %	*γ*_82_ %	*γ*_74_ %	*γ*_75_ %	*γ*_84_ %	*u*_1_ %	*u*_2_ %
20–30	$1.19 ^{+ 0.13}_{-0.07}$	1.154e+03	1.5	${}^{ +16}_{ -12}$	${}^{ +12}_{ -10}$	${}^{ +20}_{ -14}$	${}^{+ 6.4}_{-6.0}$	${}^{+5.9}_{-5.0}$	${}^{ +26}_{ -21}$	${}^{+ 2.2}_{-2.6}$	${}^{+ 5.4}_{-4.7}$	${}^{+ 5.4}_{-5.1}$	${}^{-0.0}_{+ 0.0}$	${}^{+ 0.0}_{-0.0}$	${}^{+3.0}_{-3.2}$	${}^{-0.0}_{+ 0.0}$	${}^{+ 4.5}_{-4.5}$	±20.	∓0.7	±0.0	±0.3	±2.0	±1.0	±0.5
30–45	$1.09 ^{+ 0.09}_{-0.03}$	1.225e+02	1.3	${}^{+ 8.4}_{-7.4}$	${}^{ +11}_{ -10}$	${}^{+10.}_{-9.6}$	${}^{+ 6.0}_{-5.8}$	${}^{+3.0}_{-2.9}$	${}^{ +23}_{ -19}$	${}^{+ 3.1}_{-3.1}$	${}^{+ 3.6}_{-3.6}$	${}^{+ 5.4}_{-5.4}$	${}^{+ 0.0}_{+ 0.0}$	${}^{+ 0.0}_{-0.0}$	${}^{+3.8}_{-3.9}$	${}^{-0.1}_{+ 0.1}$	${}^{+ 4.6}_{-4.8}$	±5.7	∓1.1	±0.3	±0.2	±1.0	±1.0	±0.5
45–60	$1.05 ^{+ 0.07}_{-0.03}$	1.439e+01	2.3	${}^{+ 1.6}_{-1.6}$	${}^{+10.}_{-8.8}$	${}^{+ 8.3}_{-7.3}$	${}^{+ 3.9}_{-3.5}$	${}^{+1.8}_{-2.0}$	${}^{ +14}_{ -11}$	${}^{+ 3.4}_{-3.1}$	${}^{+ 2.1}_{-2.0}$	${}^{+ 5.4}_{-4.8}$	${}^{+ 0.1}_{-0.2}$	${}^{+ 0.0}_{-0.1}$	${}^{+2.8}_{-2.7}$	${}^{+ 0.5}_{-0.4}$	${}^{+ 4.0}_{-3.6}$	±1.8	∓0.6	±0.5	±0.0	±1.0	±1.0	±0.5
60–80	$1.04 ^{+ 0.06}_{-0.03}$	2.422e+00	1.3	${}^{-1.1}_{+ 1.0}$	${}^{+ 8.4}_{-7.3}$	${}^{+ 5.0}_{-4.4}$	${}^{+ 2.2}_{-1.8}$	${}^{+4.7}_{-4.3}$	${}^{+ 9.4}_{-7.8}$	${}^{+ 4.2}_{-3.9}$	${}^{+2.0}_{-1.6}$	${}^{+ 6.3}_{-5.8}$	${}^{+ 1.0}_{-0.6}$	${}^{+0.1}_{-0.1}$	${}^{+ 1.1}_{-0.6}$	${}^{+ 2.9}_{-2.6}$	${}^{+3.9}_{-3.6}$	±2.3	∓0.1	±0.5	±0.0	±1.0	±1.0	±0.5
80–110	$1.03 ^{+ 0.05}_{-0.03}$	2.803e-01	2.4	${}^{-0.3}_{+ 0.3}$	${}^{+ 9.7}_{-8.0}$	${}^{+ 4.7}_{-4.3}$	${}^{+ 0.8}_{-0.6}$	${}^{+3.1}_{-2.8}$	${}^{+ 8.4}_{-6.9}$	${}^{+ 4.2}_{-3.9}$	${}^{+1.4}_{-1.4}$	${}^{+ 8.0}_{-7.0}$	${}^{+ 1.6}_{-1.6}$	${}^{+0.1}_{-0.0}$	${}^{-0.4}_{+ 0.4}$	${}^{+ 6.8}_{-6.2}$	${}^{+2.5}_{-2.5}$	±4.2	∓0.2	±0.6	±0.0	±1.0	±1.0	±0.5
110–160	$1.03 ^{+ 0.05}_{-0.03}$	1.515e-02	7.4	${}^{-0.0}_{+0.0}$	${}^{+11.}_{-9.3}$	${}^{+ 6.0}_{-5.3}$	${}^{+ 1.1}_{-1.0}$	${}^{+1.6}_{-1.2}$	${}^{+ 9.4}_{-8.3}$	${}^{+ 5.3}_{-4.9}$	${}^{+1.9}_{-1.6}$	${}^{+11.}_{-9.7}$	${}^{+ 3.6}_{-3.8}$	${}^{+ 0.2}_{-0.2}$	${}^{-0.1}_{+ 0.1}$	${}^{+11.}_{-9.9}$	${}^{+ 2.3}_{-2.1}$	±3.9	∓0.7	±0.9	±0.0	±1.0	±1.0	±0.5
160–210	$1.03 ^{+ 0.05}_{-0.03}$	6.440e-04	35.	${}^{-0.0}_{+0.0}$	${}^{+ 6.7}_{-6.4}$	${}^{+ 5.6}_{-4.8}$	${}^{+ 1.6}_{-1.4}$	${}^{+ 3.3}_{-3.6}$	${}^{ +11}_{ -10}$	${}^{+ 4.9}_{-4.0}$	${}^{+1.2}_{-1.1}$	${}^{ +14}_{ -12}$	${}^{+ 6.2}_{-5.3}$	${}^{+ 0.2}_{-0.2}$	${}^{-0.0}_{+ 0.0}$	${}^{ +16}_{ -14}$	${}^{+ 2.4}_{-2.5}$	±4.1	∓1.1	±2.1	±0.0	±1.0	±1.0	±0.5
210–260	$1.03 ^{+ 0.05}_{-0.03}$	6.303e-05	114	${}^{-0.0}_{+0.0}$	${}^{ +12}_{ -13}$	${}^{+9.1}_{-12.}$	${}^{+ 1.7}_{-3.9}$	${}^{+7.9}_{-10.}$	${}^{ +24}_{ -19}$	${}^{+ 3.9}_{-6.3}$	${}^{+0.6}_{-1.7}$	${}^{ +29}_{ -20}$	${}^{ +13}_{ -13}$	${}^{-0.1}_{+ 0.0}$	${}^{-0.0}_{+ 0.0}$	${}^{ +38}_{ -24}$	${}^{+ 1.4}_{-4.6}$	±11.	±0.1	∓1.9	±0.0	±1.0	±1.0	±0.5

**Table 16:** Measured jet cross section for anti-*k*_*t*_ jets with *R*=0.6 in the rapidity bin 2.8≤|*y*|<3.6. See caption of Table 4 for details

*p*_T_ (GeV)	NPCorr	*σ* (nb/GeV)	$\delta_{\mathrm{stat.}}$ %	*γ*_5_ %	*γ*_11_ %	*γ*_17_ %	*γ*_23_ %	*γ*_29_ %	*γ*_88_ %	*γ*_36_ %	*γ*_42_ %	*γ*_48_ %	*γ*_54_ %	*γ*_60_ %	*γ*_66_ %	*γ*_72_ %	*γ*_89_ %	*γ*_80_ %	*γ*_82_ %	*γ*_74_ %	*γ*_75_ %	*γ*_85_ %	*u*_1_ %	*u*_2_ %
20–30	$1.12 ^{+ 0.16}_{-0.06}$	5.583e+02	1.8	${}^{ +17}_{ -13}$	${}^{ +12}_{ -11}$	${}^{ +21}_{ -15}$	${}^{+ 7.2}_{-6.4}$	${}^{+6.2}_{-5.2}$	${}^{ +38}_{ -30}$	${}^{+ 2.7}_{-2.7}$	${}^{+ 5.7}_{-4.9}$	${}^{+ 5.8}_{-5.3}$	${}^{-0.0}_{+ 0.0}$	${}^{+ 0.0}_{-0.0}$	${}^{+3.4}_{-3.4}$	${}^{-0.0}_{+ 0.0}$	${}^{+ 7.3}_{-6.7}$	±21.	∓0.4	±0.1	±0.4	±2.0	±2.0	±0.5
30–45	$1.05 ^{+ 0.12}_{-0.04}$	4.400e+01	2.0	${}^{+ 8.9}_{-8.6}$	${}^{ +13}_{ -12}$	${}^{ +12}_{ -11}$	${}^{+ 6.4}_{-6.1}$	${}^{+3.5}_{-3.4}$	${}^{ +43}_{ -30}$	${}^{+ 3.8}_{-3.7}$	${}^{+ 4.2}_{-4.0}$	${}^{+ 6.0}_{-6.2}$	${}^{+ 0.0}_{-0.0}$	${}^{+ 0.0}_{-0.0}$	${}^{+4.5}_{-4.4}$	${}^{-0.1}_{+ 0.1}$	${}^{+ 7.7}_{-7.8}$	±10.	±0.0	±0.5	±0.3	±1.0	±2.0	±0.5
45–60	$1.03 ^{+ 0.09}_{-0.04}$	3.267e+00	2.0	${}^{+ 1.5}_{-1.2}$	${}^{ +15}_{ -12}$	${}^{+13.}_{-9.7}$	${}^{+ 6.5}_{-4.7}$	${}^{+3.0}_{-2.9}$	${}^{ +36}_{ -25}$	${}^{+ 5.5}_{-4.5}$	${}^{+ 2.8}_{-2.3}$	${}^{+ 8.7}_{-7.1}$	${}^{+ 0.7}_{-0.2}$	${}^{+ 0.0}_{-0.0}$	${}^{+3.6}_{-3.0}$	${}^{+ 1.4}_{-0.8}$	${}^{+10.}_{-8.4}$	±3.9	∓0.5	±3.1	±0.1	±1.0	±1.0	±0.5
60–80	$1.02 ^{+ 0.08}_{-0.04}$	2.558e-01	3.3	${}^{-2.0}_{+ 1.9}$	${}^{ +12}_{ -11}$	${}^{+ 5.9}_{-6.9}$	${}^{+ 3.9}_{-4.6}$	${}^{+5.8}_{-6.5}$	${}^{ +26}_{ -21}$	${}^{+ 5.3}_{-5.9}$	${}^{+ 1.6}_{-2.6}$	${}^{+ 8.7}_{-9.6}$	${}^{+ 0.6}_{-1.2}$	${}^{-0.0}_{+ 0.0}$	${}^{+0.1}_{-0.5}$	${}^{+ 4.3}_{-5.2}$	${}^{+ 8.3}_{-9.1}$	±6.0	∓0.6	±5.5	±0.0	±1.0	±1.0	±0.5
80–110	$1.02 ^{+ 0.08}_{-0.04}$	7.530e-03	12.	${}^{-0.8}_{+0.7}$	${}^{ +12}_{ -14}$	${}^{+ 4.2}_{-7.2}$	${}^{+ 5.5}_{-6.4}$	${}^{+3.1}_{-2.0}$	${}^{ +17}_{ -20}$	${}^{+ 4.1}_{-5.9}$	${}^{+ 1.2}_{-1.3}$	${}^{ +11}_{ -13}$	${}^{+ 1.9}_{-2.1}$	${}^{+ 0.1}_{-0.0}$	${}^{-0.8}_{+ 0.8}$	${}^{ +10}_{ -11}$	${}^{+ 7.9}_{-9.1}$	±4.3	±2.4	∓2.3	±0.0	±1.0	±1.0	±0.5
110–160	$1.02 ^{+ 0.08}_{-0.04}$	1.268e-04	71.	${}^{-0.1}_{+0.1}$	${}^{ +11}_{ -26}$	${}^{+5.3}_{-21.}$	${}^{+6.2}_{-22.}$	${}^{-1.9}_{-12.}$	${}^{ +23}_{ -40}$	${}^{+3.1}_{-19.}$	${}^{-0.85}_{-13.}$	${}^{ +12}_{ -27}$	${}^{+4.8}_{-16.}$	${}^{-0.0}_{+0.0}$	${}^{-0.3}_{+ 0.3}$	${}^{+8.8}_{-28.}$	${}^{+4.8}_{-23.}$	±25.	∓0.6	±25.5	±0.0	±1.0	±1.0	±0.5

**Table 17:** Measured jet cross section for anti-*k*_*t*_ jets with *R*=0.6 in the rapidity bin 3.6≤|*y*|<4.4. See caption of Table 4 for details

*p*_T_ (GeV)	NPCorr	*σ* (nb/GeV)	$\delta_{\mathrm{stat.}}$ %	*γ*_6_ %	*γ*_12_ %	*γ*_18_ %	*γ*_24_ %	*γ*_30_ %	*γ*_88_ %	*γ*_37_ %	*γ*_43_ %	*γ*_49_ %	*γ*_55_ %	*γ*_61_ %	*γ*_67_ %	*γ*_73_ %	*γ*_89_ %	*γ*_81_ %	*γ*_82_ %	*γ*_74_ %	*γ*_75_ %	*γ*_86_ %	*u*_1_ %	*u*_2_ %
20–30	$0.99 ^{+ 0.19}_{-0.06}$	8.729e+01	4.6	${}^{ +26}_{ -16}$	${}^{ +19}_{ -15}$	${}^{ +31}_{ -19}$	${}^{+ 5.7}_{-3.9}$	${}^{+9.2}_{-6.2}$	${}^{ +93}_{ -54}$	${}^{+ 4.9}_{-3.4}$	${}^{+ 8.5}_{-5.9}$	${}^{+ 8.9}_{-6.7}$	${}^{-0.0}_{+ 0.0}$	${}^{-0.0}_{-0.0}$	${}^{+5.9}_{-4.3}$	${}^{-0.0}_{+ 0.0}$	${}^{ +15}_{ -12}$	±25.	∓2.5	±1.4	±0.6	±2.0	±2.0	±0.5
30–45	$0.95 ^{+ 0.13}_{-0.08}$	2.220e+00	5.0	${}^{ +21}_{ -14}$	${}^{ +29}_{ -20}$	${}^{ +26}_{ -19}$	${}^{+ 8.2}_{-5.3}$	${}^{+8.8}_{-6.1}$	${}^{+159}_{ -56}$	${}^{+10.0}_{-6.8}$	${}^{+ 9.8}_{-7.6}$	${}^{ +14}_{ -11}$	${}^{+ 0.0}_{-0.0}$	${}^{-0.0}_{+ 0.0}$	${}^{+11.}_{-8.2}$	${}^{+ 0.0}_{-0.0}$	${}^{ +28}_{ -18}$	±14.	∓2.4	±5.4	±1.5	±1.0	±1.0	±0.5
45–60	$0.92 ^{+ 0.13}_{-0.12}$	1.433e-02	16.	${}^{+ 0.2}_{-3.3}$	${}^{ +39}_{ -22}$	${}^{ +15}_{ -21}$	${}^{+ 2.7}_{-3.0}$	${}^{+5.3}_{-8.2}$	${}^{+109}_{ -55}$	${}^{+7.1}_{-12.}$	${}^{+ 4.5}_{-7.2}$	${}^{ +13}_{ -17}$	${}^{-0.0}_{-0.8}$	${}^{-0.0}_{+ 0.0}$	${}^{+5.7}_{-7.5}$	${}^{+ 2.8}_{-4.1}$	${}^{ +38}_{ -23}$	±26.	∓12.7	±2.2	±0.4	±1.0	±1.0	±0.5

**Table 18:** Measured jet cross section ratio in bins of *x*_T_ for anti-*k*_*t*_ jets with *R*=0.4 in the rapidity bin |*y*|<0.3. NPCorr stands for multiplicative non-perturbative corrections, *ρ* is the measured cross section ratio and $\delta_{\mathrm{stat.}}$ is the statistical uncertainty. *γ*_*i*_ and *u*_*j*_ correspond to the correlated and uncorrelated systematic uncertainties given in %. They are described in Table 1, where *i* in *γ*_*i*_ denotes a nuisance parameter. *u*_3_ is the uncertainty due to pile-up effects in the cross section measurement at $\sqrt{s}=7~\mbox{TeV}$. For each table entry, the outcome of shifting the corresponding nuisance parameter by one standard deviation, up or down, is shown as superscript or subscript, respectively. In some bins these shifts may lead to cross-section variations in the same direction. The 4.3 % uncertainty from the luminosity measurements is not in this table

*x*_T_	NPCorr	*ρ*	$\delta_{\mathrm{stat.}}$ %	*γ*_1_ %	*γ*_7_ %	*γ*_13_ %	*γ*_19_ %	*γ*_25_ %	*γ*_31_ %	*γ*_32_ %	*γ*_38_ %	*γ*_44_ %	*γ*_50_ %	*γ*_56_ %	*γ*_62_ %	*γ*_68_ %	*γ*_76_ %	*γ*_82_ %	*γ*_74_ %	*γ*_75_ %	*γ*_83_ %	*u*_1_ %	*u*_2_ %	*u*_3_ %
(1.71–2.29) e-02	$0.90 ^{+ 0.03}_{-0.06}$	1.210	2.5	${}^{+6.5}_{-5.7}$	${}^{+2.6}_{-2.5}$	${}^{+3.5}_{-3.5}$	${}^{+7.6}_{-8.2}$	${}^{+1.2}_{-1.0}$	0.0	${}^{-1.1}_{+ 1.0}$	${}^{+2.9}_{-3.1}$	${}^{+ 0.3}_{-0.7}$	${}^{-0.4}_{+ 0.3}$	${}^{-0.0}_{-0.1}$	${}^{-2.1}_{+1.4}$	${}^{+ 0.1}_{-0.1}$	∓5.0	±0.2	±0.1	±0.4	±0.9	±1.4	${}^{+0.6}_{-0.5}$	±0.3
(2.29–3.14) e-02	$0.92 ^{+ 0.03}_{-0.05}$	1.289	2.9	${}^{+4.1}_{-3.9}$	${}^{+2.1}_{-2.4}$	${}^{+2.2}_{-1.9}$	${}^{+7.0}_{-6.9}$	${}^{-0.5}_{+ 1.1}$	0.0	${}^{-0.0}_{+ 0.5}$	${}^{+2.7}_{-2.0}$	${}^{+ 0.3}_{+ 0.6}$	${}^{-0.5}_{+ 1.0}$	${}^{+0.0}_{+ 0.3}$	${}^{+1.6}_{-1.5}$	${}^{+ 0.6}_{-0.2}$	∓0.3	±0.6	±0.0	±2.0	0.0	±1.4	${}^{+ 0.5}_{-0.5}$	±0.4
(3.14–4.57) e-02	$0.94 ^{+ 0.03}_{-0.03}$	1.364	2.4	${}^{+2.6}_{-2.3}$	${}^{+2.8}_{-2.5}$	${}^{+ 1.1}_{-0.8}$	${}^{+1.8}_{-1.0}$	${}^{+ 0.2}_{-0.0}$	0.0	${}^{-0.2}_{+ 0.0}$	${}^{+1.2}_{-1.1}$	${}^{-1.0}_{+ 1.0}$	${}^{-1.6}_{+1.5}$	${}^{+ 0.1}_{+0.3}$	${}^{+4.9}_{-4.4}$	${}^{-1.3}_{+1.1}$	∓0.2	∓0.1	±0.1	±0.1	0.0	±1.4	${}^{+ 0.5}_{-0.5}$	±0.4
(4.57–6.00) e-02	$0.96 ^{+ 0.03}_{-0.03}$	1.399	1.4	${}^{-0.6}_{+0.6}$	${}^{+3.0}_{-3.2}$	${}^{-0.9}_{+ 1.0}$	${}^{+ 0.6}_{-1.1}$	${}^{+0.1}_{-0.7}$	0.0	${}^{+ 0.7}_{-0.7}$	${}^{+ 0.6}_{-1.1}$	${}^{-1.7}_{+ 1.0}$	${}^{-2.0}_{+1.5}$	${}^{-0.2}_{-0.2}$	${}^{+4.7}_{-4.6}$	${}^{-4.4}_{+4.8}$	∓0.4	∓0.0	±0.2	±0.1	0.0	±1.4	${}^{+ 0.5}_{-0.5}$	±0.4
(6.00–7.43) e-02	$0.97 ^{+ 0.02}_{-0.02}$	1.411	2.0	${}^{-0.5}_{+0.5}$	${}^{+2.2}_{-1.1}$	${}^{-1.5}_{+2.0}$	${}^{+ 1.3}_{-0.6}$	${}^{+0.7}_{-0.1}$	0.0	${}^{+ 0.3}_{-0.1}$	${}^{+ 0.7}_{-0.5}$	${}^{-1.0}_{+ 2.1}$	${}^{-2.2}_{+3.0}$	${}^{-0.2}_{+ 0.3}$	${}^{+2.1}_{-2.1}$	${}^{-6.1}_{+7.3}$	∓1.2	±0.0	±0.3	±0.0	0.0	±1.4	${}^{+ 0.5}_{-0.5}$	±0.3
(7.43–8.86) e-02	$0.98 ^{+ 0.02}_{-0.02}$	1.434	2.9	${}^{-0.1}_{+0.1}$	${}^{+1.7}_{-1.4}$	${}^{+ 0.9}_{-0.9}$	${}^{+1.5}_{-1.5}$	${}^{-1.3}_{+ 0.8}$	0.0	${}^{+ 1.0}_{-1.3}$	${}^{+ 0.5}_{-1.0}$	${}^{-0.3}_{+ 0.4}$	${}^{-2.6}_{+2.3}$	${}^{-0.5}_{+ 0.3}$	${}^{+0.1}_{-0.0}$	${}^{-6.3}_{+6.5}$	∓0.6	±0.1	±0.3	±0.0	0.0	±1.4	${}^{+ 0.5}_{-0.5}$	±0.3
(8.86–11.4) e-02	$0.99 ^{+ 0.01}_{-0.01}$	1.455	4.2	${}^{-0.0}_{+0.0}$	${}^{+1.0}_{-1.0}$	${}^{+ 0.6}_{-1.0}$	${}^{+2.9}_{-3.5}$	${}^{-1.0}_{+ 1.0}$	0.0	${}^{+1.0}_{-1.1}$	${}^{+ 0.5}_{-0.9}$	${}^{-0.4}_{+ 0.5}$	${}^{-2.5}_{+2.1}$	${}^{-0.5}_{-0.0}$	${}^{-0.1}_{+0.1}$	${}^{-4.4}_{+4.4}$	∓0.3	∓0.1	±0.2	±0.0	0.0	±1.4	${}^{+ 0.5}_{-0.5}$	±0.3
(1.14–1.43) e-01	$0.99 ^{+ 0.01}_{-0.01}$	1.468	8.5	${}^{-0.0}_{+0.0}$	${}^{+3.7}_{-3.9}$	${}^{+4.1}_{-4.4}$	${}^{+2.0}_{-1.5}$	${}^{-0.3}_{+ 0.9}$	0.0	${}^{+1.9}_{-1.6}$	${}^{+ 0.8}_{-0.1}$	${}^{-0.1}_{-0.2}$	${}^{-2.1}_{+2.6}$	${}^{+ 0.2}_{+ 0.3}$	${}^{-0.1}_{+0.1}$	${}^{-2.4}_{+2.5}$	∓0.1	∓0.0	±0.3	±0.0	0.0	±1.4	${}^{+ 0.5}_{-0.5}$	±0.2
(1.43–1.71) e-01	$0.99 ^{+ 0.01}_{-0.01}$	1.365	16.	${}^{-0.0}_{+0.0}$	${}^{+3.7}_{-3.7}$	${}^{+6.3}_{-6.0}$	${}^{+ 0.1}_{-0.2}$	${}^{-1.0}_{+ 0.8}$	0.0	${}^{+1.6}_{-1.6}$	${}^{+ 0.5}_{-0.3}$	${}^{+0.4}_{-0.2}$	${}^{-2.3}_{+2.2}$	${}^{-0.2}_{+ 0.3}$	${}^{-0.0}_{+ 0.0}$	${}^{-2.4}_{+2.4}$	∓0.2	±0.2	±0.6	±0.0	0.0	±1.4	${}^{+ 0.5}_{-0.5}$	±0.2
(1.71–2.29) e-01	$0.99 ^{+ 0.01}_{-0.01}$	1.557	29.	${}^{-0.0}_{+0.0}$	${}^{+1.5}_{-1.4}$	${}^{+ 0.8}_{-0.7}$	${}^{+1.9}_{-2.0}$	${}^{-0.4}_{+ 0.7}$	0.0	${}^{+1.6}_{-1.3}$	${}^{+ 0.1}_{-0.3}$	${}^{-1.0}_{+ 0.3}$	${}^{-1.7}_{+1.8}$	${}^{-0.7}_{+ 0.4}$	${}^{-0.0}_{+0.0}$	${}^{-2.6}_{+2.2}$	∓0.3	±0.2	±0.4	±0.0	0.0	±1.4	${}^{+ 0.5}_{-0.6}$	±0.2
(2.29–2.86) e-01	$0.99 ^{+ 0.01}_{-0.01}$	1.800	63.	${}^{-0.3}_{+0.2}$	${}^{+ 1.2}_{-0.8}$	${}^{-2.8}_{+3.0}$	${}^{+ 0.4}_{-0.3}$	${}^{+1.0}_{-0.9}$	0.0	${}^{+1.4}_{-1.3}$	${}^{+ 0.0}_{-0.7}$	${}^{+0.7}_{-0.4}$	${}^{-1.3}_{+ 0.6}$	${}^{-1.3}_{+1.5}$	${}^{-0.0}_{+ 0.0}$	${}^{-1.8}_{+2.0}$	±0.6	∓0.0	±1.0	±0.0	0.0	±1.4	${}^{+ 0.5}_{-0.6}$	±0.1
(2.86–3.43) e-01	$0.99 ^{+ 0.01}_{-0.02}$	4.807	121	${}^{+2.4}_{-2.4}$	${}^{+ 0.8}_{-0.7}$	${}^{-3.1}_{+3.3}$	${}^{+2.6}_{-2.8}$	${}^{+ 0.3}_{-0.3}$	0.0	${}^{+1.2}_{-1.5}$	${}^{+0.9}_{-1.0}$	${}^{-0.1}_{-0.3}$	${}^{-2.7}_{+3.1}$	${}^{-4.2}_{+4.6}$	${}^{+ 0.3}_{-0.3}$	${}^{-6.2}_{+6.0}$	∓1.6	∓0.1	±3.9	±0.0	0.0	±1.4	${}^{+ 0.5}_{-0.6}$	±0.1

**Table 19:** Measured jet cross section ratio in *x*_T_ bin for anti-*k*_*t*_ jets with *R*=0.4 in the rapidity bin 0.3≤|*y*|<0.8. See caption of Table 18 for details

*x*_T_	NPCorr	*ρ*	$\delta_{\mathrm{stat.}}$ %	*γ*_1_ %	*γ*_7_ %	*γ*_13_ %	*γ*_19_ %	*γ*_25_ %	*γ*_31_ %	*γ*_32_ %	*γ*_38_ %	*γ*_44_ %	*γ*_50_ %	*γ*_56_ %	*γ*_62_ %	*γ*_68_ %	*γ*_76_ %	*γ*_82_ %	*γ*_74_ %	*γ*_75_ %	*γ*_83_ %	*u*_1_ %	*u*_2_ %	*u*_3_ %
(1.71–2.29) e-02	$0.90 ^{+ 0.04}_{-0.06}$	1.278	1.9	${}^{+7.1}_{-5.7}$	${}^{+4.5}_{-6.5}$	${}^{+2.8}_{-2.4}$	${}^{+5.7}_{-6.5}$	${}^{+ 0.9}_{-1.1}$	${}^{+ 0.1}_{-0.1}$	${}^{-0.7}_{+0.5}$	${}^{+2.4}_{-2.5}$	${}^{+ 0.1}_{-0.5}$	${}^{-0.5}_{+ 0.2}$	${}^{-0.0}_{-0.1}$	${}^{+2.2}_{-2.4}$	${}^{-1.6}_{+1.4}$	∓5.1	±0.2	±0.1	±0.1	±0.9	±1.4	${}^{+0.5}_{-0.5}$	±0.4
(2.29–3.14) e-02	$0.92 ^{+ 0.04}_{-0.05}$	1.309	2.3	${}^{+4.2}_{-3.9}$	${}^{+5.4}_{-4.8}$	${}^{+2.9}_{-2.4}$	${}^{+6.3}_{-5.1}$	${}^{+ 1.4}_{-0.8}$	${}^{+ 0.1}_{-0.1}$	${}^{+ 0.2}_{+0.1}$	${}^{+2.3}_{-1.8}$	${}^{+ 0.1}_{+ 0.3}$	${}^{-0.7}_{+ 1.2}$	${}^{+ 0.0}_{+ 0.3}$	${}^{+3.7}_{-3.7}$	${}^{-3.6}_{+4.0}$	∓1.6	±0.1	±0.1	±0.3	0.0	±1.4	${}^{+ 0.5}_{-0.5}$	±0.4
(3.14–4.57) e-02	$0.94 ^{+ 0.03}_{-0.04}$	1.348	1.9	${}^{+2.5}_{-2.4}$	${}^{+1.7}_{-1.7}$	${}^{+2.4}_{-3.0}$	${}^{+0.9}_{-1.3}$	${}^{+ 0.7}_{-0.8}$	${}^{+ 0.1}_{-0.1}$	${}^{+0.4}_{-0.6}$	${}^{+1.2}_{-1.3}$	${}^{-0.9}_{+ 0.3}$	${}^{-1.5}_{+1.4}$	${}^{+ 0.1}_{-0.0}$	${}^{+2.8}_{-2.7}$	${}^{-4.7}_{+5.0}$	±0.6	±0.0	±0.1	±0.1	0.0	±1.4	${}^{+ 0.5}_{-0.5}$	±0.4
(4.57–6.00) e-02	$0.96 ^{+ 0.03}_{-0.03}$	1.401	1.1	${}^{-0.6}_{+0.6}$	${}^{+1.7}_{-2.5}$	${}^{+1.1}_{-1.5}$	${}^{+ 0.9}_{-1.4}$	${}^{+0.4}_{-1.1}$	${}^{+ 0.1}_{-0.0}$	${}^{+ 0.7}_{-1.3}$	${}^{+0.2}_{-1.0}$	${}^{-0.9}_{+ 0.5}$	${}^{-1.9}_{+1.2}$	${}^{-0.3}_{-0.1}$	${}^{+ 0.4}_{-0.6}$	${}^{-4.2}_{+3.5}$	∓0.2	±0.4	±0.3	±0.1	0.0	±1.4	${}^{+ 0.5}_{-0.5}$	±0.5
(6.00–7.43) e-02	$0.97 ^{+ 0.02}_{-0.03}$	1.463	1.6	${}^{-0.5}_{+0.5}$	${}^{+2.3}_{-1.5}$	${}^{+ 1.0}_{-0.2}$	${}^{+ 1.1}_{-0.8}$	${}^{-0.6}_{+ 1.2}$	${}^{+ 0.0}_{-0.0}$	${}^{+ 1.1}_{-0.9}$	${}^{+1.0}_{-0.5}$	${}^{-1.1}_{+1.6}$	${}^{-1.8}_{+2.4}$	${}^{+ 0.2}_{+ 0.4}$	${}^{-0.3}_{+ 0.3}$	${}^{-3.4}_{+3.8}$	∓1.4	∓0.0	±0.3	±0.0	0.0	±1.4	${}^{+ 0.5}_{-0.5}$	±0.3
(7.43–8.86) e-02	$0.98 ^{+ 0.02}_{-0.02}$	1.447	2.3	${}^{-0.1}_{+0.1}$	${}^{+2.3}_{-2.7}$	${}^{+1.1}_{-2.0}$	${}^{+1.1}_{-2.1}$	${}^{-2.4}_{+1.5}$	${}^{+ 0.0}_{-0.0}$	${}^{+ 0.6}_{-1.3}$	${}^{+0.2}_{-0.8}$	${}^{-1.3}_{+1.2}$	${}^{-2.2}_{+1.6}$	${}^{-0.2}_{-0.0}$	${}^{-0.2}_{+ 0.2}$	${}^{-3.4}_{+3.0}$	∓0.3	±0.1	±0.4	±0.0	0.0	±1.4	${}^{+ 0.5}_{-0.5}$	±0.3
(8.86–11.4) e-02	$0.99 ^{+ 0.02}_{-0.02}$	1.408	3.5	${}^{-0.0}_{+0.0}$	${}^{+2.8}_{-3.0}$	${}^{+1.5}_{-1.4}$	${}^{+2.2}_{-2.2}$	${}^{-1.7}_{+1.4}$	${}^{+ 0.0}_{-0.0}$	${}^{+ 0.9}_{-1.4}$	${}^{+0.6}_{-0.5}$	${}^{-0.7}_{+ 0.3}$	${}^{-2.0}_{+2.4}$	${}^{-0.4}_{-0.2}$	${}^{-0.0}_{+ 0.0}$	${}^{-2.7}_{+2.9}$	±0.1	±0.1	±0.5	±0.0	0.0	±1.4	${}^{+ 0.5}_{-0.5}$	±0.3
(1.14–1.43) e-01	$0.99 ^{+ 0.02}_{-0.01}$	1.409	7.2	${}^{-0.0}_{+0.0}$	${}^{+2.1}_{-1.4}$	${}^{+1.9}_{-1.2}$	${}^{+2.8}_{-2.0}$	${}^{-0.6}_{+ 0.9}$	${}^{+ 0.0}_{-0.0}$	${}^{+1.7}_{-1.2}$	${}^{+0.4}_{-0.5}$	${}^{+ 0.4}_{+ 0.2}$	${}^{-1.5}_{+1.9}$	${}^{+ 0.3}_{+0.1}$	${}^{-0.0}_{+ 0.0}$	${}^{-1.8}_{+2.2}$	∓0.2	±0.1	±0.3	±0.0	0.0	±1.4	${}^{+ 0.5}_{-0.5}$	±0.3
(1.43–1.71) e-01	$0.99 ^{+ 0.02}_{-0.01}$	1.160	15.	${}^{-0.0}_{+0.0}$	${}^{+1.3}_{-1.5}$	${}^{+2.3}_{-2.2}$	${}^{+2.1}_{-1.8}$	${}^{+0.3}_{-0.1}$	${}^{+ 0.0}_{-0.1}$	${}^{+1.3}_{-1.2}$	${}^{+ 0.3}_{-0.3}$	${}^{+ 0.4}_{+ 0.3}$	${}^{-1.6}_{+1.6}$	${}^{-0.3}_{+ 0.2}$	${}^{-0.0}_{+ 0.0}$	${}^{-1.9}_{+2.7}$	∓1.0	±0.0	±0.3	±0.0	0.0	±1.4	${}^{+ 0.5}_{-0.6}$	±0.3
(1.71–2.29) e-01	$0.99 ^{+ 0.02}_{-0.02}$	1.081	25.	${}^{-0.0}_{+0.0}$	${}^{+1.8}_{-1.4}$	${}^{+1.9}_{-1.5}$	${}^{+1.3}_{-1.4}$	${}^{+0.7}_{-0.8}$	${}^{+ 0.0}_{-0.0}$	${}^{+ 1.3}_{-0.9}$	${}^{+0.1}_{-0.2}$	${}^{+ 0.7}_{-0.5}$	${}^{-2.0}_{+1.7}$	${}^{-0.7}_{+ 1.2}$	${}^{-0.0}_{+ 0.0}$	${}^{-2.0}_{+2.4}$	∓0.6	±0.1	±0.2	±0.0	0.0	±1.4	${}^{+ 0.5}_{-0.6}$	±0.2
(2.29–2.86) e-01	$0.99 ^{+ 0.02}_{-0.02}$	2.488	55.	${}^{-0.2}_{+0.2}$	${}^{-0.8}_{+ 0.8}$	${}^{-0.4}_{-0.5}$	${}^{+ 0.6}_{-1.4}$	${}^{+0.9}_{-1.2}$	${}^{+ 0.0}_{-0.0}$	${}^{+1.1}_{-1.9}$	${}^{-0.0}_{-0.7}$	${}^{+1.0}_{-1.2}$	${}^{-0.6}_{+ 1.0}$	${}^{-2.9}_{+2.7}$	${}^{-0.0}_{+0.0}$	${}^{-2.2}_{+1.9}$	±0.9	±0.1	±0.7	±0.0	0.0	±1.4	${}^{+ 0.5}_{-0.6}$	±0.2
(2.86–3.43) e-01	$0.99 ^{+ 0.02}_{-0.03}$	7.914	69.	${}^{+2.3}_{-2.2}$	${}^{-0.3}_{-0.8}$	${}^{+1.5}_{-1.7}$	${}^{+2.1}_{-2.5}$	${}^{+ 0.3}_{-0.6}$	${}^{+ 0.0}_{-0.0}$	${}^{+1.4}_{-1.2}$	${}^{+ 0.6}_{-0.6}$	${}^{-1.1}_{-0.4}$	${}^{-2.8}_{+2.0}$	${}^{-5.9}_{+6.3}$	${}^{+ 0.5}_{-0.5}$	${}^{-6.2}_{+5.7}$	±1.2	±0.1	±2.3	±0.0	0.0	±1.4	${}^{+ 0.5}_{-0.6}$	±0.1

**Table 20:** Measured jet cross section ratio in *x*_T_ bin for anti-*k*_*t*_ jets with *R*=0.4 in the rapidity bin 0.8≤|*y*|<1.2. See caption of Table 18 for details

*x*_T_	NPCorr	*ρ*	$\delta_{\mathrm{stat.}}$ %	*γ*_2_ %	*γ*_8_ %	*γ*_14_ %	*γ*_20_ %	*γ*_26_ %	*γ*_31_ %	*γ*_33_ %	*γ*_39_ %	*γ*_45_ %	*γ*_51_ %	*γ*_57_ %	*γ*_63_ %	*γ*_69_ %	*γ*_77_ %	*γ*_82_ %	*γ*_74_ %	*γ*_75_ %	*γ*_83_ %	*u*_1_ %	*u*_2_ %	*u*_3_ %
(1.71–2.29) e-02	$0.90 ^{+ 0.04}_{-0.06}$	1.158	2.3	${}^{+6.9}_{-6.0}$	${}^{+4.8}_{-6.2}$	${}^{+2.7}_{-2.7}$	${}^{+6.3}_{-5.1}$	${}^{+ 0.9}_{-0.9}$	${}^{+3.1}_{-3.7}$	${}^{-0.4}_{+0.6}$	${}^{+2.2}_{-2.4}$	${}^{+ 0.1}_{-0.6}$	${}^{-0.4}_{+ 0.2}$	${}^{-0.0}_{-0.1}$	${}^{+2.2}_{-2.2}$	${}^{-1.7}_{+1.6}$	∓11	±0.5	±0.2	±0.2	±0.9	±1.4	${}^{+0.6}_{-0.5}$	±0.5
(2.29–3.14) e-02	$0.93 ^{+ 0.03}_{-0.04}$	1.209	2.7	${}^{+4.3}_{-3.8}$	${}^{+5.7}_{-4.6}$	${}^{+2.5}_{-2.1}$	${}^{+ 0.9}_{+0.1}$	${}^{+ 1.3}_{-0.6}$	${}^{+4.4}_{-4.2}$	${}^{+ 0.0}_{+ 0.4}$	${}^{+2.2}_{-1.8}$	${}^{+ 0.2}_{+ 0.2}$	${}^{-1.0}_{+ 1.3}$	${}^{+0.1}_{+ 0.3}$	${}^{+3.9}_{-3.7}$	${}^{-3.9}_{+4.2}$	∓3.5	∓0.1	±0.2	±0.5	0.0	±1.4	${}^{+ 0.5}_{-0.5}$	±0.8
(3.14–4.57) e-02	$0.95 ^{+ 0.02}_{-0.04}$	1.317	2.3	${}^{+2.7}_{-2.4}$	${}^{+2.6}_{-1.6}$	${}^{+3.1}_{-2.8}$	${}^{-0.4}_{+0.6}$	${}^{+ 0.9}_{-0.5}$	${}^{+4.7}_{-4.2}$	${}^{+ 0.6}_{-0.5}$	${}^{+1.5}_{-1.1}$	${}^{-0.0}_{+ 0.4}$	${}^{-1.5}_{+1.7}$	${}^{+0.1}_{-0.0}$	${}^{+3.1}_{-2.8}$	${}^{-4.5}_{+5.4}$	∓1.6	±0.1	±0.2	±0.1	0.0	±1.4	${}^{+ 0.5}_{-0.5}$	±0.7
(4.57–6.00) e-02	$0.97 ^{+ 0.02}_{-0.03}$	1.406	1.3	${}^{-0.7}_{+0.7}$	${}^{+1.5}_{-2.4}$	${}^{+ 0.9}_{-1.0}$	${}^{+ 0.3}_{-0.7}$	${}^{+0.4}_{-0.6}$	${}^{+2.0}_{-2.1}$	${}^{+ 0.7}_{-0.9}$	${}^{+ 0.5}_{-0.7}$	${}^{-1.1}_{+1.2}$	${}^{-1.9}_{+1.6}$	${}^{-0.4}_{-0.1}$	${}^{+0.4}_{-0.3}$	${}^{-4.2}_{+4.1}$	∓0.4	±0.0	±0.6	±0.0	0.0	±1.4	${}^{+ 0.5}_{-0.5}$	±0.7
(6.00–7.43) e-02	$0.98 ^{+ 0.02}_{-0.02}$	1.486	2.0	${}^{-0.6}_{+0.5}$	${}^{+2.0}_{-2.1}$	${}^{+ 0.8}_{-0.8}$	${}^{+ 1.0}_{-1.0}$	${}^{-0.8}_{+ 1.0}$	${}^{+ 0.5}_{-0.3}$	${}^{+1.2}_{-1.1}$	${}^{+1.0}_{-0.6}$	${}^{-1.1}_{+ 0.9}$	${}^{-1.9}_{+1.9}$	${}^{+ 0.2}_{+0.4}$	${}^{-0.4}_{+ 0.4}$	${}^{-3.3}_{+3.1}$	±0.1	±0.2	±0.7	±0.0	0.0	±1.4	${}^{+ 0.5}_{-0.5}$	±0.7
(7.43–8.86) e-02	$0.99 ^{+ 0.02}_{-0.02}$	1.470	2.8	${}^{-0.1}_{+0.1}$	${}^{+3.3}_{-2.9}$	${}^{+2.3}_{-2.2}$	${}^{+1.5}_{-1.6}$	${}^{-1.9}_{+1.8}$	${}^{-0.4}_{+ 0.4}$	${}^{+1.3}_{-1.3}$	${}^{+0.2}_{-0.7}$	${}^{-0.4}_{+ 1.3}$	${}^{-1.8}_{+1.9}$	${}^{-0.2}_{+ 0.0}$	${}^{-0.2}_{+ 0.2}$	${}^{-2.4}_{+3.2}$	∓0.4	±0.4	±0.8	±0.0	0.0	±1.4	${}^{+ 0.5}_{-0.5}$	±0.4
(8.86–11.4) e-02	$0.99 ^{+ 0.02}_{-0.02}$	1.441	4.2	${}^{-0.0}_{+0.0}$	${}^{+3.4}_{-3.6}$	${}^{+1.7}_{-1.5}$	${}^{+ 1.0}_{-1.6}$	${}^{-1.6}_{+ 1.0}$	${}^{-0.3}_{-0.2}$	${}^{+1.0}_{-1.6}$	${}^{+0.6}_{-0.7}$	${}^{-0.3}_{-0.1}$	${}^{-1.9}_{+1.8}$	${}^{-0.4}_{-0.2}$	${}^{-0.0}_{+ 0.0}$	${}^{-2.5}_{+2.4}$	∓1.5	±0.2	±1.0	±0.0	0.0	±1.4	${}^{+ 0.5}_{-0.5}$	±0.5
(1.14–1.43) e-01	$0.99 ^{+ 0.02}_{-0.01}$	1.491	8.8	${}^{-0.0}_{+0.0}$	${}^{+1.4}_{-1.4}$	${}^{+ 0.9}_{-1.3}$	${}^{+1.5}_{-2.0}$	${}^{-0.8}_{+ 1.0}$	${}^{-0.2}_{+ 0.5}$	${}^{+1.2}_{-1.2}$	${}^{+0.5}_{-0.6}$	${}^{-0.2}_{+ 0.2}$	${}^{-1.6}_{+2.0}$	${}^{+ 0.4}_{+0.1}$	${}^{-0.0}_{+ 0.0}$	${}^{-2.4}_{+2.6}$	∓2.0	∓0.0	±0.2	±0.0	0.0	±1.4	${}^{+ 0.5}_{-0.5}$	±0.4
(1.43–1.71) e-01	$0.99 ^{+ 0.02}_{-0.02}$	1.214	18.	${}^{-0.0}_{+0.0}$	${}^{+1.1}_{-1.8}$	${}^{+1.9}_{-2.1}$	${}^{+2.6}_{-2.8}$	${}^{-0.1}_{+ 0.0}$	${}^{-0.1}_{-0.3}$	${}^{+ 0.8}_{-1.1}$	${}^{+0.1}_{-0.1}$	${}^{-0.0}_{+ 0.0}$	${}^{-1.9}_{+2.0}$	${}^{-0.5}_{+ 0.6}$	${}^{-0.0}_{+ 0.0}$	${}^{-2.1}_{+1.6}$	±1.4	±0.4	±0.4	±0.0	0.0	±1.4	${}^{+ 0.5}_{-0.7}$	±0.5
(1.71–2.29) e-01	$0.99 ^{+ 0.02}_{-0.02}$	1.554	30.	${}^{-0.0}_{+0.0}$	${}^{+2.5}_{-1.4}$	${}^{+2.3}_{-1.7}$	${}^{+ 1.2}_{-0.7}$	${}^{+1.0}_{-0.8}$	${}^{+ 0.3}_{+ 0.6}$	${}^{+ 1.8}_{-0.9}$	${}^{+0.1}_{-0.1}$	${}^{+ 1.9}_{-0.4}$	${}^{-2.0}_{+2.1}$	${}^{-1.0}_{+1.2}$	${}^{-0.0}_{+ 0.0}$	${}^{-1.6}_{+3.0}$	∓1.7	∓0.0	±0.2	±0.0	0.0	±1.4	${}^{+ 0.7}_{-0.7}$	±0.3

**Table 21:** Measured jet cross section ratio in *x*_T_ bin for anti-*k*_*t*_ jets with *R*=0.4 in the rapidity bin 1.2≤|*y*|<2.1. See caption of Table 18 for details

*x*_T_	NPCorr	*ρ*	$\delta_{\mathrm{stat.}}$ %	*γ*_3_ %	*γ*_9_ %	*γ*_15_ %	*γ*_21_ %	*γ*_27_ %	*γ*_31_ %	*γ*_34_ %	*γ*_40_ %	*γ*_46_ %	*γ*_52_ %	*γ*_58_ %	*γ*_64_ %	*γ*_70_ %	*γ*_78_ %	*γ*_82_ %	*γ*_74_ %	*γ*_75_ %	*γ*_83_ %	*u*_1_ %	*u*_2_ %	*u*_3_ %
(1.71–2.29) e-02	$0.91 ^{+ 0.03}_{-0.06}$	1.236	1.8	${}^{+7.1}_{-5.9}$	${}^{+4.2}_{-5.3}$	${}^{+2.5}_{-1.7}$	${}^{+4.2}_{-3.4}$	${}^{+ 1.0}_{-0.9}$	${}^{+8.9}_{-9.4}$	${}^{-0.6}_{+0.7}$	${}^{+2.5}_{-2.3}$	${}^{-0.0}_{+ 0.3}$	${}^{-0.3}_{+ 0.2}$	${}^{+0.0}_{-0.1}$	${}^{+2.2}_{-2.3}$	${}^{-1.7}_{+1.8}$	∓11	±0.5	±0.1	±0.4	±0.9	±1.4	${}^{+0.5}_{-0.5}$	±0.5
(2.29–3.14) e-02	$0.93 ^{+ 0.03}_{-0.04}$	1.275	2.4	${}^{+4.3}_{-3.9}$	${}^{+5.4}_{-4.9}$	${}^{+2.4}_{-2.2}$	${}^{-0.4}_{+0.8}$	${}^{+ 1.0}_{-0.6}$	${}^{+11.}_{-9.6}$	${}^{-0.3}_{+ 0.5}$	${}^{+2.0}_{-1.6}$	${}^{-0.3}_{+ 0.4}$	${}^{-1.1}_{+1.3}$	${}^{+ 0.1}_{+0.3}$	${}^{+3.9}_{-3.6}$	${}^{-4.2}_{+4.3}$	∓2.2	±0.5	±0.1	±0.3	0.0	±1.4	${}^{+ 0.5}_{-0.5}$	±0.5
(3.14–4.57) e-02	$0.95 ^{+ 0.02}_{-0.03}$	1.278	1.9	${}^{+2.5}_{-2.6}$	${}^{+2.2}_{-1.7}$	${}^{+3.0}_{-2.8}$	${}^{-0.4}_{+0.6}$	${}^{+ 0.7}_{-0.9}$	${}^{+6.9}_{-6.3}$	${}^{+ 0.5}_{-0.7}$	${}^{+1.3}_{-1.4}$	${}^{-0.4}_{+ 0.4}$	${}^{-1.5}_{+1.5}$	${}^{+0.1}_{-0.0}$	${}^{+2.8}_{-2.8}$	${}^{-4.6}_{+5.2}$	∓0.2	∓0.0	±0.3	±0.0	0.0	±1.4	${}^{+ 0.5}_{-0.5}$	±0.6
(4.57–6.00) e-02	$0.97 ^{+ 0.02}_{-0.02}$	1.315	1.3	${}^{-0.7}_{+0.7}$	${}^{+1.8}_{-2.1}$	${}^{+1.1}_{-1.5}$	${}^{-0.4}_{+ 0.1}$	${}^{+0.8}_{-0.8}$	${}^{+2.3}_{-2.0}$	${}^{+1.0}_{-1.1}$	${}^{+ 0.5}_{-0.8}$	${}^{-0.9}_{+ 1.0}$	${}^{-2.0}_{+1.7}$	${}^{-0.3}_{-0.1}$	${}^{+0.4}_{-0.3}$	${}^{-4.4}_{+4.1}$	∓0.5	±0.3	±0.5	±0.0	0.0	±1.4	${}^{+ 0.5}_{-0.5}$	±0.5
(6.00–7.43) e-02	$0.98 ^{+ 0.01}_{-0.02}$	1.353	1.6	${}^{-0.5}_{+0.5}$	${}^{+2.6}_{-2.1}$	${}^{+ 1.4}_{-0.6}$	${}^{+ 0.1}_{+ 0.2}$	${}^{-0.5}_{+ 1.3}$	${}^{+ 0.4}_{-0.2}$	${}^{+ 1.5}_{-0.9}$	${}^{+1.3}_{-0.4}$	${}^{-0.7}_{+ 1.1}$	${}^{-1.4}_{+2.2}$	${}^{+ 0.2}_{+0.4}$	${}^{-0.4}_{+ 0.4}$	${}^{-3.2}_{+3.5}$	∓0.8	±0.1	±0.7	±0.0	0.0	±1.4	${}^{+ 0.5}_{-0.5}$	±0.6
(7.43–8.86) e-02	$0.99 ^{+ 0.02}_{-0.02}$	1.415	2.7	${}^{-0.1}_{+0.1}$	${}^{+3.1}_{-3.5}$	${}^{+1.7}_{-2.4}$	${}^{-0.6}_{+ 0.3}$	${}^{-2.2}_{+1.9}$	${}^{-1.0}_{+ 0.6}$	${}^{+ 0.9}_{-1.4}$	${}^{+0.4}_{-0.7}$	${}^{-1.2}_{+ 0.7}$	${}^{-2.0}_{+2.2}$	${}^{-0.2}_{-0.0}$	${}^{-0.2}_{+ 0.1}$	${}^{-3.3}_{+2.7}$	∓1.1	±0.1	±0.4	±0.0	0.0	±1.4	${}^{+ 0.5}_{-0.5}$	±0.5
(8.86–11.4) e-02	$0.99 ^{+ 0.02}_{-0.02}$	1.463	3.9	${}^{-0.0}_{+0.0}$	${}^{+3.4}_{-3.4}$	${}^{+1.9}_{-1.7}$	${}^{-0.5}_{-0.3}$	${}^{-2.0}_{+1.1}$	${}^{-0.4}_{+ 0.5}$	${}^{+1.1}_{-1.6}$	${}^{+0.4}_{-0.8}$	${}^{-0.3}_{+ 0.4}$	${}^{-2.5}_{+2.2}$	${}^{-0.4}_{-0.2}$	${}^{-0.0}_{+ 0.0}$	${}^{-2.3}_{+3.2}$	∓0.8	±0.1	±0.1	±0.0	0.0	±1.4	${}^{+ 0.5}_{-0.5}$	±0.4
(1.14–1.43) e-01	$0.99 ^{+ 0.03}_{-0.02}$	1.855	8.0	${}^{-0.0}_{+0.0}$	${}^{+2.5}_{-1.7}$	${}^{+2.3}_{-1.4}$	${}^{-0.0}_{+ 0.6}$	${}^{-0.2}_{+ 1.3}$	${}^{+ 0.1}_{-0.1}$	${}^{+2.1}_{-1.3}$	${}^{+0.8}_{-0.3}$	${}^{+ 0.5}_{-0.2}$	${}^{-1.4}_{+2.3}$	${}^{+ 0.5}_{+0.2}$	${}^{-0.0}_{+ 0.0}$	${}^{-2.0}_{+2.4}$	∓1.2	±0.1	±0.6	±0.0	0.0	±1.4	${}^{+ 0.5}_{-0.5}$	±0.5
(1.43–1.71) e-01	$0.99 ^{+ 0.03}_{-0.03}$	1.702	19.	${}^{-0.0}_{+0.0}$	${}^{+2.1}_{-2.6}$	${}^{+3.0}_{-2.7}$	${}^{-0.4}_{+ 0.4}$	${}^{+0.6}_{-0.3}$	${}^{+ 0.3}_{-0.2}$	${}^{+1.7}_{-1.4}$	${}^{+ 0.4}_{-0.3}$	${}^{+ 0.7}_{-0.2}$	${}^{-1.6}_{+2.2}$	${}^{-0.4}_{+ 0.5}$	${}^{-0.0}_{+ 0.0}$	${}^{-2.2}_{+2.5}$	∓3.4	±0.0	±1.1	±0.0	0.0	±1.4	${}^{+ 0.6}_{-1.0}$	±0.5
(1.71–2.29) e-01	$0.99 ^{+ 0.04}_{-0.03}$	0.483	70.	${}^{-0.0}_{+0.0}$	${}^{+1.8}_{-1.8}$	${}^{+2.0}_{-1.9}$	${}^{+1.8}_{-1.3}$	${}^{+0.8}_{-1.0}$	${}^{-1.5}_{-1.2}$	${}^{+1.9}_{-1.5}$	${}^{+ 0.2}_{-0.2}$	${}^{-0.1}_{-1.8}$	${}^{-3.5}_{+2.2}$	${}^{-1.6}_{+2.1}$	${}^{-0.0}_{+0.0}$	${}^{-4.6}_{+2.0}$	±0.1	±0.1	±0.7	±0.0	0.0	±1.4	${}^{+ 0.6}_{-1.1}$	±0.4
(2.29–2.86) e-01	$0.98 ^{+ 0.04}_{-0.04}$	3.793	122	${}^{-0.2}_{+0.2}$	${}^{-1.3}_{+1.7}$	${}^{-0.4}_{+ 0.0}$	${}^{+1.4}_{-1.9}$	${}^{+1.3}_{-0.8}$	${}^{+ 0.2}_{+ 0.7}$	${}^{+1.7}_{-2.1}$	${}^{+0.2}_{-0.6}$	${}^{+ 1.4}_{-0.5}$	${}^{-1.6}_{+2.0}$	${}^{-4.8}_{+5.2}$	${}^{-0.1}_{+ 0.1}$	${}^{-2.7}_{+3.8}$	±0.1	±0.2	±1.4	±0.0	0.0	±1.4	${}^{+ 0.5}_{-1.3}$	±0.4

**Table 22:** Measured jet cross section ratio in *x*_T_ bin for anti-*k*_*t*_ jets with *R*=0.4 in the rapidity bin 2.1≤|*y*|<2.8. See caption of Table 18 for details

*x*_T_	NPCorr	*ρ*	$\delta_{\mathrm{stat.}}$ %	*γ*_4_ %	*γ*_10_ %	*γ*_16_ %	*γ*_22_ %	*γ*_28_ %	*γ*_31_ %	*γ*_35_ %	*γ*_41_ %	*γ*_47_ %	*γ*_53_ %	*γ*_59_ %	*γ*_65_ %	*γ*_71_ %	*γ*_79_ %	*γ*_82_ %	*γ*_74_ %	*γ*_75_ %	*γ*_84_ %	*u*_1_ %	*u*_2_ %	*u*_3_ %
(1.71–2.29) e-02	$0.91 ^{+ 0.04}_{-0.05}$	1.242	2.6	${}^{+6.7}_{-6.4}$	${}^{+3.4}_{-6.0}$	${}^{+2.0}_{-2.2}$	${}^{+4.4}_{-4.7}$	${}^{+ 1.1}_{-0.9}$	${}^{ +18}_{ -20}$	${}^{-0.7}_{+0.9}$	${}^{+2.2}_{-2.3}$	${}^{-0.5}_{+ 0.2}$	${}^{-0.3}_{+ 0.4}$	${}^{-0.0}_{-0.1}$	${}^{+2.2}_{-2.4}$	${}^{-1.7}_{+2.0}$	∓10	±0.2	±0.2	±0.3	±0.9	±1.4	${}^{+0.5}_{-0.5}$	±0.6
(2.29–3.14) e-02	$0.94 ^{+ 0.03}_{-0.04}$	1.296	1.7	${}^{+4.4}_{-3.8}$	${}^{+6.1}_{-5.0}$	${}^{+3.0}_{-1.9}$	${}^{+3.8}_{-3.1}$	${}^{+ 1.4}_{-0.5}$	${}^{ +20}_{ -16}$	${}^{+ 0.1}_{+0.9}$	${}^{+2.2}_{-1.4}$	${}^{+ 0.2}_{+ 0.9}$	${}^{-1.0}_{+ 1.5}$	${}^{+ 0.1}_{+ 0.4}$	${}^{+3.9}_{-3.6}$	${}^{-3.9}_{+4.8}$	∓1.6	±0.2	±0.3	±0.1	0.0	±1.4	${}^{+ 0.5}_{-0.5}$	±0.9
(3.14–4.57) e-02	$0.96 ^{+ 0.03}_{-0.03}$	1.246	2.6	${}^{+3.3}_{-2.6}$	${}^{+2.6}_{-1.3}$	${}^{+3.6}_{-2.8}$	${}^{+1.5}_{-1.1}$	${}^{+ 0.8}_{-0.7}$	${}^{+12.}_{-9.0}$	${}^{+0.7}_{-0.7}$	${}^{+1.6}_{-1.1}$	${}^{-0.6}_{+ 1.1}$	${}^{-1.7}_{+1.6}$	${}^{+ 0.0}_{-0.0}$	${}^{+3.3}_{-2.6}$	${}^{-5.2}_{+6.0}$	±0.5	±0.2	±0.5	±0.1	0.0	±1.4	${}^{+ 0.5}_{-0.5}$	±0.8
(4.57–6.00) e-02	$0.97 ^{+ 0.03}_{-0.02}$	1.402	1.8	${}^{-0.8}_{+0.7}$	${}^{+2.0}_{-2.5}$	${}^{+ 0.9}_{-1.0}$	${}^{-0.3}_{-0.3}$	${}^{+0.3}_{-0.8}$	${}^{+2.2}_{-2.5}$	${}^{+ 0.8}_{-0.9}$	${}^{+ 0.6}_{-1.1}$	${}^{-1.1}_{+1.4}$	${}^{-2.4}_{+2.0}$	${}^{-0.3}_{-0.2}$	${}^{+0.5}_{-0.4}$	${}^{-5.2}_{+4.7}$	∓0.4	±0.2	±0.9	±0.0	0.0	±1.4	${}^{+ 0.5}_{-0.5}$	±1.0
(6.00–7.43) e-02	$0.97 ^{+ 0.03}_{-0.03}$	1.350	3.4	${}^{-0.4}_{+0.4}$	${}^{+2.7}_{-3.6}$	${}^{+ 0.9}_{-1.7}$	${}^{-0.2}_{+ 0.1}$	${}^{-1.3}_{+1.3}$	${}^{-1.0}_{-0.5}$	${}^{+1.3}_{-2.2}$	${}^{+0.9}_{-0.9}$	${}^{-2.0}_{+1.0}$	${}^{-3.1}_{+2.9}$	${}^{+ 0.2}_{+ 0.4}$	${}^{-0.4}_{+ 0.3}$	${}^{-4.5}_{+4.0}$	±0.6	±0.0	±0.8	±0.0	0.0	±1.4	${}^{+ 0.5}_{-0.5}$	±0.8
(7.43–8.86) e-02	$0.97 ^{+ 0.04}_{-0.03}$	1.427	6.6	${}^{-0.0}_{+0.0}$	${}^{+3.6}_{-4.7}$	${}^{+1.7}_{-3.0}$	${}^{-2.2}_{+ 0.9}$	${}^{-3.6}_{+2.1}$	${}^{-0.9}_{+ 0.4}$	${}^{+ 0.7}_{-1.3}$	${}^{+0.1}_{-0.9}$	${}^{-1.0}_{+ 1.0}$	${}^{-3.4}_{+2.4}$	${}^{-0.5}_{-0.0}$	${}^{-0.1}_{+ 0.1}$	${}^{-3.6}_{+4.7}$	∓0.8	±0.2	±1.1	±0.0	0.0	±1.4	${}^{+ 0.6}_{-0.5}$	±0.8
(8.86–11.4) e-02	$0.97 ^{+ 0.05}_{-0.04}$	1.411	13.	${}^{-0.0}_{+0.0}$	${}^{+4.1}_{-6.2}$	${}^{+1.5}_{-3.7}$	${}^{-0.5}_{-0.6}$	${}^{-2.9}_{+1.0}$	${}^{-0.7}_{-0.7}$	${}^{+1.6}_{-3.4}$	${}^{+1.1}_{-1.3}$	${}^{-1.5}_{-1.3}$	${}^{-3.8}_{+2.3}$	${}^{-0.6}_{-0.3}$	${}^{-0.0}_{+ 0.0}$	${}^{-4.9}_{+3.6}$	∓4.2	±0.3	±0.7	±0.0	0.0	±1.4	${}^{+ 0.5}_{-0.5}$	±0.9
(1.14–1.43) e-01	$0.97 ^{+ 0.06}_{-0.04}$	2.975	36.	${}^{-0.0}_{+0.0}$	${}^{+ 0.6}_{-2.4}$	${}^{+2.1}_{-3.2}$	${}^{+ 1.7}_{-0.0}$	${}^{-1.7}_{+1.0}$	${}^{-0.6}_{+ 0.1}$	${}^{+2.2}_{-2.5}$	${}^{-0.1}_{-0.5}$	${}^{-0.6}_{-0.3}$	${}^{-4.8}_{+3.9}$	${}^{+ 0.2}_{+1.3}$	${}^{-0.0}_{+ 0.0}$	${}^{-6.6}_{+3.9}$	±0.4	±0.1	±1.4	±0.0	0.0	±1.4	${}^{+ 0.5}_{-0.5}$	±1.0
(1.43–1.71) e-01	$0.96 ^{+ 0.07}_{-0.05}$	13.135	155	${}^{-0.2}_{+ 0.2}$	${}^{+10.}_{-9.7}$	${}^{+9.4}_{-10.}$	${}^{-1.4}_{-1.8}$	${}^{+4.0}_{-6.8}$	${}^{+17.}_{-3.4}$	${}^{+5.0}_{-6.0}$	${}^{+ 1.0}_{-2.6}$	${}^{+16.9}_{+ 0.2}$	${}^{+3.1}_{-0.9}$	${}^{-0.0}_{+ 0.0}$	${}^{-0.0}_{+ 0.0}$	${}^{+15.}_{+2.2}$	∓7.6	±1.7	±6.9	±0.0	0.0	±1.4	${}^{+ 0.5}_{-0.6}$	±0.1

**Table 23:** Measured jet cross section ratio in *x*_T_ bin for anti-*k*_*t*_ jets with *R*=0.4 in the rapidity bin 2.8≤|*y*|<3.6. See caption of Table 18 for details

*x*_T_	NPCorr	*ρ*	$\delta_{\mathrm{stat.}}$ %	*γ*_5_ %	*γ*_11_ %	*γ*_17_ %	*γ*_23_ %	*γ*_29_ %	*γ*_31_ %	*γ*_36_ %	*γ*_42_ %	*γ*_48_ %	*γ*_54_ %	*γ*_60_ %	*γ*_66_ %	*γ*_72_ %	*γ*_80_ %	*γ*_82_ %	*γ*_74_ %	*γ*_75_ %	*γ*_85_ %	*u*_1_ %	*u*_2_ %	*u*_3_ %
(1.71–2.29) e-02	$0.91 ^{+ 0.05}_{-0.04}$	1.147	3.2	${}^{+8.2}_{-6.5}$	${}^{+7.0}_{-8.3}$	${}^{+4.0}_{-3.4}$	${}^{+1.1}_{-0.3}$	${}^{+1.5}_{-1.2}$	${}^{ +29}_{ -26}$	${}^{-0.5}_{+ 0.4}$	${}^{+3.0}_{-2.8}$	${}^{+ 0.5}_{-0.7}$	${}^{-0.4}_{+ 0.4}$	${}^{-0.0}_{-0.1}$	${}^{+2.7}_{-2.9}$	${}^{-2.1}_{+2.1}$	∓14	±0.6	±0.3	±0.3	±0.9	±2.2	${}^{+0.5}_{-0.5}$	±3.9
(2.29–3.14) e-02	$0.92 ^{+ 0.05}_{-0.03}$	1.196	4.2	${}^{+4.8}_{-5.2}$	${}^{+7.4}_{-5.2}$	${}^{+4.1}_{-3.0}$	${}^{-1.7}_{+2.3}$	${}^{+1.4}_{-1.9}$	${}^{ +33}_{ -24}$	${}^{+0.2}_{-0.4}$	${}^{+2.3}_{-2.8}$	${}^{-0.2}_{+ 1.0}$	${}^{-1.3}_{+1.7}$	${}^{+ 0.1}_{+ 0.3}$	${}^{+4.4}_{-5.2}$	${}^{-5.0}_{+6.2}$	∓1.6	±0.2	±0.8	±0.7	0.0	±2.2	${}^{+0.5}_{-0.5}$	±4.8
(3.14–4.57) e-02	$0.93 ^{+ 0.05}_{-0.03}$	1.200	2.7	${}^{+3.0}_{-2.6}$	${}^{+4.0}_{-2.4}$	${}^{+5.2}_{-4.1}$	${}^{-0.4}_{+3.3}$	${}^{+ 1.0}_{-1.0}$	${}^{ +20}_{ -18}$	${}^{+ 1.3}_{-0.8}$	${}^{+1.7}_{-1.5}$	${}^{+ 0.4}_{+ 1.2}$	${}^{-2.0}_{+2.2}$	${}^{+0.0}_{-0.0}$	${}^{+3.5}_{-3.3}$	${}^{-5.7}_{+8.2}$	±1.7	±0.9	±1.3	±0.2	0.0	±1.6	${}^{+ 0.5}_{-0.5}$	±2.8
(4.57–6.00) e-02	$0.93 ^{+ 0.05}_{-0.04}$	1.213	6.3	${}^{-1.6}_{+1.6}$	${}^{+ 1.0}_{-4.6}$	${}^{-0.5}_{-3.4}$	${}^{-4.4}_{+1.9}$	${}^{-0.2}_{-2.0}$	${}^{+3.2}_{-3.4}$	${}^{+1.3}_{-3.7}$	${}^{+ 0.9}_{-2.5}$	${}^{-4.0}_{+1.3}$	${}^{-3.8}_{+2.1}$	${}^{-0.3}_{-0.2}$	${}^{-0.1}_{-0.8}$	${}^{-8.2}_{+6.6}$	∓2.7	±0.1	±3.6	±0.5	0.0	±1.4	${}^{+ 0.6}_{-0.5}$	±3.3
(6.00–7.43) e-02	$0.93 ^{+ 0.06}_{-0.04}$	0.923	20.	${}^{-1.0}_{+0.9}$	${}^{+6.9}_{-7.5}$	${}^{+1.5}_{-2.2}$	${}^{-7.0}_{+6.7}$	${}^{-3.2}_{+4.0}$	${}^{-0.9}_{-9.1}$	${}^{+1.5}_{-2.1}$	${}^{+0.4}_{-0.5}$	${}^{-0.9}_{+ 2.1}$	${}^{-5.1}_{+6.1}$	${}^{+ 0.2}_{+0.6}$	${}^{-0.9}_{+ 1.0}$	${}^{-5.9}_{+6.9}$	±7.5	±2.3	±3.2	±0.1	0.0	±1.4	${}^{+ 0.6}_{-0.5}$	±3.6
(7.43–8.86) e-02	$0.93 ^{+ 0.06}_{-0.05}$	3.288	69.	${}^{-0.6}_{+0.5}$	${}^{-8.9}_{-1.3}$	${}^{-9.1}_{+1.0}$	${}^{ -23}_{ +25}$	${}^{-18}_{ +11}$	${}^{ -15}_{ -30}$	${}^{-4.6}_{+8.1}$	${}^{-3.4}_{-2.4}$	${}^{ -16}_{ +21}$	${}^{ -18}_{ +17}$	${}^{-3.3}_{-0.1}$	${}^{-0.5}_{+0.8}$	${}^{ -17}_{ +20}$	∓15	∓1.3	±0.8	±0.1	0.0	±1.4	${}^{+ 0.6}_{-0.5}$	±8.3

**Table 24:** Measured jet cross section ratio in *x*_T_ bin for anti-*k*_*t*_ jets with *R*=0.4 in the rapidity bin 3.6≤|*y*|<4.4. See caption of Table 18 for details

*x*_T_	NPCorr	*ρ*	$\delta_{\mathrm{stat.}}$ %	*γ*_6_ %	*γ*_12_ %	*γ*_18_ %	*γ*_24_ %	*γ*_30_ %	*γ*_31_ %	*γ*_37_ %	*γ*_43_ %	*γ*_49_ %	*γ*_55_ %	*γ*_61_ %	*γ*_67_ %	*γ*_73_ %	*γ*_81_ %	*γ*_82_ %	*γ*_74_ %	*γ*_75_ %	*γ*_86_ %	*u*_1_ %	*u*_2_ %	*u*_3_ %
(1.71–2.29) e-02	$0.85 ^{+ 0.08}_{-0.04}$	1.144	8.0	${}^{+11.}_{-8.8}$	${}^{+9.3}_{-9.6}$	${}^{+4.3}_{-2.8}$	${}^{+1.6}_{-2.9}$	${}^{+ 0.9}_{-1.9}$	${}^{ +78}_{ -57}$	${}^{-1.3}_{+1.0}$	${}^{+3.0}_{-4.1}$	${}^{+ 0.4}_{-0.3}$	${}^{-1.1}_{+ 0.7}$	${}^{+0.1}_{-0.1}$	${}^{+4.8}_{-4.7}$	${}^{-4.7}_{+3.7}$	∓19	±0.7	±0.2	±2.2	±0.9	±2.2	${}^{+0.5}_{-0.5}$	±6.4
(2.29–3.14) e-02	$0.85 ^{+ 0.09}_{-0.06}$	1.245	7.1	${}^{+8.7}_{-9.2}$	${}^{+11.}_{-8.3}$	${}^{+6.2}_{-5.1}$	${}^{+1.9}_{-0.7}$	${}^{+ 1.1}_{-0.6}$	${}^{+144}_{ -54}$	${}^{-0.4}_{+ 0.9}$	${}^{+3.5}_{-3.1}$	${}^{+ 0.5}_{+ 0.4}$	${}^{-2.5}_{+3.7}$	${}^{+0.0}_{+ 1.3}$	${}^{+7.4}_{-8.5}$	${}^{-8.3}_{+12.}$	∓9.0	±5.1	±2.5	±4.9	0.0	±1.4	${}^{+ 0.5}_{-0.5}$	±7.7
(3.14–4.57) e-02	$0.85 ^{+ 0.09}_{-0.09}$	1.244	24.	${}^{+5.8}_{-4.5}$	${}^{+19.}_{-9.5}$	${}^{+19.}_{+2.8}$	${}^{ -13}_{+38}$	${}^{+2.2}_{+1.8}$	${}^{+159}_{ -63}$	${}^{-0.1}_{+ 3.0}$	${}^{+1.5}_{+1.1}$	${}^{-1.5}_{+9.6}$	${}^{-4.1}_{+9.1}$	${}^{-0.0}_{+0.0}$	${}^{+7.5}_{-5.2}$	${}^{ -14}_{ +17}$	∓8.8	±0.9	±9.0	±0.6	0.0	±1.4	${}^{+ 0.5}_{-0.5}$	±9.5

**Table 25:** Measured jet cross section ratio in *x*_T_ bin for anti-*k*_*t*_ jets with *R*=0.6 in the rapidity bin |*y*|<0.3. See caption of Table 18 for details

*x*_T_	NPCorr	*ρ*	$\delta_{\mathrm{stat.}}$ %	*γ*_1_ %	*γ*_7_ %	*γ*_13_ %	*γ*_19_ %	*γ*_25_ %	*γ*_31_ %	*γ*_32_ %	*γ*_38_ %	*γ*_44_ %	*γ*_50_ %	*γ*_56_ %	*γ*_62_ %	*γ*_68_ %	*γ*_76_ %	*γ*_82_ %	*γ*_74_ %	*γ*_75_ %	*γ*_83_ %	*u*_1_ %	*u*_2_ %	*u*_3_ %
(1.71–2.29) e-02	$1.07 ^{+ 0.02}_{-0.07}$	1.519	2.0	${}^{ +12}_{-11}$	${}^{+4.0}_{-5.6}$	${}^{ +12}_{ -11}$	${}^{+6.1}_{-7.1}$	${}^{+2.6}_{-2.6}$	0.0	${}^{-0.8}_{+ 0.3}$	${}^{+3.0}_{-3.6}$	${}^{+0.4}_{-1.4}$	${}^{-0.4}_{+ 0.3}$	${}^{-0.0}_{-0.1}$	${}^{-1.5}_{+ 0.7}$	${}^{+ 0.1}_{-0.2}$	∓5.0	±1.0	±0.1	±0.8	±0.9	±1.4	${}^{+ 0.6}_{-0.5}$	±0.3
(2.29–3.14) e-02	$1.04 ^{+ 0.03}_{-0.05}$	1.536	2.6	${}^{+8.0}_{-6.9}$	${}^{+3.6}_{-3.4}$	${}^{+7.8}_{-6.4}$	${}^{+3.7}_{-2.5}$	${}^{+ 0.8}_{-0.2}$	0.0	${}^{-0.2}_{+ 0.6}$	${}^{+2.5}_{-1.7}$	${}^{-0.1}_{+ 0.7}$	${}^{-0.7}_{+ 1.1}$	${}^{+ 0.1}_{+0.4}$	${}^{+1.5}_{-1.3}$	${}^{+ 0.5}_{-0.0}$	∓1.7	±0.4	±0.0	±0.7	0.0	±1.4	${}^{+ 0.5}_{-0.5}$	±0.6
(3.14–4.57) e-02	$1.01 ^{+ 0.02}_{-0.02}$	1.530	2.1	${}^{+2.5}_{-2.1}$	${}^{+4.6}_{-2.9}$	${}^{+3.2}_{-1.9}$	${}^{+1.4}_{-0.8}$	${}^{+ 0.6}_{-0.1}$	0.0	${}^{+ 0.3}_{+ 0.1}$	${}^{+1.7}_{-1.2}$	${}^{-0.1}_{+ 0.9}$	${}^{-1.4}_{+1.7}$	${}^{+ 0.1}_{+0.4}$	${}^{+5.4}_{-4.9}$	${}^{-1.1}_{+1.3}$	∓2.4	±0.0	±0.2	±0.1	0.0	±1.4	${}^{+ 0.5}_{-0.5}$	±0.4
(4.57–6.00) e-02	$0.99 ^{+ 0.01}_{-0.01}$	1.533	1.3	${}^{-1.1}_{+1.0}$	${}^{+2.0}_{-1.1}$	${}^{-3.3}_{+3.3}$	${}^{+2.0}_{-2.0}$	${}^{-0.5}_{+ 0.2}$	0.0	${}^{+ 0.7}_{-0.4}$	${}^{+0.8}_{-0.9}$	${}^{-1.0}_{+ 1.2}$	${}^{-2.1}_{+1.7}$	${}^{-0.3}_{-0.1}$	${}^{+5.1}_{-4.4}$	${}^{-4.7}_{+4.8}$	∓1.1	±0.3	±0.2	±0.1	0.0	±1.4	${}^{+ 0.5}_{-0.5}$	±0.4
(6.00–7.43) e-02	$0.99 ^{+ 0.01}_{-0.01}$	1.544	1.9	${}^{-0.6}_{+0.5}$	${}^{-0.1}_{+ 0.4}$	${}^{-2.6}_{+1.9}$	${}^{+2.8}_{-3.0}$	${}^{-0.2}_{+ 0.0}$	0.0	${}^{+ 0.8}_{-1.1}$	${}^{+ 0.8}_{-1.2}$	${}^{-0.5}_{+ 1.1}$	${}^{-1.5}_{+1.8}$	${}^{-0.1}_{+ 0.1}$	${}^{+2.1}_{-2.6}$	${}^{-5.6}_{+6.5}$	∓0.9	∓0.1	±0.2	±0.1	0.0	±1.4	${}^{+ 0.5}_{-0.5}$	±0.3
(7.43–8.86) e-02	$0.99 ^{+ 0.01}_{-0.01}$	1.532	2.6	${}^{-0.1}_{+0.1}$	${}^{+ 0.5}_{-1.2}$	${}^{+ 0.7}_{-0.3}$	${}^{+3.0}_{-2.7}$	${}^{-0.5}_{+ 0.4}$	0.0	${}^{+1.6}_{-1.6}$	${}^{+ 1.0}_{-1.0}$	${}^{-0.1}_{+ 0.5}$	${}^{-2.3}_{+2.0}$	${}^{-0.4}_{+ 0.1}$	${}^{+0.1}_{-0.3}$	${}^{-6.0}_{+6.5}$	∓0.3	∓0.0	±0.3	±0.3	0.0	±1.4	${}^{+ 0.5}_{-0.5}$	±0.3
(8.86–11.4) e-02	$1.00 ^{+ 0.01}_{-0.01}$	1.512	3.8	${}^{-0.0}_{+0.0}$	${}^{+2.6}_{-2.3}$	${}^{+ 0.8}_{-0.9}$	${}^{+2.7}_{-2.6}$	${}^{-1.2}_{+1.6}$	0.0	${}^{+1.4}_{-1.2}$	${}^{+ 0.4}_{-0.9}$	${}^{-0.0}_{+ 0.3}$	${}^{-2.1}_{+2.1}$	${}^{-0.6}_{+ 0.1}$	${}^{-0.2}_{+0.2}$	${}^{-4.0}_{+4.6}$	∓0.3	±0.1	±0.2	±0.2	0.0	±1.4	${}^{+ 0.5}_{-0.5}$	±0.3
(1.14–1.43) e-01	$1.00 ^{+ 0.01}_{-0.01}$	1.503	7.7	${}^{-0.0}_{+0.0}$	${}^{+4.1}_{-3.6}$	${}^{+2.6}_{-2.7}$	${}^{+2.1}_{-1.9}$	${}^{-1.2}_{+ 1.0}$	0.0	${}^{+1.5}_{-1.5}$	${}^{+ 0.6}_{-0.1}$	${}^{+0.5}_{+ 0.1}$	${}^{-2.3}_{+2.6}$	${}^{+ 0.2}_{+ 0.5}$	${}^{-0.1}_{+0.1}$	${}^{-2.4}_{+2.6}$	±0.1	±0.1	±0.4	±0.0	0.0	±1.4	${}^{+ 0.5}_{-0.5}$	±0.2
(1.43–1.71) e-01	$1.00 ^{+ 0.01}_{-0.01}$	1.553	14.	${}^{-0.0}_{+0.0}$	${}^{+5.8}_{-6.0}$	${}^{+2.7}_{-3.0}$	${}^{+ 0.3}_{-0.4}$	${}^{-1.4}_{+1.2}$	0.0	${}^{+ 0.9}_{-1.2}$	${}^{+ 0.1}_{-0.1}$	${}^{-0.1}_{-0.1}$	${}^{-2.8}_{+2.6}$	${}^{-0.4}_{+ 0.4}$	${}^{-0.0}_{+0.0}$	${}^{-2.7}_{+2.4}$	∓0.1	∓0.1	±0.4	±0.0	0.0	±1.4	${}^{+ 0.5}_{-0.5}$	±0.4
(1.71–2.29) e-01	$1.00 ^{+ 0.01}_{-0.01}$	1.525	25.	${}^{-0.0}_{+0.0}$	${}^{+3.1}_{-2.0}$	${}^{-2.1}_{+2.6}$	${}^{+ 0.9}_{-0.8}$	${}^{-1.2}_{+1.2}$	0.0	${}^{+1.7}_{-1.1}$	${}^{+ 0.3}_{-0.3}$	${}^{-0.4}_{+ 0.7}$	${}^{-2.0}_{+2.0}$	${}^{-0.6}_{+ 0.6}$	${}^{-0.0}_{+0.0}$	${}^{-1.5}_{+2.4}$	∓0.6	∓0.0	±0.3	±0.0	0.0	±1.4	${}^{+ 0.5}_{-0.6}$	±0.2
(2.29–2.86) e-01	$1.01 ^{+ 0.01}_{-0.01}$	1.978	55.	${}^{-0.2}_{+0.2}$	${}^{+ 0.1}_{-0.3}$	${}^{-2.5}_{+2.4}$	${}^{+ 0.1}_{-0.8}$	${}^{+1.4}_{-1.7}$	0.0	${}^{+1.3}_{-1.7}$	${}^{-0.1}_{-0.6}$	${}^{+0.1}_{-0.5}$	${}^{-1.5}_{+1.1}$	${}^{-1.7}_{+1.7}$	${}^{-0.0}_{+ 0.0}$	${}^{-2.4}_{+1.9}$	±0.3	∓0.0	±0.8	±0.0	0.0	±1.4	${}^{+ 0.5}_{-0.6}$	±0.2
(2.86–3.43) e-01	$1.01 ^{+ 0.01}_{-0.01}$	3.484	123	${}^{+2.1}_{-2.1}$	${}^{+2.0}_{-2.0}$	${}^{-1.1}_{+1.4}$	${}^{+1.5}_{-2.1}$	${}^{+ 0.8}_{-0.8}$	0.0	${}^{+1.0}_{-1.5}$	${}^{+0.7}_{-1.2}$	${}^{-0.4}_{+ 0.5}$	${}^{-3.0}_{+3.6}$	${}^{-4.2}_{+4.7}$	${}^{+ 0.3}_{-0.3}$	${}^{-6.9}_{+6.9}$	∓1.2	∓0.1	±2.4	±0.0	0.0	±1.4	${}^{+ 0.5}_{-0.6}$	±0.1

**Table 26:** Measured jet cross section ratio in *x*_T_ bin for anti-*k*_*t*_ jets with *R*=0.6 in the rapidity bin 0.3≤|*y*|<0.8. See caption of Table 18 for details

*x*_T_	NPCorr	*ρ*	$\delta_{\mathrm{stat.}}$ %	*γ*_1_ %	*γ*_7_ %	*γ*_13_ %	*γ*_19_ %	*γ*_25_ %	*γ*_31_ %	*γ*_32_ %	*γ*_38_ %	*γ*_44_ %	*γ*_50_ %	*γ*_56_ %	*γ*_62_ %	*γ*_68_ %	*γ*_76_ %	*γ*_82_ %	*γ*_74_ %	*γ*_75_ %	*γ*_83_ %	*u*_1_ %	*u*_2_ %	*u*_3_ %
(1.71–2.29) e-02	$1.08 ^{+ 0.02}_{-0.08}$	1.523	1.6	${}^{ +13}_{-11}$	${}^{+4.2}_{-4.6}$	${}^{+8.3}_{-7.1}$	${}^{+7.0}_{-6.7}$	${}^{+1.2}_{-1.0}$	${}^{+ 0.3}_{-0.2}$	${}^{-0.2}_{+ 0.1}$	${}^{+2.9}_{-2.9}$	${}^{+ 0.6}_{-1.0}$	${}^{-0.5}_{+ 0.2}$	${}^{-0.0}_{-0.1}$	${}^{+2.6}_{-2.8}$	${}^{-1.7}_{+1.5}$	∓5.0	±0.2	±0.1	±0.4	±0.9	±1.4	${}^{+0.5}_{-0.5}$	±0.3
(2.29–3.14) e-02	$1.05 ^{+ 0.02}_{-0.05}$	1.500	1.9	${}^{+8.0}_{-7.4}$	${}^{+4.8}_{-4.0}$	${}^{+6.8}_{-5.9}$	${}^{+5.0}_{-4.2}$	${}^{+ 0.3}_{+ 0.2}$	${}^{+ 0.2}_{-0.1}$	${}^{+0.2}_{+ 0.2}$	${}^{+2.2}_{-1.8}$	${}^{+ 0.6}_{+ 0.3}$	${}^{-0.9}_{+1.5}$	${}^{+ 0.1}_{+ 0.4}$	${}^{+3.9}_{-3.9}$	${}^{-3.6}_{+4.5}$	∓2.8	±0.1	±0.1	±0.3	0.0	±1.4	${}^{+0.5}_{-0.5}$	±0.5
(3.14–4.57) e-02	$1.01 ^{+ 0.02}_{-0.03}$	1.507	1.7	${}^{+2.2}_{-2.3}$	${}^{+3.8}_{-3.7}$	${}^{+5.7}_{-5.1}$	${}^{+1.9}_{-1.7}$	${}^{+1.1}_{-1.5}$	${}^{+ 0.1}_{-0.1}$	${}^{+0.6}_{-0.9}$	${}^{+1.1}_{-1.5}$	${}^{-0.2}_{+ 0.0}$	${}^{-1.5}_{+1.2}$	${}^{+ 0.1}_{-0.0}$	${}^{+2.9}_{-3.0}$	${}^{-4.6}_{+5.0}$	∓0.4	∓0.0	±0.2	±0.1	0.0	±1.4	${}^{+ 0.5}_{-0.5}$	±0.5
(4.57–6.00) e-02	$1.00 ^{+ 0.01}_{-0.02}$	1.538	1.0	${}^{-1.1}_{+1.1}$	${}^{+2.2}_{-2.8}$	${}^{+ 0.9}_{-0.7}$	${}^{+0.7}_{-1.1}$	${}^{+2.1}_{-2.5}$	${}^{+ 0.1}_{-0.1}$	${}^{+ 0.6}_{-1.0}$	${}^{+ 0.5}_{-0.9}$	${}^{-1.2}_{+ 0.9}$	${}^{-2.1}_{+1.5}$	${}^{-0.4}_{-0.2}$	${}^{+ 0.4}_{-0.4}$	${}^{-4.3}_{+3.7}$	∓0.2	∓0.1	±0.4	±0.1	0.0	±1.4	${}^{+ 0.5}_{-0.5}$	±0.5
(6.00–7.43) e-02	$1.00 ^{+ 0.01}_{-0.01}$	1.553	1.4	${}^{-0.6}_{+0.5}$	${}^{+3.0}_{-2.4}$	${}^{-0.0}_{+ 0.3}$	${}^{+1.4}_{-1.1}$	${}^{+0.2}_{+ 0.2}$	${}^{+ 0.1}_{-0.0}$	${}^{+ 1.0}_{-1.2}$	${}^{+0.8}_{-0.4}$	${}^{-1.2}_{+1.4}$	${}^{-1.9}_{+2.4}$	${}^{+ 0.1}_{+ 0.4}$	${}^{-0.4}_{+ 0.4}$	${}^{-3.6}_{+3.6}$	∓0.2	±0.0	±0.6	±0.1	0.0	±1.4	${}^{+ 0.5}_{-0.5}$	±0.4
(7.43–8.86) e-02	$1.00 ^{+ 0.01}_{-0.01}$	1.542	2.1	${}^{-0.1}_{+0.1}$	${}^{+1.8}_{-2.8}$	${}^{+ 0.8}_{-1.2}$	${}^{+1.2}_{-1.1}$	${}^{-2.4}_{+2.4}$	${}^{+ 0.0}_{-0.1}$	${}^{+ 0.8}_{-1.3}$	${}^{+0.2}_{-0.5}$	${}^{-0.9}_{+ 1.0}$	${}^{-1.7}_{+2.0}$	${}^{-0.2}_{+ 0.0}$	${}^{-0.2}_{+ 0.2}$	${}^{-2.7}_{+3.1}$	∓0.4	±0.0	±0.5	±0.1	0.0	±1.4	${}^{+ 0.5}_{-0.5}$	±0.3
(8.86–11.4) e-02	$1.00 ^{+ 0.01}_{-0.01}$	1.482	3.1	${}^{-0.0}_{+0.0}$	${}^{+2.1}_{-2.4}$	${}^{-0.3}_{+ 0.3}$	${}^{+1.6}_{-2.0}$	${}^{-2.5}_{+1.8}$	${}^{+ 0.1}_{-0.1}$	${}^{+1.0}_{-1.4}$	${}^{+0.5}_{-0.6}$	${}^{-1.0}_{+ 0.1}$	${}^{-2.2}_{+2.0}$	${}^{-0.4}_{-0.2}$	${}^{-0.0}_{+ 0.0}$	${}^{-3.0}_{+2.5}$	∓0.2	±0.1	±0.5	±0.2	0.0	±1.4	${}^{+ 0.5}_{-0.5}$	±0.3
(1.14–1.43) e-01	$1.00 ^{+ 0.01}_{-0.01}$	1.483	6.4	${}^{-0.0}_{+0.0}$	${}^{-0.6}_{+ 1.0}$	${}^{-0.8}_{+ 0.6}$	${}^{+2.1}_{-1.8}$	${}^{-0.3}_{+ 0.6}$	${}^{+ 0.0}_{-0.0}$	${}^{+1.4}_{-1.1}$	${}^{+0.4}_{-0.4}$	${}^{-0.2}_{+ 0.0}$	${}^{-1.7}_{+2.0}$	${}^{+ 0.4}_{+0.1}$	${}^{-0.0}_{+ 0.0}$	${}^{-2.8}_{+2.1}$	±0.1	±0.1	±0.2	±0.2	0.0	±1.4	${}^{+ 0.5}_{-0.5}$	±0.2
(1.43–1.71) e-01	$1.01 ^{+ 0.01}_{-0.01}$	1.340	14.	${}^{-0.0}_{+0.0}$	${}^{-1.0}_{+ 0.3}$	${}^{+2.2}_{-2.6}$	${}^{+ 0.9}_{-1.0}$	${}^{+0.5}_{-0.3}$	${}^{+ 0.0}_{-0.0}$	${}^{+1.1}_{-1.5}$	${}^{+ 0.3}_{-0.3}$	${}^{+ 0.5}_{-0.1}$	${}^{-1.4}_{+1.3}$	${}^{-0.5}_{+ 0.2}$	${}^{-0.0}_{+ 0.0}$	${}^{-1.7}_{+1.8}$	∓0.4	±0.0	±0.3	±0.0	0.0	±1.4	${}^{+ 0.5}_{-0.6}$	±0.3
(1.71–2.29) e-01	$1.01 ^{+ 0.01}_{-0.01}$	1.025	24.	${}^{-0.0}_{+0.0}$	${}^{+3.4}_{-3.8}$	${}^{+2.2}_{-2.8}$	${}^{+1.1}_{-1.4}$	${}^{-0.1}_{-0.8}$	${}^{+ 0.0}_{-0.0}$	${}^{+1.1}_{-1.2}$	${}^{-0.0}_{-0.1}$	${}^{+ 0.9}_{-1.1}$	${}^{-2.1}_{+1.3}$	${}^{-0.8}_{+1.2}$	${}^{-0.0}_{+ 0.0}$	${}^{-2.2}_{+1.6}$	±0.2	∓0.0	±0.3	±0.0	0.0	±1.4	${}^{+ 0.5}_{-0.6}$	±0.2
(2.29–2.86) e-01	$1.01 ^{+ 0.01}_{-0.01}$	2.883	47.	${}^{-0.2}_{+0.2}$	${}^{+ 0.2}_{-0.5}$	${}^{+2.6}_{-2.9}$	${}^{-0.1}_{-0.3}$	${}^{+0.4}_{-0.4}$	${}^{+ 0.0}_{-0.1}$	${}^{+1.2}_{-1.4}$	${}^{+ 0.1}_{-0.5}$	${}^{+ 0.4}_{-1.6}$	${}^{-0.9}_{+ 0.9}$	${}^{-2.7}_{+3.0}$	${}^{-0.0}_{+ 0.0}$	${}^{-2.0}_{+1.5}$	±0.8	±0.0	±1.0	±0.0	0.0	±1.4	${}^{+ 0.5}_{-0.6}$	±0.2
(2.86–3.43) e-01	$1.01 ^{+ 0.01}_{-0.01}$	6.537	68.	${}^{+2.0}_{-2.0}$	${}^{+ 0.5}_{-1.6}$	${}^{+4.5}_{-4.4}$	${}^{+0.8}_{-1.2}$	${}^{-0.5}_{+ 0.1}$	${}^{+ 0.0}_{-0.0}$	${}^{+1.1}_{-1.2}$	${}^{+ 0.3}_{-0.7}$	${}^{-1.8}_{+ 0.8}$	${}^{-2.9}_{+2.8}$	${}^{-6.0}_{+6.4}$	${}^{+ 0.5}_{-0.4}$	${}^{-6.3}_{+7.3}$	∓0.0	±0.0	±4.2	±0.0	0.0	±1.4	${}^{+ 0.5}_{-0.6}$	±0.1

**Table 27:** Measured jet cross section ratio in *x*_T_ bin for anti-*k*_*t*_ jets with *R*=0.6 in the rapidity bin 0.8≤|*y*|<1.2. See caption of Table 18 for details

*x*_T_	NPCorr	*ρ*	$\delta_{\mathrm{stat.}}$ %	*γ*_2_ %	*γ*_8_ %	*γ*_14_ %	*γ*_20_ %	*γ*_26_ %	*γ*_31_ %	*γ*_33_ %	*γ*_39_ %	*γ*_45_ %	*γ*_51_ %	*γ*_57_ %	*γ*_63_ %	*γ*_69_ %	*γ*_77_ %	*γ*_82_ %	*γ*_74_ %	*γ*_75_ %	*γ*_83_ %	*u*_1_ %	*u*_2_ %	*u*_3_ %
(1.71–2.29) e-02	$1.09 ^{+ 0.02}_{-0.07}$	1.499	1.9	${}^{ +13}_{-11}$	${}^{+4.1}_{-3.9}$	${}^{+8.4}_{-6.8}$	${}^{+5.8}_{-4.5}$	${}^{+1.0}_{-1.1}$	${}^{+2.4}_{-2.7}$	${}^{-0.3}_{+ 0.4}$	${}^{+3.0}_{-2.8}$	${}^{+ 0.3}_{-0.7}$	${}^{-0.4}_{+ 0.3}$	${}^{-0.0}_{-0.1}$	${}^{+2.8}_{-2.5}$	${}^{-1.8}_{+1.8}$	∓12	∓0.2	±0.1	±0.3	±0.9	±1.4	${}^{+ 0.6}_{-0.5}$	±0.6
(2.29–3.14) e-02	$1.05 ^{+ 0.02}_{-0.05}$	1.433	2.3	${}^{+7.8}_{-6.5}$	${}^{+4.8}_{-3.7}$	${}^{+6.9}_{-5.5}$	${}^{+3.6}_{-2.5}$	${}^{+ 0.2}_{+ 0.6}$	${}^{+2.6}_{-1.7}$	${}^{+ 0.2}_{+0.6}$	${}^{+2.2}_{-1.4}$	${}^{+ 0.3}_{+ 0.6}$	${}^{-1.0}_{+ 1.4}$	${}^{+ 0.1}_{+ 0.4}$	${}^{+4.0}_{-3.5}$	${}^{-3.8}_{+4.4}$	∓5.1	∓0.1	±0.1	±0.4	0.0	±1.4	${}^{+ 0.5}_{-0.5}$	±0.8
(3.14–4.57) e-02	$1.02 ^{+ 0.02}_{-0.02}$	1.505	2.0	${}^{+2.4}_{-2.1}$	${}^{+3.4}_{-3.3}$	${}^{+5.4}_{-4.5}$	${}^{+2.0}_{-1.4}$	${}^{+1.3}_{-1.2}$	${}^{+2.0}_{-1.7}$	${}^{+0.8}_{-0.6}$	${}^{+1.3}_{-1.2}$	${}^{-0.4}_{+ 0.2}$	${}^{-1.5}_{+1.7}$	${}^{+ 0.1}_{-0.0}$	${}^{+3.2}_{-2.9}$	${}^{-4.8}_{+5.3}$	±0.5	±0.0	±0.2	±0.2	0.0	±1.4	${}^{+ 0.5}_{-0.5}$	±0.7
(4.57–6.00) e-02	$1.00 ^{+ 0.01}_{-0.01}$	1.515	1.2	${}^{-1.1}_{+1.0}$	${}^{+2.8}_{-3.0}$	${}^{+1.1}_{-1.1}$	${}^{-0.7}_{+0.1}$	${}^{+2.3}_{-2.8}$	${}^{+ 1.0}_{-0.9}$	${}^{+ 0.8}_{-1.2}$	${}^{+0.7}_{-0.9}$	${}^{-0.9}_{+ 0.8}$	${}^{-2.0}_{+1.5}$	${}^{-0.4}_{-0.1}$	${}^{+ 0.4}_{-0.3}$	${}^{-4.0}_{+3.9}$	∓0.9	±0.3	±0.6	±0.0	0.0	±1.4	${}^{+ 0.5}_{-0.5}$	±0.8
(6.00–7.43) e-02	$1.00 ^{+ 0.01}_{-0.01}$	1.595	1.8	${}^{-0.6}_{+0.6}$	${}^{+3.3}_{-2.6}$	${}^{+ 0.0}_{+ 0.1}$	${}^{-0.6}_{+ 1.0}$	${}^{+ 0.1}_{+ 0.5}$	${}^{+ 0.1}_{+ 0.0}$	${}^{+ 1.2}_{-1.0}$	${}^{+0.9}_{-0.3}$	${}^{-0.9}_{+ 1.3}$	${}^{-1.8}_{+2.2}$	${}^{+ 0.1}_{+0.4}$	${}^{-0.4}_{+ 0.4}$	${}^{-3.1}_{+3.6}$	∓0.4	±0.3	±0.8	±0.0	0.0	±1.4	${}^{+ 0.5}_{-0.5}$	±0.6
(7.43–8.86) e-02	$1.00 ^{+ 0.01}_{-0.01}$	1.546	2.5	${}^{-0.1}_{+0.1}$	${}^{+1.7}_{-2.2}$	${}^{+ 0.8}_{-0.7}$	${}^{-0.0}_{-0.3}$	${}^{-2.2}_{+2.4}$	${}^{-0.7}_{+ 0.4}$	${}^{+ 1.1}_{-0.9}$	${}^{+0.2}_{-0.7}$	${}^{-1.1}_{+1.3}$	${}^{-2.0}_{+2.1}$	${}^{-0.2}_{-0.0}$	${}^{-0.2}_{+ 0.2}$	${}^{-3.3}_{+3.1}$	∓0.3	±0.3	±0.8	±0.1	0.0	±1.4	${}^{+ 0.5}_{-0.5}$	±0.5
(8.86–11.4) e-02	$1.00 ^{+ 0.01}_{-0.01}$	1.510	3.8	${}^{-0.0}_{+0.0}$	${}^{+1.8}_{-2.4}$	${}^{-0.3}_{+ 0.3}$	${}^{+1.4}_{-1.2}$	${}^{-2.4}_{+1.9}$	${}^{-0.2}_{+ 0.1}$	${}^{+1.1}_{-1.5}$	${}^{+0.9}_{-0.6}$	${}^{-1.0}_{-0.0}$	${}^{-2.2}_{+2.1}$	${}^{-0.3}_{-0.2}$	${}^{-0.0}_{+ 0.0}$	${}^{-3.0}_{+2.1}$	∓0.3	±0.1	±1.0	±0.1	0.0	±1.4	${}^{+ 0.5}_{-0.5}$	±0.5
(1.14–1.43) e-01	$1.01 ^{+ 0.01}_{-0.01}$	1.432	8.1	${}^{-0.0}_{+0.0}$	${}^{+ 0.3}_{+ 0.9}$	${}^{+ 0.2}_{-0.0}$	${}^{+2.1}_{-1.2}$	${}^{+ 0.2}_{+ 0.4}$	${}^{+ 0.5}_{-0.4}$	${}^{+2.1}_{-1.4}$	${}^{+0.6}_{-0.4}$	${}^{+ 1.3}_{+ 0.4}$	${}^{-0.8}_{+ 1.7}$	${}^{+ 0.4}_{+0.1}$	${}^{-0.0}_{+ 0.0}$	${}^{-0.8}_{+ 2.5}$	∓2.6	±0.1	±0.2	±0.3	0.0	±1.4	${}^{+ 0.5}_{-0.5}$	±0.4
(1.43–1.71) e-01	$1.01 ^{+ 0.01}_{-0.01}$	1.355	16.	${}^{-0.0}_{+0.0}$	${}^{-0.9}_{+ 0.5}$	${}^{+2.5}_{-2.1}$	${}^{+ 0.9}_{-1.1}$	${}^{+0.4}_{+ 0.1}$	${}^{+ 0.2}_{+ 0.3}$	${}^{+1.2}_{-1.1}$	${}^{+0.2}_{-0.2}$	${}^{+ 0.6}_{+ 0.3}$	${}^{-1.5}_{+2.0}$	${}^{-0.5}_{+0.4}$	${}^{-0.0}_{+ 0.0}$	${}^{-1.2}_{+2.7}$	∓1.0	∓0.1	±0.4	±0.3	0.0	±1.4	${}^{+ 0.5}_{-0.7}$	±0.4
(1.71–2.29) e-01	$1.01 ^{+ 0.01}_{-0.01}$	1.533	27.	${}^{-0.0}_{+0.0}$	${}^{+3.8}_{-4.5}$	${}^{+2.5}_{-3.5}$	${}^{+2.4}_{-2.4}$	${}^{+0.2}_{-1.4}$	${}^{-0.2}_{-1.2}$	${}^{+1.4}_{-1.3}$	${}^{+ 0.1}_{-0.5}$	${}^{+ 0.3}_{-2.2}$	${}^{-2.4}_{+ 0.6}$	${}^{-0.7}_{+ 0.9}$	${}^{-0.0}_{+ 0.0}$	${}^{-3.4}_{+1.3}$	±0.2	±0.5	±0.3	±0.0	0.0	±1.4	${}^{+ 0.7}_{-0.7}$	±0.3
(2.29–2.86) e-01	$1.01 ^{+ 0.01}_{-0.01}$	1.213	130	${}^{-0.3}_{+0.4}$	${}^{+ 0.6}_{-0.7}$	${}^{+3.2}_{-3.6}$	${}^{+ 0.8}_{-1.2}$	${}^{+0.2}_{-0.6}$	${}^{+ 0.8}_{-0.4}$	${}^{+1.4}_{-2.4}$	${}^{+ 0.0}_{-0.5}$	${}^{+1.4}_{-1.8}$	${}^{-0.4}_{+ 0.8}$	${}^{-3.8}_{+3.6}$	${}^{-0.1}_{+0.1}$	${}^{-0.9}_{+ 1.6}$	∓1.6	±0.5	±2.7	±0.0	0.0	±1.4	${}^{+ 1.4}_{-0.7}$	±0.3

**Table 28:** Measured jet cross section ratio in *x*_T_ bin for anti-*k*_*t*_ jets with *R*=0.6 in the rapidity bin 1.2≤|*y*|<2.1. See caption of Table 18 for details

*x*_T_	NPCorr	*ρ*	$\delta_{\mathrm{stat.}}$ %	*γ*_3_ %	*γ*_9_ %	*γ*_15_ %	*γ*_21_ %	*γ*_27_ %	*γ*_31_ %	*γ*_34_ %	*γ*_40_ %	*γ*_46_ %	*γ*_52_ %	*γ*_58_ %	*γ*_64_ %	*γ*_70_ %	*γ*_78_ %	*γ*_82_ %	*γ*_74_ %	*γ*_75_ %	*γ*_83_ %	*u*_1_ %	*u*_2_ %	*u*_3_ %
(1.71–2.29) e-02	$1.09 ^{+ 0.02}_{-0.06}$	1.522	1.5	${}^{ +13}_{-11}$	${}^{+4.0}_{-4.2}$	${}^{+8.6}_{-7.0}$	${}^{+6.0}_{-4.6}$	${}^{+0.9}_{-1.0}$	${}^{ +11}_{ -11}$	${}^{-0.6}_{+ 0.2}$	${}^{+2.8}_{-2.8}$	${}^{+ 0.3}_{-0.4}$	${}^{-0.4}_{+ 0.3}$	${}^{-0.0}_{-0.1}$	${}^{+2.6}_{-2.6}$	${}^{-1.9}_{+1.8}$	∓12	±0.9	±0.1	±0.3	±0.9	±1.4	${}^{+ 0.5}_{-0.5}$	±0.6
(2.29–3.14) e-02	$1.05 ^{+ 0.03}_{-0.04}$	1.517	2.0	${}^{+8.5}_{-7.1}$	${}^{+5.1}_{-3.8}$	${}^{+7.3}_{-5.6}$	${}^{+4.2}_{-3.0}$	${}^{+ 0.4}_{+ 0.4}$	${}^{+10.}_{-8.6}$	${}^{+ 0.4}_{+0.4}$	${}^{+2.4}_{-1.6}$	${}^{+ 0.7}_{+ 0.4}$	${}^{-1.0}_{+1.6}$	${}^{+0.1}_{+ 0.4}$	${}^{+4.2}_{-3.9}$	${}^{-3.8}_{+4.6}$	∓5.0	±1.0	±0.1	±0.4	0.0	±1.4	${}^{+ 0.5}_{-0.5}$	±0.4
(3.14–4.57) e-02	$1.02 ^{+ 0.02}_{-0.02}$	1.413	1.7	${}^{+2.5}_{-2.4}$	${}^{+3.9}_{-3.4}$	${}^{+5.9}_{-4.9}$	${}^{+3.0}_{-3.2}$	${}^{+1.3}_{-1.4}$	${}^{+5.9}_{-5.0}$	${}^{+0.8}_{-0.8}$	${}^{+1.4}_{-1.5}$	${}^{-0.5}_{+ 0.2}$	${}^{-1.5}_{+1.6}$	${}^{+ 0.1}_{-0.0}$	${}^{+3.1}_{-3.0}$	${}^{-4.7}_{+5.4}$	∓0.4	±0.0	±0.2	±0.1	0.0	±1.4	${}^{+ 0.5}_{-0.5}$	±0.6
(4.57–6.00) e-02	$1.01 ^{+ 0.01}_{-0.01}$	1.436	1.1	${}^{-1.1}_{+1.1}$	${}^{+2.2}_{-2.8}$	${}^{+ 0.6}_{-0.9}$	${}^{+0.4}_{-0.6}$	${}^{+1.8}_{-2.6}$	${}^{+ 0.6}_{-1.4}$	${}^{+ 0.3}_{-1.0}$	${}^{+ 0.3}_{-0.9}$	${}^{-1.4}_{+1.2}$	${}^{-2.2}_{+1.6}$	${}^{-0.4}_{-0.2}$	${}^{+ 0.4}_{-0.4}$	${}^{-4.6}_{+4.1}$	∓0.5	∓0.1	±0.5	±0.0	0.0	±1.4	${}^{+ 0.5}_{-0.5}$	±0.6
(6.00–7.43) e-02	$1.01 ^{+ 0.01}_{-0.01}$	1.485	1.5	${}^{-0.6}_{+0.5}$	${}^{+3.9}_{-2.6}$	${}^{+ 0.3}_{+ 0.2}$	${}^{+ 0.9}_{-0.2}$	${}^{+ 0.4}_{+ 0.4}$	${}^{+ 0.4}_{+ 0.4}$	${}^{+1.3}_{-1.2}$	${}^{+1.0}_{-0.4}$	${}^{-0.4}_{+ 1.4}$	${}^{-1.8}_{+2.3}$	${}^{+ 0.2}_{+0.4}$	${}^{-0.4}_{+ 0.4}$	${}^{-3.2}_{+3.6}$	∓1.3	∓0.1	±0.5	±0.2	0.0	±1.4	${}^{+ 0.5}_{-0.5}$	±0.7
(7.43–8.86) e-02	$1.01 ^{+ 0.01}_{-0.01}$	1.554	2.5	${}^{-0.1}_{+0.1}$	${}^{+2.1}_{-2.9}$	${}^{+1.1}_{-1.4}$	${}^{+1.0}_{-1.4}$	${}^{-2.4}_{+2.4}$	${}^{-0.5}_{+ 0.0}$	${}^{+1.0}_{-1.5}$	${}^{+0.4}_{-0.9}$	${}^{-0.8}_{+ 1.1}$	${}^{-1.8}_{+1.9}$	${}^{-0.2}_{-0.0}$	${}^{-0.2}_{+ 0.1}$	${}^{-2.8}_{+3.3}$	∓1.3	±0.0	±0.5	±0.1	0.0	±1.4	${}^{+ 0.5}_{-0.5}$	±0.5
(8.86–11.4) e-02	$1.01 ^{+ 0.01}_{-0.01}$	1.460	3.6	${}^{-0.0}_{+0.0}$	${}^{+2.9}_{-2.9}$	${}^{+ 0.1}_{-0.2}$	${}^{+ 0.9}_{-1.5}$	${}^{-2.4}_{+1.6}$	${}^{+ 0.3}_{-0.9}$	${}^{+1.6}_{-2.1}$	${}^{+1.0}_{-0.9}$	${}^{+ 0.0}_{-0.4}$	${}^{-1.9}_{+2.0}$	${}^{-0.4}_{-0.2}$	${}^{-0.0}_{+ 0.0}$	${}^{-2.0}_{+2.4}$	∓0.0	±0.2	±0.3	±0.1	0.0	±1.4	${}^{+ 0.5}_{-0.5}$	±0.5
(1.14–1.43) e-01	$1.01 ^{+ 0.01}_{-0.01}$	1.792	7.7	${}^{-0.0}_{+0.0}$	${}^{-0.7}_{+ 0.9}$	${}^{-0.6}_{+ 0.5}$	${}^{-0.6}_{+ 1.2}$	${}^{-0.1}_{+ 0.8}$	${}^{-0.4}_{-0.1}$	${}^{+1.7}_{-1.2}$	${}^{+0.3}_{-0.6}$	${}^{-0.5}_{-0.4}$	${}^{-2.1}_{+2.3}$	${}^{+ 0.4}_{+ 0.1}$	${}^{-0.0}_{+ 0.0}$	${}^{-2.9}_{+1.6}$	∓0.1	±0.2	±0.6	±0.1	0.0	±1.4	${}^{+ 0.5}_{-0.5}$	±0.5
(1.43–1.71) e-01	$1.02 ^{+ 0.01}_{-0.01}$	1.840	17.	${}^{-0.0}_{+0.0}$	${}^{-0.5}_{-0.5}$	${}^{+3.2}_{-3.4}$	${}^{-1.8}_{+1.1}$	${}^{+0.5}_{-0.8}$	${}^{-0.1}_{-0.8}$	${}^{+1.4}_{-1.7}$	${}^{+ 0.3}_{-0.4}$	${}^{+ 1.7}_{-0.6}$	${}^{-1.5}_{+1.2}$	${}^{-0.6}_{+ 0.6}$	${}^{-0.0}_{+ 0.0}$	${}^{-0.7}_{+ 2.1}$	∓2.9	∓0.1	±1.0	±0.0	0.0	±1.4	${}^{+ 0.6}_{-1.0}$	±0.5
(1.71–2.29) e-01	$1.02 ^{+ 0.02}_{-0.01}$	0.780	46.	${}^{-0.0}_{+0.0}$	${}^{+5.4}_{-4.0}$	${}^{+4.0}_{-3.2}$	${}^{+ 0.6}_{+ 0.3}$	${}^{+0.5}_{-0.3}$	${}^{-0.3}_{+ 1.2}$	${}^{+2.0}_{-1.1}$	${}^{+ 0.4}_{+0.0}$	${}^{+ 0.8}_{-0.4}$	${}^{-3.3}_{+3.5}$	${}^{-1.4}_{+1.7}$	${}^{-0.0}_{+ 0.0}$	${}^{-3.3}_{+3.6}$	±0.3	∓0.0	±0.7	±0.0	0.0	±1.4	${}^{+ 0.6}_{-1.1}$	±0.4
(2.29–2.86) e-01	$1.02 ^{+ 0.02}_{-0.01}$	2.500	125	${}^{-0.3}_{+0.2}$	${}^{+ 0.8}_{+ 0.2}$	${}^{+3.4}_{-4.0}$	${}^{+1.1}_{-1.9}$	${}^{-0.5}_{-0.7}$	${}^{+ 0.8}_{+ 0.4}$	${}^{+1.3}_{-2.3}$	${}^{-0.1}_{-0.7}$	${}^{+ 1.6}_{-0.9}$	${}^{-0.1}_{+ 2.3}$	${}^{-5.3}_{+5.2}$	${}^{-0.1}_{+ 0.1}$	${}^{-3.0}_{+4.6}$	∓1.3	±0.5	±2.2	±0.0	0.0	±1.4	${}^{+ 0.5}_{-1.3}$	±0.5

**Table 29:** Measured jet cross section ratio in *x*_T_ bin for anti-*k*_*t*_ jets with *R*=0.6 in the rapidity bin 2.1≤|*y*|<2.8. See caption of Table 18 for details

*x*_T_	NPCorr	*ρ*	$\delta_{\mathrm{stat.}}$ %	*γ*_4_ %	*γ*_10_ %	*γ*_16_ %	*γ*_22_ %	*γ*_28_ %	*γ*_31_ %	*γ*_35_ %	*γ*_41_ %	*γ*_47_ %	*γ*_53_ %	*γ*_59_ %	*γ*_65_ %	*γ*_71_ %	*γ*_79_ %	*γ*_82_ %	*γ*_74_ %	*γ*_75_ %	*γ*_84_ %	*u*_1_ %	*u*_2_ %	*u*_3_ %
(1.71–2.29) e-02	$1.07 ^{+ 0.05}_{-0.05}$	1.502	2.1	${}^{ +13}_{-11}$	${}^{+3.3}_{-3.8}$	${}^{+8.1}_{-6.6}$	${}^{+3.3}_{-4.1}$	${}^{+0.3}_{-1.0}$	${}^{ +17}_{ -17}$	${}^{-1.0}_{+ 0.4}$	${}^{+2.0}_{-2.5}$	${}^{-0.3}_{-0.4}$	${}^{-0.6}_{+ 0.3}$	${}^{-0.0}_{-0.1}$	${}^{+1.9}_{-2.4}$	${}^{-2.1}_{+1.6}$	∓15	±0.5	±0.2	±0.3	±0.9	±1.4	${}^{+ 0.5}_{-0.5}$	±0.7
(2.29–3.14) e-02	$1.04 ^{+ 0.04}_{-0.02}$	1.462	1.5	${}^{+8.1}_{-7.1}$	${}^{+4.3}_{-4.4}$	${}^{+6.4}_{-6.3}$	${}^{+5.0}_{-4.6}$	${}^{+ 0.0}_{+ 0.4}$	${}^{ +17}_{ -14}$	${}^{-0.3}_{+0.5}$	${}^{+2.1}_{-1.7}$	${}^{-0.2}_{+ 0.4}$	${}^{-1.1}_{+1.4}$	${}^{+0.0}_{+ 0.3}$	${}^{+3.9}_{-3.7}$	${}^{-4.3}_{+4.4}$	∓2.1	±0.5	±0.2	±0.2	0.0	±1.4	${}^{+ 0.5}_{-0.5}$	±1.0
(3.14–4.57) e-02	$1.02 ^{+ 0.03}_{-0.01}$	1.421	2.2	${}^{+2.7}_{-2.4}$	${}^{+4.3}_{-3.8}$	${}^{+6.5}_{-5.4}$	${}^{+4.0}_{-3.7}$	${}^{+1.5}_{-1.4}$	${}^{+9.1}_{-8.2}$	${}^{+0.9}_{-0.5}$	${}^{+1.6}_{-1.4}$	${}^{-0.4}_{+ 0.3}$	${}^{-1.7}_{+1.8}$	${}^{+ 0.1}_{-0.0}$	${}^{+3.3}_{-3.0}$	${}^{-5.1}_{+5.9}$	∓0.8	±0.2	±0.4	±0.0	0.0	±1.4	${}^{+ 0.5}_{-0.5}$	±0.8
(4.57–6.00) e-02	$1.01 ^{+ 0.02}_{-0.01}$	1.505	1.5	${}^{-1.1}_{+1.0}$	${}^{+2.7}_{-4.0}$	${}^{+ 0.7}_{-1.5}$	${}^{+0.3}_{-1.0}$	${}^{+2.0}_{-3.2}$	${}^{+1.4}_{-2.5}$	${}^{+ 0.6}_{-1.6}$	${}^{+ 0.3}_{-1.0}$	${}^{-1.5}_{+ 0.7}$	${}^{-2.3}_{+2.3}$	${}^{-0.4}_{-0.1}$	${}^{+ 0.2}_{-0.3}$	${}^{-5.4}_{+4.6}$	∓0.2	±0.0	±0.8	±0.1	0.0	±1.4	${}^{+ 0.5}_{-0.5}$	±0.8
(6.00–7.43) e-02	$1.02 ^{+ 0.02}_{-0.01}$	1.452	3.1	${}^{-0.4}_{+0.4}$	${}^{+3.9}_{-4.0}$	${}^{+ 0.6}_{-0.4}$	${}^{-0.5}_{+ 0.7}$	${}^{+0.1}_{+ 0.8}$	${}^{-0.5}_{-0.3}$	${}^{+1.4}_{-2.0}$	${}^{+ 1.4}_{-0.7}$	${}^{-1.5}_{+ 0.9}$	${}^{-2.1}_{+2.8}$	${}^{+ 0.2}_{+ 0.7}$	${}^{-0.4}_{+ 0.4}$	${}^{-4.4}_{+4.7}$	∓1.0	±0.5	±0.8	±0.1	0.0	±1.4	${}^{+ 0.5}_{-0.5}$	±0.8
(7.43–8.86) e-02	$1.02 ^{+ 0.02}_{-0.01}$	1.497	6.0	${}^{-0.0}_{+0.0}$	${}^{+2.2}_{-4.0}$	${}^{+ 0.5}_{-1.9}$	${}^{-1.6}_{+ 0.9}$	${}^{-4.1}_{+2.9}$	${}^{-1.2}_{-0.3}$	${}^{+ 0.5}_{-2.1}$	${}^{-0.1}_{-0.8}$	${}^{-0.9}_{+ 0.8}$	${}^{-3.4}_{+3.0}$	${}^{-0.8}_{-0.1}$	${}^{-0.1}_{+ 0.1}$	${}^{-3.9}_{+3.1}$	∓2.3	±0.0	±0.8	±0.0	0.0	±1.4	${}^{+ 0.6}_{-0.5}$	±0.7
(8.86–11.4) e-02	$1.02 ^{+ 0.03}_{-0.01}$	1.617	12.	${}^{-0.0}_{+0.0}$	${}^{+5.0}_{-2.9}$	${}^{+ 0.6}_{-0.5}$	${}^{-0.6}_{+ 0.4}$	${}^{-2.7}_{+2.5}$	${}^{+ 0.9}_{+ 0.3}$	${}^{+2.8}_{-3.0}$	${}^{+1.1}_{-1.2}$	${}^{+ 0.1}_{-0.1}$	${}^{-2.8}_{+2.9}$	${}^{-0.4}_{-0.2}$	${}^{-0.0}_{+ 0.0}$	${}^{-3.0}_{+4.1}$	∓4.9	±0.1	±0.5	±0.2	0.0	±1.4	${}^{+ 0.5}_{-0.5}$	±1.1
(1.14–1.43) e-01	$1.02 ^{+ 0.03}_{-0.01}$	2.594	35.	${}^{-0.0}_{+0.0}$	${}^{-1.3}_{+2.2}$	${}^{+ 1.4}_{+ 0.0}$	${}^{-0.6}_{+ 0.9}$	${}^{+1.2}_{+1.5}$	${}^{+1.4}_{+2.8}$	${}^{+3.8}_{-3.1}$	${}^{+0.3}_{-2.1}$	${}^{+2.4}_{+1.8}$	${}^{-4.3}_{+4.6}$	${}^{+ 0.3}_{+ 0.2}$	${}^{-0.0}_{+ 0.0}$	${}^{-0.8}_{+ 7.6}$	∓3.8	∓0.4	±1.5	±0.3	0.0	±1.4	${}^{+ 0.5}_{-0.5}$	±0.7
(1.43–1.71) e-01	$1.02 ^{+ 0.03}_{-0.01}$	6.804	143	${}^{-0.2}_{+0.2}$	${}^{+8.2}_{-7.5}$	${}^{+12.}_{-9.9}$	${}^{-1.0}_{+ 0.8}$	${}^{+8.7}_{-6.9}$	${}^{ +12}_{ -13}$	${}^{+5.8}_{-5.4}$	${}^{+2.2}_{-1.0}$	${}^{ +16}_{ -13}$	${}^{+4.2}_{-6.5}$	${}^{+0.8}_{-0.4}$	${}^{-0.0}_{+ 0.0}$	${}^{+5.9}_{-9.4}$	∓2.1	∓0.3	±8.9	±0.0	0.0	±1.4	${}^{+ 0.5}_{-0.6}$	±0.1

**Table 30:** Measured jet cross section ratio in *x*_T_ bin for anti-*k*_*t*_ jets with *R*=0.6 in the rapidity bin 2.8≤|*y*|<3.6. See caption of Table 18 for details

*x*_T_	NPCorr	*ρ*	$\delta_{\mathrm{stat.}}$ %	*γ*_5_ %	*γ*_11_ %	*γ*_17_ %	*γ*_23_ %	*γ*_29_ %	*γ*_31_ %	*γ*_36_ %	*γ*_42_ %	*γ*_48_ %	*γ*_54_ %	*γ*_60_ %	*γ*_66_ %	*γ*_72_ %	*γ*_80_ %	*γ*_82_ %	*γ*_74_ %	*γ*_75_ %	*γ*_85_ %	*u*_1_ %	*u*_2_ %	*u*_3_ %
(1.71–2.29) e-02	$1.04 ^{+ 0.07}_{-0.04}$	1.337	2.8	${}^{ +15}_{-13}$	${}^{+4.5}_{-5.5}$	${}^{+9.8}_{-8.6}$	${}^{+1.5}_{-1.3}$	${}^{+1.6}_{-1.6}$	${}^{ +24}_{ -24}$	${}^{-0.5}_{+ 0.3}$	${}^{+3.2}_{-3.4}$	${}^{+ 0.8}_{-1.6}$	${}^{-0.6}_{+ 0.4}$	${}^{+0.0}_{-0.1}$	${}^{+3.0}_{-3.1}$	${}^{-2.4}_{+2.1}$	∓15	±0.4	±0.4	±0.7	±0.9	±2.2	${}^{+0.5}_{-0.5}$	±4.1
(2.29–3.14) e-02	$1.02 ^{+ 0.06}_{-0.02}$	1.381	3.2	${}^{+9.2}_{-8.0}$	${}^{+5.8}_{-5.7}$	${}^{+7.6}_{-7.6}$	${}^{+ 0.5}_{+0.3}$	${}^{+ 0.4}_{+ 0.5}$	${}^{ +28}_{ -20}$	${}^{+ 0.1}_{+ 0.5}$	${}^{+2.5}_{-1.9}$	${}^{+ 0.3}_{+ 0.5}$	${}^{-1.4}_{+2.0}$	${}^{+ 0.1}_{+0.5}$	${}^{+5.1}_{-4.6}$	${}^{-5.0}_{+5.5}$	∓4.9	±0.0	±0.3	±0.4	0.0	±2.2	${}^{+ 0.5}_{-0.5}$	±4.9
(3.14–4.57) e-02	$1.01 ^{+ 0.05}_{-0.02}$	1.313	2.3	${}^{+1.4}_{-3.6}$	${}^{+3.8}_{-5.8}$	${}^{+6.0}_{-7.4}$	${}^{-4.8}_{+4.0}$	${}^{+1.2}_{-2.7}$	${}^{ +14}_{ -17}$	${}^{+0.1}_{-2.4}$	${}^{+1.1}_{-2.5}$	${}^{-1.6}_{+ 0.3}$	${}^{-2.2}_{+2.5}$	${}^{+ 0.1}_{-0.0}$	${}^{+3.1}_{-4.6}$	${}^{-7.0}_{+7.8}$	±1.4	±1.3	±2.2	±0.0	0.0	±1.6	${}^{+ 0.5}_{-0.5}$	±3.1
(4.57–6.00) e-02	$1.01 ^{+ 0.05}_{-0.02}$	1.376	5.2	${}^{-1.9}_{+2.1}$	${}^{+4.3}_{-6.0}$	${}^{+1.8}_{-1.3}$	${}^{-3.1}_{+3.0}$	${}^{+3.7}_{-3.6}$	${}^{+5.1}_{-3.5}$	${}^{+1.7}_{-2.0}$	${}^{+ 0.9}_{-1.5}$	${}^{-1.8}_{+1.5}$	${}^{-3.8}_{+3.5}$	${}^{-0.6}_{-0.2}$	${}^{+ 0.4}_{+ 0.4}$	${}^{-8.0}_{+7.2}$	∓2.4	±0.5	±4.9	±0.1	0.0	±1.4	${}^{+ 0.6}_{-0.5}$	±3.2
(6.00–7.43) e-02	$1.01 ^{+ 0.05}_{-0.02}$	1.134	16.	${}^{-0.7}_{+1.0}$	${}^{+10.}_{-4.7}$	${}^{+1.7}_{+1.1}$	${}^{+ 1.3}_{-0.8}$	${}^{+0.1}_{+ 3.2}$	${}^{+ 4.5}_{+ 0.0}$	${}^{+5.1}_{-2.1}$	${}^{+2.6}_{-0.5}$	${}^{-1.0}_{+4.0}$	${}^{-4.3}_{+4.7}$	${}^{+ 0.2}_{+ 0.8}$	${}^{-0.8}_{+ 0.9}$	${}^{-4.8}_{+8.8}$	∓2.8	±1.1	±3.0	±0.0	0.0	±1.4	${}^{+ 0.6}_{-0.5}$	±3.8
(7.43–8.86) e-02	$1.01 ^{+ 0.05}_{-0.02}$	3.696	61.	${}^{-0.4}_{+0.5}$	${}^{+5.1}_{-2.9}$	${}^{-3.3}_{+ 0.6}$	${}^{-4.3}_{-0.9}$	${}^{-8.6}_{+8.5}$	${}^{-8.0}_{+17.}$	${}^{+1.4}_{-2.0}$	${}^{-1.2}_{-0.1}$	${}^{-3.0}_{+9.6}$	${}^{-7.8}_{+4.2}$	${}^{-2.6}_{-0.1}$	${}^{-0.4}_{+ 0.3}$	${}^{ -12}_{ +12}$	∓7.8	∓0.9	±5.8	±0.7	0.0	±1.4	${}^{+ 0.6}_{-0.5}$	±3.9

**Table 31:** Measured jet cross section ratio in *x*_T_ bin for anti-*k*_*t*_ jets with *R*=0.6 in the rapidity bin 3.6≤|*y*|<4.4. See caption of Table 18 for details

*x*_T_	NPCorr	*ρ*	$\delta_{\mathrm{stat.}}$ %	*γ*_6_ %	*γ*_12_ %	*γ*_18_ %	*γ*_24_ %	*γ*_30_ %	*γ*_31_ %	*γ*_37_ %	*γ*_43_ %	*γ*_49_ %	*γ*_55_ %	*γ*_61_ %	*γ*_67_ %	*γ*_73_ %	*γ*_81_ %	*γ*_82_ %	*γ*_74_ %	*γ*_75_ %	*γ*_86_ %	*u*_1_ %	*u*_2_ %	*u*_3_ %
(1.71–2.29) e-02	$0.98 ^{+ 0.08}_{-0.02}$	1.469	6.7	${}^{ +22}_{-15}$	${}^{+11.}_{-4.2}$	${}^{+17.}_{-8.1}$	${}^{+3.7}_{-2.8}$	${}^{+2.2}_{+ 0.1}$	${}^{ +71}_{ -48}$	${}^{+ 0.0}_{+ 1.0}$	${}^{+4.8}_{-2.7}$	${}^{+ 1.6}_{+ 0.3}$	${}^{-0.3}_{+ 1.8}$	${}^{+0.0}_{-0.1}$	${}^{+6.0}_{-4.0}$	${}^{-3.4}_{+5.1}$	∓16	±2.0	±1.8	±1.7	±0.9	±2.2	${}^{+0.5}_{-0.5}$	±5.6
(2.29–3.14) e-02	$0.97 ^{+ 0.08}_{-0.04}$	1.344	6.0	${}^{ +13}_{-14}$	${}^{+5.1}_{-12.}$	${}^{ +10}_{ -16}$	${}^{-1.2}_{-5.6}$	${}^{-1.7}_{-4.7}$	${}^{+100}_{ -50}$	${}^{-1.3}_{-4.7}$	${}^{+2.3}_{-7.1}$	${}^{-1.0}_{-4.7}$	${}^{-2.1}_{+2.1}$	${}^{+ 0.1}_{+0.6}$	${}^{+5.4}_{-11.}$	${}^{-7.6}_{+8.3}$	∓0.3	±2.0	±3.6	±3.6	0.0	±1.4	${}^{+ 0.5}_{-0.5}$	±7.1
(3.14–4.57) e-02	$0.94 ^{+ 0.13}_{-0.09}$	1.381	20.	${}^{+0.1}_{-2.9}$	${}^{+23.}_{-7.5}$	${}^{ +25}_{ -10}$	${}^{-7.8}_{+7.6}$	${}^{+1.6}_{-1.8}$	${}^{+120}_{ -45}$	${}^{+2.8}_{+1.8}$	${}^{+0.8}_{-1.1}$	${}^{+11.}_{+2.1}$	${}^{-3.4}_{+7.0}$	${}^{+ 0.0}_{-0.0}$	${}^{+1.9}_{-5.4}$	${}^{-9.7}_{+16.}$	∓18	∓0.6	±2.5	±1.1	0.0	±1.4	${}^{+ 0.5}_{-0.5}$	±8.3

**Table 32:** Measured jet cross section ratio in bins of *p*_T_ for anti-*k*_*t*_ jets with *R*=0.4 in the rapidity bin |*y*|<0.3. NPCorr stands for multiplicative non-perturbative corrections, *ρ* is the measured cross section and $\delta_{\mathrm{stat.}}$ is the statistical uncertainty. *γ*_*i*_ and *u*_*j*_ correspond to the correlated and uncorrelated systematic uncertainties given in %. They are described in Table 1, where *i* in *γ*_*i*_ denotes a nuisance parameter. *u*_3_ is the uncertainty due to pile-up effects in the cross section measurement at $\sqrt{s}=7~\mbox{TeV}$. For each table entry, the outcome of shifting the corresponding nuisance parameter by one standard deviation, up or down, is shown as superscript or subscript, respectively. In some bins these shifts may lead to cross-section variations in the same direction. The 4.3 % uncertainty from the luminosity measurements is not in this table

*p*_T_ (GeV)	NPCorr	*ρ*	$\delta_{\mathrm{stat.}}$ %	*γ*_1_ %	*γ*_7_ %	*γ*_13_ %	*γ*_19_ %	*γ*_25_ %	*γ*_31_ %	*γ*_32_ %	*γ*_38_ %	*γ*_44_ %	*γ*_50_ %	*γ*_56_ %	*γ*_62_ %	*γ*_68_ %	*γ*_76_ %	*γ*_82_ %	*γ*_74_ %	*γ*_75_ %	*γ*_83_ %	*u*_1_ %	*u*_2_ %	*u*_3_ %
20–30	$0.90 ^{+ 0.03}_{-0.12}$	2.783e-01	1.9	${}^{+ 2.5}_{-0.0}$	${}^{+ 1.1}_{-0.4}$	${}^{+ 0.7}_{-0.3}$	${}^{-1.0}_{-0.6}$	${}^{+1.2}_{+ 0.1}$	0.0	${}^{-0.1}_{+ 0.1}$	${}^{+ 0.4}_{+ 0.0}$	${}^{+0.4}_{+ 0.0}$	${}^{-0.0}_{+ 0.0}$	${}^{+ 0.0}_{-0.0}$	${}^{-0.4}_{-0.1}$	${}^{-0.0}_{+ 0.0}$	∓0.3	∓0.1	±0.2	±1.6	0.0	±1.4	${}^{+ 0.7}_{-0.5}$	±0.8
30–45	$0.92 ^{+ 0.03}_{-0.05}$	2.711e-01	3.2	${}^{-0.1}_{+ 1.3}$	${}^{+ 0.6}_{+ 0.3}$	${}^{+ 0.8}_{+ 0.6}$	${}^{+ 1.5}_{-0.4}$	${}^{+0.4}_{+ 0.5}$	0.0	${}^{+ 1.0}_{-0.1}$	${}^{+ 0.4}_{+ 0.3}$	${}^{+1.0}_{+ 0.1}$	${}^{+ 0.0}_{-0.0}$	${}^{+ 0.0}_{-0.0}$	${}^{+1.0}_{-0.2}$	${}^{-0.0}_{+ 0.0}$	∓2.6	±0.0	±0.1	±1.1	0.0	±1.4	${}^{+ 0.6}_{-0.5}$	±0.5
45–60	$0.94 ^{+ 0.02}_{-0.02}$	2.246e-01	4.0	${}^{-0.3}_{+ 0.5}$	${}^{-0.2}_{-0.2}$	${}^{+ 0.1}_{+ 0.1}$	${}^{+ 0.3}_{+ 0.2}$	${}^{+0.1}_{-0.1}$	0.0	${}^{+ 0.3}_{+ 0.1}$	${}^{-0.0}_{+ 0.4}$	${}^{+0.3}_{-0.1}$	${}^{+ 0.1}_{-0.2}$	${}^{-0.3}_{-0.0}$	${}^{+ 0.4}_{-0.0}$	${}^{-0.0}_{-0.0}$	∓0.7	∓0.0	±0.2	±0.7	0.0	±1.4	${}^{+ 0.6}_{-0.5}$	±0.3
60–80	$0.96 ^{+ 0.02}_{-0.02}$	1.922e-01	1.6	${}^{-0.2}_{+ 0.1}$	${}^{+ 0.4}_{-0.8}$	${}^{-0.1}_{-0.2}$	${}^{+ 0.5}_{-0.3}$	${}^{+0.1}_{+ 0.1}$	0.0	${}^{-0.1}_{+ 0.1}$	${}^{+ 0.2}_{+ 0.1}$	${}^{+0.5}_{-0.6}$	${}^{+ 0.1}_{-0.0}$	${}^{+ 0.0}_{-0.1}$	${}^{+0.1}_{-0.1}$	${}^{-0.0}_{-0.0}$	∓0.4	±0.1	±0.2	±0.4	0.0	±1.4	${}^{+ 0.5}_{-0.5}$	±0.3
80–110	$0.98 ^{+ 0.02}_{-0.01}$	1.618e-01	1.8	${}^{-0.0}_{-0.0}$	${}^{+ 1.7}_{-0.7}$	${}^{+ 0.9}_{-0.1}$	${}^{+ 0.8}_{-0.4}$	${}^{+0.5}_{-0.2}$	0.0	${}^{+ 1.0}_{-0.4}$	${}^{+ 0.4}_{-0.1}$	${}^{+1.2}_{-0.1}$	${}^{+ 0.6}_{-0.2}$	${}^{+ 0.1}_{+ 0.2}$	${}^{+0.1}_{-0.1}$	${}^{+ 0.4}_{-0.1}$	±1.0	±0.0	±0.2	±2.0	0.0	±1.4	${}^{+ 0.5}_{-0.5}$	±0.4
110–160	$0.99 ^{+ 0.02}_{-0.01}$	1.343e-01	3.2	${}^{+0.0}_{-0.0}$	${}^{+ 0.3}_{-0.8}$	${}^{+ 0.3}_{-0.7}$	${}^{+0.3}_{-0.3}$	${}^{-0.0}_{-0.0}$	0.0	${}^{-0.0}_{-0.4}$	${}^{-0.2}_{+0.1}$	${}^{+ 0.4}_{-1.1}$	${}^{-0.1}_{-0.3}$	${}^{+ 0.2}_{+ 0.2}$	${}^{-0.1}_{+ 0.1}$	${}^{+ 0.1}_{-0.5}$	∓0.5	±0.0	±0.3	±0.1	0.0	±1.4	${}^{+ 0.5}_{-0.5}$	±0.4
160–210	$0.99 ^{+ 0.01}_{-0.01}$	1.003e-01	8.6	${}^{+0.0}_{-0.0}$	${}^{+ 0.6}_{-1.1}$	${}^{+ 0.7}_{-1.1}$	${}^{+0.1}_{-0.6}$	${}^{-0.1}_{-0.2}$	0.0	${}^{+ 0.3}_{-0.5}$	${}^{+0.1}_{-0.3}$	${}^{+1.1}_{-1.6}$	${}^{+ 0.3}_{-1.0}$	${}^{+ 0.1}_{-0.5}$	${}^{-0.0}_{+ 0.0}$	${}^{+1.1}_{-1.3}$	∓1.1	±0.2	±0.6	±0.1	0.0	±1.4	${}^{+ 0.5}_{-0.5}$	±0.4
210–260	$1.00 ^{+ 0.01}_{-0.01}$	6.674e-02	19.	${}^{+0.0}_{-0.0}$	${}^{+ 0.5}_{-0.2}$	${}^{+ 0.5}_{-0.0}$	${}^{+ 0.3}_{+0.3}$	${}^{+ 0.7}_{+ 0.0}$	0.0	${}^{+ 0.2}_{+ 0.0}$	${}^{+ 0.1}_{+0.1}$	${}^{+ 0.9}_{-0.2}$	${}^{+ 0.7}_{+ 0.1}$	${}^{+ 0.1}_{+ 0.1}$	${}^{+ 0.0}_{-0.0}$	${}^{+ 0.9}_{-0.3}$	±0.3	±0.0	±0.6	±0.0	0.0	±1.4	${}^{+ 0.5}_{-0.5}$	±0.3
260–310	$1.00 ^{+ 0.01}_{-0.01}$	5.769e-02	39.	${}^{-0.0}_{+0.0}$	${}^{+ 0.5}_{-0.8}$	${}^{+ 0.1}_{-0.1}$	${}^{-0.1}_{+ 0.1}$	${}^{+ 0.1}_{-0.4}$	0.0	${}^{+ 0.5}_{-0.6}$	${}^{+ 0.0}_{-0.2}$	${}^{+0.9}_{-1.6}$	${}^{+ 0.6}_{-0.9}$	${}^{-0.3}_{-0.1}$	${}^{+ 0.0}_{-0.0}$	${}^{+ 0.9}_{-1.7}$	±0.6	±0.1	±0.6	±0.0	0.0	±1.4	${}^{+ 0.5}_{-0.5}$	±0.3
310–400	$1.00 ^{+ 0.01}_{-0.01}$	7.173e-02	46.	${}^{-0.0}_{+0.0}$	${}^{+ 0.2}_{-0.5}$	${}^{+ 0.0}_{-0.5}$	${}^{-0.1}_{-0.6}$	${}^{+0.4}_{-0.7}$	0.0	${}^{+ 0.2}_{-0.3}$	${}^{-0.2}_{-0.3}$	${}^{+2.2}_{-1.7}$	${}^{+1.2}_{-1.5}$	${}^{-0.3}_{-0.2}$	${}^{+0.0}_{-0.0}$	${}^{+2.7}_{-2.6}$	±0.1	∓0.0	±0.4	±0.0	0.0	±1.4	${}^{+ 0.5}_{-0.5}$	±0.3
400–500	$1.00 ^{+ 0.01}_{-0.01}$	5.275e-02	115	0.0	${}^{+0.7}_{-0.3}$	${}^{+ 0.8}_{-0.4}$	${}^{+1.9}_{-1.1}$	${}^{+1.9}_{-1.1}$	0.0	${}^{+ 1.2}_{-0.8}$	${}^{+ 0.4}_{+ 0.0}$	${}^{+3.2}_{-2.7}$	${}^{+2.6}_{-2.0}$	${}^{+ 0.7}_{-0.2}$	0.0	${}^{+4.2}_{-3.1}$	∓0.6	±0.2	±0.6	±0.0	0.0	±1.4	${}^{+0.5}_{-0.5}$	±0.2

**Table 33:** Measured jet cross section ratio in *p*_T_ bin for anti-*k*_*t*_ jets with *R*=0.4 in the rapidity bin 0.3≤|*y*|<0.8. See caption of Table 32 for details

*p*_T_ (GeV)	NPCorr	*ρ*	$\delta_{\mathrm{stat.}}$ %	*γ*_1_ %	*γ*_7_ %	*γ*_13_ %	*γ*_19_ %	*γ*_25_ %	*γ*_31_ %	*γ*_32_ %	*γ*_38_ %	*γ*_44_ %	*γ*_50_ %	*γ*_56_ %	*γ*_62_ %	*γ*_68_ %	*γ*_76_ %	*γ*_82_ %	*γ*_74_ %	*γ*_75_ %	*γ*_83_ %	*u*_1_ %	*u*_2_ %	*u*_3_ %
20–30	$0.90 ^{+ 0.01}_{-0.12}$	2.829e-01	1.5	${}^{+ 2.3}_{+ 0.5}$	${}^{-1.3}_{-0.2}$	${}^{+ 1.5}_{+ 0.3}$	${}^{-1.0}_{-0.2}$	${}^{+0.1}_{+ 0.2}$	${}^{+ 0.2}_{-0.1}$	${}^{-0.1}_{-0.2}$	${}^{+ 0.2}_{+0.0}$	${}^{-0.1}_{-0.2}$	${}^{+ 0.0}_{+ 0.0}$	${}^{+ 0.1}_{-0.0}$	${}^{-0.3}_{-0.1}$	${}^{-0.0}_{+ 0.0}$	∓1.6	∓0.2	±0.2	±1.3	0.0	±1.4	${}^{+ 0.7}_{-0.5}$	±0.8
30–45	$0.92 ^{+ 0.02}_{-0.05}$	2.586e-01	2.6	${}^{-0.7}_{+ 1.3}$	${}^{+ 1.1}_{-0.6}$	${}^{-0.0}_{+ 0.6}$	${}^{+ 0.8}_{-0.4}$	${}^{-0.1}_{+ 0.1}$	${}^{+ 0.1}_{-0.1}$	${}^{+ 0.4}_{-0.2}$	${}^{+0.2}_{+ 0.2}$	${}^{+ 0.3}_{-0.1}$	${}^{+ 0.1}_{-0.0}$	${}^{+0.1}_{-0.1}$	${}^{+ 0.4}_{-0.0}$	${}^{+ 0.0}_{+ 0.0}$	∓0.9	±0.1	±0.0	±1.0	0.0	±1.4	${}^{+ 0.6}_{-0.5}$	±0.5
45–60	$0.95 ^{+ 0.02}_{-0.02}$	2.057e-01	3.0	${}^{-0.6}_{-0.1}$	${}^{+ 0.5}_{-0.6}$	${}^{-0.1}_{-0.4}$	${}^{+ 0.2}_{-0.3}$	${}^{-0.0}_{-0.6}$	${}^{+ 0.1}_{-0.1}$	${}^{-0.1}_{-0.4}$	${}^{+0.0}_{-0.5}$	${}^{+ 0.3}_{-0.6}$	${}^{-0.1}_{-0.3}$	${}^{-0.3}_{-0.0}$	${}^{-0.0}_{-0.2}$	${}^{-0.0}_{-0.4}$	±0.6	±0.1	±0.2	±0.5	0.0	±1.4	${}^{+ 0.6}_{-0.5}$	±0.3
60–80	$0.96 ^{+ 0.02}_{-0.02}$	1.886e-01	1.2	${}^{-0.2}_{+ 0.0}$	${}^{+ 0.0}_{-1.0}$	${}^{-0.3}_{-0.5}$	${}^{-0.2}_{-0.5}$	${}^{+0.1}_{-0.4}$	${}^{+ 0.1}_{-0.0}$	${}^{-0.2}_{-0.3}$	${}^{-0.4}_{-0.1}$	${}^{-0.2}_{-0.7}$	${}^{-0.1}_{-0.2}$	${}^{-0.0}_{-0.1}$	${}^{-0.3}_{-0.0}$	${}^{+ 0.2}_{-0.7}$	∓0.5	±0.4	±0.2	±0.1	0.0	±1.4	${}^{+ 0.5}_{-0.5}$	±0.4
80–110	$0.98 ^{+ 0.02}_{-0.01}$	1.625e-01	1.4	${}^{-0.0}_{+ 0.0}$	${}^{+ 0.7}_{-0.4}$	${}^{+ 0.8}_{-0.4}$	${}^{+ 0.8}_{-0.4}$	${}^{+0.4}_{+ 0.0}$	${}^{+ 0.0}_{-0.0}$	${}^{+ 0.5}_{-0.2}$	${}^{+ 0.3}_{+0.1}$	${}^{+ 0.7}_{-0.3}$	${}^{+ 0.4}_{+ 0.0}$	${}^{+ 0.1}_{+ 0.3}$	${}^{-0.0}_{+ 0.1}$	${}^{+ 0.7}_{-0.5}$	∓0.4	±0.1	±0.2	±0.3	0.0	±1.4	${}^{+ 0.5}_{-0.5}$	±0.4
110–160	$0.99 ^{+ 0.02}_{-0.01}$	1.295e-01	2.6	${}^{+0.0}_{-0.0}$	${}^{+ 0.8}_{-0.7}$	${}^{+ 0.4}_{-0.5}$	${}^{+0.2}_{-0.2}$	${}^{+ 0.1}_{-0.2}$	${}^{+ 0.0}_{-0.0}$	${}^{+0.2}_{-0.3}$	${}^{+ 0.1}_{-0.2}$	${}^{+ 0.7}_{-1.1}$	${}^{+0.3}_{-0.4}$	${}^{+ 0.1}_{-0.1}$	${}^{+ 0.0}_{+ 0.0}$	${}^{+0.9}_{-1.0}$	±0.5	±0.2	±0.6	±0.1	0.0	±1.4	${}^{+ 0.5}_{-0.5}$	±0.4
160–210	$1.00 ^{+ 0.01}_{-0.01}$	8.841e-02	7.4	${}^{+0.0}_{-0.0}$	${}^{+ 0.4}_{-1.2}$	${}^{+ 0.1}_{-1.1}$	${}^{+0.3}_{-1.1}$	${}^{+ 0.1}_{-1.0}$	${}^{+ 0.0}_{-0.0}$	${}^{-0.0}_{-0.8}$	${}^{-0.5}_{-0.5}$	${}^{+ 0.6}_{-1.3}$	${}^{+ 0.3}_{-1.2}$	${}^{-0.3}_{-0.3}$	${}^{+ 0.0}_{-0.0}$	${}^{+ 0.6}_{-1.8}$	±0.4	±0.1	±0.3	±0.1	0.0	±1.4	${}^{+ 0.5}_{-0.5}$	±0.5
210–260	$1.00 ^{+ 0.01}_{-0.01}$	5.519e-02	18.	${}^{+0.0}_{-0.0}$	${}^{+ 1.3}_{-0.2}$	${}^{+ 1.0}_{+ 0.1}$	${}^{+0.5}_{-0.2}$	${}^{+ 1.3}_{-0.5}$	${}^{+ 0.0}_{-0.0}$	${}^{+0.8}_{-0.4}$	${}^{+ 0.6}_{+ 0.1}$	${}^{+ 1.8}_{-0.6}$	${}^{+1.1}_{-0.3}$	${}^{+ 0.2}_{+ 0.2}$	${}^{+ 0.0}_{-0.0}$	${}^{+1.9}_{-1.0}$	∓0.3	±0.1	±0.2	±0.0	0.0	±1.4	${}^{+ 0.5}_{-0.5}$	±0.3
260–310	$1.00 ^{+ 0.01}_{-0.01}$	4.967e-02	32.	${}^{+0.0}_{-0.0}$	${}^{+ 0.5}_{-0.8}$	${}^{-0.1}_{-0.9}$	${}^{-0.3}_{-0.4}$	${}^{+ 0.1}_{-0.8}$	${}^{+ 0.1}_{-0.0}$	${}^{-0.2}_{-0.2}$	${}^{-0.2}_{-0.2}$	${}^{+1.5}_{-1.5}$	${}^{+ 0.6}_{-0.9}$	${}^{-0.1}_{-0.1}$	${}^{+ 0.0}_{-0.0}$	${}^{+1.9}_{-2.1}$	∓0.4	±0.0	±0.3	±0.0	0.0	±1.4	${}^{+ 0.5}_{-0.5}$	±0.3
310–400	$1.00 ^{+ 0.01}_{-0.01}$	6.368e-02	44.	${}^{+0.0}_{-0.0}$	${}^{+1.2}_{-1.1}$	${}^{+ 1.1}_{-0.5}$	${}^{+ 0.5}_{-0.2}$	${}^{+ 0.9}_{-0.8}$	${}^{+ 0.0}_{-0.0}$	${}^{+ 0.3}_{-0.5}$	${}^{+0.4}_{-0.2}$	${}^{+2.5}_{-2.5}$	${}^{+2.1}_{-1.3}$	${}^{-0.1}_{-0.3}$	${}^{+ 0.0}_{-0.0}$	${}^{+3.3}_{-3.2}$	±0.0	∓0.0	±0.3	±0.0	0.0	±1.4	${}^{+ 0.5}_{-0.5}$	±0.3
400–500	$1.00 ^{+ 0.01}_{-0.01}$	7.413e-02	78.	0.0	${}^{+1.7}_{-2.4}$	${}^{+1.5}_{-2.3}$	${}^{+ 0.9}_{-0.9}$	${}^{+1.1}_{-1.8}$	${}^{+ 0.0}_{-0.0}$	${}^{+ 0.9}_{-1.0}$	${}^{+0.2}_{-0.4}$	${}^{+3.9}_{-4.6}$	${}^{+2.6}_{-3.5}$	${}^{+ 0.5}_{-0.2}$	${}^{+ 0.0}_{-0.0}$	${}^{+5.8}_{-6.3}$	∓0.2	±0.0	±0.5	±0.0	0.0	±1.4	${}^{+ 0.5}_{-0.5}$	±0.3

**Table 34:** Measured jet cross section ratio in *p*_T_ bin for anti-*k*_*t*_ jets with *R*=0.4 in the rapidity bin 0.8≤|*y*|<1.2. See caption of Table 32 for details

*p*_T_ (GeV)	NPCorr	*ρ*	$\delta_{\mathrm{stat.}}$ %	*γ*_2_ %	*γ*_8_ %	*γ*_14_ %	*γ*_20_ %	*γ*_26_ %	*γ*_31_ %	*γ*_33_ %	*γ*_39_ %	*γ*_45_ %	*γ*_51_ %	*γ*_57_ %	*γ*_63_ %	*γ*_69_ %	*γ*_77_ %	*γ*_82_ %	*γ*_74_ %	*γ*_75_ %	*γ*_83_ %	*u*_1_ %	*u*_2_ %	*u*_3_ %
20–30	$0.90 ^{+ 0.01}_{-0.12}$	2.584e-01	1.8	${}^{+ 1.4}_{+ 0.5}$	${}^{-1.2}_{-0.1}$	${}^{+ 0.8}_{+ 0.4}$	${}^{+1.3}_{+1.0}$	${}^{+0.2}_{+ 0.3}$	${}^{+ 0.1}_{-0.3}$	${}^{+ 0.1}_{-0.1}$	${}^{+ 0.2}_{+0.1}$	${}^{+ 0.0}_{+ 0.0}$	${}^{+ 0.0}_{+ 0.0}$	${}^{+ 0.1}_{-0.0}$	${}^{-0.1}_{+ 0.1}$	${}^{-0.0}_{+ 0.0}$	∓0.1	∓0.0	±0.3	±1.0	0.0	±1.4	${}^{+ 0.6}_{-0.5}$	±1.1
30–45	$0.93 ^{+ 0.01}_{-0.05}$	2.367e-01	3.1	${}^{-1.3}_{+ 1.0}$	${}^{+ 0.6}_{-0.8}$	${}^{-0.9}_{-0.0}$	${}^{-2.1}_{+1.6}$	${}^{-0.6}_{-0.1}$	${}^{-0.3}_{-0.9}$	${}^{-0.1}_{-0.5}$	${}^{-0.3}_{-0.1}$	${}^{-0.3}_{-0.3}$	${}^{+ 0.0}_{-0.0}$	${}^{+0.1}_{-0.0}$	${}^{-0.1}_{-0.4}$	${}^{-0.0}_{+ 0.0}$	±1.2	∓0.1	±0.0	±1.0	0.0	±1.4	${}^{+ 0.6}_{-0.5}$	±0.9
45–60	$0.95 ^{+ 0.01}_{-0.02}$	1.930e-01	3.5	${}^{-0.3}_{+ 0.2}$	${}^{+ 0.5}_{-0.5}$	${}^{+ 0.1}_{-0.4}$	${}^{-0.7}_{+ 0.0}$	${}^{-0.1}_{-0.2}$	${}^{+ 0.5}_{-0.6}$	${}^{-0.2}_{-0.1}$	${}^{-0.1}_{-0.1}$	${}^{+ 0.2}_{-0.4}$	${}^{-0.1}_{-0.3}$	${}^{-0.3}_{-0.0}$	${}^{+ 0.0}_{-0.0}$	${}^{-0.2}_{-0.1}$	∓0.8	±0.2	±0.5	±0.5	0.0	±1.4	${}^{+ 0.6}_{-0.5}$	±0.6
60–80	$0.97 ^{+ 0.01}_{-0.02}$	1.751e-01	1.5	${}^{-0.1}_{+ 0.1}$	${}^{+ 0.9}_{-0.8}$	${}^{+ 1.0}_{-0.5}$	${}^{+ 1.0}_{-0.5}$	${}^{+0.6}_{-0.2}$	${}^{+ 1.4}_{-0.8}$	${}^{+ 0.9}_{-0.4}$	${}^{+0.3}_{-0.0}$	${}^{+ 1.0}_{-0.6}$	${}^{+ 0.1}_{-0.3}$	${}^{-0.0}_{-0.1}$	${}^{+ 0.1}_{+ 0.2}$	${}^{+ 0.6}_{-0.7}$	±0.3	∓0.0	±0.5	±0.1	0.0	±1.4	${}^{+ 0.6}_{-0.5}$	±0.5
80–110	$0.99 ^{+ 0.02}_{-0.01}$	1.536e-01	1.7	${}^{-0.0}_{-0.0}$	${}^{+ 0.9}_{+ 0.2}$	${}^{+ 0.4}_{+ 0.2}$	${}^{+ 0.3}_{+ 0.3}$	${}^{+0.3}_{+ 0.1}$	${}^{+ 0.2}_{+ 0.3}$	${}^{+ 0.2}_{+ 0.4}$	${}^{+ 0.2}_{+0.2}$	${}^{+ 0.5}_{+ 0.2}$	${}^{+ 0.3}_{+ 0.2}$	${}^{+ 0.1}_{+ 0.3}$	${}^{-0.0}_{+ 0.0}$	${}^{+ 0.4}_{+ 0.1}$	∓1.4	±0.1	±0.5	±0.3	0.0	±1.4	${}^{+ 0.5}_{-0.5}$	±0.8
110–160	$0.99 ^{+ 0.02}_{-0.01}$	1.183e-01	3.2	${}^{+0.0}_{-0.0}$	${}^{+ 1.2}_{+ 0.0}$	${}^{+ 1.0}_{-0.0}$	${}^{+ 0.3}_{+0.2}$	${}^{+ 0.3}_{-0.0}$	${}^{+ 1.1}_{-0.2}$	${}^{+ 0.6}_{+ 0.1}$	${}^{+ 0.2}_{+ 0.0}$	${}^{+ 1.2}_{-0.6}$	${}^{+ 0.6}_{-0.1}$	${}^{+0.1}_{-0.1}$	${}^{-0.0}_{-0.0}$	${}^{+ 1.5}_{-0.5}$	∓1.2	∓0.1	±1.4	±0.1	0.0	±1.4	${}^{+ 0.5}_{-0.5}$	±0.7
160–210	$1.00 ^{+ 0.01}_{-0.01}$	8.349e-02	9.1	${}^{+0.0}_{-0.0}$	${}^{+ 0.6}_{-1.9}$	${}^{+ 0.3}_{-1.5}$	${}^{+0.2}_{-1.4}$	${}^{+ 0.2}_{-0.7}$	${}^{+ 0.4}_{-1.5}$	${}^{+0.1}_{-1.2}$	${}^{-0.3}_{-0.3}$	${}^{+1.1}_{-1.8}$	${}^{+ 0.4}_{-1.7}$	${}^{-0.3}_{-0.2}$	${}^{+ 0.0}_{-0.0}$	${}^{+1.3}_{-2.2}$	∓1.3	±0.0	±0.3	±0.0	0.0	±1.4	${}^{+ 0.5}_{-0.5}$	±0.7
210–260	$1.00 ^{+ 0.02}_{-0.01}$	4.556e-02	22.	0.0	${}^{+0.7}_{-0.5}$	${}^{+ 0.6}_{-0.2}$	${}^{+ 0.4}_{-0.3}$	${}^{+0.8}_{-0.4}$	${}^{+ 0.7}_{-0.6}$	${}^{+ 0.3}_{-0.3}$	${}^{+ 0.4}_{+0.1}$	${}^{+1.4}_{-1.6}$	${}^{+ 0.8}_{-0.6}$	${}^{+ 0.3}_{+ 0.3}$	${}^{+ 0.0}_{-0.0}$	${}^{+1.5}_{-2.4}$	±1.3	∓0.1	±0.4	±0.0	0.0	±1.4	${}^{+ 0.5}_{-0.5}$	±0.7
260–310	$1.00 ^{+ 0.02}_{-0.01}$	4.087e-02	42.	${}^{-0.0}_{+0.0}$	${}^{+1.5}_{-1.4}$	${}^{+ 0.7}_{-0.7}$	${}^{-0.3}_{-0.1}$	${}^{+1.0}_{-0.8}$	${}^{+1.4}_{-1.4}$	${}^{+ 0.2}_{-0.4}$	${}^{+ 0.0}_{-0.1}$	${}^{+2.8}_{-2.6}$	${}^{+1.8}_{-1.6}$	${}^{-0.0}_{-0.1}$	${}^{-0.0}_{+0.0}$	${}^{+3.4}_{-3.5}$	∓2.0	∓0.0	±0.6	±0.0	0.0	±1.4	${}^{+ 0.5}_{-0.5}$	±0.4
310–400	$0.99 ^{+ 0.02}_{-0.01}$	1.199e-02	124	${}^{-0.5}_{+0.4}$	${}^{+2.2}_{-2.8}$	${}^{+1.6}_{-1.7}$	${}^{-0.1}_{-0.5}$	${}^{+1.8}_{-2.2}$	${}^{+2.6}_{-3.3}$	${}^{+ 0.6}_{-1.3}$	${}^{+0.1}_{-0.5}$	${}^{+4.0}_{-5.1}$	${}^{+3.5}_{-3.5}$	${}^{-0.3}_{-0.4}$	${}^{-0.1}_{+ 0.1}$	${}^{+5.5}_{-6.0}$	∓1.0	±0.2	±2.1	±0.0	0.0	±1.4	${}^{+ 0.5}_{-0.5}$	±0.5

**Table 35:** Measured jet cross section ratio in *p*_T_ bin for anti-*k*_*t*_ jets with *R*=0.4 in the rapidity bin 1.2≤|*y*|<2.1. See caption of Table 32 for details

*p*_T_ (GeV)	NPCorr	*ρ*	$\delta_{\mathrm{stat.}}$ %	*γ*_3_ %	*γ*_9_ %	*γ*_15_ %	*γ*_21_ %	*γ*_27_ %	*γ*_31_ %	*γ*_34_ %	*γ*_40_ %	*γ*_46_ %	*γ*_52_ %	*γ*_58_ %	*γ*_64_ %	*γ*_70_ %	*γ*_78_ %	*γ*_82_ %	*γ*_74_ %	*γ*_75_ %	*γ*_83_ %	*u*_1_ %	*u*_2_ %	*u*_3_ %
20–30	$0.91 ^{+ 0.02}_{-0.12}$	2.515e-01	1.3	${}^{+ 3.0}_{-0.1}$	${}^{-0.5}_{-0.8}$	${}^{+ 2.2}_{-0.1}$	${}^{+ 3.3}_{+ 0.2}$	${}^{+0.7}_{-0.1}$	${}^{+1.0}_{-1.4}$	${}^{+ 0.4}_{-0.4}$	${}^{+ 0.7}_{-0.3}$	${}^{+ 0.5}_{-0.5}$	${}^{-0.0}_{+ 0.0}$	${}^{+ 0.0}_{-0.0}$	${}^{+0.2}_{-0.3}$	${}^{-0.0}_{+ 0.0}$	∓1.5	∓0.0	±0.2	±1.1	0.0	±1.4	${}^{+ 0.7}_{-0.5}$	±1.3
30–45	$0.94 ^{+ 0.01}_{-0.07}$	2.259e-01	2.4	${}^{-0.4}_{+ 0.8}$	${}^{+1.6}_{-1.3}$	${}^{+ 0.2}_{-0.3}$	${}^{-1.3}_{+1.3}$	${}^{-0.0}_{-0.1}$	${}^{+1.4}_{-1.0}$	${}^{+ 0.6}_{-0.6}$	${}^{+0.3}_{-0.3}$	${}^{+ 0.7}_{-0.6}$	${}^{+ 0.0}_{-0.0}$	${}^{+0.1}_{-0.0}$	${}^{+ 0.5}_{-0.6}$	${}^{+ 0.0}_{-0.0}$	∓0.8	±0.3	±0.1	±0.9	0.0	±1.4	${}^{+ 0.6}_{-0.5}$	±1.0
45–60	$0.96 ^{+ 0.01}_{-0.04}$	1.841e-01	2.8	${}^{-0.3}_{+ 0.4}$	${}^{+ 0.6}_{-0.5}$	${}^{+ 0.2}_{-0.3}$	${}^{-0.5}_{+ 0.3}$	${}^{+0.3}_{-0.2}$	${}^{+ 0.4}_{-0.8}$	${}^{+ 0.4}_{-0.1}$	${}^{+0.3}_{-0.0}$	${}^{+ 0.6}_{-0.4}$	${}^{+ 0.1}_{-0.3}$	${}^{-0.3}_{-0.0}$	${}^{+ 0.2}_{+ 0.1}$	${}^{+ 0.1}_{-0.2}$	±0.0	±0.0	±0.3	±0.6	0.0	±1.4	${}^{+ 0.5}_{-0.5}$	±0.6
60–80	$0.98 ^{+ 0.01}_{-0.03}$	1.565e-01	1.2	${}^{-0.3}_{+ 0.2}$	${}^{-0.1}_{+ 0.1}$	${}^{-0.3}_{+ 0.4}$	${}^{+ 0.1}_{-0.1}$	${}^{-0.0}_{+ 0.1}$	${}^{-0.5}_{+ 0.3}$	${}^{-0.2}_{+ 0.1}$	${}^{-0.2}_{+0.4}$	${}^{-0.3}_{+ 0.1}$	${}^{+ 0.1}_{-0.3}$	${}^{-0.0}_{-0.1}$	${}^{-0.2}_{+ 0.2}$	${}^{+ 0.1}_{-0.2}$	∓0.7	∓0.0	±0.3	±0.4	0.0	±1.4	${}^{+ 0.5}_{-0.5}$	±0.5
80–110	$0.99 ^{+ 0.01}_{-0.03}$	1.229e-01	1.7	${}^{-0.0}_{-0.0}$	${}^{+ 0.7}_{-0.8}$	${}^{+ 0.5}_{-0.5}$	${}^{+ 0.3}_{-0.3}$	${}^{+0.2}_{+ 0.0}$	${}^{+ 0.8}_{-0.7}$	${}^{+ 0.3}_{-0.2}$	${}^{+ 0.2}_{+0.0}$	${}^{+ 0.6}_{-0.6}$	${}^{+ 0.3}_{-0.1}$	${}^{+ 0.1}_{+ 0.3}$	${}^{-0.1}_{+ 0.1}$	${}^{+ 0.7}_{-0.8}$	±0.5	±0.2	±0.3	±0.2	0.0	±1.4	${}^{+ 0.5}_{-0.5}$	±0.5
110–160	$1.00 ^{+ 0.01}_{-0.02}$	8.560e-02	2.9	${}^{-0.0}_{-0.0}$	${}^{+1.8}_{-1.0}$	${}^{+ 1.1}_{-0.9}$	${}^{+ 0.5}_{-0.0}$	${}^{+0.4}_{-0.3}$	${}^{+1.2}_{-1.2}$	${}^{+ 0.7}_{-0.5}$	${}^{+ 0.4}_{-0.2}$	${}^{+1.9}_{-1.5}$	${}^{+ 0.7}_{-0.6}$	${}^{+ 0.2}_{-0.1}$	${}^{-0.0}_{+ 0.0}$	${}^{+2.2}_{-1.5}$	∓1.2	∓0.0	±0.4	±0.0	0.0	±1.4	${}^{+ 0.5}_{-0.5}$	±0.6
160–210	$1.00 ^{+ 0.02}_{-0.02}$	6.069e-02	8.4	${}^{+0.0}_{-0.0}$	${}^{+1.4}_{-2.0}$	${}^{+1.0}_{-1.8}$	${}^{-0.1}_{-0.3}$	${}^{+ 0.6}_{-1.3}$	${}^{+1.8}_{-2.3}$	${}^{+ 0.5}_{-1.0}$	${}^{-0.3}_{-0.2}$	${}^{+2.2}_{-2.6}$	${}^{+ 0.9}_{-1.6}$	${}^{-0.3}_{-0.2}$	${}^{+ 0.0}_{-0.0}$	${}^{+2.6}_{-3.5}$	∓1.1	±0.1	±1.2	±0.0	0.0	±1.4	${}^{+ 0.5}_{-0.5}$	±0.5
210–260	$1.00 ^{+ 0.03}_{-0.01}$	2.678e-02	24.	${}^{+0.0}_{-0.0}$	${}^{+2.0}_{-1.8}$	${}^{+1.7}_{-1.0}$	${}^{+ 0.9}_{-0.8}$	${}^{+1.6}_{-1.1}$	${}^{+2.7}_{-3.2}$	${}^{+ 0.9}_{-0.7}$	${}^{+0.6}_{-0.1}$	${}^{+3.3}_{-3.5}$	${}^{+2.1}_{-1.8}$	${}^{+ 0.3}_{+ 0.2}$	${}^{+ 0.0}_{-0.0}$	${}^{+3.9}_{-4.5}$	∓2.2	±0.1	±1.4	±0.0	0.0	±1.4	${}^{+ 0.5}_{-0.5}$	±0.6
260–310	$0.99 ^{+ 0.03}_{-0.01}$	9.332e-03	77.	${}^{-0.0}_{+0.0}$	${}^{+2.9}_{-1.8}$	${}^{+2.0}_{-1.4}$	${}^{+1.1}_{-1.2}$	${}^{+2.2}_{-1.5}$	${}^{+4.5}_{-3.1}$	${}^{+ 1.0}_{-1.0}$	${}^{+0.2}_{-0.5}$	${}^{+5.9}_{-5.2}$	${}^{+3.5}_{-2.0}$	${}^{+ 0.1}_{-0.3}$	${}^{-0.0}_{+ 0.0}$	${}^{+6.5}_{-6.1}$	±0.1	±0.0	±0.7	±0.0	0.0	±1.4	${}^{+ 0.5}_{-0.5}$	±0.5
310–400	$0.99 ^{+ 0.03}_{-0.02}$	1.011e-02	122	${}^{-0.1}_{+0.1}$	${}^{+2.7}_{-4.4}$	${}^{+2.6}_{-3.2}$	${}^{+ 1.0}_{-2.3}$	${}^{+2.5}_{-3.6}$	${}^{+5.1}_{-7.3}$	${}^{+1.1}_{-2.3}$	${}^{+0.3}_{-0.7}$	${}^{+5.3}_{-7.6}$	${}^{+3.8}_{-4.9}$	${}^{-0.4}_{-0.7}$	${}^{-0.0}_{+ 0.0}$	${}^{+7.6}_{-9.4}$	∓1.9	∓0.0	±0.2	±0.0	0.0	±1.4	${}^{+ 0.5}_{-0.5}$	±0.4

**Table 36:** Measured jet cross section ratio in *p*_T_ bin for anti-*k*_*t*_ jets with *R*=0.4 in the rapidity bin 2.1≤|*y*|<2.8. See caption of Table 32 for details

*p*_T_ (GeV)	NPCorr	*ρ*	$\delta_{\mathrm{stat.}}$ %	*γ*_4_ %	*γ*_10_ %	*γ*_16_ %	*γ*_22_ %	*γ*_28_ %	*γ*_31_ %	*γ*_35_ %	*γ*_41_ %	*γ*_47_ %	*γ*_53_ %	*γ*_59_ %	*γ*_65_ %	*γ*_71_ %	*γ*_79_ %	*γ*_82_ %	*γ*_74_ %	*γ*_75_ %	*γ*_84_ %	*u*_1_ %	*u*_2_ %	*u*_3_ %
20–30	$0.92 ^{+ 0.02}_{-0.11}$	2.265e-01	2.0	${}^{+ 3.6}_{-0.2}$	${}^{-0.7}_{-0.7}$	${}^{+ 2.5}_{-0.3}$	${}^{+ 1.4}_{-0.2}$	${}^{+0.9}_{+ 0.2}$	${}^{+3.2}_{-2.3}$	${}^{+ 0.2}_{-0.2}$	${}^{+ 0.8}_{+0.0}$	${}^{+ 0.5}_{-0.1}$	${}^{-0.0}_{+ 0.0}$	${}^{+ 0.0}_{-0.0}$	${}^{+ 0.1}_{-0.1}$	${}^{-0.0}_{+ 0.0}$	∓2.6	±0.3	±0.1	±1.2	0.0	±1.4	${}^{+ 0.5}_{-0.5}$	±1.5
30–45	$0.95 ^{+ 0.01}_{-0.04}$	1.915e-01	2.0	${}^{-0.6}_{+ 0.8}$	${}^{+1.5}_{-1.2}$	${}^{+ 0.2}_{-0.4}$	${}^{-0.1}_{+ 0.0}$	${}^{-0.1}_{+ 0.0}$	${}^{+1.6}_{-1.6}$	${}^{+ 0.6}_{-0.4}$	${}^{+ 0.3}_{+0.0}$	${}^{+ 0.5}_{-0.3}$	${}^{+ 0.0}_{+ 0.0}$	${}^{+ 0.1}_{-0.0}$	${}^{+ 0.5}_{-0.2}$	${}^{+ 0.0}_{+ 0.0}$	∓0.0	±0.7	±0.3	±0.7	0.0	±1.4	${}^{+ 0.5}_{-0.5}$	±1.1
45–60	$0.97 ^{+ 0.02}_{-0.02}$	1.290e-01	3.9	${}^{-0.4}_{+ 0.1}$	${}^{+ 1.1}_{-1.0}$	${}^{+ 0.5}_{-0.6}$	${}^{+ 0.0}_{-0.4}$	${}^{+0.0}_{-0.4}$	${}^{+2.2}_{-1.3}$	${}^{-0.0}_{-0.6}$	${}^{+ 0.1}_{-0.3}$	${}^{+ 0.8}_{-0.9}$	${}^{-0.0}_{-0.4}$	${}^{-0.3}_{-0.0}$	${}^{-0.1}_{-0.1}$	${}^{+ 0.2}_{-0.5}$	±1.3	±0.0	±0.5	±0.1	0.0	±1.4	${}^{+ 0.5}_{-0.5}$	±0.7
60–80	$0.98 ^{+ 0.02}_{-0.01}$	1.055e-01	1.7	${}^{-0.3}_{+ 0.2}$	${}^{+ 1.2}_{-0.6}$	${}^{+ 0.7}_{-0.4}$	${}^{+ 0.3}_{+ 0.1}$	${}^{+0.6}_{-0.2}$	${}^{+1.1}_{-1.2}$	${}^{+ 0.7}_{-0.1}$	${}^{+ 0.3}_{+0.1}$	${}^{+ 1.0}_{-0.6}$	${}^{+ 0.4}_{-0.4}$	${}^{-0.0}_{-0.1}$	${}^{+0.0}_{+ 0.2}$	${}^{+ 0.9}_{-0.8}$	∓1.8	±0.2	±0.5	±0.3	0.0	±1.4	${}^{+ 0.5}_{-0.5}$	±0.6
80–110	$0.98 ^{+ 0.03}_{-0.00}$	6.313e-02	2.7	${}^{-0.0}_{+ 0.0}$	${}^{+3.9}_{-3.1}$	${}^{+3.3}_{-1.6}$	${}^{+ 0.6}_{-0.6}$	${}^{+0.7}_{-0.3}$	${}^{+3.4}_{-3.1}$	${}^{+ 2.4}_{-0.9}$	${}^{+ 0.7}_{-0.3}$	${}^{+3.5}_{-2.1}$	${}^{+ 1.0}_{-0.5}$	${}^{+ 0.2}_{+ 0.3}$	${}^{-0.1}_{+ 0.1}$	${}^{+3.5}_{-2.0}$	∓1.6	±0.8	±0.6	±0.1	0.0	±1.4	${}^{+ 0.5}_{-0.5}$	±0.9
110–160	$0.98 ^{+ 0.05}_{-0.00}$	2.256e-02	8.6	${}^{+0.0}_{-0.0}$	${}^{+4.1}_{-4.3}$	${}^{+2.7}_{-3.0}$	${}^{+ 0.6}_{-0.6}$	${}^{+ 0.8}_{-0.8}$	${}^{+4.5}_{-4.6}$	${}^{+1.9}_{-1.4}$	${}^{+0.5}_{-0.5}$	${}^{+4.1}_{-4.7}$	${}^{+1.1}_{-1.4}$	${}^{+ 0.1}_{-0.0}$	${}^{-0.0}_{+ 0.0}$	${}^{+4.4}_{-4.9}$	±0.1	∓0.0	±0.9	±0.0	0.0	±1.4	${}^{+ 0.5}_{-0.5}$	±0.8
160–210	$0.98 ^{+ 0.07}_{-0.01}$	8.884e-03	39.	${}^{-0.0}_{-0.0}$	${}^{+4.3}_{-6.8}$	${}^{+2.3}_{-6.5}$	${}^{+ 0.5}_{-1.5}$	${}^{+1.0}_{-2.7}$	${}^{+7.0}_{-11.}$	${}^{+1.7}_{-2.2}$	${}^{-0.2}_{-0.7}$	${}^{+6.1}_{-11.}$	${}^{+3.1}_{-5.5}$	${}^{-0.2}_{-0.3}$	${}^{-0.0}_{+0.0}$	${}^{+7.7}_{-13.}$	±0.6	±0.9	±1.9	±0.0	0.0	±1.4	${}^{+ 0.5}_{-0.5}$	±1.0
210–260	$0.97 ^{+ 0.07}_{-0.02}$	6.248e-03	113	0.0	${}^{+5.6}_{-3.5}$	${}^{+2.9}_{-1.3}$	${}^{-1.6}_{-0.6}$	${}^{+2.0}_{-2.8}$	${}^{ +12}_{ -16}$	${}^{-1.7}_{-1.2}$	${}^{-0.8}_{-1.3}$	${}^{+7.0}_{-17.}$	${}^{+4.2}_{-4.2}$	${}^{+0.1}_{-0.7}$	${}^{+ 0.0}_{-0.0}$	${}^{ +21}_{ -20}$	∓4.2	±3.1	±11.5	±0.0	0.0	±1.4	${}^{+ 0.5}_{-0.5}$	±0.8

**Table 37:** Measured jet cross section ratio in *p*_T_ bin for anti-*k*_*t*_ jets with *R*=0.4 in the rapidity bin 2.8≤|*y*|<3.6. See caption of Table 32 for details

*p*_T_ (GeV)	NPCorr	*ρ*	$\delta_{\mathrm{stat.}}$ %	*γ*_5_ %	*γ*_11_ %	*γ*_17_ %	*γ*_23_ %	*γ*_29_ %	*γ*_31_ %	*γ*_36_ %	*γ*_42_ %	*γ*_48_ %	*γ*_54_ %	*γ*_60_ %	*γ*_66_ %	*γ*_72_ %	*γ*_80_ %	*γ*_82_ %	*γ*_74_ %	*γ*_75_ %	*γ*_85_ %	*u*_1_ %	*u*_2_ %	*u*_3_ %
20–30	$0.92 ^{+ 0.02}_{-0.08}$	1.654e-01	2.4	${}^{+3.7}_{-1.0}$	${}^{-0.4}_{-2.0}$	${}^{+2.7}_{-1.2}$	${}^{-5.2}_{+6.8}$	${}^{+0.8}_{-0.5}$	${}^{+4.7}_{-5.2}$	${}^{+ 0.8}_{-0.6}$	${}^{+ 0.9}_{-0.7}$	${}^{+ 0.7}_{-1.0}$	${}^{+ 0.0}_{+ 0.0}$	${}^{+ 0.1}_{-0.0}$	${}^{+0.5}_{-0.6}$	${}^{-0.0}_{+ 0.0}$	∓4.1	∓0.2	±0.2	±0.7	0.0	±2.2	${}^{+ 0.5}_{-0.5}$	±4.8
30–45	$0.94 ^{+ 0.03}_{-0.03}$	1.056e-01	2.8	${}^{-0.7}_{+ 1.1}$	${}^{+2.9}_{-2.8}$	${}^{+ 1.1}_{-0.9}$	${}^{-3.6}_{+3.9}$	${}^{-0.0}_{+0.0}$	${}^{+7.7}_{-8.1}$	${}^{+ 0.8}_{-0.6}$	${}^{+ 0.5}_{-0.0}$	${}^{+1.5}_{-0.6}$	${}^{+ 0.1}_{-0.0}$	${}^{+ 0.1}_{+ 0.0}$	${}^{+0.8}_{-0.4}$	${}^{+ 0.1}_{-0.0}$	±0.6	±0.2	±0.5	±1.0	0.0	±2.2	${}^{+ 0.5}_{-0.5}$	±3.4
45–60	$0.95 ^{+ 0.05}_{-0.01}$	5.306e-02	4.4	${}^{-0.1}_{+ 0.4}$	${}^{+3.6}_{-4.2}$	${}^{+2.5}_{-3.5}$	${}^{-1.1}_{+1.7}$	${}^{+0.9}_{-0.8}$	${}^{+11.}_{-9.6}$	${}^{+1.3}_{-1.3}$	${}^{+ 0.8}_{-0.5}$	${}^{+2.6}_{-2.8}$	${}^{-0.1}_{-0.3}$	${}^{-0.4}_{-0.0}$	${}^{+1.0}_{-0.4}$	${}^{+ 0.5}_{-0.7}$	∓1.9	±0.6	±2.2	±0.2	0.0	±1.4	${}^{+ 0.5}_{-0.5}$	±2.2
60–80	$0.95 ^{+ 0.06}_{-0.01}$	2.070e-02	4.2	${}^{-0.9}_{+ 0.7}$	${}^{+4.2}_{-7.0}$	${}^{+2.9}_{-5.4}$	${}^{-0.6}_{-0.8}$	${}^{+1.3}_{-1.9}$	${}^{+8.1}_{-11.}$	${}^{+2.3}_{-3.3}$	${}^{+1.0}_{-0.6}$	${}^{+3.4}_{-6.0}$	${}^{+ 1.3}_{-0.7}$	${}^{+ 0.0}_{-0.1}$	${}^{+ 0.0}_{+ 0.5}$	${}^{+2.6}_{-3.5}$	∓6.5	±0.9	±4.4	±0.3	0.0	±1.4	${}^{+ 0.5}_{-0.5}$	±3.9
80–110	$0.95 ^{+ 0.06}_{-0.01}$	3.470e-03	15.	${}^{-0.3}_{+ 0.2}$	${}^{+2.9}_{-8.8}$	${}^{+1.1}_{-6.9}$	${}^{-1.5}_{-4.4}$	${}^{+0.4}_{-2.1}$	${}^{+4.8}_{-13.}$	${}^{+1.8}_{-6.6}$	${}^{+ 0.6}_{-2.2}$	${}^{+1.4}_{-8.3}$	${}^{+1.4}_{-2.7}$	${}^{+ 0.1}_{+ 0.4}$	${}^{-0.7}_{+ 0.4}$	${}^{+2.9}_{-8.8}$	∓0.4	±1.7	±2.5	±0.7	0.0	±1.4	${}^{+ 0.5}_{-0.5}$	±4.8
110–160	$0.94 ^{+ 0.06}_{-0.02}$	1.298e-03	69.	${}^{-0.1}_{+0.0}$	${}^{-4.9}_{-21.}$	${}^{-4.3}_{-9.6}$	${}^{-5.5}_{-8.3}$	${}^{-2.4}_{-3.5}$	${}^{ +14}_{ -33}$	${}^{-1.8}_{-3.5}$	${}^{-2.1}_{-4.2}$	${}^{-6.3}_{-23.}$	${}^{-2.7}_{-3.3}$	${}^{+ 0.0}_{+0.0}$	${}^{-0.3}_{+ 0.1}$	${}^{-5.0}_{-21.}$	∓26	∓0.1	±26.5	±0.2	0.0	±1.4	${}^{+ 0.5}_{-0.5}$	±2.8

**Table 38:** Measured jet cross section ratio in *p*_T_ bin for anti-*k*_*t*_ jets with *R*=0.4 in the rapidity bin 3.6≤|*y*|<4.4. See caption of Table 32 for details

*p*_T_ (GeV)	NPCorr	*ρ*	$\delta_{\mathrm{stat.}}$ %	*γ*_6_ %	*γ*_12_ %	*γ*_18_ %	*γ*_24_ %	*γ*_30_ %	*γ*_31_ %	*γ*_37_ %	*γ*_43_ %	*γ*_49_ %	*γ*_55_ %	*γ*_61_ %	*γ*_67_ %	*γ*_73_ %	*γ*_81_ %	*γ*_82_ %	*γ*_74_ %	*γ*_75_ %	*γ*_86_ %	*u*_1_ %	*u*_2_ %	*u*_3_ %
20–30	$0.86 ^{+ 0.10}_{-0.00}$	5.680e-02	5.9	${}^{+10.}_{-4.0}$	${}^{+3.0}_{-6.0}$	${}^{+8.7}_{-4.7}$	${}^{+2.0}_{-1.6}$	${}^{+2.6}_{-2.2}$	${}^{ +29}_{ -31}$	${}^{+1.1}_{-1.9}$	${}^{+2.6}_{-2.6}$	${}^{+2.6}_{-3.5}$	${}^{+ 0.0}_{+ 0.0}$	${}^{+0.0}_{-0.0}$	${}^{+1.4}_{-2.0}$	${}^{+ 0.0}_{-0.0}$	∓13	∓0.9	±0.3	±1.5	0.0	±2.2	${}^{+ 0.5}_{-0.5}$	±6.0
30–45	$0.86 ^{+ 0.12}_{-0.03}$	1.539e-02	6.5	${}^{+1.9}_{-7.8}$	${}^{ +13}_{ -18}$	${}^{+7.1}_{-12.}$	${}^{+1.5}_{-2.2}$	${}^{+2.9}_{-3.5}$	${}^{ +76}_{ -55}$	${}^{+4.2}_{-5.2}$	${}^{+3.4}_{-9.6}$	${}^{+5.3}_{-11.}$	${}^{+ 0.0}_{-0.0}$	${}^{+0.0}_{-0.0}$	${}^{+4.0}_{-10.}$	${}^{+ 0.1}_{-0.1}$	∓12	±1.4	±3.0	±2.0	0.0	±1.4	${}^{+ 0.5}_{-0.5}$	±10.4
45–60	$0.85 ^{+ 0.11}_{-0.05}$	1.034e-03	18.	${}^{-1.3}_{-3.0}$	${}^{ +16}_{ -15}$	${}^{+7.7}_{-10.}$	${}^{+ 0.7}_{-3.4}$	${}^{+1.4}_{-5.2}$	${}^{ +92}_{ -62}$	${}^{+3.4}_{-6.3}$	${}^{+2.2}_{-5.1}$	${}^{+7.8}_{-9.6}$	${}^{-0.3}_{-1.0}$	${}^{-0.4}_{-0.0}$	${}^{+2.1}_{-5.0}$	${}^{+1.2}_{-2.2}$	∓15	±1.4	±15.1	±0.8	0.0	±1.4	${}^{+0.5}_{-0.5}$	±6.4

**Table 39:** Measured jet cross section ratio in *p*_T_ bin for anti-*k*_*t*_ jets with *R*=0.6 in the rapidity bin |*y*|<0.3. See caption of Table 32 for details

*p*_T_ (GeV)	NPCorr	*ρ*	$\delta_{\mathrm{stat.}}$ %	*γ*_1_ %	*γ*_7_ %	*γ*_13_ %	*γ*_19_ %	*γ*_25_ %	*γ*_31_ %	*γ*_32_ %	*γ*_38_ %	*γ*_44_ %	*γ*_50_ %	*γ*_56_ %	*γ*_62_ %	*γ*_68_ %	*γ*_76_ %	*γ*_82_ %	*γ*_74_ %	*γ*_75_ %	*γ*_83_ %	*u*_1_ %	*u*_2_ %	*u*_3_ %
20–30	$0.77 ^{+ 0.03}_{-0.17}$	2.530e-01	1.5	${}^{+ 1.4}_{+ 0.3}$	${}^{+ 0.6}_{+ 0.0}$	${}^{+ 3.1}_{+ 0.3}$	${}^{+ 0.3}_{-0.3}$	${}^{+1.5}_{+ 0.4}$	0.0	${}^{-0.1}_{+ 0.0}$	${}^{+ 0.5}_{+ 0.1}$	${}^{+0.4}_{+ 0.3}$	${}^{+ 0.0}_{+ 0.0}$	${}^{+ 0.0}_{-0.0}$	${}^{-0.3}_{-0.2}$	${}^{-0.0}_{+ 0.0}$	∓1.9	±0.2	±0.1	±1.4	0.0	±1.4	${}^{+ 0.7}_{-0.5}$	±0.8
30–45	$0.86 ^{+ 0.02}_{-0.08}$	2.441e-01	2.7	${}^{+ 0.1}_{+ 0.0}$	${}^{+1.6}_{-1.1}$	${}^{+ 0.4}_{+ 0.1}$	${}^{+ 0.7}_{-0.6}$	${}^{-0.7}_{+ 0.3}$	0.0	${}^{+ 0.1}_{-0.3}$	${}^{-0.2}_{-0.1}$	${}^{+0.6}_{-0.4}$	${}^{+ 0.0}_{-0.0}$	${}^{+ 0.0}_{-0.1}$	${}^{+0.5}_{-0.6}$	${}^{-0.0}_{+ 0.0}$	∓0.6	±0.1	±0.1	±0.8	0.0	±1.4	${}^{+ 0.6}_{-0.5}$	±0.6
45–60	$0.90 ^{+ 0.02}_{-0.04}$	2.162e-01	3.4	${}^{-0.8}_{-0.1}$	${}^{-0.6}_{-0.4}$	${}^{-1.0}_{-0.1}$	${}^{-0.7}_{-0.3}$	${}^{-0.4}_{-0.5}$	0.0	${}^{-0.4}_{-0.3}$	${}^{-0.8}_{-0.2}$	${}^{-0.5}_{-0.5}$	${}^{-0.2}_{-0.3}$	${}^{-0.4}_{-0.0}$	${}^{-0.6}_{-0.4}$	${}^{-0.0}_{-0.0}$	∓1.2	∓0.1	±0.1	±1.0	0.0	±1.4	${}^{+ 0.6}_{-0.5}$	±0.4
60–80	$0.92 ^{+ 0.01}_{-0.02}$	1.851e-01	1.4	${}^{-0.2}_{+ 0.1}$	${}^{+ 0.1}_{-1.6}$	${}^{+ 0.3}_{-0.4}$	${}^{+ 0.4}_{-1.0}$	${}^{+0.3}_{-0.5}$	0.0	${}^{+ 0.2}_{-0.8}$	${}^{+ 0.1}_{-0.4}$	${}^{+0.7}_{-1.5}$	${}^{+ 0.1}_{-0.4}$	${}^{+ 0.0}_{-0.1}$	${}^{+0.4}_{-1.0}$	${}^{-0.0}_{-0.1}$	±1.0	±0.1	±0.2	±0.4	0.0	±1.4	${}^{+ 0.5}_{-0.5}$	±0.3
80–110	$0.95 ^{+ 0.01}_{-0.01}$	1.604e-01	1.7	${}^{+ 0.1}_{-0.0}$	${}^{+ 0.8}_{-0.4}$	${}^{+ 0.5}_{+ 0.1}$	${}^{+ 0.9}_{-0.1}$	${}^{+0.2}_{+ 0.4}$	0.0	${}^{+ 0.6}_{-0.2}$	${}^{+ 0.5}_{+ 0.3}$	${}^{+0.6}_{-0.3}$	${}^{+ 0.5}_{+ 0.1}$	${}^{+ 0.2}_{+ 0.3}$	${}^{+ 0.1}_{+0.3}$	${}^{+ 0.5}_{+ 0.1}$	∓1.0	∓0.0	±0.1	±0.3	0.0	±1.4	${}^{+ 0.5}_{-0.5}$	±0.6
110–160	$0.97 ^{+ 0.01}_{-0.01}$	1.323e-01	2.9	${}^{+0.0}_{-0.0}$	${}^{+ 1.0}_{-0.1}$	${}^{+ 0.5}_{-0.2}$	${}^{+0.1}_{-0.1}$	${}^{+ 0.0}_{-0.0}$	0.0	${}^{-0.0}_{-0.0}$	${}^{-0.2}_{+0.2}$	${}^{+ 0.9}_{-0.3}$	${}^{-0.1}_{-0.2}$	${}^{+ 0.2}_{+ 0.2}$	${}^{-0.2}_{+ 0.2}$	${}^{+ 0.1}_{-0.5}$	∓0.4	∓0.0	±0.3	±0.1	0.0	±1.4	${}^{+ 0.5}_{-0.5}$	±0.4
160–210	$0.98 ^{+ 0.01}_{-0.01}$	9.933e-02	7.9	${}^{+0.0}_{-0.0}$	${}^{+1.0}_{-1.2}$	${}^{+ 0.3}_{-1.0}$	${}^{-0.2}_{-0.3}$	${}^{-0.1}_{-0.3}$	0.0	${}^{+ 0.3}_{-0.4}$	${}^{+ 0.0}_{-0.2}$	${}^{+0.9}_{-1.1}$	${}^{+ 0.1}_{-0.7}$	${}^{-0.1}_{-0.5}$	${}^{-0.0}_{-0.0}$	${}^{+ 0.6}_{-1.3}$	∓0.1	±0.0	±0.4	±0.1	0.0	±1.4	${}^{+ 0.5}_{-0.5}$	±0.4
210–260	$0.99 ^{+ 0.01}_{-0.01}$	6.966e-02	17.	${}^{+0.0}_{-0.0}$	${}^{+ 1.4}_{-0.6}$	${}^{+ 0.9}_{-0.3}$	${}^{+0.6}_{-0.1}$	${}^{+ 0.8}_{-0.3}$	0.0	${}^{+ 0.8}_{-0.2}$	${}^{+0.2}_{-0.1}$	${}^{+ 1.8}_{-0.6}$	${}^{+ 1.5}_{-0.5}$	${}^{+0.1}_{-0.2}$	${}^{+ 0.0}_{-0.0}$	${}^{+ 1.9}_{-0.9}$	∓1.0	±0.1	±0.4	±0.1	0.0	±1.4	${}^{+ 0.5}_{-0.5}$	±0.3
260–310	$0.99 ^{+ 0.01}_{-0.01}$	6.273e-02	34.	${}^{-0.0}_{+0.0}$	${}^{-0.3}_{-0.8}$	${}^{+ 0.3}_{-0.4}$	${}^{+ 0.0}_{-0.5}$	${}^{+0.4}_{-0.9}$	0.0	${}^{+ 0.1}_{-0.9}$	${}^{+ 0.2}_{-0.5}$	${}^{+1.0}_{-1.4}$	${}^{+ 0.3}_{-1.1}$	${}^{-0.1}_{-0.2}$	${}^{+0.0}_{-0.0}$	${}^{+1.0}_{-1.7}$	±0.1	±0.0	±0.4	±0.3	0.0	±1.4	${}^{+ 0.5}_{-0.5}$	±0.3
310–400	$1.00 ^{+ 0.01}_{-0.01}$	5.772e-02	47.	0.0	${}^{+0.9}_{-1.0}$	${}^{+ 0.9}_{-1.2}$	${}^{+ 0.0}_{-0.2}$	${}^{+1.2}_{-1.0}$	0.0	${}^{+ 0.4}_{-0.6}$	${}^{-0.1}_{-0.4}$	${}^{+2.3}_{-1.8}$	${}^{+1.5}_{-1.9}$	${}^{-0.1}_{-0.2}$	${}^{+0.0}_{-0.0}$	${}^{+3.0}_{-2.1}$	±0.3	±0.1	±0.3	±0.2	0.0	±1.4	${}^{+ 0.5}_{-0.5}$	±0.3
400–500	$1.00 ^{+ 0.01}_{-0.01}$	4.224e-02	120	${}^{+0.0}_{-0.0}$	${}^{+ 2.0}_{-0.1}$	${}^{+ 1.5}_{-0.1}$	${}^{+ 0.9}_{+0.1}$	${}^{+ 1.5}_{-0.5}$	0.0	${}^{+ 1.7}_{-0.0}$	${}^{+ 0.4}_{+0.4}$	${}^{+3.1}_{-2.5}$	${}^{+2.9}_{-1.6}$	${}^{+ 0.6}_{+ 0.1}$	0.0	${}^{+3.7}_{-3.2}$	∓0.9	±0.1	±0.5	±0.0	0.0	±1.4	${}^{+ 0.5}_{-0.5}$	±0.2

**Table 40:** Measured jet cross section ratio in *p*_T_ bin for anti-*k*_*t*_ jets with *R*=0.6 in the rapidity bin 0.3≤|*y*|<0.8. See caption of Table 32 for details

*p*_T_ (GeV)	NPCorr	*ρ*	$\delta_{\mathrm{stat.}}$ %	*γ*_1_ %	*γ*_7_ %	*γ*_13_ %	*γ*_19_ %	*γ*_25_ %	*γ*_31_ %	*γ*_32_ %	*γ*_38_ %	*γ*_44_ %	*γ*_50_ %	*γ*_56_ %	*γ*_62_ %	*γ*_68_ %	*γ*_76_ %	*γ*_82_ %	*γ*_74_ %	*γ*_75_ %	*γ*_83_ %	*u*_1_ %	*u*_2_ %	*u*_3_ %
20–30	$0.77 ^{+ 0.03}_{-0.17}$	2.458e-01	1.2	${}^{+ 2.0}_{+ 0.0}$	${}^{+ 1.1}_{-0.2}$	${}^{+ 3.0}_{+ 0.2}$	${}^{+ 0.7}_{-0.1}$	${}^{+0.6}_{-0.1}$	${}^{+ 0.2}_{-0.2}$	${}^{+ 0.1}_{-0.3}$	${}^{+0.4}_{-0.1}$	${}^{+ 0.2}_{-0.3}$	${}^{+ 0.0}_{+ 0.0}$	${}^{+0.1}_{-0.0}$	${}^{-0.0}_{-0.1}$	${}^{-0.0}_{+ 0.0}$	∓1.8	±0.1	±0.2	±1.1	0.0	±1.4	${}^{+ 0.7}_{-0.5}$	±0.8
30–45	$0.87 ^{+ 0.01}_{-0.13}$	2.414e-01	2.1	${}^{-0.5}_{+ 0.4}$	${}^{+ 0.7}_{-0.8}$	${}^{-0.4}_{+ 0.1}$	${}^{+ 0.0}_{-0.3}$	${}^{-0.2}_{-0.3}$	${}^{+ 0.1}_{-0.2}$	${}^{+ 0.3}_{-0.6}$	${}^{-0.1}_{-0.2}$	${}^{+ 0.2}_{-0.4}$	${}^{+ 0.0}_{-0.0}$	${}^{+0.1}_{-0.0}$	${}^{+ 0.1}_{-0.4}$	${}^{+ 0.0}_{+ 0.0}$	∓0.4	±0.6	±0.1	±0.8	0.0	±1.4	${}^{+ 0.6}_{-0.5}$	±0.6
45–60	$0.91 ^{+ 0.01}_{-0.09}$	2.013e-01	2.7	${}^{-0.8}_{+ 0.0}$	${}^{-0.2}_{-0.7}$	${}^{-0.5}_{-0.7}$	${}^{-0.6}_{-0.4}$	${}^{-0.3}_{-0.6}$	${}^{+ 0.1}_{-0.1}$	${}^{-0.5}_{-0.5}$	${}^{-0.3}_{-0.4}$	${}^{-0.2}_{-0.8}$	${}^{-0.1}_{-0.2}$	${}^{-0.4}_{-0.0}$	${}^{-0.3}_{-0.3}$	${}^{-0.2}_{-0.2}$	±0.5	∓0.1	±0.4	±0.5	0.0	±1.4	${}^{+ 0.6}_{-0.5}$	±0.4
60–80	$0.93 ^{+ 0.01}_{-0.06}$	1.823e-01	1.1	${}^{-0.1}_{+ 0.1}$	${}^{+ 0.1}_{-0.6}$	${}^{-0.2}_{-0.2}$	${}^{+ 0.3}_{-0.3}$	${}^{+0.1}_{-0.1}$	${}^{+ 0.1}_{-0.1}$	${}^{+ 0.2}_{-0.3}$	${}^{+0.1}_{-0.0}$	${}^{+ 0.0}_{-0.5}$	${}^{-0.0}_{-0.3}$	${}^{-0.0}_{-0.1}$	${}^{-0.3}_{+ 0.1}$	${}^{+ 0.4}_{-0.5}$	±0.0	∓0.0	±0.3	±0.3	0.0	±1.4	${}^{+ 0.5}_{-0.5}$	±0.3
80–110	$0.95 ^{+ 0.01}_{-0.04}$	1.585e-01	1.2	${}^{+ 0.1}_{+0.0}$	${}^{+ 1.0}_{-0.2}$	${}^{+ 0.4}_{+ 0.1}$	${}^{+ 0.4}_{+ 0.0}$	${}^{+ 0.3}_{+ 0.1}$	${}^{+ 0.0}_{-0.1}$	${}^{+ 0.5}_{+ 0.0}$	${}^{+0.1}_{+ 0.1}$	${}^{+ 0.8}_{-0.0}$	${}^{+ 0.1}_{+ 0.1}$	${}^{+ 0.1}_{+0.4}$	${}^{-0.0}_{+ 0.1}$	${}^{+ 0.8}_{-0.3}$	∓0.7	±0.0	±0.4	±0.3	0.0	±1.4	${}^{+ 0.5}_{-0.5}$	±0.5
110–160	$0.97 ^{+ 0.00}_{-0.03}$	1.266e-01	2.4	${}^{+0.0}_{-0.0}$	${}^{+ 0.7}_{-0.8}$	${}^{+ 0.6}_{-0.4}$	${}^{+0.4}_{-0.1}$	${}^{+ 0.1}_{-0.2}$	${}^{+ 0.1}_{-0.0}$	${}^{+0.2}_{-0.3}$	${}^{+ 0.1}_{-0.2}$	${}^{+ 0.7}_{-1.0}$	${}^{+0.4}_{-0.4}$	${}^{+ 0.1}_{-0.1}$	${}^{+ 0.0}_{-0.0}$	${}^{+0.9}_{-0.9}$	±0.0	±0.2	±0.8	±0.1	0.0	±1.4	${}^{+ 0.5}_{-0.5}$	±0.5
160–210	$0.99 ^{+ 0.01}_{-0.02}$	9.318e-02	6.6	${}^{+0.0}_{-0.0}$	${}^{-0.1}_{-0.4}$	${}^{+ 0.2}_{-0.5}$	${}^{-0.0}_{-0.5}$	${}^{+ 0.2}_{-0.7}$	${}^{+ 0.1}_{-0.0}$	${}^{-0.0}_{-0.4}$	${}^{-0.3}_{-0.2}$	${}^{+ 0.7}_{-1.3}$	${}^{+ 0.2}_{-0.9}$	${}^{-0.4}_{-0.2}$	${}^{+ 0.0}_{-0.0}$	${}^{+ 1.0}_{-1.9}$	∓0.8	±0.1	±0.3	±0.1	0.0	±1.4	${}^{+ 0.5}_{-0.5}$	±0.5
210–260	$0.99 ^{+ 0.01}_{-0.01}$	4.872e-02	17.	${}^{+0.0}_{-0.0}$	${}^{+ 0.7}_{-0.1}$	${}^{+ 0.7}_{-0.1}$	${}^{+0.5}_{-0.1}$	${}^{+ 0.8}_{-0.2}$	${}^{+ 0.0}_{-0.0}$	${}^{+0.4}_{-0.3}$	${}^{+ 0.5}_{+ 0.1}$	${}^{+ 1.4}_{-0.8}$	${}^{+0.7}_{-0.2}$	${}^{+ 0.2}_{+ 0.2}$	${}^{+ 0.0}_{-0.0}$	${}^{+1.4}_{-0.9}$	±0.2	±0.1	±0.3	±0.1	0.0	±1.4	${}^{+ 0.5}_{-0.5}$	±0.4
260–310	$1.00 ^{+ 0.01}_{-0.01}$	4.947e-02	29.	${}^{+0.0}_{-0.0}$	${}^{+1.1}_{-1.7}$	${}^{+ 1.0}_{-0.9}$	${}^{+ 0.6}_{-0.2}$	${}^{+1.3}_{-1.2}$	${}^{+ 0.0}_{-0.0}$	${}^{+ 0.2}_{-0.6}$	${}^{-0.1}_{-0.1}$	${}^{+1.9}_{-1.7}$	${}^{+1.5}_{-1.4}$	${}^{-0.0}_{-0.1}$	${}^{+ 0.0}_{-0.0}$	${}^{+2.2}_{-2.0}$	±0.2	±0.1	±0.5	±0.1	0.0	±1.4	${}^{+ 0.5}_{-0.5}$	±0.3
310–400	$1.00 ^{+ 0.01}_{-0.01}$	5.279e-02	45.	${}^{+0.0}_{-0.0}$	${}^{+1.2}_{-1.7}$	${}^{+1.2}_{-1.5}$	${}^{-0.0}_{-0.5}$	${}^{+ 0.8}_{-1.3}$	${}^{+ 0.0}_{-0.0}$	${}^{+ 0.5}_{-0.9}$	${}^{+0.2}_{-0.2}$	${}^{+1.9}_{-2.8}$	${}^{+1.5}_{-1.8}$	${}^{-0.3}_{-0.3}$	${}^{+ 0.0}_{-0.0}$	${}^{+2.8}_{-3.5}$	±0.4	±0.0	±0.4	±0.2	0.0	±1.4	${}^{+ 0.5}_{-0.5}$	±0.3
400–500	$1.00 ^{+ 0.01}_{-0.01}$	9.406e-02	63.	0.0	${}^{+2.5}_{-1.4}$	${}^{+ 1.6}_{-0.9}$	${}^{+ 0.5}_{+ 0.1}$	${}^{+1.4}_{-0.6}$	${}^{+ 0.0}_{-0.0}$	${}^{+ 1.0}_{-0.3}$	${}^{+0.2}_{-0.1}$	${}^{+3.7}_{-3.3}$	${}^{+3.2}_{-2.3}$	${}^{+ 0.5}_{+ 0.0}$	${}^{+ 0.0}_{-0.0}$	${}^{+4.6}_{-6.0}$	∓0.3	∓0.1	±0.7	±0.2	0.0	±1.4	${}^{+ 0.5}_{-0.5}$	±0.2

**Table 41:** Measured jet cross section ratio in *p*_T_ bin for anti-*k*_*t*_ jets with *R*=0.6 in the rapidity bin 0.8≤|*y*|<1.2. See caption of Table 32 for details

*p*_T_ (GeV)	NPCorr	*ρ*	$\delta_{\mathrm{stat.}}$ %	*γ*_2_ %	*γ*_8_ %	*γ*_14_ %	*γ*_20_ %	*γ*_26_ %	*γ*_31_ %	*γ*_33_ %	*γ*_39_ %	*γ*_45_ %	*γ*_51_ %	*γ*_57_ %	*γ*_63_ %	*γ*_69_ %	*γ*_77_ %	*γ*_82_ %	*γ*_74_ %	*γ*_75_ %	*γ*_83_ %	*u*_1_ %	*u*_2_ %	*u*_3_ %
20–30	$0.79 ^{+ 0.03}_{-0.17}$	2.412e-01	1.4	${}^{+ 1.3}_{+ 0.2}$	${}^{+ 0.6}_{-0.1}$	${}^{+ 2.1}_{+ 0.3}$	${}^{+ 2.0}_{+ 0.3}$	${}^{+0.7}_{+ 0.0}$	${}^{+ 0.8}_{-0.9}$	${}^{+ 0.1}_{-0.2}$	${}^{+0.7}_{-0.1}$	${}^{+ 0.5}_{-0.2}$	${}^{+ 0.0}_{+ 0.0}$	${}^{+0.0}_{-0.0}$	${}^{+ 0.1}_{-0.1}$	${}^{-0.0}_{+ 0.0}$	∓1.8	∓0.1	±0.3	±1.6	0.0	±1.4	${}^{+ 0.6}_{-0.5}$	±1.1
30–45	$0.88 ^{+ 0.02}_{-0.12}$	2.224e-01	2.5	${}^{-0.1}_{-0.0}$	${}^{+ 1.1}_{-0.6}$	${}^{+ 0.0}_{+ 0.3}$	${}^{-0.3}_{+ 0.0}$	${}^{-0.0}_{-0.4}$	${}^{+1.2}_{-1.4}$	${}^{+ 0.4}_{-0.7}$	${}^{+0.2}_{-0.3}$	${}^{+ 0.6}_{-0.7}$	${}^{+ 0.0}_{-0.1}$	${}^{+0.1}_{-0.1}$	${}^{+ 0.4}_{-0.6}$	${}^{+ 0.0}_{+ 0.0}$	±0.6	∓0.0	±0.1	±0.5	0.0	±1.4	${}^{+ 0.6}_{-0.5}$	±1.1
45–60	$0.92 ^{+ 0.01}_{-0.08}$	2.001e-01	3.2	${}^{-0.6}_{-0.2}$	${}^{-0.3}_{-0.3}$	${}^{-0.4}_{-0.2}$	${}^{-0.8}_{-0.7}$	${}^{-0.1}_{-0.9}$	${}^{-0.3}_{-0.7}$	${}^{-0.2}_{-0.6}$	${}^{-0.1}_{-0.7}$	${}^{-0.2}_{-0.5}$	${}^{-0.1}_{-0.3}$	${}^{-0.3}_{-0.0}$	${}^{-0.2}_{-0.6}$	${}^{-0.1}_{-0.3}$	±1.3	±0.0	±0.6	±0.3	0.0	±1.4	${}^{+ 0.6}_{-0.5}$	±0.7
60–80	$0.94 ^{+ 0.01}_{-0.06}$	1.703e-01	1.3	${}^{-0.1}_{+ 0.1}$	${}^{-0.0}_{-0.3}$	${}^{-0.4}_{-0.2}$	${}^{-0.1}_{+ 0.1}$	${}^{-0.2}_{-0.3}$	${}^{+ 0.0}_{-0.6}$	${}^{+ 0.0}_{-0.3}$	${}^{+0.0}_{-0.1}$	${}^{-0.0}_{-0.6}$	${}^{+ 0.1}_{-0.3}$	${}^{+ 0.0}_{-0.1}$	${}^{-0.1}_{+ 0.2}$	${}^{+ 0.4}_{-0.5}$	∓1.4	±0.2	±0.5	±0.2	0.0	±1.4	${}^{+ 0.6}_{-0.5}$	±0.6
80–110	$0.96 ^{+ 0.01}_{-0.04}$	1.462e-01	1.5	${}^{+ 0.0}_{+0.0}$	${}^{+ 0.7}_{-0.0}$	${}^{+ 0.6}_{-0.1}$	${}^{+ 0.5}_{+ 0.3}$	${}^{+ 0.5}_{+ 0.4}$	${}^{+ 0.6}_{-0.1}$	${}^{+ 0.5}_{+ 0.1}$	${}^{+0.3}_{+ 0.4}$	${}^{+ 0.6}_{+ 0.0}$	${}^{+ 0.3}_{+ 0.3}$	${}^{+ 0.1}_{+0.3}$	${}^{-0.0}_{+ 0.1}$	${}^{+ 0.8}_{-0.4}$	∓0.6	±0.2	±0.6	±0.3	0.0	±1.4	${}^{+ 0.5}_{-0.5}$	±0.8
110–160	$0.98 ^{+ 0.01}_{-0.02}$	1.183e-01	2.8	${}^{+ 0.0}_{+0.0}$	${}^{+ 0.5}_{-0.6}$	${}^{+ 0.4}_{+ 0.2}$	${}^{+ 0.4}_{+ 0.0}$	${}^{+ 0.1}_{-0.1}$	${}^{+ 0.4}_{-0.2}$	${}^{+ 0.2}_{-0.0}$	${}^{+0.0}_{+ 0.0}$	${}^{+ 0.6}_{-0.8}$	${}^{+ 0.3}_{-0.2}$	${}^{+0.1}_{-0.1}$	${}^{+ 0.0}_{-0.0}$	${}^{+ 0.9}_{-0.7}$	∓0.0	±0.1	±1.4	±0.1	0.0	±1.4	${}^{+ 0.5}_{-0.5}$	±0.7
160–210	$0.99 ^{+ 0.01}_{-0.01}$	7.971e-02	8.3	${}^{+0.0}_{-0.0}$	${}^{-0.1}_{-0.2}$	${}^{+ 0.2}_{-0.3}$	${}^{-0.2}_{-0.2}$	${}^{+ 0.2}_{-0.5}$	${}^{+ 0.4}_{-0.3}$	${}^{-0.1}_{-0.4}$	${}^{-0.3}_{-0.1}$	${}^{+ 0.9}_{-0.7}$	${}^{+ 0.2}_{-0.8}$	${}^{-0.3}_{-0.2}$	${}^{+ 0.0}_{-0.0}$	${}^{+1.1}_{-1.2}$	∓1.5	±0.1	±0.3	±0.0	0.0	±1.4	${}^{+ 0.5}_{-0.5}$	±0.8
210–260	$1.00 ^{+ 0.01}_{-0.01}$	4.111e-02	21.	${}^{-0.0}_{+0.0}$	${}^{+ 1.2}_{-0.5}$	${}^{+ 1.0}_{-0.2}$	${}^{+ 0.8}_{+ 0.1}$	${}^{+ 1.1}_{-0.1}$	${}^{+ 1.1}_{-0.8}$	${}^{+ 0.6}_{-0.1}$	${}^{+0.3}_{+ 0.2}$	${}^{+2.1}_{-1.1}$	${}^{+ 1.2}_{-0.5}$	${}^{+ 0.2}_{+0.4}$	${}^{+ 0.0}_{-0.0}$	${}^{+2.6}_{-1.6}$	±0.6	±0.1	±0.4	±0.0	0.0	±1.4	${}^{+ 0.5}_{-0.5}$	±0.6
260–310	$1.00 ^{+ 0.01}_{-0.01}$	3.987e-02	39.	${}^{-0.0}_{+0.0}$	${}^{+1.0}_{-1.9}$	${}^{+ 0.9}_{-1.2}$	${}^{+ 0.4}_{-0.8}$	${}^{+1.3}_{-1.5}$	${}^{+ 0.8}_{-1.6}$	${}^{+ 0.4}_{-0.7}$	${}^{-0.2}_{-0.5}$	${}^{+2.7}_{-2.7}$	${}^{+1.5}_{-1.6}$	${}^{-0.2}_{-0.1}$	${}^{-0.0}_{+ 0.0}$	${}^{+3.1}_{-3.7}$	∓2.3	±0.3	±0.6	±0.1	0.0	±1.4	${}^{+ 0.5}_{-0.5}$	±0.5
310–400	$1.00 ^{+ 0.01}_{-0.01}$	2.054e-02	83.	${}^{-0.5}_{+0.5}$	${}^{+1.7}_{-2.6}$	${}^{+ 0.8}_{-1.8}$	${}^{-0.2}_{-0.1}$	${}^{+0.7}_{-1.8}$	${}^{+1.9}_{-2.4}$	${}^{+ 0.4}_{-1.0}$	${}^{-0.1}_{-0.1}$	${}^{+3.2}_{-4.9}$	${}^{+2.5}_{-3.4}$	${}^{-0.2}_{-0.4}$	${}^{-0.1}_{+0.1}$	${}^{+4.5}_{-6.6}$	∓1.3	±0.1	±1.8	±0.1	0.0	±1.4	${}^{+ 0.5}_{-0.5}$	±0.5

**Table 42:** Measured jet cross section ratio in *p*_T_ bin for anti-*k*_*t*_ jets with *R*=0.6 in the rapidity bin 1.2≤|*y*|<2.1. See caption of Table 32 for details

*p*_T_ (GeV)	NPCorr	*ρ*	$\delta_{\mathrm{stat.}}$ %	*γ*_3_ %	*γ*_9_ %	*γ*_15_ %	*γ*_21_ %	*γ*_27_ %	*γ*_31_ %	*γ*_34_ %	*γ*_40_ %	*γ*_46_ %	*γ*_52_ %	*γ*_58_ %	*γ*_64_ %	*γ*_70_ %	*γ*_78_ %	*γ*_82_ %	*γ*_74_ %	*γ*_75_ %	*γ*_83_ %	*u*_1_ %	*u*_2_ %	*u*_3_ %
20–30	$0.81 ^{+ 0.03}_{-0.17}$	2.277e-01	1.0	${}^{+ 1.8}_{-0.4}$	${}^{+ 1.0}_{-0.7}$	${}^{+ 3.0}_{-0.4}$	${}^{+ 2.9}_{+ 0.1}$	${}^{+0.7}_{-0.2}$	${}^{+1.2}_{-1.2}$	${}^{+ 0.3}_{-0.4}$	${}^{+ 0.6}_{-0.2}$	${}^{+ 0.4}_{-0.4}$	${}^{-0.0}_{+ 0.0}$	${}^{+ 0.1}_{-0.0}$	${}^{+0.2}_{-0.3}$	${}^{-0.0}_{+ 0.0}$	∓1.5	∓0.2	±0.2	±1.3	0.0	±1.4	${}^{+ 0.7}_{-0.5}$	±1.4
30–45	$0.89 ^{+ 0.02}_{-0.09}$	2.204e-01	1.9	${}^{-0.2}_{+ 0.5}$	${}^{+ 0.7}_{-0.4}$	${}^{-0.4}_{+ 0.6}$	${}^{-0.9}_{+ 0.7}$	${}^{+0.1}_{+ 0.1}$	${}^{+ 0.9}_{-0.9}$	${}^{+ 0.6}_{-0.2}$	${}^{+0.3}_{-0.1}$	${}^{+ 0.3}_{-0.4}$	${}^{+ 0.0}_{-0.0}$	${}^{+0.1}_{-0.0}$	${}^{+ 0.5}_{-0.3}$	${}^{+ 0.0}_{+ 0.0}$	±0.2	±0.1	±0.1	±0.6	0.0	±1.4	${}^{+ 0.6}_{-0.5}$	±1.1
45–60	$0.93 ^{+ 0.01}_{-0.05}$	1.783e-01	2.4	${}^{-0.7}_{+ 0.2}$	${}^{-0.2}_{-0.9}$	${}^{-0.4}_{-1.0}$	${}^{-0.4}_{-0.1}$	${}^{-0.1}_{-0.6}$	${}^{-0.2}_{-1.4}$	${}^{-0.0}_{-0.3}$	${}^{-0.1}_{-0.4}$	${}^{+ 0.1}_{-0.9}$	${}^{+ 0.0}_{-0.4}$	${}^{-0.3}_{-0.0}$	${}^{-0.1}_{-0.2}$	${}^{+ 0.0}_{-0.4}$	±0.6	±0.3	±0.3	±0.7	0.0	±1.4	${}^{+ 0.5}_{-0.5}$	±0.7
60–80	$0.95 ^{+ 0.01}_{-0.03}$	1.517e-01	1.0	${}^{-0.2}_{+ 0.1}$	${}^{+ 0.3}_{-0.3}$	${}^{-0.2}_{+ 0.3}$	${}^{-0.3}_{+ 0.2}$	${}^{+0.0}_{+ 0.1}$	${}^{+ 0.1}_{-0.2}$	${}^{+ 0.1}_{-0.1}$	${}^{-0.2}_{+0.2}$	${}^{+ 0.0}_{+ 0.1}$	${}^{+ 0.1}_{-0.2}$	${}^{+ 0.0}_{-0.1}$	${}^{-0.3}_{+ 0.3}$	${}^{+ 0.2}_{-0.3}$	∓0.8	±0.3	±0.3	±0.2	0.0	±1.4	${}^{+ 0.5}_{-0.5}$	±0.6
80–110	$0.97 ^{+ 0.01}_{-0.02}$	1.200e-01	1.5	${}^{+ 0.0}_{-0.0}$	${}^{+ 1.1}_{-0.4}$	${}^{+ 0.6}_{+ 0.3}$	${}^{+ 0.4}_{+ 0.1}$	${}^{+0.2}_{+ 0.4}$	${}^{+ 0.8}_{-0.1}$	${}^{+ 0.5}_{+ 0.2}$	${}^{+ 0.2}_{+0.4}$	${}^{+ 1.0}_{-0.2}$	${}^{+ 0.3}_{+ 0.3}$	${}^{+ 0.1}_{+ 0.4}$	${}^{-0.1}_{+ 0.1}$	${}^{+ 1.1}_{-0.2}$	∓1.2	±0.0	±0.3	±0.3	0.0	±1.4	${}^{+ 0.5}_{-0.5}$	±0.4
110–160	$0.99 ^{+ 0.01}_{-0.01}$	8.341e-02	2.7	${}^{-0.0}_{+0.0}$	${}^{+1.2}_{-1.4}$	${}^{+ 0.9}_{-0.8}$	${}^{+ 0.3}_{-0.5}$	${}^{+0.2}_{-0.4}$	${}^{+1.3}_{-1.5}$	${}^{+ 0.6}_{-0.9}$	${}^{+ 0.2}_{-0.4}$	${}^{+1.4}_{-1.7}$	${}^{+ 0.7}_{-0.7}$	${}^{+ 0.1}_{-0.0}$	${}^{+0.0}_{-0.0}$	${}^{+1.7}_{-1.7}$	∓0.6	∓0.1	±0.5	±0.0	0.0	±1.4	${}^{+ 0.5}_{-0.5}$	±0.6
160–210	$1.00 ^{+ 0.01}_{-0.01}$	5.681e-02	7.8	${}^{+0.0}_{-0.0}$	${}^{+ 0.8}_{-1.2}$	${}^{+ 0.9}_{-1.3}$	${}^{+0.3}_{-0.3}$	${}^{+ 0.6}_{-1.2}$	${}^{+1.9}_{-2.6}$	${}^{+ 0.4}_{-1.0}$	${}^{-0.2}_{-0.3}$	${}^{+2.5}_{-2.8}$	${}^{+ 0.9}_{-1.8}$	${}^{-0.3}_{-0.2}$	${}^{+ 0.0}_{-0.0}$	${}^{+2.8}_{-3.6}$	∓1.0	±0.1	±1.1	±0.0	0.0	±1.4	${}^{+ 0.5}_{-0.5}$	±0.6
210–260	$1.00 ^{+ 0.01}_{-0.01}$	3.231e-02	21.	${}^{+0.0}_{-0.0}$	${}^{+2.0}_{-1.0}$	${}^{+ 1.7}_{-0.7}$	${}^{+ 0.5}_{+0.2}$	${}^{+ 1.7}_{-0.9}$	${}^{+3.3}_{-1.9}$	${}^{+ 1.0}_{-0.6}$	${}^{+0.6}_{+ 0.1}$	${}^{+3.5}_{-2.6}$	${}^{+2.1}_{-1.2}$	${}^{+ 0.3}_{+0.3}$	${}^{+ 0.0}_{-0.0}$	${}^{+4.3}_{-3.8}$	∓2.0	±0.3	±1.1	±0.2	0.0	±1.4	${}^{+ 0.5}_{-0.5}$	±0.7
260–310	$1.01 ^{+ 0.01}_{-0.01}$	7.692e-03	79.	${}^{-0.0}_{+0.0}$	${}^{+3.3}_{-3.3}$	${}^{+2.5}_{-2.1}$	${}^{+ 1.1}_{-1.0}$	${}^{+3.0}_{-2.5}$	${}^{+4.6}_{-4.6}$	${}^{+ 1.2}_{-0.9}$	${}^{+0.5}_{-0.4}$	${}^{+5.8}_{-4.6}$	${}^{+3.9}_{-3.1}$	${}^{+ 0.0}_{-0.2}$	${}^{-0.0}_{+ 0.0}$	${}^{+7.2}_{-6.2}$	±0.0	±0.3	±1.0	±0.1	0.0	±1.4	${}^{+ 0.5}_{-0.5}$	±0.5
310–400	$1.01 ^{+ 0.01}_{-0.01}$	7.883e-03	125	${}^{-0.1}_{+0.1}$	${}^{+2.6}_{-4.0}$	${}^{+2.4}_{-3.3}$	${}^{+ 0.0}_{-1.1}$	${}^{+1.3}_{-2.8}$	${}^{+4.2}_{-6.4}$	${}^{+ 0.3}_{-1.6}$	${}^{+0.3}_{-0.2}$	${}^{+6.5}_{-8.4}$	${}^{+3.6}_{-4.6}$	${}^{-0.2}_{-0.2}$	${}^{-0.0}_{+ 0.0}$	${}^{+9.1}_{-10.}$	∓3.9	±1.0	±0.9	±0.1	0.0	±1.4	${}^{+ 0.5}_{-0.5}$	±0.5

**Table 43:** Measured jet cross section ratio in *p*_T_ bin for anti-*k*_*t*_ jets with *R*=0.6 in the rapidity bin 2.1≤|*y*|<2.8. See caption of Table 32 for details

*p*_T_ (GeV)	NPCorr	*ρ*	$\delta_{\mathrm{stat.}}$ %	*γ*_4_ %	*γ*_10_ %	*γ*_16_ %	*γ*_22_ %	*γ*_28_ %	*γ*_31_ %	*γ*_35_ %	*γ*_41_ %	*γ*_47_ %	*γ*_53_ %	*γ*_59_ %	*γ*_65_ %	*γ*_71_ %	*γ*_79_ %	*γ*_82_ %	*γ*_74_ %	*γ*_75_ %	*γ*_84_ %	*u*_1_ %	*u*_2_ %	*u*_3_ %
20–30	$0.82 ^{+ 0.03}_{-0.14}$	1.981e-01	1.6	${}^{+ 3.0}_{-0.9}$	${}^{+ 1.5}_{-0.9}$	${}^{+ 4.5}_{-0.8}$	${}^{+ 0.7}_{-0.6}$	${}^{+1.0}_{-0.2}$	${}^{+2.8}_{-2.1}$	${}^{-0.0}_{-0.5}$	${}^{+ 0.8}_{-0.3}$	${}^{+ 0.6}_{-0.5}$	${}^{-0.0}_{+ 0.0}$	${}^{+ 0.0}_{-0.0}$	${}^{-0.0}_{-0.3}$	${}^{-0.0}_{+ 0.0}$	∓2.6	±0.2	±0.1	±0.5	0.0	±1.4	${}^{+ 0.5}_{-0.5}$	±1.7
30–45	$0.90 ^{+ 0.02}_{-0.08}$	1.779e-01	1.6	${}^{-0.4}_{+ 0.6}$	${}^{+ 0.5}_{-0.4}$	${}^{-0.6}_{+ 0.3}$	${}^{+ 0.4}_{-0.5}$	${}^{-0.1}_{-0.1}$	${}^{+ 0.6}_{-2.3}$	${}^{+ 0.5}_{-0.4}$	${}^{+0.1}_{-0.1}$	${}^{+ 0.4}_{-0.6}$	${}^{+ 0.1}_{+ 0.0}$	${}^{+0.1}_{-0.0}$	${}^{+ 0.3}_{-0.4}$	${}^{+ 0.0}_{+ 0.0}$	±1.9	±0.9	±0.3	±0.6	0.0	±1.4	${}^{+ 0.5}_{-0.5}$	±1.2
45–60	$0.94 ^{+ 0.02}_{-0.04}$	1.204e-01	3.3	${}^{-1.1}_{+ 0.4}$	${}^{+ 0.8}_{-0.9}$	${}^{+ 0.4}_{-0.8}$	${}^{-0.2}_{+ 0.0}$	${}^{-0.2}_{-0.4}$	${}^{+ 0.2}_{-0.9}$	${}^{-0.2}_{-0.2}$	${}^{-0.1}_{-0.3}$	${}^{+ 0.3}_{-0.4}$	${}^{-0.1}_{-0.4}$	${}^{-0.4}_{-0.1}$	${}^{-0.4}_{-0.1}$	${}^{+ 0.1}_{-0.3}$	±0.6	±0.3	±0.5	±0.3	0.0	±1.4	${}^{+ 0.5}_{-0.5}$	±0.7
60–80	$0.96 ^{+ 0.02}_{-0.03}$	1.002e-01	1.6	${}^{-0.2}_{+ 0.2}$	${}^{+1.3}_{-1.6}$	${}^{+ 0.6}_{-0.7}$	${}^{-0.2}_{+ 0.0}$	${}^{+0.5}_{-1.0}$	${}^{+1.6}_{-1.8}$	${}^{+ 0.7}_{-1.1}$	${}^{-0.0}_{-0.2}$	${}^{+1.1}_{-1.4}$	${}^{+ 0.5}_{-0.3}$	${}^{+ 0.1}_{-0.1}$	${}^{-0.0}_{+ 0.2}$	${}^{+ 0.8}_{-1.1}$	∓0.8	±0.1	±0.5	±0.3	0.0	±1.4	${}^{+ 0.5}_{-0.5}$	±0.7
80–110	$0.98 ^{+ 0.01}_{-0.02}$	5.944e-02	2.5	${}^{+ 0.0}_{+0.0}$	${}^{+3.2}_{-2.2}$	${}^{+1.1}_{-1.0}$	${}^{+ 0.1}_{+ 0.1}$	${}^{+0.6}_{-0.3}$	${}^{+2.7}_{-1.8}$	${}^{+ 1.0}_{-0.8}$	${}^{+ 0.2}_{-0.1}$	${}^{+2.4}_{-1.9}$	${}^{+ 0.5}_{-0.3}$	${}^{+ 0.1}_{+ 0.3}$	${}^{-0.1}_{+ 0.1}$	${}^{+2.3}_{-2.2}$	∓1.9	±0.2	±0.6	±0.2	0.0	±1.4	${}^{+ 0.5}_{-0.5}$	±1.0
110–160	$1.00 ^{+ 0.01}_{-0.01}$	2.368e-02	7.4	${}^{+0.0}_{-0.0}$	${}^{+4.1}_{-3.5}$	${}^{+3.0}_{-2.1}$	${}^{+ 0.8}_{-0.5}$	${}^{+ 1.1}_{-0.6}$	${}^{+4.2}_{-3.5}$	${}^{+2.4}_{-2.1}$	${}^{+1.2}_{-0.6}$	${}^{+4.5}_{-4.1}$	${}^{+1.6}_{-1.9}$	${}^{+ 0.2}_{-0.2}$	${}^{-0.0}_{+ 0.0}$	${}^{+5.1}_{-4.1}$	∓2.5	±0.7	±0.9	±0.0	0.0	±1.4	${}^{+ 0.5}_{-0.5}$	±0.8
160–210	$1.01 ^{+ 0.02}_{-0.01}$	8.807e-03	35.	${}^{+0.0}_{-0.0}$	${}^{+2.0}_{-2.4}$	${}^{+2.6}_{-1.6}$	${}^{+ 0.6}_{-0.8}$	${}^{+1.5}_{-1.8}$	${}^{+3.9}_{-4.3}$	${}^{+2.2}_{-1.3}$	${}^{+0.1}_{-0.4}$	${}^{+5.8}_{-5.1}$	${}^{+3.0}_{-2.4}$	${}^{-0.2}_{-0.3}$	${}^{-0.0}_{+ 0.0}$	${}^{+6.3}_{-6.4}$	∓1.9	±1.1	±2.1	±0.1	0.0	±1.4	${}^{+ 0.5}_{-0.5}$	±0.8
210–260	$1.01 ^{+ 0.02}_{-0.01}$	5.215e-03	115	0.0	${}^{+8.3}_{-8.2}$	${}^{+5.3}_{-8.4}$	${}^{+ 0.5}_{-2.6}$	${}^{+5.0}_{-6.6}$	${}^{ +15}_{ -13}$	${}^{+1.4}_{-3.9}$	${}^{+0.6}_{-0.7}$	${}^{ +19}_{ -12}$	${}^{+8.7}_{-8.5}$	${}^{+ 0.1}_{+ 0.8}$	${}^{+ 0.0}_{-0.0}$	${}^{ +24}_{ -15}$	∓9.0	∓0.0	±2.0	±0.1	0.0	±1.4	${}^{+ 0.5}_{-0.5}$	±0.8

**Table 44:** Measured jet cross section ratio in *p*_T_ bin for anti-*k*_*t*_ jets with *R*=0.6 in the rapidity bin 2.8≤|*y*|<3.6. See caption of Table 32 for details

*p*_T_ (GeV)	NPCorr	*ρ*	$\delta_{\mathrm{stat.}}$ %	*γ*_5_ %	*γ*_11_ %	*γ*_17_ %	*γ*_23_ %	*γ*_29_ %	*γ*_31_ %	*γ*_36_ %	*γ*_42_ %	*γ*_48_ %	*γ*_54_ %	*γ*_60_ %	*γ*_66_ %	*γ*_72_ %	*γ*_80_ %	*γ*_82_ %	*γ*_74_ %	*γ*_75_ %	*γ*_85_ %	*u*_1_ %	*u*_2_ %	*u*_3_ %
20–30	$0.84 ^{+ 0.03}_{-0.12}$	1.432e-01	1.9	${}^{+ 2.7}_{-0.9}$	${}^{+1.3}_{-1.3}$	${}^{+4.5}_{-1.1}$	${}^{-1.2}_{+1.5}$	${}^{+0.9}_{-0.4}$	${}^{+2.5}_{-4.0}$	${}^{+ 0.3}_{-0.5}$	${}^{+ 0.8}_{-0.4}$	${}^{+ 0.6}_{-0.5}$	${}^{-0.0}_{+ 0.0}$	${}^{+ 0.1}_{-0.0}$	${}^{+0.1}_{-0.4}$	${}^{-0.0}_{+ 0.0}$	∓3.5	±0.3	±0.1	±0.6	0.0	±2.2	${}^{+ 0.5}_{-0.5}$	±5.5
30–45	$0.91 ^{+ 0.03}_{-0.08}$	1.032e-01	2.4	${}^{-0.2}_{-0.8}$	${}^{+1.8}_{-2.1}$	${}^{+ 0.3}_{-1.0}$	${}^{-2.7}_{+2.1}$	${}^{+0.4}_{-0.6}$	${}^{+5.2}_{-6.8}$	${}^{+ 1.0}_{-0.9}$	${}^{+ 0.6}_{-0.4}$	${}^{+ 0.6}_{-1.1}$	${}^{+ 0.1}_{-0.0}$	${}^{+ 0.1}_{-0.0}$	${}^{+0.9}_{-0.7}$	${}^{+ 0.1}_{-0.0}$	∓0.3	∓0.1	±0.5	±0.8	0.0	±2.2	${}^{+ 0.5}_{-0.5}$	±3.9
45–60	$0.95 ^{+ 0.03}_{-0.05}$	5.089e-02	3.8	${}^{-0.6}_{+ 1.2}$	${}^{+6.0}_{-2.9}$	${}^{+5.1}_{-2.0}$	${}^{-0.5}_{+ 3.4}$	${}^{+0.9}_{-0.6}$	${}^{+10.}_{-9.5}$	${}^{+ 2.0}_{-0.8}$	${}^{+ 0.6}_{-0.0}$	${}^{+3.7}_{-1.7}$	${}^{+ 0.6}_{-0.4}$	${}^{-0.3}_{-0.0}$	${}^{+0.6}_{+ 0.2}$	${}^{+ 1.0}_{-0.5}$	∓2.6	±0.5	±3.1	±0.4	0.0	±1.4	${}^{+ 0.5}_{-0.5}$	±2.1
60–80	$0.97 ^{+ 0.03}_{-0.03}$	2.057e-02	3.6	${}^{-0.8}_{+ 0.9}$	${}^{+4.2}_{-5.1}$	${}^{+1.4}_{-3.0}$	${}^{-1.9}_{+ 0.8}$	${}^{+1.7}_{-2.9}$	${}^{+9.1}_{-11.}$	${}^{+1.6}_{-2.7}$	${}^{-0.3}_{-1.2}$	${}^{+2.9}_{-5.0}$	${}^{-0.0}_{-0.8}$	${}^{-0.0}_{-0.1}$	${}^{-0.9}_{+ 0.3}$	${}^{+1.8}_{-3.3}$	∓3.6	±0.6	±5.5	±0.5	0.0	±1.4	${}^{+ 0.5}_{-0.5}$	±4.1
80–110	$0.99 ^{+ 0.03}_{-0.02}$	4.221e-03	12.	${}^{-0.3}_{+ 0.2}$	${}^{+4.5}_{-8.3}$	${}^{+ 0.3}_{-3.6}$	${}^{-0.6}_{-0.3}$	${}^{+0.6}_{+ 1.0}$	${}^{+6.5}_{-13.}$	${}^{+ 0.5}_{-2.1}$	${}^{-0.1}_{+0.5}$	${}^{+4.7}_{-6.8}$	${}^{+ 0.4}_{-0.1}$	${}^{+ 0.1}_{+ 0.5}$	${}^{-0.4}_{+ 0.4}$	${}^{+4.8}_{-6.6}$	∓0.9	±2.3	±2.3	±0.2	0.0	±1.4	${}^{+ 0.5}_{-0.5}$	±4.9
110–160	$1.00 ^{+ 0.04}_{-0.02}$	8.403e-04	71.	${}^{-0.0}_{+0.0}$	${}^{+2.7}_{-20.}$	${}^{+1.4}_{-17.}$	${}^{-3.2}_{-13.}$	${}^{-2.6}_{-11.}$	${}^{+8.9}_{-38.}$	${}^{-0.4}_{-16.0}$	${}^{-1.9}_{-12.}$	${}^{+3.9}_{-21.}$	${}^{+2.3}_{-13.}$	${}^{+0.0}_{-0.0}$	${}^{-0.1}_{+ 0.2}$	${}^{+ 0.7}_{-21.8}$	∓22	±0.5	±25.5	±0.0	0.0	±1.4	${}^{+0.5}_{-0.5}$	±3.1

**Table 45:** Measured jet cross section ratio in *p*_T_ bin for anti-*k*_*t*_ jets with *R*=0.6 in the rapidity bin 3.6≤|*y*|<4.4. See caption of Table 32 for details

*p*_T_ (GeV)	NPCorr	*ρ*	$\delta_{\mathrm{stat.}}$ %	*γ*_6_ %	*γ*_12_ %	*γ*_18_ %	*γ*_24_ %	*γ*_30_ %	*γ*_31_ %	*γ*_37_ %	*γ*_43_ %	*γ*_49_ %	*γ*_55_ %	*γ*_61_ %	*γ*_67_ %	*γ*_73_ %	*γ*_81_ %	*γ*_82_ %	*γ*_74_ %	*γ*_75_ %	*γ*_86_ %	*u*_1_ %	*u*_2_ %	*u*_3_ %
20–30	$0.84 ^{+ 0.08}_{-0.03}$	5.789e-02	4.6	${}^{+10.}_{-3.6}$	${}^{+6.8}_{-4.3}$	${}^{+12.}_{-4.7}$	${}^{+ 2.5}_{-0.6}$	${}^{+3.7}_{-0.7}$	${}^{ +22}_{ -23}$	${}^{+ 2.4}_{-1.0}$	${}^{+ 3.3}_{-0.8}$	${}^{+3.3}_{-1.3}$	${}^{+ 0.0}_{+ 0.0}$	${}^{+ 0.0}_{-0.0}$	${}^{+2.6}_{-0.9}$	${}^{+ 0.0}_{+ 0.0}$	∓10	±1.3	±1.4	±0.7	0.0	±2.2	${}^{+ 0.5}_{-0.5}$	±6.5
30–45	$0.89 ^{+ 0.09}_{-0.03}$	1.603e-02	5.1	${}^{+9.8}_{-5.5}$	${}^{ +15}_{ -10}$	${}^{+12.}_{-8.6}$	${}^{+5.1}_{-2.5}$	${}^{+5.0}_{-3.3}$	${}^{ +58}_{ -35}$	${}^{+6.5}_{-4.0}$	${}^{+5.5}_{-4.0}$	${}^{+7.4}_{-5.3}$	${}^{+ 0.1}_{-0.0}$	${}^{+ 0.1}_{+0.0}$	${}^{+6.1}_{-4.5}$	${}^{+ 0.1}_{-0.1}$	∓7.6	±1.7	±5.4	±1.7	0.0	±1.4	${}^{+ 0.5}_{-0.5}$	±11.5
45–60	$0.90 ^{+ 0.12}_{-0.08}$	1.109e-03	16.	${}^{-2.3}_{-0.9}$	${}^{ +24}_{ -12}$	${}^{+4.4}_{-12.}$	${}^{+ 0.7}_{-1.2}$	${}^{+2.4}_{-5.7}$	${}^{ +55}_{ -46}$	${}^{+1.9}_{-7.8}$	${}^{+1.9}_{-4.7}$	${}^{+5.8}_{-11.}$	${}^{-0.6}_{-1.1}$	${}^{-0.7}_{+0.0}$	${}^{+2.0}_{-4.3}$	${}^{+1.8}_{-3.9}$	∓24	±12.4	±2.2	±0.7	0.0	±1.4	${}^{+ 0.5}_{-0.5}$	±6.5

## Footnotes

1 Rapidity is defined as *y*=0.5ln[(*E*+*p*_z_)/(*E*−*p*_z_)] where *E* denotes the energy and *p*_z_ is the component of the momentum along the beam direction.

2 ATLAS uses a right-handed coordinate system with its origin at the nominal interaction point (IP) in the centre of the detector and the *z*-axis along the beam pipe. The *x*-axis points from the IP to the centre of the LHC ring, and the *y*-axis points upward. The pseudorapidity is defined in terms of the polar angle *θ* as *η*=−lntan(*θ*/2).

3 The origin of these fluctuations are rare event topologies in gluon emissions *q*→*qg* and gluon splittings $g \rightarrow q \bar{q}$, related to the fact that by default Powheg Box 1.0 does not consider the corresponding configurations with opposite ordering of the *p*_T_ for the final state parton: *q*→*gq* and $g \rightarrow\bar{q}q$. These processes can be activated in revision 2169 using the Powheg option doublefsr = 1, which offers an improved handling of the suppression of these events. More details are given in Ref. [66].

4 The electromagnetic scale is the basic calorimeter signal scale for the ATLAS calorimeter. It has been established using test-beam measurements for electrons and muons to give the correct response for the energy deposited in electromagnetic showers, but it does not correct for the lower response of the calorimeter to hadrons.

5 As written in Sect. 9, the measurement at $\sqrt{s}=2.76~\mbox{TeV}$ uses a quality selection for jets with low *p*_T_ in Monte Carlo simulation at |*η*|∼1, which is a different treatment than was done for the published measurement at $\sqrt{s}=7~\mbox{TeV}$ [25]. The ratio is extracted using the coherent treatment in the two measurements at the different beam energies, shifting the measured cross-section at $\sqrt{s}=7~\mbox{TeV}$ from the published result within its uncertainty. The shifts are sizable in the bin 0.8≤|*y*|<1.2 only. For jets with *R*=0.4 (*R*=0.6), they are 13 % (10 %) in the 20≤*p*_T_<30 GeV bin, and 1.5 % (2.6 %) in the 30≤*p*_T_<45 GeV bin. In the rapidity range 1.2≤|*y*|<2.1, the shift is 1.8 % (1.9 %) at 20≤*p*_T_<30 GeV. These bins in the $\sqrt{s}=7~\mbox{TeV}$ measurement only enter in the extraction of *ρ*(*y*,*p*_T_) and not in that of *ρ*(*y*,*x*_T_).
